# Large Scale Gene Expression Profiles of Regenerating Inner Ear Sensory Epithelia

**DOI:** 10.1371/journal.pone.0000525

**Published:** 2007-06-13

**Authors:** R. David Hawkins, Stavros Bashiardes, Kara E. Powder, Samin A. Sajan, Veena Bhonagiri, David M. Alvarado, Judith Speck, Mark E. Warchol, Michael Lovett

**Affiliations:** 1 Division of Human Genetics, Department of Genetics, Washington University School of Medicine, St. Louis, Missouri, United States of America; 2 Department of Otolaryngology, Washington University School of Medicine, St. Louis, Missouri, United States of America; Texas A&M University, United States of America

## Abstract

Loss of inner ear sensory hair cells (HC) is a leading cause of human hearing loss and balance disorders. Unlike mammals, many lower vertebrates can regenerate these cells. We used cross-species microarrays to examine this process in the avian inner ear. Specifically, changes in expression of over 1700 transcription factor (TF) genes were investigated in hair cells of auditory and vestibular organs following treatment with two different damaging agents and regeneration *in vitro*. Multiple components of seven distinct known signaling pathways were clearly identifiable: *TGFβ, PAX, NOTCH, WNT, NFKappaB, INSULIN/IGF1* and *AP1*. Numerous components of apoptotic and cell cycle control pathways were differentially expressed, including *p27^KIP^* and TFs that regulate its expression. A comparison of expression trends across tissues and treatments revealed identical patterns of expression that occurred at identical times during regenerative proliferation. Network analysis of the patterns of gene expression in this large dataset also revealed the additional presence of many components (and possible network interactions) of estrogen receptor signaling, circadian rhythm genes and parts of the polycomb complex (among others). Equal numbers of differentially expressed genes were identified that have not yet been placed into any known pathway. Specific time points and tissues also exhibited interesting differences: For example, 45 zinc finger genes were specifically up-regulated at later stages of cochlear regeneration. These results are the first of their kind and should provide the starting point for more detailed investigations of the role of these many pathways in HC recovery, and for a description of their possible interactions.

## Introduction

The human cochlea possesses approximately 16,000 sensory hair cells (HCs), which are necessary for normal hearing [1]. These cells are mechanoreceptors that detect sound, head movements and acceleration, and provide input into the auditory and vestibular branches of the eighth cranial nerve. Hair cells in the mammalian ear are produced during a fixed period of embryonic development, and can be lost later in life as a consequence of acoustic trauma, exposure to ototoxic drugs or inner ear infections. The mammalian vestibular organs possess a very limited ability for sensory regeneration [2], [3] and the mammalian cochlea is completely unable to regenerate hair cells [4]. As a result, the loss of sensory cells from the mammalian ear can result in permanent deficits in hearing and balance. Such disorders are very common; so-called ‘sensorineural’ hearing loss afflicts about 30 million Americans [1].

Sensory epithelia (SE) in the ears of all vertebrates are populated by two basic cell types: hair cells and supporting cells. The key limitation to regeneration in the mammalian ear is the inability of mammalian supporting cells to proliferate or change phenotype in response to hair cell injury. Several attempts have been made to induce regeneration in the vestibular organs by exposure to exogenous mitogens, but these have had limited success [5] [6]. More recently, viral gene transfer techniques have been used to transfect supporting cells in the damaged cochlea with the gene encoding the *Atoh1* transcription factor. This factor, formerly known as *Math1*, is required for normal hair cell differentiation [7] and also appears to be capable of converting the phenotype of mature supporting cells into replacement hair cells. These new sensory receptors can also re-establish synaptic connections with afferent neurons, leading to a moderate restoration of hearing in a guinea pig model of hearing loss [8] [9].

Unlike mammals, spontaneous regeneration of sensory hair cells has been extensively documented in the hearing and balance organs of non-mammalian vertebrates. The avian inner ear, in particular, has a very robust capacity for sensory regeneration. Death of hair cells quickly triggers renewed proliferation of epithelial supporting cells, and the progeny of these divisions can differentiate as replacement hair cells and supporting cells [10], [11]. These observations have led to the hope that understanding the biological basis of sensory regeneration in the avian ear might provide insights into how to induce similar repair in humans. Relatively little is known about the genetic regulatory mechanisms that permit regeneration in the avian ear and this has limited the development of strategies for inducing such regeneration in mammals. Previous studies of the avian regenerative process have identified genes on an individual (and essentially “candidate gene”) basis (e.g. *DELTA1*-[12]; *FGFR3*-[13]; *PROX1*-[14]).

In this study we employed two genomic technologies to obtain a more global picture of gene expression changes during avian hair cell regeneration. Specifically, we used micro cDNA amplification methods and custom gene microarrays [15] [16] to interrogate nearly all identified transcription factor (TF) genes in the SE of the chick cochlea and utricle during the early phases of regeneration. We identified activity within known signaling pathways, several of which have not been previously implicated or explored in SE regeneration, and we identified many as-yet unexplored genes that were differentially expressed. We were able to identify treatment, tissue and time point-specific changes in TF expression. Notably, a basal set of TFs was present in both sensory tissues at all stages. We also identified commonalities and tissue-specific differences between the sensory epithelia of the utricle and cochlea that resulted from different damage regimes. This is the first study of its kind to characterize large-scale gene expression profiles during sensory regeneration. These results now provide the starting point for a detailed investigation of the role of these genes in HC recovery, and for a description of their possible interactions.

## Results

### Study design

We profiled changes in transcription factor expression in SE of the cochlea and utricle following two distinct forms of *in vitro* injury: (1) laser ‘wounding’ of cultured SE or; (2) ototoxic hair cell death caused by treatment with the aminoglycoside antibiotic neomycin. In the first case, cultured SE [17] received linear ‘wounds’ with a pulsed laser microbeam. Creation of the lesion typically required 3–5 min/culture; during this time, control cultures were removed from the incubator and kept under identical conditions, but did not receive lesions. Wounded epithelia were allowed to recover for 30 min, 1 hr, 2 hrs or 3 hrs after the lesions. Equal numbers of lesioned and unlesioned specimens were analyzed at each recovery time point. For the second injury regimen, utricles or cochleae were cultured for 24 hr in medium that contained 1 mM neomycin [18]. A sample of SE was collected immediately after this treatment; this constituted the 0 hr time point for the regenerative time course. Other cultures were rinsed and maintained in neomycin-free medium for an additional 24 or 48 hr. Equal numbers of specimens were cultured under identical conditions, but did not receive neomycin; these served as time-matched controls for comparative gene expression profiling.

Hybridizations were conducted on multiple biological samples (see below). The sampling times for neomycin-lesioned specimens were chosen based upon previous studies of hair cell death and supporting cell proliferation, which indicate that S-phase entry by supporting cells peaks at about 48 hrs after ototoxic injury *in vitro* [19]. On the other hand, the laser time points were expected to provide us with insights into the very early signaling events that occur after epithelial injury. Our expectation in employing these two damage regimes was that we might find some degree of overlap between the two time courses, but that we might also be able to discern treatment-specific, as well as cochlea- and utricle-specific, changes in gene expression.

After appropriate survival times, RNA was prepared and converted into microarray targets by previously described methods [15]. TF gene expression was then assayed by comparative hybridization (injured specimens vs. time-matched controls) to custom transcription factor gene microarrays [16]. These microarrays contained 50 mer oligonucleotide probes, spotted in duplicate, that interrogate the vast majority of human transcription factor genes (plus a few probes to non-TFs such as sonic hedgehog [SHH]). We used this array as a cross-species profiling tool to measure the expression of the orthologous chicken TF genes. We, and others, have previously demonstrated that cross-species hybridizations can be reliably used on this type of array platform [15] [20]. The study described here was embarked upon before the recent publication of the draft chicken genomic DNA sequence [21] or the availability of commercial chicken gene chips. With the release of most of the chicken genomic DNA sequence it is possible to assess sequence identity between our human probes and their chicken orthologs. An analysis of this type indicates that ∼98% of our probes have >70% sequence identity with the correct chicken ortholog (data not shown). Hybridization stringencies were adjusted to accommodate this degree of identity for all of the comparative data described in this study. Each time course was replicated with additional biological samples, including controls. Every treatment time point and time-matched control was hybridized to a minimum of four microarrays; two replicates plus two dye switch experiments. All of our data, array designs and analysis parameters are available through the NCBI Gene Expression Omnibus (GEO; http://www.ncbi.nlm.nih.gov/geo/).

### Array analysis

In order to quantify gene expression changes, along with associated statistical confidence limits, all expression data were analyzed as described below (see Materials and Methods). Briefly, array data were first normalized by LOWESS, a locally weighted linear regression model, to compensate for dye effects. Second, data from the multiple hybridizations for a given time point were hierarchically clustered, along with data from additional time points, to assess the similarity and reproducibility of the data across multiple biological samples and dye switches. T-tests (calculated by conducting a one sample t-test on the adjusted intensity data of the entire array of selected hybridizations at each time point) were performed between samples to assess reproducibility and similarity. The data were also filtered to remove oligonucleotide probes that fell below a threshold for background intensity. This threshold was determined by the intensity of controls spots. During the creation of self-organizing maps (see below), not all genes passed the filtering steps in both time courses. In these cases we extracted the missing values from the primary data and “filled in” the values to construct the patterns of gene expression across all seven time points. In general the vast majority of TFs showed relatively modest gene expression fold changes. This may be due to a compression of the dynamic range in cross-species hybridizations [15]. Our prior experience in employing this array platform for cross-species hybridizations indicated that changes as low as 1.2-fold frequently reflected higher changes when assessed by q-PCR ([15]and see q-PCR below).

### Differential gene expression in the four time courses

In the antibiotic damage regime the 24 and 48 hr time points reflected gene expression changes within supporting cells, as the majority of hair cells had been killed by the ototoxic antibiotic [18], [22]. By 48 hrs many of the supporting cells had progressed into the S-phase of the cell cycle [19]. By contrast, the laser damage regime resulted in a 100–200 µm-wide ‘wound’ in the cultured sensory epithelia. The wounds typically closed within 16–24 hrs of recovery time. The initial phase of wound repair was due to cell migration, but elevated levels of cell proliferation were also observed at the wound sites (but not at distant, uninjured regions) at 16–48 hrs after injury. For the utricle, after the data analysis steps described above, 143 TFs had passed through the data filters for differential gene expression (>1.2-fold change at one or more time point and a p-value of <0.05) over the three neomycin damage time points (Supplemental Tables S1 and S2). Gene expression in laser damaged SE was compared to time-matched controls at 30 min, 1 hr, 2 hrs, and 3 hrs after laser lesions. For the utricle, a total of 66 TFs were differentially expressed across the four laser time points (Supplemental Tables S3 and S4).

Analysis of the cochlear treatments revealed a much larger number of significant changes in TF gene expression than were found for the utricle. A total of 484 genes were differentially expressed (>1.2-fold change and p-value of <0.05) across the cochlear neomycin time course (Supplemental Tables S5 and S6). Analysis of the cochlear laser comparisons revealed a total of 217 differentially expressed genes (listed in Supplemental Tables S7 and S8). Overall, when overlaps between the various lists of genes were taken into account, a total of 605 TFs accounted for all of the statistically significant changes in gene expression observed across the two cochlear time courses, and a total of 188 TF genes were differentially expressed across the two utricle time courses. It is possible that these apparent differences in numbers of differentially expressed genes between the two epithelia, reflect more synchronization of regenerative signaling events in the cochlea when compared to undamaged controls. It is notable that the undamaged avian utricle is in a continual low-level state of hair cell turn-over [23]. This process may result in asynchronies in gene expression between injured and uninjured utricles. This might lower apparent fold-changes or increase variability (leading to higher p-values) when the damaged utricles are compared to the undamaged (but constantly regenerating) utricles. It is also possible that the larger number of expression changes in the cochlea reflects a more robust regenerative program in this particular sensory epithelia.

### Validation of expression changes

To independently verify apparent differences in gene expression we selected 11 genes that showed modest fold-changes for at least one time point within at least one of the four regenerative time courses. The orthologous chicken genes were identified by BLAST searches and PCR primers were designed for use in qPCR assays on new biological samples. In most cases these assays were conducted upon tissues from single time points. However, for three genes (*CTNNB1*, *KIAA0173* and *TRIP15*) the genes were tested across one entire time course (the utricle neomycin time course). A total of 19 time point/tissue/treatment combinations were tested overall. The results of these multiple qPCR assays are shown in Table 1 and compared to the apparent fold-changes observed from the array analysis. As observed in our previous study with this type of cross-species array platform [15], these data confirmed the trends seen in the microarray data and indicated that the microarray, in general, under-reported the actual levels of changes that occurred across the four time courses. The only example showing >15% deviation from this under reporting was the level of *CTNNB1* mRNA in the utricle at 48 hrs after neomycin damage. In this case, the q-PCR assay reported an increase of 2.3 fold relative to undamaged utricle, whereas the array reported a 3.1-fold increase.

**Table 1:** QPCR validation of microarray fold-changes.

Tissue	Treatment	Timepoint	Gene Name	Array Fold Change	QPCR Fold Change
Utricle	NEOMYCIN	0 HRS	CEBPG	−1.55	−2.78
Utricle	NEOMYCIN	0 HRS	CTNNB1	−1.27	−1.43
Utricle	NEOMYCIN	24 HRS	CTNNB1	−1.12	−1.25
Utricle	NEOMYCIN	48 HRS	CTNNB1	3.09	2.27
Utricle	NEOMYCIN	0 HRS	KIAA0173	1.36	1.69
Utricle	NEOMYCIN	24 HRS	KIAA0173	1.07	1.02
Utricle	NEOMYCIN	48 HRS	KIAA0173	1.29	1.60
Utricle	NEOMYCIN	0 HRS	TRIP15	−1.25	−1.60
Utricle	NEOMYCIN	24 HRS	TRIP15	1.02	1.20
Utricle	NEOMYCIN	48 HRS	TRIP15	1.41	1.30
Utricle	NEOMYCIN	48 HRS	BCL11A	1.33	2.22
Utricle	NEOMYCIN	48 HRS	CUTL1	1.38	1.30
Cochlea	NEOMYCIN	0 HRS	TRIP15	−1.47	−1.25
Cochlea	NEOMYCIN	48 HRS	JUND	−1.36	−1.66
Cochlea	LASER	30 MINS	FOS	1.50	12.50
Cochlea	LASER	1 HRS	NFIL3	1.32	1.8
Cochlea	LASER	1 HRS	IRLB	1.29	2.5
Cochlea	LASER	2 HRS	GATA3	−1.29	−1.42
Cochlea	LASER	3 HRS	JUND	1.31	2.4

Triplicate qPCR assays were carried out on new biological samples, using PCR primers designed to the orthologous chicken gene sequences. These are compared to the apparent fold-changes observed from the array analysis.

### Comparing and contrasting differential gene expression across tissues and treatments

**Figure 1:**
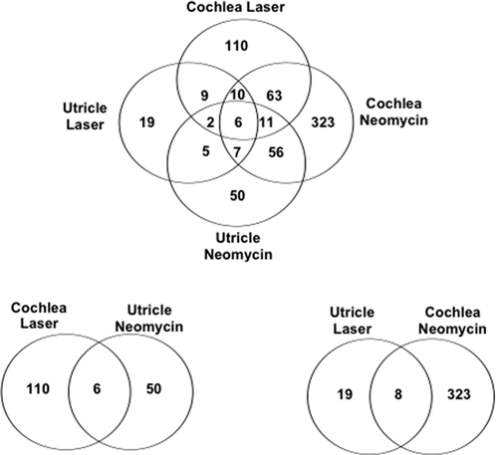
Overlaps between differentially expressed genes in the four treatment/time course combinations. These Venn diagrams illustrate the overlaps between genes that were scored as being either up or down regulated. To be included as an overlap a gene must be differentially expressed in at least one time point in both time courses (>1.2-fold and p-value< = 0.05), but the time points or trends do not have to necessarily match. For a detailed listing of all of the genes in the overlaps see Supplementary Tables S2, S4, S6 and S8).

Despite the large differences in the sheer volume of changes in the two regenerating epithelia, there were interesting underlying overlaps between the various transcriptional programs. These are shown in summary form as the Venn diagrams in Figure 1. For example, a comparison of all changes in gene expression between both neomycin time courses (utricle and cochlea) revealed 80 shared genes, of which 21 showed identical trends in at least one identical time point. These time point commonalities are shown in Table 2 and in a more expanded form in Supplementary Table S9. The largest area of overlap between the cochlea and utricle was at 48 hrs where 11 genes showed identical patterns of gene expression (Table 2). Four of these were down-regulated and seven were up-regulated versus the time-matched controls, but literature searches and pathway analysis did not reveal any known unifying functional network that ties these together. Among this group were; *CTNNB1* (a component of canonical *WNT* signaling [24]) *NR1H3* (a.k.a. *LXRalpha*), an orphan nuclear receptor that is known to play a role in insulin regulation of cholesterol homeostasis in hepatocytes [25], [26] and; *BCL11A* (a zinc finger gene associated with various hematopoietic malignancies [27], [28]). Antibiotic damage in the avian utricle results in a program of cell division that peaks at 48 hrs after ototoxic injury [29] and returns to normal levels at 4 days post injury. A recent study [30] also indicates that markers of hair cell differentiation are expressed in the SE at >80 hrs after ototoxic injury. Therefore, the pattern of expression of these 11 genes may mark a period at which the proliferative program is declining and the differentiation program is initiating within the regenerating SE.

**Table 2:** Genes that exhibit identical patterns of expression in at least one identical time-point.

UTR-NEO			COCH-NEO			UTR-LSR				COCH-LSR				
0 hr	24 hr	48 hr	0 hr	24 hr	48 hr	30 min	1 hr	2 hr	3 hr	30 min	1 hr	2 hr	3 hr	GENE
−1	0	0	−1	0	0	0	1	0	0	0	0	0	−1	COPS2
−1	0	0	−1	0	−1	–	–	–	–	–	–	–	–	GTF2F1
−1	0	0	−1	−1	−1	0	0	0	0	–	–	–	–	IRF2
−1	0	0	−1	0	0	–	–	–	–	–	–	–	–	FOXJ3
−1	0	0	−1	0	−1	–	–	–	–	–	–	–	–	NFE2L1
1	1	1	1	0	0	−1	0	0	1	0	0	0	0	ZNF93
1	0	0	1	0	1	0	0	0	0	–	–	–	–	ZNF90
0	−1	−1	0	−1	0	0	0	0	1	–	–	–	–	ZNF44
0	−1	0	0	−1	0	0	0	0	0	−1	0	0	0	TITF1
1	−1	0	0	−1	0	0	0	0	0	0	−1	0	0	MAPK8IP1
0	0	−1	0	−1	−1	0	0	0	0	–	–	–	–	TAF10
0	0	−1	0	0	−1	0	1	0	0	0	1	0	0	NR1H3
0	0	−1	0	0	−1	0	0	0	1	–	–	–	–	FHL1
0	1	−1	0	0	−1	0	0	0	0	–	–	–	–	FOXA1
0	0	1	0	−1	1	0	0	0	1	–	–	–	–	MYCBP
0	0	1	0	0	1	0	0	0	1	0	0	0	1	BCL11A
0	0	1	0	0	1	–	–	–	–	–	–	–	–	CTNNB1
0	0	1	0	0	1	–	–	–	–	0	0	0	0	ZNF324
1	0	1	0	0	1	0	0	0	1	–	–	–	–	TBX15
1	0	1	0	0	1	0	0	0	0	–	–	–	–	ZNF79
1	0	1	0	1	1	0	0	0	1	0	0	0	1	ZNF248
–	–	–	1	0	0	−1	0	0	1	−1	0	0	0	ZBTB6
–	–	–	–	–	–	1	0	0	0	1	0	1	0	C1orf142
0	0	−1	0	0	−1	0	1	0	0	0	1	0	0	NR1H3
–	–	–	0	−1	0	0	1	−1	0	0	1	0	0	MYT2
0	0	0	0	−1	−1	0	0	0	1	0	0	0	1	JUND
1	0	0	−1	0	0	0	0	0	1	0	0	0	1	TTLL4
1	0	0	0	1	0	0	0	0	1	0	0	0	1	HOXD8
0	0	1	0	0	1	0	0	0	1	0	0	0	1	BCL11A
1	0	1	0	1	1	0	0	0	1	0	0	0	1	ZNF248
–	–	–	1	0	0	0	0	0	1	0	0	0	1	ZNF75A
–	–	–	0	0	1	0	0	0	1	0	0	0	1	C14orf106
–	–	–	0	0	1	0	0	0	1	0	0	0	1	TBX5

Each time course and treatment combination is shown across the top.rows of the table: Utr-neo = utricle neomycin; Coch-lsr = cochlea laser and so on. Specific genes that exhibit similar patterns of gene expression are shown down the right hand column. Shaded entries illustrate similar patterns. 1 = up-regulation, 0 = no change and −1 = down-regulation relative to untreated controls. All values are derived from Supplementary Tables S2, S4, S6 and S8 and are >1.2-fold and p-value< = 0.05.

A similar set of comparisons were conducted on all the differentially expressed TFs in the laser time courses (Figure 1 and Table 2). These shared 27 differentially expressed genes, of which 12 showed the same trends for at least one parallel time point (Table 1). These changes might either reflect laser-specific responses or be early-acting effects in the common pathway of hair cell regeneration. It is interesting to note that the 3 hr laser time points shared two up-regulated genes in common with the 48 hr neomycin time points (*BCL11A* and another zinc finger gene, *ZNF248*). The last laser time points also showed up-regulation of five additional genes [*JUND, TTLL4* (a.k.a. *KIAA0173*) *HOXD8* and *ZNF75A*].

A comparison of trends within a given SE (irrespective of the method of injury) revealed 5 genes (*CEBPG, HSF1, LOC51131, PSMC5* and *SSX4*) that appear to reflect regenerative responses specific to the utricle (i.e. they were not differentially expressed in the regenerating cochlea SE). A similar comparison of trends in the cochlea identified 90 genes that occurred in both laser and neomycin lists of differentially expressed genes (Figure 1). In this case, 61 of these gene expression changes appeared to be specific to the regenerating cochlea (cf Supplementary Tables S2, S4, S6 and S8). Among these were two forkhead genes (*FOXC1* and *FOXH1*) three homeobox genes (*HOXA4, HOXC10* and *HOXD1*) five zinc finger transcription factor genes (*ZFHX1b, ZFP106, ZNF123, ZNF175* and *ZNF258*) and three genes from the circadian rhythm pathway (*PER1, PER2* and *TIMELESS*). Several of these will be discussed in more detail below. None of these genes has been previously investigated in the regenerating or developing inner ear.

Notably, we also identified a core group of six genes [*BCL11A, HOXD8, TTLL4, ZNF248* (a.k.a. *LOC57209*), *NR1H3*, and *COPS2* (a.k.a. *TRIP15*)] that change their expression levels across all four treatments and tissue combinations. It should be noted that although these genes vary across all of the time courses, they do not necessarily follow exactly parallel patterns of gene expression across time courses. Thus, *COPS2* was initially down regulated in both neomycin time courses, was up-regulated at 1 hr after laser treatment in the utricle, but was down-regulated at 3 hrs after laser treatment in the cochlea. By contrast, some genes such as *BCL11A,* appeared quite consistent between the four combinations; *BCL11A* expression peaked at 3hrs in both laser time courses and peaked at 48 hrs in both neomycin time courses.

Beyond these various overlaps between treatments and time courses, the largest group of gene expression changes in each of the four treatment/time point combinations were specific to just one SE and one treatment. For example, changes in the expression of 323 TF genes were specific to cochlea SE that was recovering from antibiotic damage (Figure 1). Many of these changes (for example the large group of zinc finger genes mentioned below) peaked at the 48 hour time point of cochlear SE regeneration.

### Identification of known pathways and processes among the differentially expressed genes

The comparative data listed in Supplementary Tables S1, S2, S3, S4, S5, S6, S7, S8, S9, S10, S11, S12, S13, S14, S15, S16, S17 and S18 were manually curated via interrogation of Gene Ontology databases as well as Medline literature citations. This served to identify multiple components or “signatures” of seven distinct signaling pathways within all four regenerative time courses. The identified pathways were those previously shown to be mediated by; *TGFβ, PAX, NOTCH, WNT, NFKappaB, Insulin/IGF1*, and *AP1* signaling. All of these have been implicated, in one way or another, in the normal development of the vertebrate inner ear. These signatures (along with their time course(s) and associated references to each component) are listed in Supplementary Tables S10, S11, S12, S13, S14, S15, S16. Again, as with the common genes described above, even within one identified pathway, the profiles of changes in each time course were frequently quite different. Nevertheless, some commonalities could be discerned; for example, the homeobox gene *TITF1/NKX2.1* (a component of both the *TGFβ* and *PAX* pathways) which interacts with both *SMAD3* and *PAX8* [31]–[33] showed a similar profile in both neomycin time courses.

**Figure 2:**
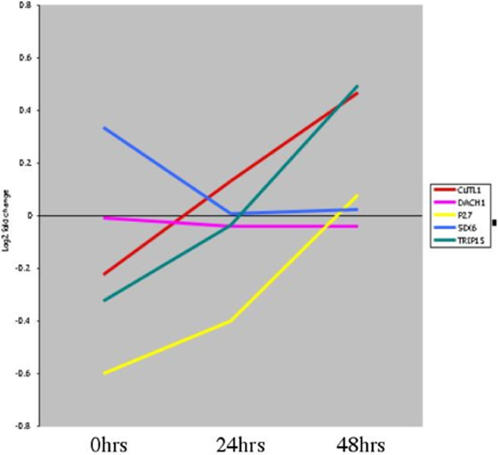
Gene expression changes in *p27^Kip^* and four genes that may regulate its expression. This diagram shows a combination of semi-quantitative PCR data (for *p27^Kip^)* and microarray data for the other four genes conducted on the utricle neomycin time courses. Each gene expression profile is color coded with the key to the right of each figure. The X-axis lists time points and the Y-axis is the log_2_ fold-change at each time point. Expression values are derived from Supplementary Table S2, except for *DACH1* which is detectably expressed, but is not significantly differentially expressed across the time course.

Not surprisingly, an additional grouping of genes fell within a set that we termed cell cycle/apoptosis genes (listed as Supplementary Table S17). Of interest among this set of genes were three that have been implicated in the regulation of *p27^KIP^*, a cyclin dependent kinase inhibitor that is a key regulator of cell proliferation during cochlear development [34]. Although *p27^KIP^* is expressed in supporting cells and may act as a block to cellular proliferation [35], a probe for this gene was not included on our array. Therefore, we conducted a semi-quantitative PCR analysis of the chicken *p27^KIP^* gene in the utricule neomycin specimens. This is shown in Figure 2 and indicates that *p27^KIP^* transcription was down-regulated after utricle SE damage and then returned to normal levels by 48 hrs after the removal of the antibiotic. Figure 2 also shows microarray data for four other genes that have been previously shown to regulate *p27^KIP^*. These are: *COPS2*, a component of the *COP9* signalosome [36], that can inhibit G1-S transition through interactions with *p27^KIP^*; *CUTL1* a transcription factor that inhibits *p27^KIP^* transcription [37]; *SIX6* within the *PAX* pathway which also represses *p27^KIP^* transcription [38]; and *DACH1* (a component of both the *PAX* and *TGF*–pathways) which interacts with *SIX6* to repress *p27^KIP^* transcription [38]. It is interesting to note that for the *COPS2* and *SIX6* genes the microarray data were consistent with their previously described interactions with *p27^KIP^* (i.e. *SIX6* transcript levels decreased over the time course and *COPS2* levels initially declined and then increased). *CUTL1* (a putative repressor of *p27^KIP^*) also appeared to increase in expression level over the time course and *DACH1* transcript levels did not significantly vary through the time course. This set of five genes is just one example of the many changes in known pathway components that can be constructed into mechanistic and testable hypotheses from this dataset.

### Clustering with self organizing maps

**Figure 3:**
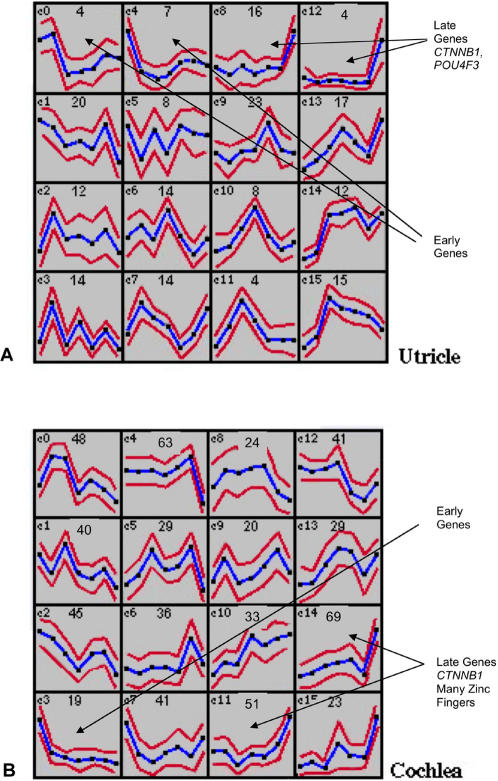
Analysis of the datasets by Self Organizing Maps. All of the differentially expressed genes listed in Supplementary Tables S2, S4, S6 and S8 were uploaded into Genecluster 2 and 16 centroids per organ were generated. Each box (centroid) in this figure is numbered from C0–C15 and they reflect common patterns of expression for clustered groups of genes within the dataset. The X-axis for each centroid consists of each time point and runs from the laser 30 min time point through 1 hr, 2 hrs, 3 hrs and then into the neomycin 0 time point followed by the neomycin 24 and 48 hr time points. The Y-axis indicates expression level (fold-change). The number in the top left of each centroid indicates the number of genes that fall into this cluster of co-expression. The top line indicates the upper boundary of expression for all of these genes and the lower line indicates the lower boundary. The middle line is the mean. All of the specific genes that fall into each centroid are listed in Supplementary Tables S18 and S19. Figure 3A shows the clustering for the utricle time points and Figure 3B shows the clustering for cochlea time points. Arrows indicate various patterns or genes within specific centroids that are described in the text.

As described above, literature/database searches plus manual curation of the data assisted us in placing a total of 70 known TFs into possible interactive pathways. However, the vast majority of the TFs in our set have no known function or correlations with known pathways. In order to potentially identify these relationships and to better discern possible patterns of co-expression within these data, we derived self-organizing maps (SOMs) by combining all differentially expressed genes across both time courses for each tissue type. This form of unsupervised clustering ([39], [40]) produces clusters of genes (with upper and lower limit bars) that show similar patterns of expression across a time course or set of treatments. In this case the situation is somewhat artificial, since in building these graphs we made the arbitrary choice that the 3 hr laser time point would precede the neomycin zero time point changes on the X-axis, whereas in reality the laser time course probably overlaps the early stages (0 hr to 24 hr) of neomycin recovery. Nevertheless, the purpose of these clusters was to visualize apparent patterns and potential clusters of genes within the data. Figure 3A shows a group of 16 SOM centroids (clusters of genes that show similar patterns of differential expression across all the time points) constructed using Genecluster 2 [39], [40] for the utricle time courses. Figure 3B shows sixteen centroids for the cochlea data. The actual names of every gene in each cluster are listed in Supplementary Tables S18 and S19. Some clusters exhibited relatively large temporal fluctuations in gene expression across both time courses. One example of this is centroid 3 in Figure 3A which includes a total of 14 genes such as *CEBPG, JUND, FOXP1*, and *HOXA13*. By contrast, centroids 8 and 12 in Figure 3A illustrate genes that show relatively small changes in expression, except at the 48 hour neomycin time point where they were all up regulated. These were the predominantly late genes in the utricle regenerative time course. These two centroids together comprised 19 genes and included *POU4F3* (previously implicated in hearing loss [41]), *CTNNB1* and *PPARGC1* (both in the *WNT* pathway). At the other end of the spectrum were the 11 genes in centroids 0 and 4 of Figure 3A that appeared to be activated early and peak in expression at the first or second laser time point. Among these are the nuclear hormone receptor *NR1I3,* which plays a role in transcriptional activation of genes involved in drug metabolism [42], [43] *SIX3*, a homeobox gene that regulates *PAX6* and *SOX2* in the developing eye [44] and *LOC51637*, a TF of unknown function, that we previously found to be up-regulated in the chicken utricle [15] relative to the cochlea.

The cochlea regenerative SOMs (16 in total) are shown in Figure 3B. In this case the predominantly late genes fall into centroids 11 and 14 and total 118 genes. However, additional examples of gradual up-regulation occur in centroids 10 and 15 (55 additional genes). Interestingly, of the 118 genes in centroids 11 and 14, a total of 45 are zinc finger transcription factors (as defined by being either ZF or ZNF family members listed in Supplementary Table S19). The vast majority of these are of unknown function and unknown target specificity. If the genes in centroids 10 and 15 are included, the total number of zinc finger TFs peaking in expression at the 48Hr time point rises to 61 (35% of the 173 total genes in these centroids). By contrast, the other twelve centroids in Figure 3B all together contain 19 zinc finger transcription factors (4% of a total of 432 genes in these centroids). Therefore, it appears that a dramatic burst of zinc finger gene expression occurs specifically at these late stages of regenerartive proliferation in the cochlear SE. This contrasts with the utricle SOMs where zinc finger TFs are distributed fairly evenly through the centroids. In common with the utricle time courses, *CTNNB1* peaks at 48 hrs in the cochlear time courses but, unlike in the utricle, *POU4F3* peaks earlier, at the 24Hr time point (in centroid 4 of Figure 3B). The predominately early genes (19 in total) in Figure 3B are contained within centroid 3. Of interest within this group are *EGR1*, which can be induced by *IGF* signaling [45], *NFIL3* which is a nuclear factor regulated by *IL3* [46], [47] and Neurogenin 1, which is involved in fate choice during inner ear development [47].

### Contrasting patterns of TF genes that are detectably expressed

**Figure 4:**
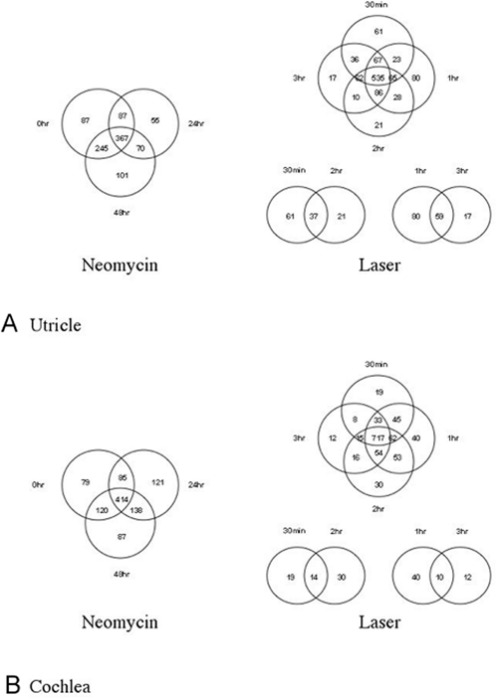
Detectably expressed TFs in the four treatment/time course combinations. All TFs that were present (as judged by exceeding a background intensity level) at any stage were considered in this analysis, irrespective of differential expression at any time point. Overlaps between these sets of TFs are illustrated in these Venn diagrams. Figure 4A shows overlaps for the utricle time points and treatments. Figure 4B shows overlaps for the cochlea time points and treatments. For a detailed listing of all of the genes in these diagrams see Supplementary Tables S20 and S21.

In addition to using the normalized intensity values to identify differentially expressed genes, we also used intensity values to determine which TFs were detectably expressed at any given time point, irrespective of any fold-change. This is a useful dataset since, at the level of detection of our microarrays, it defines lists of TFs that specify the normal functioning of the SE and makes no distinction between genes that never vary and those that change in their expression levels. This involved scoring all genes as “on” that reproducibly exceeded a background intensity level (and likewise any gene that failed to meet this cutoff was arbitrarily scored as “off”). This cut-off was based upon control oligonucleotides that were imbedded within our arrays and have no known homologous sequences in the chicken genome. Venn diagrams (Figures 4A and 4B) illustrate the results of this analysis. It is important to realize the differences between this analysis and the listings of differentially expressed genes in Supplemental tables S1 through [Supplementary-material pone.0000525.s003] [Supplementary-material pone.0000525.s004] [Supplementary-material pone.0000525.s005] [Supplementary-material pone.0000525.s006] [Supplementary-material pone.0000525.s007] [Supplementary-material pone.0000525.s008] S8. A gene such as *CEBPG* is among those that are differentially expressed in both the utricle laser and neomycin time courses (Supplementary Tables S1, S2, S3 and S4). However, in the Venn diagrams this gene is scored as being detectably expressed at all time points (albeit at different levels between them). In Figure 4A it therefore falls among the 367 genes that are commonly present in all time-ponts in the neomycin Venn diagram and the 535 common genes in the laser Venn diagram (Figure 4A). Within these two sets of common genes (that are apparently on in either the neomycin or laser time courses) there are 256 that are shared (Supplemental Tables S20 and S21). These comprise a core group of expressed TF genes for the sensory epithelium of the utricle, irrespective of time point or treatment. Likewise, the cochlea has a core group of 346 TF genes that are common to both time courses at all time points. There are also a group of 134 genes that are detectably expressed at all times in all four time courses. Additionally, the Venn diagrams identify many genes “uniquely” detectable at individual time points. In some cases these may overlap with those scored as being differentially expressed, or they may only just exceed the background threshold level at those particular time points. This analysis also indicates that the largest number of detectably expressed genes occurs at 0 and 48 hr in the Neomycin time course and at the 1 hr time point in the laser time course. A complete listing of the gene overlaps summarized in Figures 4A and 4B is provided in Supplemental Tables S20 and S21.

## Discussion

In this study we report the first large-scale analysis of changes in gene expression during the process of avian hair cell regeneration. We have detected the signatures of seven known signaling pathways in our data, and changes in expression levels of genes common to particular time courses and treatments. In general, we observed modest fold-changes in TF gene expression across all of the time courses and treatments. This is not unexpected for this class of genes, even when the array platform is within a species (rather than the type of cross-species platform used in this study). For example, in a recent study of gene expression changes of ∼25,000 genes in mouse organogenesis (from embryonic day 8.0 to postnatal day 1 [48]), a total of 160 TF genes changed by >1.2-fold in their expression level. In that particular study, the average TF change was 1.63-fold and the range was from a 3.66-fold down-regulation to a 3.63-fold up-regulation. This is remarkably similar to our observations in the current report and it presumably reflects the fact that small changes in these potent transcriptional regulators can have large downstream effects. One should also consider that some TFs are constitutively expressed and their activation is mediated by other events, such as phosphorylation (reviewed in [49]). In that regard, it is interesting to note that in our experiments we also detected changes in the expression levels of many of these genes whose protein products are regulated by phosphorylation events. For example, *JUND, CEBPG* and *CEBPB* are known to be activated by phosphorylation [49], [50] and yet we observed clear and reproducible changes in transcript levels for many of these types of TF genes.

Most of the changes we observed have not yet been linked into known networks or pathways. These genes should provide a rich future source of clues into the genetic programming of avian hair cell regeneration and SE development in general. Our descriptions of pathways and trends presented here is just the beginning of a systems biology approach to this important process. Within our data set there are numerous examples of individual, differentially expressed genes that have not previously been implicated in inner ear development or differentiation. For example, the *FOXP1* gene has previously been implicated in cardiac development [51] but from our data appears to be rapidly up-regulated early in the utricle laser time courses (see Supplementary Tables S3 and S4). We have recently confirmed that this gene is also differentially expressed during embryonic development of the mouse vestibular organs that give rise to these SE (Sajan, Warchol and Lovett in preparation).

There are also changes in particular classes of genes that may reflect underlying important pathways. One such example is the change in expression of the Polycomb complex genes *EZH1*, *EZH2* (enhancer of zeste 1 and 2) *CBX1, CBX3, CBX4, CBX6,* and *CBX8* (chromobox genes) that occur in the regenerative time courses in both the cochlea and utricle SE (Supplementary Table S1, S2, S3, S4, S5, S6, S7 and S8). These genes are especially noteworthy because of their roles in controlling cell fate decisions [52] and in preventing stem cell exhaustion [53] via epigenetic mechanisms [54]–[56].

**Figure 5:**
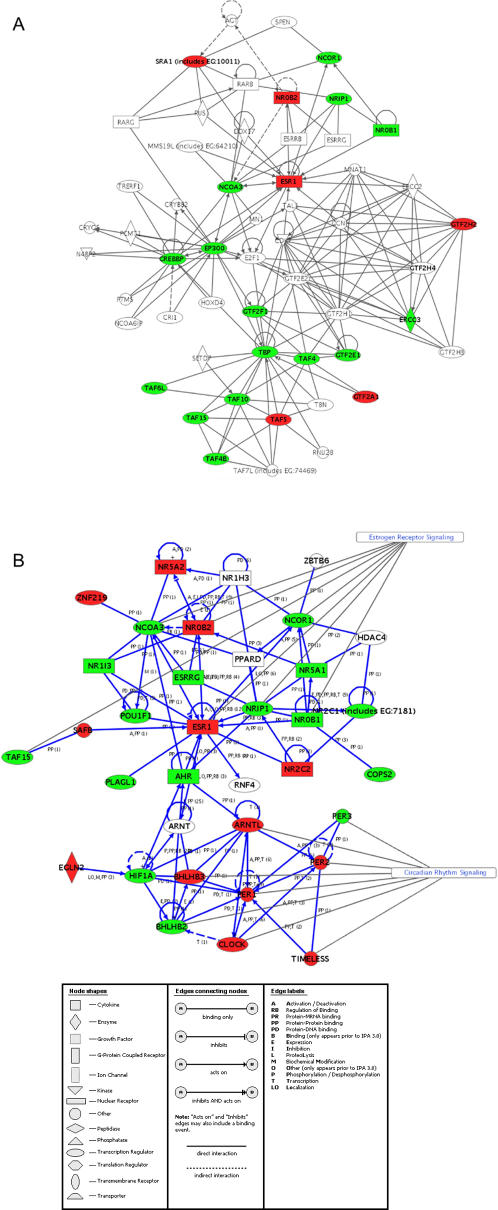
Two examples of Ingenuity gene networks constructed from cochlear differentially expressed genes. Genes that showed differential expression in both the laser and neomycin cochlear time courses (listed in Supplementary Tables S6 and S8) were uploaded to the web-based Ingenuity program (Ingenuity® Systems, www.ingenuity.com) and the network of interactions shown here was generated. Each interaction is shown according to the following legend and is supported by at least one literature citation (available from the Ingenuity website). Figure 5A shows the network of interactions for genes specifically identified within the cochlear neomycin time course as being part of Estrogen receptor signaling. Figure 5B shows the network of interactions surrounding Circadian rhythm signaling and was generated by uploading all of the cochlear differentially expressed genes (rather than a subset as in 5A). Red denotes up-regulation and green down-regulation in at least one time point. Genes shown in bold with no shading vary across a time course (e.g. *GTF2H4* in Figure 5A was up-regulated at 24 hrs and down-regulated at 48 hrs). All other genes were either not represented on the microarray or were not significantly differentially expressed. A key to additional Ingenuity labels is listed below.

A more global method for interrogating the data presented in this report is to make use of web-delivered tools to discover possible networks or canonical pathways. Ingenuity Pathways Analysis (IPA; Ingenuity® Systems, www.ingenuity.com) is one such set of tools. We uploaded the specific sets of shared genes (fold-changes and p-values) listed in Supplementary Tables S1, S2, S3, S4, S5, S6, S7 and S8 into the IPA application. These genes were then used to generate biological networks developed form information contained in the Ingenuity Pathways Knowledge Base (IPKB). All connections within the IPKB are supported by at least one reference from the literature (see www.ingenuity.com). IPA also computed a p-value for each generated network derived from a right-tailed Fisher's exact test, which indicates the probability that the focus genes in a network are found together because of chance alone. A complete description of all of these networks is beyond the scope of the current study. However, it is interesting to note that one of the highest scoring networks (p-value of 2.4×10^−6^) shared between both the cochlea and utricle regenerating SE, involves multiple components of estrogen receptor (ER) signaling. Figure 5A shows an IPA network of these various ER components (analyzed separately from other differentially expressed genes). Previous studies have commented on the presence of estrogen receptor in the developing mammalian inner ear [57], but it is not clear what endogenous ligand(s) might activate this pathway or whether this acts via the ligand-independent route [58]. It is known that ligand–independent activation of ER can be achieved by ER phosphorylation mediated by various signaling pathways[59].

An additional example of IPA network building involves the significant enrichment (p-value of 5×10^−7^) in the cochlear regenerating epithelia for genes involved in regulation of circadian rhythm (e.g. *BHLHB3, PER1, CREB1, PER2, TIMELESS, CLOCK*). This network has not previously been implicated in sensory epithelia regeneration or differentiation. These genes are classically thought of as regulating 24 hour periodicities in gene expression (reviewed in [60]) although they may play roles in other processes, such as genotoxic stress [61]. Figure 5B shows an IPA network for the cochlear differentially expressed genes (in this case, from an analysis of all differentially expressed cochlear genes). This indicates that both circadian rhythm and estrogen receptor signaling may converge within this network. It is also interesting to note that the circadian rhythm pathway may interact with the aforementioned Polycomb complex *via EZH2* activity, since the mammalian *EZH2* gene product binds to the *PER1* and *PER2* promoters [62] .

These examples serve to illustrate some of the routes that these data now open up for the further understanding of this complex network of interactions. Together these changes in gene expression lead to the proliferation of supporting cells and the eventual repopulation of the inner ear hair cells. However, unraveling which of these candidate genes are necessary and sufficient for the process will require more direct tests of their effects; such as RNAi treatments, gene knock-out and gene knock-in technologies in both the avian and the mammalian inner ear.

## Material and Methods

### Culture of sensory organs and isolated sensory epithelia

White Leghorn chicks (10–21 days post hatch) were euthanized by CO_2_ asphyxiation and decapitated. Cochlea and utricles were isolated as described previously [15]. Detailed methods for the preparation of organ cultures and cultures of isolated SE have also been reported previously (cochlea: [63]; utricle: [18]; isolated epithelia: [17]).

### Ototoxic hair cell injury

Cochleae and utricles were placed in small culture wells (Mat-Tek; 1 cm diameter; 1 specimen/well) that contained 100 µm of Medium-199 (with Earles salts, 25 mM HEPES; 2,200 mg/L sodium bicarbonate, and 0.69 mM l-glutamine; Invitrogen), supplemented with 10% fetal bovine serum (FBS; Invitrogen). Half of the specimens also received 1 mM neomycin sulfate (Sigma), in order to lesion sensory hair cells [2], [18]. Other specimens were cultured under identical conditions, expect that they did not receive neomycin; these served as time-matched controls. Utricles and cochleae were maintained under these conditions for 24 hours. At this point, some specimens were harvested as described below; these formed the 0 hr recovery group. Other specimens were rinsed 3× with fresh Medium-199/10%FBS and maintained in culture for an additional 24 or 48 hr. After appropriate survival times, specimens were rinsed with fresh Medium-199 and incubated for 60 min in 500 µg/ml thermolysin (Sigma; prepared in Medium-199), at 37°C. The sensory organs were then transferred to chilled Medium-199 and the sensory epithelia (consisting of sheets of hair cells and supporting cells) were separated from the associated stromal tissue using a 27-gauge needle. Isolated epithelia were dissolved in 100 µl Trizol and stored at −80°C until further processing. Each biological sample consisted of four neomycin-treated and four time-matched control epithelia at each survival time.

### Laser microbeam ablations

SE from the cochlea or utricle were isolated using thermolysin [17], cut into small pieces, and grown for 7–10 days on fibronectin-coated wells (Mat-Tek) that contained 50 µl Medium-199/10%FBS. Initial immunocytochemical studies confirmed the purity of the epithelial cultures. Cell-cell junctions in the sensory epithelia of the avian cochlea and utricle are mediated by the adhesion molecule N-cadherin, while junctions between cells from the surrounding nonsensory tissue are mediated by E-cadherin [17], [64], [65]. Incubation of epithelial cultures with an antibody directed against chick N-cadherin (NCD-2; Zymed) resulted in strong immunoreactivity at nearly all cell-cell junctions (Supplementary Figure S1). This result (which is consistent with previous findings [17]) indicates that the cultures are almost entirely comprised of cells from the sensory epithelia and contain very few extrasensory cells.

Once the cultures were semi-confluent, they were lesioned via laser microsurgery [63]. Individual cultures were placed on the stage of an inverted microscope (Zeiss Axiovert 135) that was equipped with a pulsed laser microbeam device (Photonics Instruments). The beam was focused through a 100× objective lens at a place that was coincident with the cultured cells and the pulse controller was adjusted to run at ∼10 pulses/sec. The specimen was then moved slowly through the beam path, using the microscope stage manipulators, resulting in the creation of a linear ‘wound’ in the confluent epithelium. Typically, 2–3 wounds were made in each culture, separated by 100–200 µm. Unlesioned (control) cultures were also removed from the incubator and were placed on the side of the microscope stage during the lesioning of their time-matched counterparts. The lesioned and unlesioned cultures were then returned to the incubator and maintained for an additional 30 min, 1 hr, 2 hr, or 3 hr. Four individual lesioned and unlesioned specimens constituted one biological sample at each of the four recovery times. RNA from the cultures was obtained by addition of 100 µl of Trizol (Invitrogen) to each well. Samples were then stored at −80°C until further processing.

### cDNA amplification

Isolation of RNA, cDNA synthesis and amplification were carried out as previously described [15] with the following modifications: following the second round of amplification and Sephadex G50 minicolumn purification, cDNA was added to an *in vitro* transcription kit (Megascript T7 high yield kit, Ambion) as per the manufacturers instructions. Run-off RNAs were LiCl precipitated, washed with 75% ethanol, dried and resuspended in water at a concentration of 0.5–1 µg/µl. The overall yield and quality of run-off products were assessed by gel electrophoresis.

### Target labeling and microarray hybridizations

Run-off RNAs from each sample were used as templates in an oligo dT_12–15_ primed cDNA synthesis reaction that included amino-allyl dUTP (Sigma, 0.2 mM). The cDNA was then either coupled to Cy3 or Cy5 mono ester dyes (Amersham Pharmacia). Labeled cDNA populations were precipitated and resuspended in 20 µl hybridization buffer (50% formamide, 6× SSPE, 5× Denhart's, 0.5% SDS. 10% dextran sulfate). Microarray slides were hybridized at 37°C for 12 h and were washed as described in [15].

### Microarray Design

The microarrray employed in this study consisted of 50 mer oligonucleotides designed to the vast majority of human transcription factor genes (Messina et al 2004). These probes were designed from coding regions and have previously been shown to accurately report on the majority of chicken TFs when used under appropriate hybridization conditions [15]. The array also contained probes for a limited number of non-TFs (for example, sonic hedgehog [SHH]). Oligonucleotides were spotted in duplicate on glass slides. The array also contained a number of control 50 mers that have no homolog in the human (or chicken) genome. These were used to measure array sensitivity and to assess background correction levels.

At least two biological samples (and frequently three or more) were analyzed per time point, treatment and tissue. For every time point to control comparison we conducted at least four separate microarray hybridizations (two comparisons and two dye switches). We also compared each experimental time point to the next experimental time point and control time points to adjacent control time point hybridizations. A total of 226 array comparisons were conducted for this study. These data are available through http://www.ncbi.nlm.nih.gov/geo/with accession numbers GPL4384, GSE5991, GSE6050, GSE6051 and GSE6052. These data comply with the “minimum information about a microarray experiment” (MIAME) requirements.

### Data Analysis

Microarray images were analyzed with the BioDiscovery Imagene software package. The Cy3 and Cy5 images were computationally overlaid, aligned and gridded. The intensity of each spot was then measured by laser scanning. The raw intensity files generated by Imagene were analyzed by MIDAS2.17 and TMEV2.2 programs of TIGR software (http://www.tigr.org) and GeneCluster 2.0 (http://www.broad.mit.edu/cancer/software/genecluster2/gc2.html). Together, these provide functions for normalizing and analyzing the data. Data were normalized by implementing locally weighted linear regression (LOWESS). After data adjustment, Hierarchical Clustering (HCL) of the data was performed using TIGR TMEV. HCL was implemented to filter out poor hybridizations. Low intensity filtering was performed to exclude genes with intensities lower than the specified thresholds. An arbitrary cut off (usually in the range of 800–1200) was chosen based on the control spot intensities (see Microarray design above). This value was also based on the distribution of intensity values of the array. Approximately 10–20% of the genes were removed in this way. We next selected genes that followed the same trend in at least 80% of the replicated hybridizations. The average fold change was then calculated by averaging the values across the replicate hybridizations. Since the genes identified by these methods (as being differentially expressed) did not necessarily have expression ratios at all the time points (some were filtered out at one stage, but were retained at another) we manually extracted the adjusted intensity values at these time points and calculated the ratios from the corresponding hybridization data. Such “filled-in” expression ratios allowed us to analyze the expression of all the selected genes across the entire time course for a particular treatment. P-values were calculated by conducting a one sample t-test on the adjusted intensity data of the entire array of selected hybridizations at each time point.

### Quantitative PCR (qPCR)

Validation of microarray fold changes was assessed by qPCR as previously described [15] using a Stratagene MX3000P machine. cGAPDH was used as a control gene to calculate ΔCt in order to normalize data. Normalized values were used to calculate ΔCt to determine fold changes.

## Supporting Information

Figure S1 Immunoreactivity for N-cadherin in cultures of isolated epithelial cells from the chick cochlea (A) and utricle (B). Epithelial cultures were maintained in vitro for seven days, at which time the specimens were fixed and processed for immunocytochemical localization of N-cadherin. Labeling for N-cadherin (green) was observed at nearly all cell-cell junctions, confirming that the cultured cells originated from the sensory epithelia of the cochlea or utricle (see text). Cell nuclei were also labeled with DAPI (blue). Scale bar = 20 µm.
(2.52 MB DOC)

Table S1 Utricle Neomycin Differentially Expressed Genes (194 total). This listing shows all genes that exhibited>1.2-fold changes in expression, irrespective of p-value. For p-value filtered data see Table S2.
(0.21 MB DOC)

Table S2 Utricle Neomycin Differential Expression>1.2-fold and P< = 0.05
(0.06 MB PDF)

Table S3 Utricle Laser Differentially Expressed Genes (261 total). This listing shows all genes that exhibited>1.2-fold changes in expression, irrespective of P-value. For p-value filtered data see Table S4.
(0.36 MB DOC)

Table S4 Utricle Laser Differential Expression>1.2-fold and P< = 0.05
(0.05 MB PDF)

Table S5 Cochlea Neomycin Differentially Expressed Genes (533 total). This listing shows all genes that exhibited>1.2-fold changes in expression, irrespective of P-value. For p-value filtered data see Table S6
(0.71 MB DOC)

Table S6 Cochlea Neomycin Differential Expression>1.2-fold and P< = 0.05
(0.10 MB PDF)

Table S7 Cochlea Laser Differentially Expressed Genes (386 genes total). This listing shows all genes that exhibited>1.2-fold changes in expression, irrespective of P-value. For p-value filtered data see Table S8.
(0.53 MB DOC)

Table S8 Cochlea Laser Differential Expression>1.2-fold and P< = 0.05
(0.07 MB PDF)

Table S9 A listing of genes that exhibit similar patterns of expression across the four timecourses. utr-neo = utricle neomycin timecourse , coch-lsr = cochlea laser timecourse and so on. 1 = up-regulation, 0 = no change and −1 = down-regulation relative to untreated controls.
(0.04 MB PDF)

Table S10 Transforming Growth Factor-Beta Signaling. CN = Cochlea Neomycin timecourse. CL = Cochlea Laser timecourse. UN = Utricle Neomycin timecourse. UL = Utricle Laser timecourse.
(0.03 MB DOC)

Table S11 The PAX-EYA-SIX pathway. CN = Cochlea Neomycin timecourse. CL = Cochlea Laser timecourse. UN = Utricle Neomycin timecourse. UL = Utricle Laser timecourse.
(0.03 MB DOC)

Table S12 Notch signaling. CN = Cochlea Neomycin timecourse. CL = Cochlea Laser timecourse. UN = Utricle Neomycin timecourse. UL = Utricle Laser timecourse.
(0.02 MB DOC)

Table S13 WNT signaling. CN = Cochlea Neomycin timecourse. CL = Cochlea Laser timecourse. UN = Utricle Neomycin timecourse. UL = Utricle Laser timecourse.
(0.03 MB DOC)

Table S14 NFKappaB. CN = Cochlea Neomycin timecourse. CL = Cochlea Laser timecourse. UN = Utricle Neomycin timecourse. UL = Utricle Laser timecourse.
(0.02 MB DOC)

Table S15 Insulin/IGF signaling. CN = Cochlea Neomycin timecourse. CL = Cochlea Laser timecourse. UN = Utricle Neomycin timecourse. UL = Utricle Laser timecourse.
(0.02 MB DOC)

Table S16 AP-1 Signaling. CN = Cochlea Neomycin timecourse. CL = Cochlea Laser timecourse. UN = Utricle Neomycin timecourse. UL = Utricle Laser timecourse.
(0.03 MB DOC)

Table S17 Cell Cycle/Apoptosis. CN = Cochlea Neomycin timecourse. CL = Cochlea Laser timecourse. UN = Utricle Neomycin timecourse. UL = Utricle Laser timecourse.
(0.03 MB DOC)

Table S18 Utricle Self Organizing Map Centroid Groups shown in Figure 3A.
(0.03 MB DOC)

Table S19 Cochlea Self Organizing Map Centroid Groups shown in Figure 3B.
(0.04 MB DOC)

Table S20 Utricle Detectably Expressed Genes
(0.23 MB DOC)

Table S21 Cochlea Detectably Expressed Genes
(0.23 MB DOC)
