# Supplementary figures and images for: Large Scale Gene Expression Profiles of Regenerating Inner Ear Sensory Epithelia
Source: PLoS One. 2007 Jun 13;2(6):e525. doi: 10.1371/journal.pone.0000525 (PMC1888727; doi:10.1371/journal.pone.0000525)

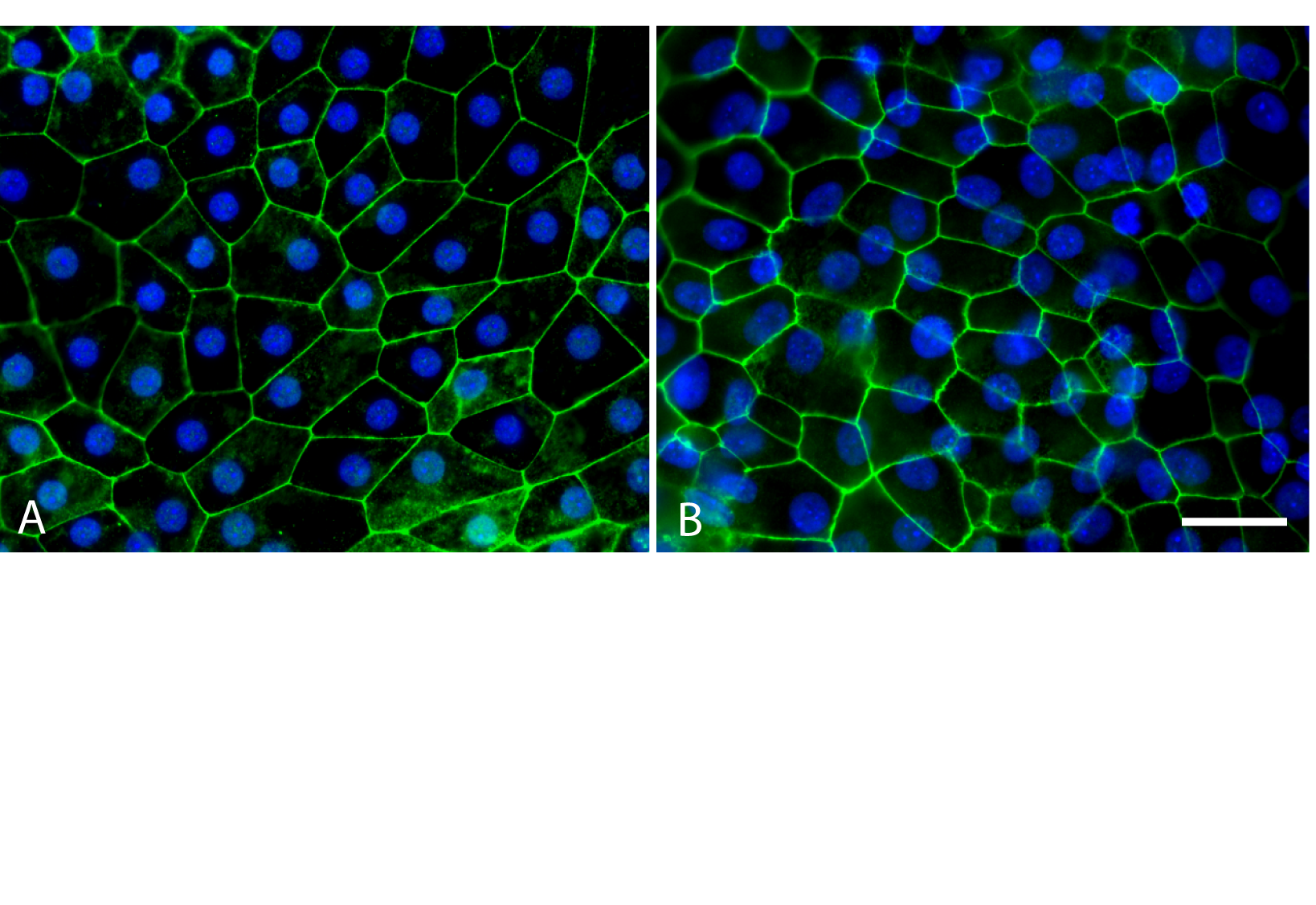

Supplement: Figure S1 — Immunoreactivity for N-cadherin in cultures of isolated epithelial cells from the chick cochlea (A) and utricle (B). Epithelial cultures were maintained in vitro for seven days, at which time the specimens were fixed and processed for immunocytochemical localization of N-cadherin. Labeling for N-cadherin (green) was observed at nearly all cell-cell junctions, confirming that the cultured cells originated from the sensory epithelia of the cochlea or utricle (see text). Cell nuclei were also labeled with DAPI (blue). Scale bar = 20 µm. (2.52 MB DOC) [file pone.0000525.s001.doc]
