# Supplementary material for: Large Scale Gene Expression Profiles of Regenerating Inner Ear Sensory Epithelia
Source: PLoS One. 2007 Jun 13;2(6):e525. doi: 10.1371/journal.pone.0000525 (PMC1888727; doi:10.1371/journal.pone.0000525)
Supplement: Table S1 — Utricle Neomycin Differentially Expressed Genes (194 total). This listing shows all genes that exhibited>1.2-fold changes in expression, irrespective of p-value. For p-value filtered data see Table S2. (0.21 MB DOC) [file pone.0000525.s002.doc]

Supplemental Table S1.

|  | **O hr** | | **24 hr** | | **48 hr** | |  |
| --- | --- | --- | --- | --- | --- | --- | --- |
| **Gene ID** | **Fold change** | **P-value** | **Fold Change** | **P-value** | **Fold Change** | **P-value** | **Notes/Description** |
| AF5Q31 | 1.113 | 0.475 | 0.662 | 0.211 | 1.071 | 0.702 | ALL1 fused gene from 5q31 |
| ARIX | 0.745 | 0.14 | 1.039 | 0.568 | 0.939 | 0.513 | aristaless homeobox |
| ASH2L | 0.99 | 0.89 | 0.781 | 0.006 | 1.028 | 0.548 | ash2 (absent, small, or homeotic, Drosophila, homolog)-like |
| ATF2 | 0.804 | 0.021 | 1.013 | 0.886 | 0.754 | 0.39 | activating transcription factor 2 |
| BCL11A | 1.327 | 0.127 | 1.051 | 0.577 | 1.334 | 0.026 | B-cell CLL/lymphoma 11A (zinc finger protein) |
| BCL11B | 0.842 | 0.146 | 0.919 | 0.262 | 0.785 | 0.001 | B-cell CLL/lymphoma 11B (zinc finger protein) |
| BRD1 | 0.635 | 0.006 | 0.94 | 0.646 | 0.603 | 0.002 | bromodomain-containing 1 |
| C21orf18 | 0.538 | 0.029 | 0.954 | 0.779 | 0.603 | 0.003 | chromosome 21 open reading frame 18 |
| CBX3 | 1.21 | 0.046 | 1.017 | 0.772 | 0.951 | 0.532 | chromobox homolog 3 (Drosophila HP1 gamma) |
| CBX4 | 0.588 | 0.037 | 1.192 | 0.074 | 0.823 | 0.511 | chromobox homolog 4 (Drosophila Pc class) |
| CDK7 | 1.022 | 0.816 | 0.871 | 0.216 | 0.829 | 0.036 | cyclin-dependent kinase 7 (homolog of Xenopus MO15 cdk-activating kinase) |
| CEBPB | 1.062 | 0.443 | 0.788 | 0.453 | 0.878 | 0.9 | CCAAT/enhancer binding protein (C/EBP), beta |
| CEBPG | 0.646 | 0.04 | 1.236 | 0.43 | 0.506 | 0.008 | CCAAT/enhancer binding protein (C/EBP), gamma |
| CHD3 | 1.057 | 0.451 | 1.059 | 0.26 | 1.39 | 0.123 | chromodomain helicase DNA binding protein 3 |
| CITED1 | 1.521 | 0.036 | 0.689 | 0.182 | 0.985 | 0.885 | Cbp/p300-interacting transactivator, with Glu/Asp-rich carboxy-terminal domain, 1 |
| CREBL1 | 0.994 | 0.902 | 1.072 | 0.088 | 1.322 | 0.032 | cAMP responsive element binding protein-like 1 |
| CREG | 0.95 | 0.424 | 1.124 | 0.159 | 0.807 | 0.045 | cellular repressor of E1A-stimulated genes |
| CRIP2 | 1.005 | 0.873 | 1.047 | 0.494 | 0.63 | 0.134 | cysteine-rich protein 2 |
| CROC4 | 0.907 | 0.062 | 0.689 | 0.05 | 0.972 | 0.857 | transcriptional activator of the c-fos promoter |
| CRSP6 | 0.694 | 0.013 | 1.062 | 0.705 | 0.989 | 0.949 | cofactor required for Sp1 transcriptional activation, subunit 6 (77kD) |
| CRX | 1.17 | 0.057 | 1.200 | 0.038 | 1.048 | 0.268 | cone-rod homeobox |
| CSDA | 1.056 | 0.484 | 0.973 | 0.747 | 1.321 | 0.008 | cold shock domain protein A |
| CSRP2 | 1.006 | 0.939 | 0.914 | 0.393 | 0.778 | 0.049 | cysteine and glycine-rich protein 2 |
| CTNNB1 | 0.789 | 0.092 | 0.89 | 0.101 | 3.097 | 0.035 | catenin (cadherin-associated protein), beta 1 (88kD) |
| CUTL1 | 0.857 | 0.012 | 1.097 | 0.14 | 1.381 | 0.006 | cut (Drosophila)-like 1 (CCAAT displacement protein) |
| DEAF1 | 1.321 | 0.002 | 1.275 | 0.24 | 1.003 | 0.97 | deformed epidermal autoregulatory factor 1 (Drosophila) |
| DKFZP434B0335 | 0.69 | 0.112 | 0.905 | 0.256 | 0.807 | 0.001 | DKFZP434B0335 protein |
| DLX4 | 1.027 | 0.7 | 1.132 | 0.373 | 0.82 | 0.138 | distal-less homeobox 4 |
| DLX6 | 0.784 | 0.016 | 0.85 | 0.087 | 0.991 | 0.953 | distal-less homeobox 6 |
| DUX2 | 1.254 | 0.01 | 1.198 | 0.086 | 1.136 | 0.041 | double homeobox 2 |
| E2F2 | 0.927 | 0.627 | 1.203 | 0.006 | 0.558 | 0.087 | E2F transcription factor 2 |
| E2F5 | 0.778 | 0.015 | 0.949 | 0.446 | 1.07 | 0.45 | E2F transcription factor 5, p130-binding |
| EBF | 0.789 | 0.009 | 1.08 | 0.413 | 1.16 | 0.184 | early B-cell factor |
| EOMES | 0.979 | 0.81 | 0.992 | 0.877 | 1.209 | 1.09E-04 | eomesodermin (Xenopus laevis) homolog |
| ERCC6 | 0.928 | 0.253 | 0.964 | 0.54 | 1.203 | 0.007 | excision repair cross-complementing rodent repair deficiency, complementation group 6 |
| ESR1 | 0.962 | 0.681 | 0.787 | 0.597 | 1.647 | 0.163 | estrogen receptor 1 |
| ETV5 | 0.952 | 0.441 | 0.829 | 0.028 | 1.017 | 0.876 | ets variant gene 5 (ets-related molecule) |
| EYA3 | 1.031 | 0.668 | 0.728 | 0.075 | 0.936 | 0.375 | eyes absent (Drosophila) homolog 3 |
| EZH1 | 0.708 | 0.127 | 1.011 | 0.898 | 0.749 | 0.102 | enhancer of zeste homolog 1 |
| EZH2 | 1.076 | 0.383 | 0.883 | 0.917 | 1.234 | 0.005 | enhancer of zeste homolog 2 |
| FHL1 | 0.709 | 0.062 | 1.042 | 0.512 | 0.743 | 4.63E-05 | four and a half LIM domains 1 |
| FHL2 | 0.889 | 0.029 | 1.037 | 0.606 | 0.802 | 0.255 | four and a half LIM domains 2 |
| FLJ10251 | 1.225 | 0.002 | 1.229 | 0.135 | 1.057 | 0.29 | hypothetical protein FLJ10251 |
| FLJ10891 | 1.25 | 0.005 | 1.174 | 0.22 | 1.025 | 0.356 | hypothetical protein FLJ10891 |
| FLJ12827 | 1.219 | 0.018 | 1.01 | 0.811 | 1.143 | 0.33 | zinc finger protein ZNF408 |
| FLJ13590 | 1.248 | 0.006 | 1.023 | 0.458 | 1.207 | 0.029 | zinc finger protein ZNF408 |
| FLJ20321 | 0.999 | 0.97583 | 1.034 | 0.386 | 1.288 | 0.083 | castor homolog 1, zinc finger (Drosophila) (CASZ1) |
| FLJ20595 | 1.346 | 0.01 | 0.917 | 0.252 | 0.91 | 0.055 | likely ortholog of mouse zinc finger protein ZFP29 |
| FLJ22252 | 1.05 | 0.154 | 1.031 | 0.732 | 0.824 | 0.168 | SRY (sex determining region Y)-box 17 (SOX17) |
| FOG2 | 0.974 | 0.736 | 0.808 | 0.113 | 0.76 | 0.001 | friend of GATA2 |
| FOXF2 | 1.021 | 0.731 | 1.201 | 0.055 | 1.07 | 0.4 | forkhead box F2 |
| GCN5L1 | 0.93 | 0.301 | 1.164 | 0.172 | 1.355 | 0.161 | GCN5 (general control of amino-acid synthesis, yeast, homolog)-like 1 |
| GIOT-2 | 0.977 | 0.813 | 0.689 | 0.047 | 0.702 | 0.036 | GIOT-2 for gonadotropin inducible transcription repressor-2 |
| GLI2 | 0.857 | 0.067 | 1.065 | 0.387 | 1.343 | 0.194 | GLI-Kruppel family member GLI2 |
| GTF2A1 | 1.431 | 0.013 | 1.171 | 0.101 | 1.321 | 0.041 | general transcription factor IIA, 1 (37kD and 19kD subunits) |
| GTF2E1 | 1.063 | 0.399 | 0.92 | 0.044 | 1.22 | 0.007 | general transcription factor IIE, polypeptide 1 (alpha subunit, 56kD) |
| GTF2F1 | 0.785 | 0.001 | 0.939 | 0.237 | 1.18 | 0.068 | general transcription factor IIF, polypeptide 1 (74kD subunit) |
| GTF2H1 | 0.936 | 0.161 | 1.023 | 0.763 | 1.283 | 0.013 | general transcription factor IIH, polypeptide 1 (62kD subunit) |
| GTF2H3 | 1.049 | 0.505 | 1.017 | 0.697 | 1.2 | 0.006 | general transcription factor IIH, polypeptide 3 (34kD subunit) |
| GTF3C4 | 1.286 | 0.022 | 0.876 | 0.829 | 1.106 | 0.165 | general transcription factor IIIC, polypeptide 4 (90kD) |
| H-L(3)MBT | 1.474 | 0.002 | 1.041 | 0.739 | 0.968 | 0.717 | lethal (3) malignant brain tumor l(3)mbt protein (Drosophila) homolog |
| HEY1 | 0.786 | 0.033 | 0.922 | 0.517 | 1.02 | 0.837 | hairy/enhancer-of-split related with YRPW motif 1 |
| HHEX | 1.123 | 0.031 | 1.023 | 0.665 | 1.452 | 0.046 | hematopoietically expressed homeobox |
| HIF1A | 0.816 | 0.081 | 0.703 | 0.048 | 1.071 | 0.616 | hypoxia-inducible factor 1, alpha subunit (basic helix-loop-helix transcription factor) |
| HIRA | 1.095 | 0.328 | 0.948 | 0.194 | 1.22 | 0.029 | HIR (histone cell cycle regulation defective, S. cerevisiae) homolog A |
| HIVEP1 | 1.013 | 0.866 | 1.038 | 0.672 | 1.243 | 0.041 | human immunodeficiency virus type I enhancer-binding protein 1 |
| HMGIY | 0.899 | 0.187 | 0.75 | 0.255 | 0.946 | 0.354 | high-mobility group (nonhistone chromosomal) protein isoforms I and Y |
| HNF3A | 1.01 | 0.787 | 1.262 | 8.52E-05 | 0.801 | 0.039 | hepatocyte nuclear factor 3, alpha |
| HNF3B | 0.859 | 0.162 | 0.912 | 0.217 | 1.948 | 0.055 | hepatocyte nuclear factor 3, beta |
| HOXA13 | 0.67 | 0.008 | 0.947 | 0.502 | 0.934 | 0.437 | homeo box A13 |
| HOXB7 | 1.135 | 0.07 | 0.976 | 0.724 | 1.277 | 0.018 | homeo box B7 |
| HOXB9 | 1.285 | 0.356 | 0.995 | 0.974 | 0.797 | 0.299 | homeobox B9 |
| HOXD12 | 1.066 | 0.385 | 1.127 | 0.011 | 1.367 | 0.047 | homeo box D12 |
| HOXD8 | 1.39 | 0.035 | 1.226 | 0.147 | 1.339 | 0.312 | homeobox D8 |
| HRIHFB2436 | 0.971 | 0.679 | 1.056 | 0.594 | 0.778 | 0.101 | endocrine regulator |
| HSAJ2425 | 1.288 | 0.043 | 0.978 | 0.788 | 1.282 | 0.127 | p65 protein |
| HSF1 | 0.937 | 0.614 | 0.904 | 0.61 | 0.58 | 0.015 | heat shock transcription factor 1 |
| HSF2BP | 0.892 | 0.272 | 0.749 | 0.075 | 0.729 | 0.044 | heat shock transcription factor 2 binding protein |
| HSPC018 | 1.11 | 0.134 | 0.969 | 0.404 | 1.2 | 0.014 | GTP binding protein 1 (GTPBP1) |
| HSPX153 | 1.174 | 0.012 | 1.04 | 0.417 | 1.385 | 0.063 | HPX-153 homeobox |
| ILF1 | 0.645 | 0.018 | 0.944 | 0.475 | 1.06 | 0.089 | interleukin enhancer binding factor 1 |
| ILF2 | 1.013 | 0.79 | 0.726 | 0.063 | 0.921 | 0.304 | interleukin enhancer binding factor 2 |
| DNAJ | 0.724 | 0.014 | 0.981 | 0.714 | 0.711 | 0.061 | immune dysregulation, polyendocrinopathy, enteropathy, X-linked |
| IRF2 | 0.681 | 0.029 | 1.188 | 0.117 | 0.829 | 0.058 | interferon regulatory factor 2 |
| IRF3 | 0.981 | 0.735 | 1.015 | 0.731 | 1.316 | 0.123 | interferon regulatory factor 3 |
| ISGF3G | 0.799 | 0.008 | 1.051 | 0.382 | 1.054 | 0.316 | interferon-stimulated transcription factor 3, gamma (48kD) |
| JUN | 0.829 | 0.336 | 0.993 | 0.971 | 0.957 | 0.698 | v-jun avian sarcoma virus 17 oncogene homolog |
| JUND | 0.849 | 0.21 | 1.36 | 0.661 | 0.751 | 0.074 | Jun D proto-oncogene |
| KIAA0014 | 1.037 | 0.616 | 1.297 | 0.454 | 1.89 | 0.061 | leucine rich repeat containing 14 (LRRC14) |
| KIAA0130 | 1.35 | 0.003 | 1.109 | 0.392 | 0.943 | 0.687 | thyroid hormone receptor-associated protein, 100 kDa (TRAP100) |
| KIAA0173 | 1.365 | 0.009 | 0.932 | 0.707 | 1.289 | 0.11 | tubulin tyrosine ligase-like family, member 4 (TTLL4) |
| KIAA0395 | 0.983 | 0.785 | 0.871 | 0.049 | 1.29 | 0.044 | triple homeobox 1 (TIX1) |
| KIAA1041 | 0.665 | 0.016 | 0.837 | 0.148 | 0.934 | 0.624 | forkhead box J3 (FOXJ3) |
| KIAA1528 | 1.187 | 0.033 | 1.028 | 0.408 | 1.353 | 0.002 | deltex homolog 2 (Drosophila) (DTX2) |
| LHX4 | 1.257 | 0.025 | 1.024 | 0.665 | 0.964 | 0.091 | LIM homeobox protein 4 |
| LOC51058 | 1.268 | 0.089 | 1.031 | 0.853 | 1.497 | 0.052 | hypothetical protein |
| LOC51131 | 1.201 | 0.036 | 1.009 | 0.855 | 1.21 | 0.097 | putative zinc finger protein NY-REN-34 antigen |
| LOC57209 | 1.631 | 0.019 | 1.157 | 0.28 | 1.29 | 0.014 | Kruppel-type zinc finger protein |
| LOC58500 | 0.887 | 0.245 | 0.969 | 0.722 | 1.301 | 0.275 | zinc finger protein (clone 647) |
| LZLP | 1.157 | 0.023 | 0.954 | 0.421 | 1.324 | 0.012 | leucine zipper-like protein |
| MADH2 | 1.03 | 0.678 | 1.102 | 0.191 | 1.32 | 0.034 | MAD (mothers against decapentaplegic, Drosophila) homolog 2 |
| MADH7 | 0.909 | 0.34 | 1.228 | 0.765 | 0.823 | 0.14 | MAD (mothers against decapentaplegic, Drosophila) homolog 7 |
| MAPK8IP1 | 1.228 | 0.054 | 0.642 | 0.008 | 0.889 | 0.231 | mitogen-activated protein kinase 8 interacting protein 1 |
| MEIS2 | 1.16 | 0.059 | 1.202 | 0.187 | 1.114 | 0.026 | homeobox protein MEIS2 |
| MGC2508 | 0.985 | 0.836 | 1.055 | 0.516 | 1.216 | 0.273 | hypothetical protein MGC2508 |
| MID1 | 0.812 | 0.161 | 1.027 | 0.677 | 0.976 | 0.79 | midline 1 (Opitz/BBB syndrome), zinc finger X and Y |
| MLLT2 | 1.027 | 0.491 | 1.018 | 0.805 | 1.313 | 0.096 | myeloid/lymphoid or mixed-lineage leukemia (trithorax (Drosophila) homolog); translocated to 2 |
| MLLT6 | 1.144 | 0.118 | 0.749 | 0.046 | 0.917 | 0.231 | myeloid/lymphoid or mixed-lineage leukemia (trithorax (Drosophila) homolog); translocated to 6 |
| MNT | 0.875 | 0.162 | 0.969 | 0.793 | 1.235 | 0.187 | MAX binding protein |
| MORF | 0.805 | 0.108 | 0.792 | 0.132 | 0.74 | 0.038 | histone acetyltransferase |
| MTA1L1 | 0.937 | 0.478 | 1.013 | 0.856 | 0.81 | 0.023 | metastasis-associated 1-like 1 |
| MTF1 | 1.016 | 0.872 | 1.028 | 0.811 | 0.766 | 0.003 | metal-regulatory transcription factor 1 |
| MYBL2 | 0.785 | 0.014 | 0.936 | 0.152 | 1.009 | 0.958 | v-myb avian myeloblastosis viral oncogene homolog-like 2 |
| MYCBP | 0.941 | 0.34 | 0.931 | 0.553 | 1.364 | 0.006 | c-myc binding protein |
| MYCL2 | 1.245 | 0.019 | 1.032 | 0.844 | 1.073 | 0.359 | v-myc avian myelocytomatosis viral oncogene homolog 2 |
| MYT1 | 0.916 | 0.203 | 1.098 | 0.217 | 0.822 | 0.013 | myelin transcription factor 1 |
| NEUROD6 | 0.84 | 0.142 | 1.07 | 0.349 | 0.792 | 3.86E-04 | neurogenic differentiation 6 |
| NFE2L1 | 0.813 | 0.005 | 1.085 | 0.372 | 1.065 | 0.672 | nuclear factor (erythroid-derived 2)-like 1 |
| NFIB | 0.801 | 0.021 | 1.08 | 0.465 | 1.01 | 0.623 | nuclear factor I/B |
| NFKBIA | 0.991 | 0.799 | 1.008 | 0.818 | 0.819 | 0.119 | nuclear factor of kappa light polypeptide gene enhancer in B-cells inhibitor, alpha |
| NHLH2 | 0.873 | 0.047 | 0.776 | 0.124 | 1.036 | 0.797 | nescient helix loop helix 2 |
| NR1H2 | 1.018 | 0.756 | 0.976 | 0.776 | 1.479 | 0.128 | nuclear receptor subfamily 1, group H, member 2 |
| NR1H3 | 0.945 | 0.626 | 0.739 | 0.205 | 0.734 | 0.025 | nuclear receptor subfamily 1, group H, member 3 |
| NR5A2 | 0.992 | 0.896 | 0.985 | 0.376 | 0.778 | 0.162 | nuclear receptor subfamily 5, group A, member 2 |
| NRF | 1.281 | 0.005 | 0.929 | 0.698 | 1.054 | 0.507 | transcription factor NRF |
| NRL | 1.116 | 0.137 | 1.012 | 0.52 | 1.281 | 0.082 | neural retina leucine zipper |
| PBX4 | 0.886 | 0.226 | 1.088 | 0.236 | 0.729 | 0.003 | pre-B-cell leukemia transcription factor 4 |
| PDEF | 1.292 | 0.016 | 1.003 | 0.962 | 1.097 | 0.027 | prostate epithelium-specific Ets transcription factor |
| PILB | 1.39 | 0.002 | 1.12 | 0.088 | 1.164 | 0.032 | pilin-like transcription factor |
| PKNOX2 | 1.056 | 0.228 | 1.084 | 0.289 | 1.262 | 0.216 | PBX/knotted 1 homeobox 2 |
| PLAG1 | 1.132 | 0.014 | 1.224 | 0.024 | 1.035 | 0.335 | pleiomorphic adenoma gene 1 |
| PMF1 | 1.031 | 0.465 | 0.81 | 0.019 | 0.953 | 0.585 | polyamine-modulated factor 1 |
| POU4F1 | 0.937 | 0.378 | 1.037 | 0.507 | 1.285 | 0.051 | POU domain, class 4, transcription factor 1 |
| POU4F3 | 0.936 | 0.202 | 0.894 | 0.602 | 1.211 | 0.03 | POU domain, class 4, transcription factor 3 |
| PPARBP | 0.823 | 0.04 | 1.079 | 0.278 | 1.043 | 0.719 | peroxisome proliferator activated receptor binding protein |
| PPARGC1 | 0.978 | 0.816 | 1.056 | 0.301 | 1.73 | 0.014 | peroxisome proliferative activated receptor, gamma, coactivator 1 |
| PROP1 | 1.20 | 0.009 | 0.939 | 0.376 | 0.948 | 0.439 | prophet of Pit1, paired-like homeodomain transcription factor |
| PSMC5 | 0.82 | 0.013 | 0.822 | 0.02 | 1.221 | 0.03 | proteasome (prosome, macropain) 26S subunit, ATPase, 5 |
| PTTG1IP | 1.135 | 0.083 | 1.018 | 0.463 | 1.532 | 0.098 | pituitary tumor-transforming 1 interacting protein |
| PURA | 0.746 | 0.016 | 1.395 | 0.01 | 0.897 | 0.169 | purine-rich element binding protein A |
| RBL2 | 0.859 | 0.121 | 1.231 | 0.023 | 0.803 | 0.319 | retinoblastoma-like 2 (p130) |
| RERE | 1.266 | 2.98E-04 | 1.114 | 0.26 | 1.184 | 0.105 | arginine-glutamic acid dipeptide (RE) repeats |
| RFX3 | 0.803 | 0.007 | 1.03 | 0.616 | 0.699 | 0.002 | regulatory factor X, 3 (influences HLA class II expression) |
| RNF10 | 0.987 | 0.857 | 0.996 | 0.975 | 0.787 | 0.015 | ring finger protein 10 |
| RNF14 | 0.916 | 0.172 | 0.961 | 0.729 | 2.058 | 0.025 | ring finger protein 14 |
| RNF15 | 0.934 | 0.323 | 1.104 | 0.31 | 0.81 | 0.015 | ring finger protein 15 |
| SAP30 | 1.203 | 0.03 | 0.843 | 0.268 | 0.985 | 0.703 | sin3-associated polypeptide, 30kD |
| SCAND2 | 0.936 | 0.191 | 1.122 | 0.179 | 0.757 | 0.06 | SCAN domain-containing 2 |
| SIX3 | 0.753 | 0.003 | 0.934 | 0.401 | 0.95 | 0.547 | sine oculis homeobox (Drosophila) homolog 3 |
| SIX6 | 1.263 | 0.01 | 1.006 | 0.881 | 1.015 | 0.783 | sine oculis homeobox (Drosophila) homolog 6 |
| SMARCA4 | 1.013 | 0.825 | 0.988 | 0.806 | 1.347 | 0.124 | selective LIM binding factor |
| SMARCB1 | 0.825 | 0.036 | 1.01 | 0.789 | 0.814 | 0.183 | SWI/SNF related, matrix associated, actin dependent regulator of chromatin, subfamily a, member 4 |
| SOX14 | 0.826 | 0.031 | 0.833 | 0.085 | 1.185 | 0.067 | SWI/SNF related, matrix associated, actin dependent regulator of chromatin, subfamily b, member 1 |
| SREBF1 | 0.825 | 0.079 | 1.045 | 0.479 | 0.834 | 0.014 | SRY (sex determining region Y)-box 14 |
| SSX4 | 1.106 | 0.075 | 1.085 | 0.381 | 1.417 | 0.022 | sterol regulatory element binding transcription factor 1 |
| STAT3 | 0.802 | 0.034 | 1.254 | 0.121 | 0.885 | 0.009 | signal transducer and activator of transcription 3 (acute-phase response factor) |
| SUPT4H1 | 0.828 | 0.197 | 1.189 | 0.055 | 0.799 | 0.12 | suppressor of Ty (S.cerevisiae) 4 homolog |
| TAF-172 | 0.871 | 0.314 | 1.048 | 0.712 | 0.784 | 0.007 | TBP-associated factor 172 |
| TAF1B | 0.744 | 0.026 | 1.013 | 0.921 | 0.948 | 0.568 | TATA box binding protein (TBP)-associated factor, RNA polymerase I, B, 63kD |
| TAF1C | 0.991 | 0.918 | 1.062 | 0.466 | 0.829 | 0.015 | TATA box binding protein (TBP)-associated factor, RNA polymerase I, C, 110kD |
| TAF2B | 0.782 | 0.125 | 0.919 | 0.02 | 1.212 | 0.168 | TATA box binding protein (TBP)-associated factor, RNA polymerase II, B, 150kD |
| TAF2H | 0.802 | 0.097 | 1.062 | 0.611 | 0.659 | 0.019 | TATA box binding protein (TBP)-associated factor, RNA polymerase II, H, 30kD |
| TAF2I | 0.796 | 0.025 | 1.024 | 0.702 | 0.871 | 0.062 | TATA box binding protein (TBP)-associated factor, RNA polymerase II, I, 28kD |
| TAF2K | 0.81 | 0.007 | 1.007 | 0.946 | 0.955 | 0.656 | TATA box binding protein (TBP)-associated factor, RNA polymerase II, K |
| TBX15 | 1.423 | 0.008 | 1.277 | 0.106 | 1.20 | 0.029 | T-box 15 |
| TBX21 | 0.907 | 0.247 | 1.048 | 0.371 | 1.232 | 0.277 | T-box 21 |
| TCF21 | 0.702 | 0.016 | 1.042 | 0.46 | 0.83 | 0.003 | transcription factor 21 |
| TCF8 | 0.943 | 0.296 | 0.734 | 0.091 | 0.754 | 0.005 | transcription factor 8 (represses interleukin 2 expression) |
| TGFB1I1 | 1.013 | 0.794 | 0.83 | 0.042 | 0.927 | 0.466 | transforming growth factor beta 1 induced transcript 1 |
| TITF1 | 0.984 | 0.867 | 0.815 | 0.028 | 1.129 | 0.416 | TG-interacting factor (TALE family homeobox) |
| TNRC12 | 0.991 | 0.802 | 0.699 | 0.014 | 0.96 | 0.538 | trinucleotide repeat containing 12 |
| TNRC5 | 0.876 | 0.466 | 1.432 | 0.161 | 0.668 | 0.137 | trinucleotide repeat containing 5 |
| TRIP15 | 0.799 | 0.013 | 0.976 | 0.589 | 1.41 | 0.141 | thyroid receptor interacting protein 15 |
| UBTF | 1.129 | 0.072 | 1.293 | 0.1 | 1.079 | 0.274 | upstream binding transcription factor, RNA polymerase I |
| WHSC1 | 1.248 | 0.065 | 1.054 | 0.41 | 1.221 | 0.049 | Wolf-Hirschhorn syndrome candidate 1 |
| XBP1 | 0.824 | 0.236 | 0.888 | 0.165 | 1.076 | 0.388 | X-box binding protein 1 |
| ZF5128 | 1.004 | 0.968 | 1.054 | 0.406 | 1.238 | 0.021 | zinc finger protein |
| ZFP91 | 0.922 | 0.377 | 1.043 | 0.384 | 0.805 | 0.467 | zinc finger protein 91 homolog (mouse) |
| ZNF10 | 1.626 | 0.01 | 1.297 | 0.259 | 0.997 | 0.951 | zinc finger protein 10 (KOX 1) |
| ZNF174 | 1.357 | 0.104 | 0.907 | 0.385 | 1.503 | 0.022 | zinc finger protein 174 |
| ZNF20 | 1.239 | 0.013 | 0.952 | 0.399 | 1.131 | 0.009 | zinc finger protein 20 (KOX 13) |
| ZNF212 | 1.312 | 0.008 | 1.08 | 0.175 | 0.857 | 0.38 | zinc finger protein 212 |
| ZNF239 | 0.993 | 0.907 | 0.881 | 0.092 | 1.295 | 0.058 | zinc finger protein 239 |
| ZNF274 | 0.797 | 0.372 | 0.818 | 0.045 | 0.849 | 0.048 | zinc finger protein 274 |
| ZNF281 | 1.314 | 0.006 | 1.092 | 0.081 | 1.014 | 0.628 | zinc finger protein 281 |
| ZNF286 | 1.058 | 0.28 | 1.16 | 0.119 | 1.495 | 0.026 | zinc finger protein 286 |
| ZNF288 | 0.795 | 0.026 | 0.889 | 0.154 | 1.197 | 0.031 | zinc finger protein 288 |
| ZNF6 | 1.034 | 0.73 | 0.985 | 0.825 | 1.326 | 0.271 | zinc finger protein 6 (CMPX1) |
| ZNF7 | 0.823 | 0.02 | 0.902 | 0.18 | 1.198 | 0.369 | zinc finger protein 7 (KOX 4, clone HF.16) |
| ZNF76 | 0.913 | 0.206 | 0.813 | 0.022 | 1.07 | 0.467 | zinc finger protein 76 (expressed in testis) |
| ZNF79 | 1.359 | 0.035 | 1.06 | 0.321 | 1.549 | 0.003 | zinc finger protein 79 (pT7) |
| ZNF90 | 1.451 | 0.034 | 1.127 | 0.039 | 1.157 | 0.21 | zinc finger protein 90 (HTF9) |
| ZNF93 | 1.31 | 0.029 | 1.20 | 0.041 | 1.346 | 0.002 | zinc finger protein 93 (HTF34) |
| ZXDA/B | 0.94 | 0.276 | 0.988 | 0.818 | 1.226 | 0.235 | zinc finger, X-linked, duplicated B |
