# Supplementary material for: Large Scale Gene Expression Profiles of Regenerating Inner Ear Sensory Epithelia
Source: PLoS One. 2007 Jun 13;2(6):e525. doi: 10.1371/journal.pone.0000525 (PMC1888727; doi:10.1371/journal.pone.0000525)
Supplement: Table S2 — Utricle Neomycin Differential Expression>1.2-fold and P< = 0.05 (0.06 MB PDF) [file pone.0000525.s003.pdf]

Supplemental Table S2

		O hr		24 hr		48 hr			
**Gene ID**	**Entrez**	**Fold change**	**P-value**	**Fold Change**	**P-value**	**Fold Change**	**P-value**	**Updated syml**	**Updated description**
ASH2L	9070	0.99	0.89	0.781	0.006	1.028	0.548	ASH2L	ash2 (absent, small, or homeotic)-like (Drosophila)
ATF2	1386	0.804	0.021	1.013	0.886	0.754	0.39	ATF2	activating transcription factor 2
BCL11A	53335	1.327	0.127	1.051	0.577	1.334	0.026	BCL11A	B-cell CLL/lymphoma 11A (zinc finger protein)
BCL11B	64919	0.842	0.146	0.919	0.262	0.785	0.001	BCL11B	B-cell CLL/lymphoma 11B (zinc finger protein)
BRD1	23774	0.635	0.006	0.94	0.646	0.603	0.002	BRD1	bromodomain containing 1
C21orf18	54093	0.538	0.029	0.954	0.779	0.603	0.003	SETD4	SET domain containing 4
CBX3	11335	1.21	0.046	1.017	0.772	0.951	0.532	CBX3	Chromobox homolog 3 (HP1 gamma homolog, Drosophila)
CBX4	8535	0.588	0.037	1.192	0.074	0.823	0.511	CBX4	chromobox homolog 4 (Pc class homolog, Drosophila)
CDK7	1022	1.022	0.816	0.871	0.216	0.829	0.036	CDK7	cyclin-dependent kinase 7 (MO15 homolog, Xenopus laevis, cdk-activating
CEBPG	1054	0.646	0.04	1.236	0.43	0.506	0.008	CEBPG	CCAAT/enhancer binding protein (C/EBP), gamma
CITED1	4435	1.521	0.036	0.689	0.182	0.985	0.885	CITED1	Cbp/p300-interacting transactivator, with Glu/Asp-rich carboxy-terminal d
CREBL1	1388	0.994	0.902	1.072	0.088	1.322	0.032	CREBL1	cAMP responsive element binding protein-like 1
CREG	8804	0.95	0.424	1.124	0.159	0.807	0.045	CREG1	Cellular repressor of E1A-stimulated genes 1
CROC4	10485	0.907	0.062	0.689	0.05	0.972	0.857	CROC4	Transcriptional activator of the c-fos promoter
CRSP6	9440	0.694	0.013	1.062	0.705	0.989	0.949	CRSP6	cofactor required for Sp1 transcriptional activation, subunit 6, 77kDa
CRX	1406	1.17	0.057	1.2	0.038	1.048	0.268	CRX	cone-rod homeobox
CSDA	8531	1.056	0.484	0.973	0.747	1.321	0.008	CSDA	cold shock domain protein A
CSRP2	1466	1.006	0.939	0.914	0.393	0.778	0.049	CSRP2	cysteine and glycine-rich protein 2
CTNNB1	1499	0.789	0.092	0.89	0.101	3.097	0.035	CTNNB1	catenin (cadherin-associated protein), beta 1, 88kDa
CUTL1	1523	0.857	0.012	1.097	0.14	1.381	0.006	CUTL1	Cut-like 1, CCAAT displacement protein (Drosophila)
DEAF1	10522	1.321	0.002	1.275	0.24	1.003	0.97	DEAF1	deformed epidermal autoregulatory factor 1 (Drosophila)
DKFZP434B0	25851	0.69	0.112	0.905	0.256	0.807	0.001	DKFZP434B03	DKFZP434B0335 protein
DLX6	1750	0.784	0.016	0.85	0.087	0.991	0.953	DLX6	distal-less homeobox 6
DNAJ	10294	0.724	0.014	0.981	0.714	0.711	0.061	DNAJA2	DnaJ (Hsp40) homolog, subfamily A, member 2
DUX2	26583	1.254	0.01	1.198	0.086	1.136	0.041	DUX2	double homeobox, 2
E2F2	1870	0.927	0.627	1.203	0.006	0.558	0.087	E2F2	E2F transcription factor 2
E2F5	1875	0.778	0.015	0.949	0.446	1.07	0.45	E2F5	E2F transcription factor 5, p130-binding
EBF	1879	0.789	0.009	1.08	0.413	1.16	0.184	EBF	early B-cell factor
EOMES	8320	0.979	0.81	0.992	0.877	1.209	1.09E-04	EOMES	eomesodermin homolog (Xenopus laevis)
ERCC6	2074	0.928	0.253	0.964	0.54	1.203	0.007	ERCC6	excision repair cross-complementing rodent repair deficiency, complement
ETV5	2119	0.952	0.441	0.829	0.028	1.017	0.876	ETV5	ets variant gene 5 (ets-related molecule)
EZH2	2146	1.076	0.383	0.883	0.917	1.234	0.005	EZH2	enhancer of zeste homolog 2 (Drosophila)
FHL1	2273	0.709	0.062	1.042	0.512	0.743	4.63E-05	FHL1	four and a half LIM domains 1
FLJ10251	55693	1.225	0.002	1.229	0.135	1.057	0.29	JMJD2D	Jumonji domain containing 2D
FLJ10891	55762	1.25	0.005	1.174	0.22	1.025	0.356	ZNF701	Zinc finger protein 701
FLJ12827	79797	1.219	0.018	1.01	0.811	1.143	0.33	FLJ12827	Zinc finger protein 408
FLJ13590	79898	1.248	0.006	1.023	0.458	1.207	0.029	ZNF613	Zinc finger protein 613
FLJ20595	54993	1.346	0.01	0.917	0.252	0.91	0.055	ZSCAN2	Zinc finger and SCAN domain containing 2
FOG2	23414	0.974	0.736	0.808	0.113	0.76	0.001	ZFPM2	Friend of GATA2 /// zinc finger protein, multitype 2
GIOT-2	51710	0.977	0.813	0.689	0.047	0.702	0.036	ZNF44	Zinc finger protein 44
GTF2A1	2957	1.431	0.013	1.171	0.101	1.321	0.041	GTF2A1	general transcription factor IIA, 1, 19/37kDa
GTF2E1	2960	1.063	0.399	0.92	0.044	1.22	0.007	GTF2E1	general transcription factor IIE, polypeptide 1, alpha 56kDa
GTF2F1	2962	0.785	0.001	0.939	0.237	1.18	0.068	GTF2F1	general transcription factor IIF, polypeptide 1, 74kDa
GTF2H1	2965	0.936	0.161	1.023	0.763	1.283	0.013	GTF2H1	general transcription factor IIH, polypeptide 1, 62kDa
GTF2H3	2967	1.049	0.505	1.017	0.697	1.2	0.006	GTF2H3	general transcription factor IIH, polypeptide 3, 34kDa
GTF3C4	9329	1.286	0.022	0.876	0.829	1.106	0.165	GTF3C4	general transcription factor IIIC, polypeptide 4, 90kDa
HEY1	23462	0.786	0.033	0.922	0.517	1.02	0.837	HEY1	hairy/enhancer-of-split related with YRPW motif 1
HHEX	3087	1.123	0.031	1.023	0.665	1.452	0.046	HHEX	homeobox, hematopoietically expressed
HIF1A	3091	0.816	0.081	0.703	0.048	1.071	0.616	HIF1A	hypoxia-inducible factor 1, alpha subunit (basic helix-loop-helix transcripti
HIRA	7290	1.095	0.328	0.948	0.194	1.22	0.029	HIRA	HIR histone cell cycle regulation defective homolog A (S. cerevisiae)
HIVEP1	3096	1.013	0.866	1.038	0.672	1.243	0.041	HIVEP1	human immunodeficiency virus type I enhancer binding protein 1

H-L(3)MBT	83746	1.474	0.002	1.041	0.739	0.968	0.717	L3MBTL2	L(3)mbt-like 2 (Drosophila)
HNF3A	3169	1.01	0.787	1.262	8.52E-05	0.801	0.039	FOXA1	Forkhead box A1
HOXA13	3209	0.67	0.008	0.947	0.502	0.934	0.437	HOXA13	homeobox A13
HOXB7	3217	1.135	0.07	0.976	0.724	1.277	0.018	HOXB7	homeobox B7
HOXD12	3238	1.066	0.385	1.127	0.011	1.367	0.047	HOXD12	homeobox D12
HOXD8	3234	1.39	0.035	1.226	0.147	1.339	0.312	HOXD8	Homeobox D8
HSAJ2425	55566	1.288	0.043	0.978	0.788	1.282	0.127	HSAJ2425	p65 protein
HSF1	3297	0.937	0.614	0.904	0.61	0.58	0.015	HSF1	Heat shock transcription factor 1
HSF2BP	11077	0.892	0.272	0.749	0.075	0.729	0.044	HSF2BP	Heat shock transcription factor 2 binding protein
HSPC018	9567	1.11	0.134	0.969	0.404	1.2	0.014	GTPBP1	GTP binding protein 1
ILF1	3607	0.645	0.018	0.944	0.475	1.06	0.089	FOXK2	Forkhead box K2
IRF2	3660	0.681	0.029	1.188	0.117	0.829	0.058	IRF2	Interferon regulatory factor 2
ISGF3G	10379	0.799	0.008	1.051	0.382	1.054	0.316	ISGF3G	interferon-stimulated transcription factor 3, gamma 48kDa
KIAA0130	9862	1.35	0.003	1.109	0.392	0.943	0.687	THRAP4	Thyroid hormone receptor associated protein 4
KIAA0173	9654	1.365	0.009	0.932	0.707	1.289	0.11	TTLL4	Tubulin tyrosine ligase-like family, member 4
KIAA0395	23051	0.983	0.785	0.871	0.049	1.29	0.044	ZHX3	Zinc fingers and homeoboxes 3
KIAA1041	22887	0.665	0.016	0.837	0.148	0.934	0.624	FOXJ3	Forkhead box J3
KIAA1528	113878	1.187	0.033	1.028	0.408	1.353	0.002	DTX2	Deltex homolog 2 (Drosophila)
LHX4	89884	1.257	0.025	1.024	0.665	0.964	0.091	LHX4	LIM homeobox 4
LOC51058	51058	1.268	0.089	1.031	0.853	1.497	0.052	ZNF691	Zinc finger protein 691
LOC51131	51131	1.201	0.036	1.009	0.855	1.21	0.097	PHF11	PHD finger protein 11
LOC57209	57209	1.631	0.019	1.157	0.28	1.29	0.014	ZNF248	Zinc finger protein 248
LZLP	LZLP	1.157	0.023	0.954	0.421	1.324	0.012	LZLP	Discontinued
MADH2	4087	1.03	0.678	1.102	0.191	1.32	0.034	SMAD2	SMAD family member 2 /// mothers against decapentaplegic homolog 2
MAPK8IP1	9479	1.228	0.054	0.642	0.008	0.889	0.231	MAPK8IP1	mitogen-activated protein kinase 8 interacting protein 1
MLLT6	4302	1.144	0.118	0.749	0.046	0.917	0.231	MLLT6	myeloid/lymphoid or mixed-lineage leukemia (trithorax homolog, Drosoph
MORF	23522	0.805	0.108	0.792	0.132	0.74	0.038	MYST4	MYST histone acetyltransferase (monocytic leukemia) 4
MTA1L1	9219	0.937	0.478	1.013	0.856	0.81	0.023	MTA2	Metastasis associated 1 family, member 2
MTF1	4520	1.016	0.872	1.028	0.811	0.766	0.003	MTF1	metal-regulatory transcription factor 1
MYBL2	4605	0.785	0.014	0.936	0.152	1.009	0.958	MYBL2	v-myb myeloblastosis viral oncogene homolog (avian)-like 2
MYCBP	26292	0.941	0.34	0.931	0.553	1.364	0.006	MYCBP	c-myc binding protein
MYCL2	4611	1.245	0.019	1.032	0.844	1.073	0.359	MYCL2	V-myc myelocytomatosis viral oncogene homolog 2 (avian)
MYT1	4661	0.916	0.203	1.098	0.217	0.822	0.013	MYT1	myelin transcription factor 1
NEUROD6	63974	0.84	0.142	1.07	0.349	0.792	3.86E-04	NEUROD6	neurogenic differentiation 6
NFE2L1	4779	0.813	0.005	1.085	0.372	1.065	0.672	NFE2L1	nuclear factor (erythroid-derived 2)-like 1
NFIB	4781	0.801	0.021	1.08	0.465	1.01	0.623	NFIB	nuclear factor I/B
NR1H3	10062	0.945	0.626	0.739	0.205	0.734	0.025	NR1H3	nuclear receptor subfamily 1, group H, member 3
NRF	55922	1.281	0.005	0.929	0.698	1.054	0.507	NKRF	NF-kappaB repressing factor
PBX4	80714	0.886	0.226	1.088	0.236	0.729	0.003	PBX4	pre-B-cell leukemia transcription factor 4
PDEF	25803	1.292	0.016	1.003	0.962	1.097	0.027	SPDEF	SAM pointed domain containing ets transcription factor /// prostate epithe
PILB	22921	1.39	0.002	1.12	0.088	1.164	0.032	MSRB2	Methionine sulfoxide reductase B2 /// pilin-like transcription factor
PLAG1	5324	1.132	0.014	1.224	0.024	1.035	0.335	PLAG1	pleiomorphic adenoma gene 1
PMF1	11243	1.031	0.465	0.81	0.019	0.953	0.585	PMF1	polyamine-modulated factor 1
POU4F1	5457	0.937	0.378	1.037	0.507	1.285	0.051	POU4F1	POU domain, class 4, transcription factor 1
POU4F3	5459	0.936	0.202	0.894	0.602	1.211	0.03	POU4F3	POU domain, class 4, transcription factor 3
PPARBP	5469	0.823	0.04	1.079	0.278	1.043	0.719	PPARBP	PPAR binding protein
PPARGC1	10891	0.978	0.816	1.056	0.301	1.73	0.014	PPARGC1A	Peroxisome proliferator-activated receptor gamma, coactivator 1 alpha
PROP1	5626	1.2	0.009	0.939	0.376	0.948	0.439	PROP1	prophet of Pit1, paired-like homeodomain transcription factor
PSMC5	5705	0.82	0.013	0.822	0.02	1.221	0.03	PSMC5	proteasome (prosome, macropain) 26S subunit, ATPase, 5
PURA	5813	0.746	0.016	1.395	0.01	0.897	0.169	PURA	purine-rich element binding protein A
RBL2	5934	0.859	0.121	1.231	0.023	0.803	0.319	RBL2	retinoblastoma-like 2 (p130)
RERE	473	1.266	2.98E-04	1.114	0.26	1.184	0.105	RERE	arginine-glutamic acid dipeptide (RE) repeats
RFX3	5991	0.803	0.007	1.03	0.616	0.699	0.002	RFX3	regulatory factor X, 3 (influences HLA class II expression)
RNF10	9921	0.987	0.857	0.996	0.975	0.787	0.015	RNF10	ring finger protein 10
RNF14	9604	0.916	0.172	0.961	0.729	2.058	0.025	RNF14	ring finger protein 14

RNF15	10475	0.934	0.323	1.104	0.31	0.81	0.015	TRIM38	Tripartite motif-containing 38
SAP30	8819	1.203	0.03	0.843	0.268	0.985	0.703	SAP30	Sin3A-associated protein, 30kDa
SIX3	6496	0.753	0.003	0.934	0.401	0.95	0.547	SIX3	sine oculis homeobox homolog 3 (Drosophila)
SIX6	4990	1.263	0.01	1.006	0.881	1.015	0.783	SIX6	sine oculis homeobox homolog 6 (Drosophila)
SMARCB1	6598	0.825	0.036	1.01	0.789	0.814	0.183	SMARCB1	SWI/SNF related, matrix associated, actin dependent regulator of chromat
SOX14	8403	0.826	0.031	0.833	0.085	1.185	0.067	SOX14	SRY (sex determining region Y)-box 14
SREBF1	6720	0.825	0.079	1.045	0.479	0.834	0.014	SREBF1	sterol regulatory element binding transcription factor 1
SSX4	10214	1.106	0.075	1.085	0.381	1.417	0.022	SSX2	synovial sarcoma, X breakpoint 2 /// synovial sarcoma, X breakpoint 4 ///
STAT3	6774	0.802	0.034	1.254	0.121	0.885	0.009	STAT3	signal transducer and activator of transcription 3 (acute-phase response fa
TAF-172	9044	0.871	0.314	1.048	0.712	0.784	0.007	BTAF1	BTAF1 RNA polymerase II, B-TFIID transcription factor-associated, 170kD
TAF1B	9014	0.744	0.026	1.013	0.921	0.948	0.568	TAF1B	TATA box binding protein (TBP)-associated factor, RNA polymerase I, B, 6
TAF1C	9013	0.991	0.918	1.062	0.466	0.829	0.015	TAF1C	TATA box binding protein (TBP)-associated factor, RNA polymerase I, C, 1
TAF2H	6881	0.802	0.097	1.062	0.611	0.659	0.019	TAF10	TAF10 RNA polymerase II, TATA box binding protein (TBP)-associated fact
TAF2I	6882	0.796	0.025	1.024	0.702	0.871	0.062	TAF11	TAF11 RNA polymerase II, TATA box binding protein (TBP)-associated fact
TAF2K	6884	0.81	0.007	1.007	0.946	0.955	0.656	TAF13	TAF13 RNA polymerase II, TATA box binding protein (TBP)-associated fact
TBX15	6913	1.423	0.008	1.277	0.106	1.2	0.029	TBX15	T-box 15
TCF21	6943	0.702	0.016	1.042	0.46	0.83	0.003	TCF21	transcription factor 21
TCF8	6935	0.943	0.296	0.734	0.091	0.754	0.005	TCF8	transcription factor 8 (represses interleukin 2 expression)
TGFB1I1	7041	1.013	0.794	0.83	0.042	0.927	0.466	TGFB1I1	transforming growth factor beta 1 induced transcript 1
TITF1	7080	0.984	0.867	0.815	0.028	1.129	0.416	TITF1	thyroid transcription factor 1
TNRC12	57634	0.991	0.802	0.699	0.014	0.96	0.538	EP400	E1A binding protein p400
TRIP15	9318	0.799	0.013	0.976	0.589	1.41	0.141	COPS2	COP9 constitutive photomorphogenic homolog subunit 2 (Arabidopsis)
WHSC1	7468	1.248	0.065	1.054	0.41	1.221	0.049	WHSC1	Wolf-Hirschhorn syndrome candidate 1
ZF5128	25799	1.004	0.968	1.054	0.406	1.238	0.021	ZNF324	Zinc finger protein 324
ZNF10	7556	1.626	0.01	1.297	0.259	0.997	0.951	ZNF10	zinc finger protein 10
ZNF174	7727	1.357	0.104	0.907	0.385	1.503	0.022	ZNF174	zinc finger protein 174
ZNF20	7568	1.239	0.013	0.952	0.399	1.131	0.009	ZNF20	zinc finger protein 20
ZNF212	7988	1.312	0.008	1.08	0.175	0.857	0.38	ZNF212	zinc finger protein 212
ZNF274	10782	0.797	0.372	0.818	0.045	0.849	0.048	ZNF274	zinc finger protein 274
ZNF281	23528	1.314	0.006	1.092	0.081	1.014	0.628	ZNF281	zinc finger protein 281
ZNF286	57335	1.058	0.28	1.16	0.119	1.495	0.026	ZNF286	zinc finger protein 286
ZNF288	26137	0.795	0.026	0.889	0.154	1.197	0.031	ZBTB20	Zinc finger and BTB domain containing 20
ZNF7	7553	0.823	0.02	0.902	0.18	1.198	0.369	ZNF7	zinc finger protein 7
ZNF76	7629	0.913	0.206	0.813	0.022	1.07	0.467	ZNF76	zinc finger protein 76 (expressed in testis)
ZNF79	7633	1.359	0.035	1.06	0.321	1.549	0.003	ZNF79	zinc finger protein 79
ZNF90	7643	1.451	0.034	1.127	0.039	1.157	0.21	ZNF90	zinc finger protein 90
ZNF93	81931	1.31	0.029	1.2	0.041	1.346	0.002	ZNF93	Zinc finger protein 93

g kinase)

domain, 1

ntation group 6

tion factor)

hila); translocated to, 6

elium-specific Ets transcription

atin, subfamily b, member 1

/ synovial sarcoma, X breakpo
factor)
Da (Mot1 homolog, S. cerevisia
63kDa
110kDa
ctor, 30kDa
ctor, 28kDa
ctor, 18kDa
