# Supplementary material for: Large Scale Gene Expression Profiles of Regenerating Inner Ear Sensory Epithelia
Source: PLoS One. 2007 Jun 13;2(6):e525. doi: 10.1371/journal.pone.0000525 (PMC1888727; doi:10.1371/journal.pone.0000525)
Supplement: Table S3 — Utricle Laser Differentially Expressed Genes (261 total). This listing shows all genes that exhibited>1.2-fold changes in expression, irrespective of P-value. For p-value filtered data see Table S4. (0.36 MB DOC) [file pone.0000525.s004.doc]

Supplemental Table S3.

|  | **30min** | | **1hr** | | **2hr** | | **3hr** |  |  |
| --- | --- | --- | --- | --- | --- | --- | --- | --- | --- |
| **Gene ID** | **Fold change** | **P-value** | **Fold Change** | **P-value** | **Fold Change** | **P-value** | **Fold Change** | **P-value** | **Notes/Description** |
| ABT1 | 0.795 | 0.428 | 1.025 | 0.304 | 0.89 | 0.152 | 1.076 | 0.102 | TATA-binding protein-binding protein |
| AF5Q31 | 0.847 | 0.425 | 1.378 | 0.47 | 0.752 | 0.954 | 0.753 | 0.008 | ALL1 fused gene from 5q31 |
| ALY | 0.795 | 0.743 | 1.013 | 0.619 | 1.067 | 0.973 | 1.086 | 0.501 | transcriptional coactivator |
| AR | 1.153 | 0.618 | 1.24 | 0.362 | 0.73 | 0.365 | 0.782 | 0.091 | androgen receptor (dihydrotestosterone receptor) |
| ATBF1 | 1.096 | 0.201 | 0.647 | 0.066 | 1.594 | 0.662 | 0.768 | 0.774 | AT-binding transcription factor 1 |
| ATF7 | 1.248 | 0.576 | 1.121 | 0.85 | 1.103 | 0.756 | 1.023 | 0.718 | activating transcription factor 7 |
| BACH2 | 0.684 | 0.9 | 1.409 | 0.861 | 0.662 | 0.811 | 1.422 | 0.116 | BTB and CNC homology 1, basic leucine zipper transcription factor 2 |
| BAPX1 | 1.056 | 0.596 | 1.363 | 0.963 | 0.734 | 0.722 | 0.742 | 0.096 | bagpipe homeobox (Drosophila) homolog 1 |
| BAZ1B | 1.122 | 0.267 | 1.086 | 0.472 | 0.82 | 0.594 | 1.081 | 0.191 | bromodomain adjacent to zinc finger domain, 1B |
| BCL11A | 0.879 | 0.959 | 0.801 | 0.166 | 1.251 | 0.382 | 1.371 | 0.005 | B-cell CLL/lymphoma 11A (zinc finger protein) |
| BLZF1 | 0.777 | 0.056 | 1.003 | 0.038 | 0.923 | 0.172 | 1.215 | 0.004 | basic leucine zipper nuclear factor 1 (JEM-1) |
| BRD1 | 0.829 | 0.846 | 1.462 | 0.715 | 0.665 | 0.791 | 0.802 | 0.079 | bromodomain-containing 1 |
| BRD2 | 0.954 | 0.124 | 0.87 | 0.06 | 1.06 | 0.533 | 1.223 | 0.982 | bromodomain-containing 2 |
| BRD4 | 0.67 | 0.208 | 0.697 | 0.244 | 1.271 | 0.785 | 1.101 | 0.004 | bromodomain-containing 4 |
| BRD7 | 0.807 | 0.556 | 1.071 | 0.733 | 1.401 | 0.196 | 1.067 | 0.051 | bromodomain-containing 7 |
| BS69 | 0.919 | 0.358 | 1.152 | 0.928 | 0.976 | 0.217 | 0.825 | 0.532 | adenovirus 5 E1A binding protein |
| CCT4 | 1.082 | 0.062 | 1.167 | 0.024 | 1.045 | 0.55 | 1.243 | 0.27 | chaperonin containing TCP1, subunit 4 (delta) |
| CDK7 | 1.142 | 0.015 | 0.807 | 0.94 | 0.949 | 0.336 | 0.778 | 0.876 | cyclin-dependent kinase 7 (homolog of Xenopus MO15 cdk-activating kinase) |
| CEBPE | 0.929 | 0.495 | 0.918 | 0.744 | 0.966 | 0.546 | 0.742 | 0.327 | CCAAT/enhancer binding protein (C/EBP), epsilon |
| CEBPG | 0.481 | 0.09 | 2.27 | 0.076 | 0.817 | 0.209 | 1.351 | 0.054 | CCAAT/enhancer binding protein (C/EBP), gamma |
| CIAO1 | 1.156 | 0.502 | 1.056 | 0.297 | 0.998 | 0.944 | 1.234 | 0.174 | WD40 protein Ciao1 |
| CREM | 1.049 | 0.856 | 0.994 | 0.43 | 0.805 | 0.102 | 1.222 | 0.103 | cAMP responsive element modulator |
| CRSP6 | 0.78 | 0.16 | 1.104 | 0.555 | 1.22 | 0.416 | 1.112 | 0.061 | cofactor required for Sp1 transcriptional activation, subunit 6 (77kD) |
| CRSP7 | 0.962 | 0.765 | 1.101 | 0.559 | 0.791 | 0.501 | 1.078 | 0.076 | cofactor required for Sp1 transcriptional activation, subunit 7 (70kD) |
| CSRP2 | 0.798 | 0.255 | 1.078 | 0.548 | 0.835 | 0.307 | 0.851 | 0.027 | cysteine and glycine-rich protein 2 |
| DACH | 1.032 | 0.092 | 1.096 | 0.358 | 0.766 | 0.789 | 0.967 | 0.016 | dachshund (Drosophila) homolog |
| DDIT3 | 0.898 | 0.222 | 0.995 | 0.953 | 0.925 | 0.177 | 0.827 | 0.385 | DNA-damage-inducible transcript 3 |
| DEAF1 | 1.073 | 0.267 | 1.132 | 0.015 | 1.23 | 0.769 | 1.276 | 0.617 | deformed epidermal autoregulatory factor 1 (Drosophila) |
| DKFZP434P1750 | 0.869 | 0.008 | 0.991 | 0.682 | 1.135 | 0.911 | 1.301 | 0.162 | TBC1 domain family, member 10B (TBC1D10B) |
| DKFZp547H236 | 1.238 | 0.767 | 1.289 | 0.734 | 1.01 | 0.254 | 0.895 | 0.032 | myeloid ecotropic viral integration site 1 homolog 3 (MEIS3) |
| DKFZp762K2015 | 0.899 | 0.567 | 1.104 | 0.962 | 1.046 | 0.069 | 1.221 | 0.803 | SWI/SNF-related, matrix-associated actin-dependent regulator of chromatin, subfamily a, containing DEAD/H box 1 (SMARCAD1) |
| DKFZp762M136 | 1.124 | 0.348 | 1.016 | 0.667 | 1.081 | 0.728 | 1.337 | 0.089 | fem-1 homolog a (C.elegans) (FEM1A) |
| DLX3 | 0.698 | 0.467 | 1.051 | 0.947 | 0.907 | 0.909 | 0.849 | 0.033 | distal-less homeobox 3 |
| DRPLA | 0.963 | 0.156 | 0.906 | 0.29 | 0.586 | 0.975 | 1.087 | 0.275 | dentatorubral-pallidoluysian atrophy (atrophin-1) |
| DSIPI | 1.051 | 0.068 | 1.305 | 0.936 | 0.887 | 0.294 | 0.914 | 0.217 | delta sleep inducing peptide, immunoreactor |
| E4F1 | 0.946 | 0.014 | 1.009 | 0.101 | 1.346 | 0.227 | 1.297 | 0.421 | E4F transcription factor 1 |
| EED | 1.327 | 0.837 | 0.839 | 0.724 | 0.796 | 0.665 | 0.904 | 0.771 | embryonic ectoderm development |
| ELF3 | 1.291 | 0.007 | 1.045 | 0.314 | 0.825 | 0.595 | 1.059 | 0.16 | E74-like factor 3 (ets domain transcription factor, epithelial-specific ) |
| ELK4 | 1.04 | 0.034 | 0.769 | 0.603 | 0.978 | 0.025 | 1.154 | 0.031 | E74-like factor 4 (ets domain transcription factor) |
| EN2 | 0.91 | 0.797 | 1.254 | 0.955 | 1.151 | 0.482 | 1.123 | 0.061 | engrailed homolog 2 |
| ESR1 | 0.984 | 0.095 | 0.929 | 0.504 | 1.261 | 0.813 | 1.019 | 0.001 | estrogen receptor 1 |
| ETV1 | 0.929 | 0.286 | 1.496 | 0.895 | 1.328 | 0.833 | 1.006 | 0.005 | ets variant gene 1 |
| FHL1 | 0.681 | 0.524 | 1.406 | 0.467 | 0.928 | 0.814 | 1.26 | 0.047 | four and a half LIM domains 1 |
| FLJ10142 | 1.066 | 0.016 | 1.356 | 0.625 | 0.958 | 0.904 | 1.233 | 0.061 | likely ortholog of mouse and zebrafish forebrain embryonic zinc finger-like (FEZL) |
| FLJ10697 | 0.811 | 0.11 | 1.002 | 0.482 | 1.122 | 0.911 | 1.362 | 1.60E-04 | zinc finger protein ZNF532 |
| FLJ11186 | 0.9 | 0.136 | 0.713 | 0.294 | 1.491 | 0.345 | 1.193 | 0.007 | chromosome 14 open reading frame 106 |
| FLJ12517 | 1.193 | 0.052 | 0.766 | 0.92 | 1.012 | 0.026 | 1.218 | 0.177 | jumonji domain containing 4 (JMJD4) |
| FLJ12606 | 0.96 | 0.249 | 0.939 | 0.187 | 1.054 | 0.128 | 1.2 | 0.219 | zinc finger proteins ZNF669 or ZNF670 |
| FLJ13222 | 1.01 | 0.011 | 1.00 | 0.092 | 1.205 | 0.246 | 1.251 | 0.003 | testis expressed sequence 27 (TEX27) |
| FLJ20321 | 0.971 | 0.244 | 1.10 | 0.546 | 1.226 | 0.114 | 1.041 | 0.085 | castor homolog 1, zinc finger (Drosophila) (CASZ1) |
| FOG2 | 0.795 | 0.628 | 1.31 | 0.067 | 0.747 | 0.723 | 0.871 | 0.011 | friend of GATA2 |
| FOSB | 1.032 | 0.613 | 1.143 | 0.67 | 0.789 | 0.972 | 1.112 | 0.863 | FBJ murine osteosarcoma viral oncogene homolog B |
| FOSL1 | 1.006 | 0.477 | 1.182 | 0.59 | 0.897 | 0.161 | 1.245 | 0.095 | FOS-like antigen 1 |
| FOXB1 | 1.611 | 0.64 | 1.001 | 0.068 | 0.778 | 0.359 | 0.95 | 0.281 | forkhead box B1 |
| FOXE1 | 0.979 | 0.851 | 1.026 | 0.974 | 1.35 | 0.308 | 1.173 | 0.01 | forkhead box E1 (thyroid transcription factor 2) |
| FOXH1 | 0.947 | 0.411 | 0.775 | 0.362 | 0.948 | 0.342 | 1.017 | 0.001 | forkhead box H1 |
| FOXO3A | 1.593 | 0.415 | 1.054 | 0.783 | 0.932 | 0.359 | 0.747 | 0.978 | forkhead box O3A |
| FOXP1 | 1.261 | 0.649 | 1.699 | 0.029 | 0.822 | 0.951 | 1.156 | 0.001 | forkhead box P1 |
| GCMA | 1.082 | 0.831 | 1.024 | 0.056 | 1.249 | 0.52 | 1.115 | 0.187 | glial cells missing (Drosophila) homolog a |
| GCN5L1 | 1.10 | 0.275 | 1.346 | 0.508 | 1.129 | 0.75 | 1.027 | 0.001 | GCN5 (general control of amino-acid synthesis, yeast, homolog)-like 1 |
| GIOT-2 | 1.205 | 0.765 | 1.117 | 0.213 | 0.738 | 0.114 | 1.204 | 0.051 | GIOT-2 for gonadotropin inducible transcription repressor-2 |
| GLI2 | 1.154 | 0.338 | 0.806 | 0.153 | 0.966 | 0.405 | 0.926 | 0.194 | GLI-Kruppel family member GLI2 |
| GLI3 | 0.813 | 0.081 | 0.97 | 0.368 | 1.138 | 0.249 | 1.125 | 0.232 | GLI-Kruppel family member GLI3 (Greig cephalopolysyndactyly syndrome) |
| GLP | 0.979 | 0.18 | 0.911 | 0.143 | 1.035 | 0.269 | 1.228 | 0.812 | golgin-like protein |
| GSH2 | 1.218 | 0.689 | 1.086 | 0.51 | 1.032 | 0.304 | 0.966 | 0.071 | genomic screened homeo box 2 (mouse) homolog |
| GTF2E2 | 0.714 | 0.342 | 1.212 | 0.437 | 0.806 | 0.26 | 0.884 | 0.704 | general transcription factor IIE, polypeptide 2 (beta subunit, 34kD) |
| GTF2H2 | 1.103 | 0.396 | 0.948 | 0.404 | 0.823 | 0.237 | 1.017 | 0.233 | general transcription factor IIH, polypeptide 2 (44kD subunit) |
| HCNGP | 0.99 | 0.092 | 1.02 | 0.784 | 0.856 | 0.061 | 1.312 | 0.454 | transcriptional regulator protein |
| HDAC2 | 1.181 | 0.793 | 1.009 | 0.142 | 0.83 | 0.274 | 0.922 | 0.001 | histone deacetylase 2 |
| HES7 | 0.899 | 0.012 | 1.439 | 0.752 | 1.056 | 0.681 | 1.215 | 0.001 | hairy and enhancer of split 7 (Drosophila) |
| HEY2 | 0.922 | 0.839 | 0.988 | 0.192 | 1.218 | 0.003 | 1.122 | 0.951 | hairy/enhancer-of-split related with YRPW motif 2 |
| HLX1 | 1.316 | 0.038 | 1.032 | 0.853 | 0.925 | 0.567 | 0.943 | 0.014 | H2.0 (Drosophila)-like homeo box 1 |
| HLXB9 | 1.152 | 0.277 | 0.951 | 0.943 | 0.816 | 0.169 | 0.85 | 0.15 | homeo box HB9 |
| HMGIY | 1.076 | 0.331 | 0.883 | 0.227 | 1.272 | 0.077 | 0.855 | 0.976 | high-mobility group (nonhistone chromosomal) protein isoforms I and Y |
| HMX1 | 1.393 | 0.295 | 0.828 | 0.898 | 1.081 | 0.717 | 0.938 | 0.972 | homeo box (H6 family) 1 |
| HMX2 | 1.202 | 0.098 | 0.917 | 0.242 | 0.97 | 0.128 | 1.00 | 0.408 | homeo box (H6 family) 2 |
| HNF3A | 1.132 | 0.938 | 2.01 | 0.492 | 0.796 | 0.167 | 0.807 | 0.66 | hepatocyte nuclear factor 3, alpha |
| HNF3B | 1.036 | 0.124 | 1.19 | 0.453 | 0.978 | 0.03 | 1.203 | 0.002 | hepatocyte nuclear factor 3, beta |
| HOX11L1 | 0.88 | 0.472 | 1.085 | 0.278 | 1.377 | 0.924 | 1.161 | 0.3 | homeo box 11-like 1 |
| HOXA13 | 1.076 | 0.19 | 1.314 | 0.06 | 0.741 | 0.707 | 1.196 | 0.14 | homeo box A13 |
| HOXA5 | 1.164 | 0.398 | 0.679 | 3.83E-04 | 1.258 | 0.382 | 1.014 | 0.002 | homeo box A5 |
| HOXA6 | 0.999 | 0.142 | 0.849 | 0.324 | 1.201 | 0.445 | 0.783 | 0.059 | homeobox A6 |
| HOXA7 | 1.243 | 0.565 | 0.83 | 0.099 | 0.812 | 0.703 | 0.871 | 0.149 | homeobox A7 |
| HOXB13 | 1.22 | 0.425 | 1.079 | 0.905 | 0.96 | 0.755 | 0.992 | 0.202 | homeo box B13 |
| HOXC5 | 1.295 | 0.592 | 0.928 | 0.973 | 0.78 | 0.333 | 0.82 | 0.53 | homeo box C5 |
| HOXC6 | 0.856 | 0.238 | 1.195 | 0.598 | 1.659 | 0.704 | 1.061 | 0.425 | homeo box C6 |
| HOXD8 | 0.847 | 0.251 | 0.988 | 0.744 | 1.392 | 0.372 | 1.21 | 0.002 | homeobox D8 |
| HRIHFB2122 | 1.057 | 0.145 | 1.226 | 0.065 | 1.174 | 0.801 | 0.888 | 0.316 | TRIO and F-actin binding protein (TRIOBP or TARA) |
| HS747E2A | 0.922 | 0.19 | 0.642 | 0.124 | 1.115 | 0.472 | 0.803 | 0.088 | hypothetical protein (RING domain) |
| HSAJ2425 | 0.978 | 0.372 | 0.921 | 0.689 | 1.202 | 0.231 | 1.139 | 3.89E-04 | p65 protein |
| HSF1 | 1.103 | 0.779 | 1.187 | 0.094 | 0.94 | 0.323 | 0.784 | 0.02 | heat shock transcription factor 1 |
| H_GS165L15 | 1.061 | 0.317 | 1.115 | 0.115 | 0.813 | 0.992 | 0.868 | 0.084 | cAMP response element-binding protein |
| ID1 | 1.399 | 0.137 | 0.852 | 0.766 | 0.98 | 0.903 | 0.758 | 0.214 | inhibitor of DNA binding 1, dominant negative helix-loop-helix protein |
| ILF1 | 0.763 | 0.886 | 1.356 | 0.164 | 0.997 | 0.527 | 1.125 | 0.086 | interleukin enhancer binding factor 1 |
| DNAJ | 1.112 | 0.87 | 1.287 | 0.302 | 0.827 | 0.3 | 0.947 | 0.015 | immune dysregulation, polyendocrinopathy, enteropathy, X-linked |
| IRF2 | 1.106 | 0.371 | 1.289 | 0.79 | 0.861 | 0.613 | 1.035 | 0.034 | interferon regulatory factor 2 |
| JUN | 1.22 | 0.865 | 0.729 | 0.165 | 0.874 | 0.895 | 0.827 | 0.045 | v-jun avian sarcoma virus 17 oncogene homolog |
| JUND | 0.997 | 0.962 | 1.446 | 0.295 | 0.98 | 0.223 | 1.654 | 0.019 | Jun D proto-oncogene |
| KIAA0014 | 1.507 | 0.055 | 0.972 | 0.394 | 0.985 | 0.818 | 0.947 | 0.051 | leucine rich repeat containing 14 (LRRC14) |
| KIAA0040 | 0.643 | 0.701 | 1.021 | 0.224 | 1.166 | 0.17 | 1.207 | 0.006 | KIAA0040 gene product |
| KIAA0161 | 0.766 | 0.795 | 1.15 | 0.727 | 1.064 | 0.869 | 1.203 | 0.259 | likely ortholog of mouse ubiquitin conjugating enzyme 7 interacting protein 4 (UBCE7IP4) |
| KIAA0173 | 1.003 | 0.845 | 1.329 | 0.432 | 0.635 | 0.705 | 1.511 | 3.33E-04 | tubulin tyrosine ligase-like family, member 4 (TTLL4) |
| KIAA0237 | 0.909 | 0.815 | 1.052 | 0.725 | 1.145 | 0.602 | 1.273 | 0.498 | KIAA0237 gene product |
| KIAA0395 | 0.953 | 0.037 | 1.066 | 0.246 | 1.106 | 0.344 | 1.225 | 0.032 | triple homeobox 1 (TIX1) |
| KIAA0414 | 0.886 | 0.267 | 1.002 | 0.002 | 1.202 | 0.178 | 1.241 | 0.011 | zinc finger protein ZNF297B |
| KIAA0478 | 1.167 | 0.677 | 0.996 | 0.077 | 0.732 | 0.47 | 0.897 | 0.029 | zinc finger and BTB domain containing 40 (ZBTB40) |
| KIAA0669 | 1.042 | 0.962 | 1.179 | 0.812 | 0.907 | 0.134 | 1.23 | 0.008 | TSC22 domain family, member 2 (TSC22D2 or TILZ4) |
| KIAA1528 | 0.868 | 0.27 | 1.038 | 0.044 | 1.145 | 0.487 | 1.378 | 2.08E-04 | deltex homolog 2 (Drosophila) (DTX2) |
| KLF5 | 0.507 | 0.97 | 1.363 | 0.364 | 0.856 | 0.26 | 1.033 | 0.325 | Kruppel-like factor 5 |
| KLHL4 | 0.993 | 0.318 | 0.707 | 0.100 | 1.138 | 0.52 | 1.013 | 0.057 | kelch (Drosophila)-like 4 |
| LAF4 | 0.943 | 0.587 | 1.367 | 0.168 | 0.789 | 0.956 | 1.439 | 0.094 | lymphoid nuclear protein related to AF4 |
| LDB2 | 1.124 | 0.168 | 1.172 | 0.711 | 1.091 | 0.85 | 1.274 | 0.559 | LIM domain binding 2 |
| LDOC1 | 0.800 | 0.22 | 1.499 | 0.644 | 0.800 | 0.692 | 1.20 | 0.327 | leucine zipper, down-regulated in cancer 1 |
| LEF1 | 1.04 | 0.11 | 0.928 | 0.189 | 0.918 | 0.996 | 0.828 | 0.843 | lymphoid enhancer factor 1 |
| LIM | 1.055 | 0.934 | 0.637 | 0.624 | 1.098 | 0.792 | 1.159 | 0.023 | LIM protein (similar to rat protein kinase C-binding enigma) |
| LMX1B | 1.316 | 0.357 | 0.923 | 0.117 | 1.068 | 0.485 | 1.068 | 0.23 | LIM homeobox transcription factor 1, beta |
| LOC51036 | 0.976 | 0.059 | 0.819 | 0.007 | 1.431 | 0.296 | 1.067 | 0.026 | retinoic acid receptor-beta associated open reading frame |
| LOC51058 | 1.059 | 0.071 | 0.774 | 0.48 | 0.932 | 0.542 | 1.22 | 0.399 | hypothetical protein |
| LOC51088 | 1.043 | 0.287 | 1.064 | 0.014 | 1.073 | 0.56 | 1.2 | 0.363 | kelch-like 5 (Drosophila) (KLHL5) |
| LOC51131 | 0.811 | 0.242 | 1.013 | 0.012 | 1.178 | 0.991 | 1.302 | 0.001 | putative zinc finger protein NY-REN-34 antigen |
| LOC51193 | 0.999 | 0.493 | 1.014 | 0.79 | 1.20 | 0.502 | 1.381 | 0.109 | zinc finger protein ANC_2H01 |
| LOC51270 | 0.992 | 0.366 | 0.911 | 0.056 | 1.094 | 0.951 | 0.811 | 0.563 | E2F-like protein |
| LOC51290 | 0.943 | 0.407 | 1.11 | 0.24 | 1.11 | 0.467 | 1.207 | 0.224 | CDA14 |
| LOC51637 | 1.176 | 0.922 | 0.946 | 0.584 | 0.932 | 0.483 | 0.807 | 0.012 | chromosome 14 open reading frame 166 |
| LOC55893 | 0.909 | 0.586 | 1.026 | 0.004 | 1.083 | 0.671 | 1.209 | 0.108 | papillomavirus regulatory factor PRF-1 |
| LOC57167 | 0.877 | 0.419 | 0.748 | 0.294 | 1.096 | 0.335 | 1.18 | 0.276 | sal-like 4 (Drosophila) (SALL4) |
| LOC57209 | 0.706 | 0.764 | 0.797 | 0.298 | 1.297 | 0.251 | 1.348 | 0.012 | Kruppel-type zinc finger protein |
| LOC58500 | 0.865 | 0.004 | 1.09 | 0.13 | 1.386 | 0.437 | 1.228 | 0.177 | zinc finger protein (clone 647) |
| LOC65243 | 0.845 | 0.187 | 0.858 | 0.007 | 1.206 | 0.304 | 1.093 | 0.04 | hypothetical protein |
| LOC91120 | 1.288 | 0.714 | 1.019 | 0.374 | 0.808 | 0.338 | 1.181 | 0.006 | similar to ZINC FINGER PROTEIN 85 (ZINC FINGER PROTEIN HPF4) (HTF1) (H. sapiens) |
| LOC92283 | 1.114 | 0.594 | 0.924 | 0.133 | 0.939 | 0.586 | 1.237 | 0.004 | gonadotropin inducible transcription repressor-1 (GIOT-1) |
| M96 | 1.386 | 0.062 | 0.83 | 0.32 | 0.781 | 0.022 | 0.865 | 0.858 | putative DNA binding protein |
| MADH4 | 0.933 | 0.253 | 1.062 | 0.313 | 1.151 | 0.147 | 1.405 | 0.084 | MAD (mothers against decapentaplegic, Drosophila) homolog 4 |
| MADH5 | 0.777 | 0.141 | 1.087 | 0.013 | 0.972 | 0.476 | 0.824 | 0.138 | MAD (mothers against decapentaplegic, Drosophila) homolog 5 |
| MADH7 | 1.542 | 0.334 | 1.431 | 0.127 | 0.661 | 0.861 | 0.961 | 0.241 | MAD (mothers against decapentaplegic, Drosophila) homolog 7 |
| MAFF | 1.028 | 0.01 | 0.555 | 0.05 | 1.026 | 0.067 | 0.558 | 0.463 | v-maf musculoaponeurotic fibrosarcoma (avian) oncogene family, protein F |
| MAPK8IP1 | 0.699 | 0.69 | 0.966 | 0.014 | 0.918 | 0.749 | 1.355 | 0.239 | mitogen-activated protein kinase 8 interacting protein 1 |
| MAX | 1.258 | 0.336 | 1.156 | 0.636 | 0.705 | 0.685 | 1.025 | 0.283 | MAX protein |
| MBLL | 0.917 | 0.795 | 1.039 | 0.437 | 1.043 | 0.053 | 1.243 | 0.298 | C3H-type zinc finger protein; similar to D. melanogaster muscleblind B protein |
| MDS1 | 1.075 | 0.956 | 0.979 | 0.178 | 0.818 | 0.443 | 0.89 | 0.225 | myelodysplasia syndrome 1 |
| MED6 | 0.989 | 0.56 | 0.984 | 0.56 | 1.308 | 0.879 | 1.045 | 0.365 | RNA polymerase II transcriptional regulation mediator (Med6, S. cerevisiae, homolog of) |
| MEF2B | 1.177 | 1.94E-04 | 0.565 | 0.638 | 0.852 | 0.716 | 1.156 | 0.037 | MADS box transcription enhancer factor 2, polypeptide B (myocyte enhancer factor 2B) |
| MEIS3 | 1.086 | 0.011 | 0.878 | 0.855 | 1.059 | 0.205 | 0.806 | 0.076 | meis1-related protein 2 aka MRG2 |
| MGC11349 | 1.044 | 0.883 | 0.803 | 0.831 | 0.989 | 0.617 | 0.978 | 0.153 | hypothetical protein MGC11349 |
| MGC2508 | 1.02 | 0.082 | 0.893 | 0.611 | 1.217 | 0.602 | 1.14 | 0.12 | hypothetical protein MGC2508 |
| MHC2TA | 0.858 | 0.05 | 1.037 | 0.567 | 1.303 | 0.321 | 1.188 | 0.447 | MHC class II transactivator |
| MLLT1 | 1.406 | 0.067 | 0.991 | 0.864 | 0.752 | 0.738 | 0.757 | 0.398 | myeloid/lymphoid or mixed-lineage leukemia (trithorax (Drosophila) homolog); translocated to, 1 |
| MLLT2 | 0.997 | 0.923 | 1.026 | 0.853 | 1.200 | 0.656 | 1.277 | 0.155 | myeloid/lymphoid or mixed-lineage leukemia (trithorax (Drosophila) homolog); translocated to 2 |
| MNDA | 1.061 | 0.684 | 0.802 | 0.525 | 0.995 | 0.807 | 0.915 | 0.795 | myeloid cell nuclear differentiation antigen |
| MORF | 0.992 | 0.873 | 1.091 | 0.007 | 1.22 | 0.114 | 1.253 | 0.051 | histone acetyltransferase |
| MSC | 0.986 | 0.749 | 1.322 | 0.678 | 1.239 | 0.777 | 1.067 | 0.206 | musculin (activated B-cell factor-1) |
| MTF1 | 1.06 | 0.54 | 1.08 | 0.043 | 0.997 | 0.028 | 0.828 | 0.432 | metal-regulatory transcription factor 1 |
| MYC | 0.853 | 0.63 | 1.100 | 0.217 | 1.239 | 0.799 | 1.347 | 0.375 | c-myc proto-oncogene |
| MYCBP | 0.774 | 0.358 | 0.664 | 0.087 | 0.993 | 0.692 | 1.271 | 0.001 | c-myc binding protein |
| MYT2 | 0.917 | 0.501 | 1.406 | 0.044 | 0.685 | 0.041 | 1.176 | 0.287 | myelin transcription factor 2 |
| NCOA1 | 0.928 | 0.239 | 1.035 | 0.053 | 1.174 | 0.849 | 1.254 | 0.084 | nuclear receptor coactivator 1 |
| NFYB | 1.153 | 0.236 | 0.854 | 0.946 | 0.874 | 0.776 | 0.809 | 0.796 | nuclear transcription factor Y, beta |
| NFYC | 0.866 | 0.407 | 1.224 | 0.807 | 1.375 | 0.427 | 1.204 | 0.07 | nuclear transcription factor Y, gamma |
| NKX3A | 0.983 | 0.745 | 0.765 | 0.884 | 0.805 | 0.621 | 0.779 | 0.81 | NK homeobox (Drosophila), family 3, A |
| NMI | 0.843 | 0.558 | 1.066 | 0.224 | 1.212 | 0.325 | 1.203 | 0.55 | N-myc (and STAT) interactor |
| NR1H3 | 0.745 | 0.935 | 1.327 | 0.041 | 1.373 | 0.24 | 0.843 | 0.009 | nuclear receptor subfamily 1, group H, member 3 |
| NR1I3 | 1.068 | 0.586 | 1.258 | 0.035 | 0.924 | 0.817 | 0.968 | 0.077 | nuclear receptor subfamily 1, group I, member 3 |
| NR2E1 | 1.087 | 0.298 | 0.724 | 0.396 | 0.955 | 0.906 | 1.024 | 0.415 | nuclear receptor subfamily 2, group E, member 1 |
| OCT11 | 0.945 | 0.175 | 1.153 | 0.905 | 0.772 | 0.281 | 1.066 | 0.089 | POU domain, class 2, transcription factor 3 (POU2F3) |
| PAF65A | 0.933 | 0.027 | 1.393 | 0.882 | 0.734 | 0.394 | 1.196 | 0.965 | PCAF associated factor 65 alpha |
| PAX1 | 0.821 | 0.978 | 0.956 | 0.792 | 0.922 | 0.184 | 0.934 | 0.452 | paired box gene 1 |
| PBX1 | 0.971 | 0.174 | 0.816 | 0.815 | 1.024 | 0.502 | 0.848 | 0.021 | pre-B-cell leukemia transcription factor 1 |
| PC4 | 0.993 | 0.055 | 1.335 | 0.253 | 1.407 | 0.461 | 1.164 | 0.269 | activated RNA polymerase II transcription cofactor 4 |
| PCAR | 0.864 | 0.345 | 1.043 | 0.729 | 1.285 | 0.234 | 1.228 | 0.28 | hypothetical protein I38022 |
| PER2 | 1.042 | 0.546 | 0.965 | 0.036 | 0.965 | 0.308 | 0.813 | 0.069 | period (Drosophila) homolog 2 |
| PHAP1 | 0.956 | 0.365 | 0.878 | 0.301 | 1.196 | 0.382 | 1.335 | 0.009 | putative human HLA class II associated protein I |
| POU2AF1 | 1.213 | 0.296 | 1.213 | 0.871 | 0.827 | 0.396 | 0.894 | 0.003 | POU domain, class 2, associating factor 1 |
| POU4F1 | 0.879 | 0.34 | 0.662 | 0.347 | 0.903 | 0.581 | 1.181 | 0.84 | POU domain, class 4, transcription factor 1 |
| POU4F2 | 0.767 | 0.76 | 0.823 | 0.693 | 0.871 | 0.442 | 0.693 | 0.027 | POU domain, class 4, transcription factor 2 |
| POU5F1 | 0.905 | 0.926 | 0.893 | 0.955 | 1.241 | 0.756 | 0.957 | 0.18 | POU domain, class 5, transcription factor 1 |
| PRDM13 | 1.133 | 0.002 | 1.061 | 0.41 | 0.803 | 0.514 | 0.812 | 0.006 | PR domain containing 13 |
| PRDM16 | 0.893 | 0.041 | 1.49 | 0.325 | 0.706 | 0.049 | 1.013 | 0.06 | PR domain containing 16 |
| PRDM2 | 1.275 | 0.521 | 0.777 | 0.461 | 0.897 | 0.129 | 0.934 | 0.237 | PR domain containing 2, with ZNF domain |
| PREB | 0.866 | 0.834 | 0.941 | 0.758 | 0.917 | 0.208 | 1.307 | 0.461 | prolactin regulatory element binding |
| PSMC5 | 0.755 | 0.277 | 1.219 | 0.371 | 1.114 | 0.951 | 1.25 | 0.046 | proteasome (prosome, macropain) 26S subunit, ATPase, 5 |
| R28830_2 | 1.284 | 0.657 | 1.071 | 0.719 | 0.963 | 0.279 | 0.897 | 0.048 | similar to ZNF197 (ZNF20) |
| R32184_3 | 0.881 | 0.097 | 0.981 | 0.496 | 1.244 | 0.161 | 1.16 | 0.047 | hypothetical protein MGC4022 |
| RBL2 | 0.964 | 0.203 | 0.829 | 0.382 | 0.883 | 0.173 | 0.824 | 0.998 | retinoblastoma-like 2 (p130) |
| RFP | 1.148 | 0.201 | 0.859 | 0.102 | 1.038 | 0.421 | 1.269 | 0.015 | ret finger protein |
| RFX3 | 0.892 | 0.726 | 1.072 | 0.552 | 0.785 | 0.41 | 1.117 | 0.073 | regulatory factor X, 3 (influences HLA class II expression) |
| RGC32 | 0.725 | 0.891 | 0.994 | 0.019 | 1.063 | 0.119 | 1.343 | 0.001 | RGC32 protein |
| RING1 | 1.094 | 0.076 | 1.473 | 0.168 | 0.971 | 0.438 | 0.893 | 0.9 | ring finger protein 1 |
| RNF22 | 0.743 | 0.494 | 1.02 | 0.932 | 1.204 | 0.782 | 1.09 | 0.443 | ring finger protein 22 |
| RNF3 | 0.958 | 0.06 | 1.091 | 0.902 | 1.22 | 0.894 | 1.168 | 0.838 | ring finger protein 3 |
| RORC | 0.644 | 0.371 | 1.363 | 0.342 | 0.681 | 0.616 | 1.219 | 0.097 | RAR-related orphan receptor C |
| SBB103 | 1.197 | 0.478 | 1.244 | 0.638 | 0.867 | 0.495 | 0.978 | 0.251 | hypothetical SBBI03 protein |
| SETDB1 | 1.074 | 0.573 | 0.818 | 0.869 | 1.134 | 0.388 | 1.138 | 0.171 | SET domain, bifurcated 1 |
| SIAH1 | 0.986 | 0.073 | 0.904 | 0.641 | 1.215 | 0.129 | 0.97 | 0.572 | seven in absentia (Drosophila) homolog 1 |
| SIX4 | 1.21 | 0.99 | 1.133 | 0.167 | 1.09 | 0.277 | 1.109 | 0.027 | sine oculis homeobox (Drosophila) homolog 4 |
| SMARCA2 | 0.828 | 0.93 | 1.04 | 0.558 | 1.026 | 0.729 | 1.201 | 0.036 | SWI/SNF related, matrix associated, actin dependent regulator of chromatin, subfamily a, member 2 |
| SMARCE1 | 1.038 | 0.807 | 0.759 | 0.211 | 1.021 | 0.481 | 1.014 | 0.282 | SWI/SNF related, matrix associated, actin dependent regulator of chromatin, subfamily e, member 1 |
| SNAPC4 | 0.91 | 0.472 | 0.829 | 0.639 | 1.041 | 0.139 | 1.027 | 0.002 | small nuclear RNA activating complex, polypeptide 4, 190kD |
| SOX2 | 1.478 | 0.716 | 0.796 | 0.879 | 1.048 | 0.373 | 0.891 | 0.019 | SRY (sex determining region Y)-box 2 |
| SOX general | 0.541 | 0.105 | 1.041 | 0.33 | 0.89 | 0.692 | 0.645 | 0.014 | sex determining region Y type genes |
| SSX4 | 0.813 | 0.44 | 1.02 | 0.736 | 1.082 | 0.489 | 1.327 | 4.52E-04 | synovial sarcoma, X breakpoint 4 |
| STAT1 | 1.272 | 0.453 | 1.392 | 0.904 | 0.753 | 0.704 | 0.857 | 0.052 | signal transducer and activator of transcription 1, 91kD |
| TAF2B | 1.078 | 0.371 | 1.058 | 0.232 | 1.224 | 0.615 | 1.07 | 0.696 | TATA box binding protein (TBP)-associated factor, RNA polymerase II, B, 150kD |
| TAF2C1 | 1.08 | 0.005 | 1.065 | 0.355 | 0.95 | 0.287 | 0.815 | 0.071 | TATA box binding protein (TBP)-associated factor, RNA polymerase II, C1, 130kD |
| TAF2H | 0.977 | 0.522 | 1.522 | 0.221 | 0.823 | 0.326 | 0.92 | 0.028 | TATA box binding protein (TBP)-associated factor, RNA polymerase II, H, 30kD |
| TAL2 | 0.962 | 0.717 | 1.377 | 0.171 | 1.002 | 0.922 | 0.898 | 0.331 | T-cell acute lymphocytic leukemia 2 |
| TBX15 | 0.807 | 0.762 | 0.847 | 0.164 | 1.309 | 0.268 | 1.448 | 0.01 | T-box 15 |
| TBX2 | 1.082 | 0.673 | 1.202 | 0.699 | 1.100 | 0.55 | 0.963 | 0.453 | T-box 2 |
| TBX21 | 1.089 | 0.411 | 0.786 | 0.382 | 1.032 | 0.729 | 1.037 | 0.255 | T-box 21 |
| TBX5 | 0.758 | 0.757 | 0.974 | 0.055 | 1.217 | 0.367 | 1.192 | 0.017 | T-box 5 (Holt-Oram syndrome) |
| TCF21 | 0.973 | 0.95 | 1.502 | 0.056 | 0.784 | 0.581 | 1.44 | 0.055 | transcription factor 21 |
| TCF8 | 1.237 | 0.041 | 0.937 | 0.882 | 0.885 | 0.682 | 0.826 | 0.352 | transcription factor 8 (represses interleukin 2 expression) |
| TCFL1 | 0.831 | 0.124 | 0.874 | 0.023 | 0.939 | 0.475 | 1.293 | 0.41 | transcription factor-like 1 |
| TCFL4 | 1.35 | 0.182 | 0.947 | 0.333 | 0.766 | 0.15 | 0.889 | 0.812 | represses Txn by recruiting Sin3A-HDAC complex; has bHLH and LeuZip domains |
| TCFL5 | 1.145 | 0.815 | 0.934 | 0.344 | 1.01 | 0.65 | 1.202 | 0.668 | transcription factor-like 5 (basic helix-loop-helix) |
| TEF | 1.102 | 0.042 | 0.903 | 0.44 | 0.962 | 0.386 | 0.828 | 0.663 | thyrotrophic embryonic factor |
| TFE3 | 0.743 | 0.79 | 1.013 | 0.069 | 1.339 | 0.436 | 1.264 | 0.046 | binds to Ig heavy-chain enhancer; has HLH domain |
| TFEB | 1.01 | 0.951 | 1.058 | 0.035 | 1.20 | 0.696 | 1.226 | 0.463 | transcription factor EB |
| TGFB1I1 | 0.78 | 0.703 | 1.232 | 0.92 | 0.935 | 0.461 | 1.124 | 0.149 | transforming growth factor beta 1 induced transcript 1 |
| TIEG2 | 1.322 | 0.704 | 1.247 | 0.366 | 1.011 | 0.083 | 0.914 | 0.085 | TGFB inducible early growth response 2 |
| TITF1 | 0.868 | 0.018 | 1.22 | 0.506 | 0.871 | 0.364 | 1.222 | 0.15 | thyroid transcription factor 1 |
| TNRC6 | 1.026 | 0.137 | 1.006 | 0.686 | 1.239 | 0.804 | 1.217 | 0.46 | trinucleotide repeat containing 6 |
| TNRC9 | 0.883 | 0.082 | 1.073 | 0.029 | 0.997 | 0.772 | 1.256 | 0.035 | trinucleotide repeat containing 9 |
| TONDU | 0.9 | 0.621 | 1.036 | 0.54 | 1.087 | 0.911 | 1.25 | 0.579 | TONDU |
| TRIP15 | 0.96 | 0.087 | 1.313 | 0.041 | 0.955 | 0.898 | 0.975 | 0.011 | thyroid receptor interacting protein 15 |
| VAX2 | 0.914 | 0.101 | 0.945 | 0.046 | 0.856 | 0.184 | 0.822 | 0.45 | homeobox protein VAX2 |
| VENTX2 | 1.11 | 0.782 | 1.299 | 0.007 | 0.807 | 0.181 | 0.907 | 0.244 | haemopoietic progenitor homeobox |
| WHSC1 | 0.96 | 0.203 | 0.998 | 0.39 | 1.264 | 0.228 | 1.178 | 0.166 | Wolf-Hirschhorn syndrome candidate 1 |
| XBP1 | 0.828 | 0.227 | 1.461 | 0.513 | 1.026 | 0.39 | 0.976 | 0.008 | X-box binding protein 1 |
| ZFP36 | 0.895 | 0.13 | 1.048 | 0.866 | 1.067 | 0.175 | 1.271 | 0.764 | zinc finger protein 36, C3H type, homolog (mouse) |
| ZFY | 0.773 | 0.847 | 0.83 | 0.644 | 1.38 | 0.976 | 1.613 | 0.042 | zinc finger protein, Y-linked |
| ZHX1 | 0.84 | 0.307 | 0.976 | 0.985 | 1.295 | 0.139 | 1.278 | 0.035 | zinc-fingers and homeoboxes 1 |
| ZIC2 | 0.923 | 0.195 | 0.945 | 0.523 | 1.315 | 0.381 | 1.371 | 0.256 | zic family member 2 (odd-paired Drosophila homolog, heterotaxy 1) |
| ZIC4 | 0.928 | 0.124 | 1.03 | 0.304 | 1.186 | 0.095 | 1.247 | 0.195 | zinc family member 4 protein HZIC4 |
| ZID | 0.749 | 0.023 | 0.957 | 0.321 | 1.297 | 0.592 | 1.324 | 0.018 | zinc finger protein with interaction domain |
| ZNF135 | 0.997 | 0.412 | 0.966 | 0.328 | 0.968 | 0.318 | 0.798 | 0.028 | zinc finger protein 135 (clone pHZ-17) |
| ZNF144 | 1.304 | 0.624 | 0.989 | 0.198 | 0.885 | 0.27 | 0.908 | 0.212 | zinc finger protein 144 (Mel-18) |
| ZNF15L1 | 0.822 | 0.499 | 1.044 | 0.439 | 1.279 | 0.438 | 1.264 | 0.618 | zinc finger protein 15-like 1 (KOX 8) |
| ZNF165 | 0.884 | 0.669 | 0.914 | 0.652 | 0.886 | 0.224 | 0.827 | 0.244 | zinc finger protein 165 |
| ZNF174 | 0.818 | 0.648 | 1.094 | 0.244 | 0.926 | 0.63 | 0.962 | 0.006 | zinc finger protein 174 |
| ZNF187 | 0.775 | 0.17 | 1.038 | 0.316 | 1.329 | 0.251 | 1.311 | 0.054 | zinc finger protein 187 |
| ZNF205 | 0.986 | 0.778 | 0.726 | 0.302 | 1.045 | 0.518 | 0.897 | 0.007 | zinc finger protein 205 |
| ZNF21 | 0.973 | 0.699 | 0.806 | 0.027 | 1.124 | 0.966 | 1.132 | 0.322 | zinc finger protein 21 (KOX 14) |
| ZNF212 | 0.86 | 0.383 | 0.903 | 0.733 | 1.242 | 0.174 | 1.109 | 0.004 | zinc finger protein 212 |
| ZNF214 | 0.996 | 0.669 | 0.882 | 0.263 | 0.838 | 0.612 | 0.799 | 0.034 | zinc finger protein 214 |
| ZNF230 | 0.989 | 0.01 | 1.000 | 0.971 | 1.419 | 0.659 | 1.028 | 0.894 | zinc finger protein 230 |
| ZNF239 | 0.865 | 0.769 | 1.317 | 0.4 | 1.157 | 0.08 | 1.099 | 0.008 | zinc finger protein (C2H2) homologous to mouse MOK-2 |
| ZNF264 | 0.953 | 0.042 | 0.908 | 0.412 | 0.902 | 0.174 | 0.825 | 0.097 | zinc finger protein 264 |
| ZNF273 | 0.863 | 0.239 | 1.005 | 0.146 | 1.315 | 0.169 | 1.285 | 0.737 | zinc finger protein 273 (HZF9) |
| ZNF29 | 1.144 | 0.226 | 0.956 | 0.017 | 0.792 | 0.522 | 0.801 | 0.232 | zinc finger protein 29 (KOX 26) |
| ZNF295 | 1.162 | 0.262 | 1.015 | 0.7 | 1.035 | 0.871 | 0.826 | 0.237 | zinc finger protein 295 |
| ZNF32 | 0.896 | 0.575 | 0.892 | 0.627 | 1.328 | 0.256 | 1.195 | 0.552 | zinc finger protein 32 (KOX 30) |
| ZNF38 | 0.97 | 0.169 | 1.623 | 0.715 | 1.072 | 0.828 | 0.945 | 0.733 | zinc finger protein 38 (KOX 25) |
| ZNF41 | 0.771 | 0.24 | 0.992 | 0.885 | 1.187 | 0.938 | 1.132 | 0.025 | zinc finger protein 41 |
| ZNF44 | 0.891 | 0.583 | 1.089 | 0.586 | 1.222 | 0.729 | 1.152 | 0.084 | zinc finger protein 44 (KOX 7) |
| ZNF46 | 0.919 | 0.268 | 0.985 | 0.915 | 1.337 | 0.735 | 0.993 | 0.747 | zinc finger protein 46 (KUP) |
| ZNF75A | 0.79 | 0.953 | 1.016 | 0.228 | 1.494 | 0.275 | 1.305 | 1.07E-04 | zinc finger protein 75a |
| ZNF79 | 0.643 | 0.627 | 0.895 | 0.798 | 1.47 | 0.522 | 1.436 | 0.557 | zinc finger protein 79 (pT7) |
| ZNF80 | 1.258 | 0.554 | 1.169 | 0.486 | 0.909 | 0.627 | 0.958 | 0.492 | zinc finger protein 80 (pT17) |
| ZNF90 | 0.906 | 0.016 | 0.989 | 0.291 | 1.116 | 0.745 | 1.211 | 0.246 | zinc finger protein 90 (HTF9) |
| ZNF93 | 0.811 | 0.018 | 0.781 | 0.06 | 1.377 | 0.32 | 1.396 | 0.002 | zinc finger protein 93 (HTF34) |
| ZXDA | 0.922 | 0.002 | 0.965 | 0.03 | 0.934 | 0.86 | 0.824 | 0.013 | zinc finger, X-linked, duplicated A |
