# Supplementary material for: Large Scale Gene Expression Profiles of Regenerating Inner Ear Sensory Epithelia
Source: PLoS One. 2007 Jun 13;2(6):e525. doi: 10.1371/journal.pone.0000525 (PMC1888727; doi:10.1371/journal.pone.0000525)
Supplement: Table S4 — Utricle Laser Differential Expression>1.2-fold and P< = 0.05 (0.05 MB PDF) [file pone.0000525.s005.pdf]

Supplemental Table S4

Gene ID	Entrez	30min		1hr		2hr		3hr			
		**Fold change**	**P-value**	**Fold Change**	**P-value**	**Fold Change**	**P-value**	**Fold Change**	**P-value**	**Updated syml**	**Updated description**
MORF	23522	0.992	0.873	1.091	0.007	1.22	0.114	1.253	0.051	MYST4	MYST histone acetyltransferase (monocytic l
FLJ13222	60685	1.01	0.011	1	0.092	1.205	0.246	1.251	0.003	ZFAND3	Zinc finger, AN1-type domain 3
RFP	5987	1.148	0.201	0.859	0.102	1.038	0.421	1.269	0.015	TRIM27	Tripartite motif-containing 27
LOC92283	92283	1.114	0.594	0.924	0.133	0.939	0.586	1.237	0.004	ZNF461	Zinc finger protein 461
GIOT-2	51710	1.205	0.765	1.117	0.213	0.738	0.114	1.204	0.051	ZNF44	Zinc finger protein 44
KIAA0395	23051	0.953	0.037	1.066	0.246	1.106	0.344	1.225	0.032	ZHX3	Zinc fingers and homeoboxes 3
JUND	3727	0.997	0.962	1.446	0.295	0.98	0.223	1.654	0.019	JUND	jun D proto-oncogene
PHAP1	8125	0.956	0.365	0.878	0.301	1.196	0.382	1.335	0.009	ANP32A	Acidic (leucine-rich) nuclear phosphoprotein
KIAA0173	9654	1.003	0.845	1.329	0.432	0.635	0.705	1.511	3.33E-04	TTLL4	Tubulin tyrosine ligase-like family, member 4
HNF3B	3170	1.036	0.124	1.19	0.453	0.978	0.03	1.203	0.002	FOXA2	Forkhead box A2
KIAA0669	9819	1.042	0.962	1.179	0.812	0.907	0.134	1.23	0.008	TSC22D2	TSC22 domain family, member 2
FLJ11186	55320	0.9	0.136	0.713	0.294	1.491	0.345	1.193	0.007	C14orf106	Chromosome 14 open reading frame 106
HES7	84667	0.899	0.012	1.439	0.752	1.056	0.681	1.215	0.001	HES7	Hairy and enhancer of split 7 (Drosophila)
KIAA0414	KIAA0414	0.886	0.267	1.002	0.002	1.202	0.178	1.241	0.011	KIAA0414	Discontinued
TNRC9	27324	0.883	0.082	1.073	0.029	0.997	0.772	1.256	0.035	TNRC9	Trinucleotide repeat containing 9
BCL11A	53335	0.879	0.959	0.801	0.166	1.251	0.382	1.371	0.005	BCL11A	B-cell CLL/lymphoma 11A (zinc finger prote
KIAA1528	113878	0.868	0.27	1.038	0.044	1.145	0.487	1.378	2.08E-04	DTX2	Deltex homolog 2 (Drosophila)
HOXD8	3234	0.847	0.251	0.988	0.744	1.392	0.372	1.21	0.002	HOXD8	Homeobox D8
ZHX1	11244	0.84	0.307	0.976	0.985	1.295	0.139	1.278	0.035	ZHX1	Zinc fingers and homeoboxes 1
SMARCA2	6595	0.828	0.93	1.04	0.558	1.026	0.729	1.201	0.036	SMARCA2	SWI/SNF related, matrix associated, actin dep
SSX4	10214	0.813	0.44	1.02	0.736	1.082	0.489	1.327	4.52E-04	SSX4	Synovial sarcoma, X breakpoint 3
FLJ10697	55205	0.811	0.11	1.002	0.482	1.122	0.911	1.362	1.60E-04	ZNF532	Zinc finger protein 532
LOC51131	51131	0.811	0.242	1.013	0.012	1.178	0.991	1.302	0.001	PHF11	PHD finger protein 11
TBX15	6913	0.807	0.762	0.847	0.164	1.309	0.268	1.448	0.01	TBX15	T-box 15
ZNF75A	7627	0.79	0.953	1.016	0.228	1.494	0.275	1.305	1.07E-04	ZNF75A	Zinc finger protein 75a
BLZF1	8548	0.777	0.056	1.003	0.038	0.923	0.172	1.215	0.004	BLZF1	Basic leucine zipper nuclear factor 1 (JEM-1)
ZNF187	7741	0.775	0.17	1.038	0.316	1.329	0.251	1.311	0.054	ZNF187	Zinc finger protein 187
MYCBP	26292	0.774	0.358	0.664	0.087	0.993	0.692	1.271	0.001	MYCBP	c-myc binding protein
ZFY	7544	0.773	0.847	0.83	0.644	1.38	0.976	1.613	0.042	ZFY	Zinc finger protein, Y-linked
TBX5	6910	0.758	0.757	0.974	0.055	1.217	0.367	1.192	0.017	TBX5	T-box 5
PSMC5	5705	0.755	0.277	1.219	0.371	1.114	0.951	1.25	0.046	PSMC5	proteasome (prosome, macropain) 26S subuni
TFE3	7030	0.743	0.79	1.013	0.069	1.339	0.436	1.264	0.046	TFE3	Transcription factor binding to IGHM enhanc
RGC32	28984	0.725	0.891	0.994	0.019	1.063	0.119	1.343	0.001	RGC32	Response gene to complement 32
LOC57209	57209	0.706	0.764	0.797	0.298	1.297	0.251	1.348	0.012	ZNF248	Zinc finger protein 248
FHL1	2273	0.681	0.524	1.406	0.467	0.928	0.814	1.26	0.047	FHL1	four and a half LIM domains 1
KIAA0040	9674	0.643	0.701	1.021	0.224	1.166	0.17	1.207	0.006	KIAA0040	KIAA0040 /// Hypothetical protein LOC9674
CEBPG	1054	0.481	0.09	2.27	0.076	0.817	0.209	1.351	0.054	CEBPG	CCAAT/enhancer binding protein (C/EBP), g
ZNF93	81931	0.811	0.018	0.781	0.06	1.377	0.32	1.396	0.002	ZNF93	Zinc finger protein 93
ZID	10773	0.749	0.023	0.957	0.321	1.297	0.592	1.324	0.018	ZBTB6	Zinc finger and BTB domain containing 6
HEY2	23493	0.922	0.839	0.988	0.192	1.218	0.003	1.122	0.951	HEY2	Hairy/enhancer-of-split related with YRPW m
VENTX2	27287	1.11	0.782	1.299	0.007	0.807	0.181	0.907	0.244	VENTX	VENT homeobox homolog (Xenopus laevis)
FOXP1	27086	1.261	0.649	1.699	0.029	0.822	0.951	1.156	0.001	FOXP1	Forkhead box P1
NR1I3	9970	1.068	0.586	1.258	0.035	0.924	0.817	0.968	0.077	NR1I3	Nuclear receptor subfamily 1, group I, membe
TRIP15	9318	0.96	0.087	1.313	0.041	0.955	0.898	0.975	0.011	COPS2	COP9 constitutive photomorphogenic homolo
NR1H3	10062	0.745	0.935	1.327	0.041	1.373	0.24	0.843	0.009	NR1H3	nuclear receptor subfamily 1, group H, memb
ELF3	1999	1.291	0.007	1.045	0.314	0.825	0.595	1.059	0.16	ELF3	E74-like factor 3 (ets domain transcription fac
HLX1	3142	1.316	0.038	1.032	0.853	0.925	0.567	0.943	0.014	HLX1	H2.0-like homeobox 1 (Drosophila)

TCF8	6935	1.237	0.041	0.937	0.882	0.885	0.682	0.826	0.352	TCF8	transcription factor 8 (represses interleukin 2 [illegible]
FLJ12517	116841	1.193	0.052	0.766	0.92	1.012	0.026	1.218	0.177	C1orf142	Chromosome 1 open reading frame 142
HOXA5	3202	1.164	0.398	0.679	3.83E-04	1.258	0.382	1.014	0.002	HOXA5	Homeobox A5
LOC51036	5915	0.976	0.059	0.819	0.007	1.431	0.296	1.067	0.026	RARB	Retinoic acid receptor, beta
ZNF21	7569	0.973	0.699	0.806	0.027	1.124	0.966	1.132	0.322	ZNF182	Zinc finger protein 182 /// zinc finger protein [illegible]
MAFF	23764	1.028	0.01	0.555	0.05	1.026	0.067	0.558	0.463	MAFF	V-maf musculoaponeurotic fibrosarcoma onc[illegible]
MYT2	8827	0.917	0.501	1.406	0.044	0.685	0.041	1.176	0.287	MYT2	Myelin transcription factor 2
M96	22823	1.386	0.062	0.83	0.32	0.781	0.022	0.865	0.858	MTF2	Metal response element binding transcription [illegible]
PRDM16	63976	0.893	0.041	1.49	0.325	0.706	0.049	1.013	0.06	PRDM16	PR domain containing 16
ZXDA	7789	0.922	0.002	0.965	0.03	0.934	0.86	0.824	0.013	ZXDA	Zinc finger, X-linked, duplicated A
HSF1	3297	1.103	0.779	1.187	0.094	0.94	0.323	0.784	0.02	HSF1	Heat shock transcription factor 1
JUN	3725	1.22	0.865	0.729	0.165	0.874	0.895	0.827	0.045	JUN	v-jun sarcoma virus 17 oncogene homolog (a[illegible]
ZNF214	7761	0.996	0.669	0.882	0.263	0.838	0.612	0.799	0.034	ZNF214	Zinc finger protein 214
ZNF135	7694	0.997	0.412	0.966	0.328	0.968	0.318	0.798	0.028	ZNF135	Zinc finger protein 135
PRDM13	59336	1.133	0.002	1.061	0.41	0.803	0.514	0.812	0.006	PRDM13	PR domain containing 13
LOC51637	51637	1.176	0.922	0.946	0.584	0.932	0.483	0.807	0.012	C14orf166	Chromosome 14 open reading frame 166
AF5Q31	27125	0.847	0.425	1.378	0.47	0.752	0.954	0.753	0.008	AFF4	AF4/FMR2 family, member 4
POU4F2	5458	0.767	0.76	0.823	0.693	0.871	0.442	0.693	0.027	POU4F2	POU domain, class 4, transcription factor 2
SOX general	6736	0.541	0.105	1.041	0.33	0.89	0.692	0.645	0.014	SOX general	sex determining region Y type genes

leukemia) 4

32 family, mem
4

ein)

pendent regulato

)

nit, ATPase, 5
cer 3

4
gamma

notif 2

ber 3
og subunit 2 (Ar
ber 3
ictor, epithelial-s

expression)

21
cogene homolog

factor 2

vian)
