# Supplementary material for: Large Scale Gene Expression Profiles of Regenerating Inner Ear Sensory Epithelia
Source: PLoS One. 2007 Jun 13;2(6):e525. doi: 10.1371/journal.pone.0000525 (PMC1888727; doi:10.1371/journal.pone.0000525)
Supplement: Table S5 — Cochlea Neomycin Differentially Expressed Genes (533 total). This listing shows all genes that exhibited>1.2-fold changes in expression, irrespective of P-value. For p-value filtered data see Table S6 (0.71 MB DOC) [file pone.0000525.s006.doc]

Supplemental Table S5 .

|  | **O hr** | | | | **24 hr** | | | | **48 hr** | | | |  | | |  |
| --- | --- | --- | --- | --- | --- | --- | --- | --- | --- | --- | --- | --- | --- | --- | --- | --- |
| **Gene ID** | **Fold change** | | **P-value** | | **Fold Change** | | **P-value** | | **Fold Change** | | **P-value** | | **Notes/Description** | | |  |
| AF5Q31 | | 0.758 | | 0.122 | | 0.259 | | 0.084 | | 0.502 | | 0.057 | | ALL1 fused gene from 5q31 | | |
| AHR | | 1.023 | | 0.335 | | 1.006 | | 0.911 | | 0.691 | | 0.013 | | aryl hydrocarbon receptor | | |
| AIB3 | | 1.063 | | 0.109 | | 0.973 | | 0.490 | | 1.214 | | 0.031 | | nuclear receptor coactivator RAP250 (NCOA6) | | |
| ARIX | | 0.805 | | 0.004 | | 0.785 | | 0.007 | | 0.959 | | 0.669 | | aristaless homeobox | | |
| ARNT | | 1.004 | | 0.918 | | 1.204 | | 0.003 | | 0.776 | | 0.001 | | aryl hydrocarbon receptor nuclear translocator | | |
| ARNTL | | 1.002 | | 0.946 | | 0.980 | | 0.457 | | 1.219 | | 0.003 | | aryl hydrocarbon receptor nuclear translocator-like | | |
| ATBF1 | | 1.142 | | 0.186 | | 0.830 | | 0.020 | | 0.593 | | 0.001 | | AT-binding transcription factor 1 | | |
| ATF2 | | 1.133 | | 0.298 | | 0.923 | | 0.479 | | 0.654 | | 0.023 | | activating transcription factor 2 | | |
| ATF3 | | 1.020 | | 0.654 | | 1.127 | | 0.011 | | 0.607 | | 0.013 | | activating transcription factor 3 | | |
| ATF4 | | 0.983 | | 0.565 | | 0.556 | | 0.020 | | 1.062 | | 0.533 | | activating transcription factor 4 | | |
| ATF5 | | 0.964 | | 0.187 | | 1.090 | | 0.182 | | 0.524 | | 0.011 | | activating transcription factor 5 | | |
| ATF6 | | 1.028 | | 0.554 | | 1.053 | | 0.343 | | 0.814 | | 0.048 | | activating transcription factor 6 | | |
| ATF7 | | 1.243 | | 0.103 | | 0.984 | | 0.824 | | 0.650 | | 0.006 | | activating transcription factor 7 | | |
| BACH1 | | 1.015 | | 0.799 | | 0.861 | | 0.201 | | 0.618 | | 0.005 | | BTB and CNC homology 1, basic leucine zipper transcription factor 1 | | |
| BAPX1 | | 1.446 | | 0.009 | | 1.426 | | 0.103 | | 2.122 | | 0.005 | | bagpipe homeobox (Drosophila) homolog 1 | | |
| BARX2 | | 1.103 | | 0.302 | | 1.021 | | 0.528 | | 0.728 | | 0.005 | | BarH-like homeobox 2 | | |
| BCL11A | | 1.136 | | 0.172 | | 0.952 | | 0.364 | | 1.437 | | 0.009 | | B-cell CLL/lymphoma 11A (zinc finger protein) | | |
| BCL11B | | 0.880 | | 0.037 | | 0.786 | | 0.025 | | 0.985 | | 0.840 | | B-cell CLL/lymphoma 11B (zinc finger protein) | | |
| BCL6 | | 0.997 | | 0.919 | | 1.002 | | 0.958 | | 0.820 | | 0.015 | | B-cell CLL/lymphoma 6 (zinc finger protein 51; Drosophila krüppel homolog) | | |
| BHLHB3 | | 1.124 | | 0.212 | | 1.090 | | 0.095 | | 1.201 | | 0.062 | | basic helix-loop-helix domain containing, class B, 3 | | |
| BLZF1 | | 1.124 | | 0.162 | | 1.075 | | 0.157 | | 1.374 | | 0.034 | | basic leucine zipper nuclear factor 1 (JEM-1) | | |
| BRD1 | | 0.913 | | 0.288 | | 0.579 | | 0.001 | | 0.909 | | 0.270 | | bromodomain-containing 1 | | |
| BRD4 | | 1.570 | | 0.526 | | 0.759 | | 0.077 | | 1.862 | | 0.064 | | bromodomain-containing 4 | | |
| BRD7 | | 1.221 | | 0.010 | | 0.980 | | 0.566 | | 1.374 | | 0.005 | | bromodomain-containing 7 | | |
| BRF2 | | 1.079 | | 0.468 | | 0.938 | | 0.095 | | 0.525 | | 0.045 | | zinc finger protein 36, C3H type-like 2 | | |
| BRPF3 | | 1.060 | | 0.220 | | 0.997 | | 0.949 | | 0.793 | | 0.004 | | bromodomain and PHD finger containing, 3 | | |
| BTEB1 | | 1.245 | | 0.048 | | 0.977 | | 0.588 | | 0.829 | | 0.012 | | basic transcription element binding protein 1 | | |
| BTF3L1 | | 0.835 | | 0.125 | | 0.666 | | 0.003 | | 1.054 | | 0.561 | | basic transcription factor 3, like 1 | | |
| C21orf18 | | 0.750 | | 0.529 | | 0.675 | | 0.001 | | 0.713 | | 0.090 | | chromosome 21 open reading frame 18 | | |
| CART1 | | 1.500 | | 0.013 | | 1.383 | | 0.095 | | 1.002 | | 0.957 | | cartilage paired-class homeoprotein 1 | | |
| CBFA2T3 | | 0.943 | | 0.797 | | 1.274 | | 0.232 | | 1.242 | | 0.470 | | core-binding factor, runt domain, alpha subunit 2; translocated to, 3 | | |
| CBX1 | | 0.977 | | 0.494 | | 1.036 | | 0.754 | | 0.595 | | 0.006 | | chromobox homolog 1 (Drosophila HP1 beta) | | |
| CBX4 | | 0.985 | | 0.634 | | 0.884 | | 0.003 | | 0.716 | | 0.004 | | chromobox homolog 4 (Drosophila Pc class) | | |
| CBX6 | | 1.089 | | 0.043 | | 1.200 | | 8.07E-05 | | 1.047 | | 0.506 | | chromobox homolog 6 | | |
| CDK8 | | 1.114 | | 0.073 | | 1.241 | | 0.005 | | 1.013 | | 0.844 | | Cyclin-dependent kinase 8 | | |
| CEBPB | | 0.988 | | 0.821 | | 1.146 | | 0.234 | | 1.277 | | 0.043 | | CCAAT/enhancer binding protein (C/EBP), beta | | |
| CHD3 | | 1.232 | | 0.048 | | 0.980 | | 0.758 | | 0.762 | | 0.106 | | chromodomain helicase DNA binding protein 3 | | |
| CHD4 | | 0.911 | | 0.224 | | 1.030 | | 0.293 | | 0.582 | | 0.005 | | chromodomain helicase DNA binding protein 4 | | |
| CIAO1 | | 0.622 | | 0.023 | | 0.458 | | 0.003 | | 0.691 | | 0.034 | | WD40 protein Ciao1 | | |
| CITED1 | | 0.617 | | 0.003 | | 0.954 | | 0.263 | | 0.718 | | 0.002 | | Cbp/p300-interacting transactivator, with Glu/Asp-rich carboxy-terminal domain, 1 | | |
| CL469780 | | 0.814 | | 0.007 | | 0.863 | | 0.049 | | 0.822 | | 0.004 | | zinc finger protein ZNF364 | | |
| CREB1 | | 1.055 | | 0.404 | | 0.918 | | 0.175 | | 0.707 | | 0.004 | | cAMP responsive element binding protein-like 1 | | |
| CREBBP | | 0.882 | | 0.258 | | 1.028 | | 0.490 | | 0.822 | | 0.033 | | CREB binding protein (Rubinstein-Taybi syndrome) | | |
| CREG | | 1.138 | | 0.106 | | 0.934 | | 0.086 | | 1.252 | | 0.039 | | cellular repressor of E1A-stimulated genes | | |
| CREM | | 0.976 | | 0.321 | | 1.025 | | 0.802 | | 0.652 | | 0.017 | | cAMP responsive element modulator | | |
| CRSP6 | | 1.210 | | 0.004 | | 0.888 | | 0.334 | | 1.033 | | 0.692 | | cofactor required for Sp1 transcriptional activation, subunit 6 (77kD) | | |
| CRSP8 | | 1.063 | | 0.205 | | 0.901 | | 0.086 | | 1.200 | | 0.077 | | cofactor required for Sp1 transcriptional activation, subunit 8, 34kD | | |
| CSDA | | 1.126 | | 0.227 | | 0.961 | | 0.342 | | 0.680 | | 0.001 | | cold shock domain protein A | | |
| CTCF | | 0.968 | | 0.323 | | 0.951 | | 0.266 | | 0.644 | | 0.003 | | CCCTC-binding factor (zinc finger protein) | | |
| CTNNB1 | | 1.180 | | 0.304 | | 0.760 | | 0.336 | | 1.374 | | 0.001 | | catenin (cadherin-associated protein), beta 1 (88kD) | | |
| CUTL1 | | 1.211 | | 0.014 | | 1.079 | | 0.587 | | 1.007 | | 0.944 | | cut (Drosophila)-like 1 (CCAAT displacement protein) | | |
| DEAF1 | | 0.720 | | 0.004 | | 0.555 | | 0.127 | | 0.976 | | 0.600 | | deformed epidermal autoregulatory factor 1 (Drosophila) | | |
| DFKZP434E026 | | 1.294 | | 0.100 | | 1.758 | | 0.011 | | 1.352 | | 0.021 | | egl nine homolog 2 (C. elegans) (EGLN2) | | |
| DKFZp547H236 | | 1.078 | | 0.166 | | 1.635 | | 0.064 | | 1.272 | | 0.001 | | myeloid ecotropic viral integration site 1 homolog 3 (MEIS3) | | |
| DKFZp762M136 | | 0.972 | | 0.336 | | 0.926 | | 0.241 | | 1.285 | | 0.076 | | fem-1 homolog a (C.elegans) (FEM1A) | | |
| DLX1 | | 0.974 | | 0.326 | | 1.066 | | 0.271 | | 1.258 | | 0.036 | | distal-less homeobox 1 | | |
| DLX3 | | 1.024 | | 0.570 | | 1.074 | | 0.330 | | 0.763 | | 2.32E-04 | | distal-less homeobox 3 | | |
| DLX6 | | 0.978 | | 0.630 | | 0.913 | | 0.041 | | 0.708 | | 0.001 | | distal-less homeobox 6 | | |
| DR1 | | 0.995 | | 0.821 | | 1.109 | | 0.044 | | 1.242 | | 0.033 | | down-regulator of transcription 1, TBP-binding (negative cofactor 2) | | |
| DRIL1 | | 0.901 | | 0.320 | | 1.173 | | 0.312 | | 0.715 | | 0.002 | | dead ringer (Drosophila)-like 1 | | |
| DRPLA | | 0.817 | | 0.054 | | 0.975 | | 0.517 | | 0.881 | | 0.348 | | dentatorubral-pallidoluysian atrophy (atrophin-1) | | |
| DSIPI | | 1.154 | | 0.387 | | 1.106 | | 0.075 | | 0.813 | | 0.003 | | delta sleep inducing peptide, immunoreactor | | |
| E2F3 | | 1.074 | | 0.197 | | 1.124 | | 0.117 | | 0.785 | | 0.002 | | E2F transcription factor 3 | | |
| E2F4 | | 1.128 | | 0.070 | | 1.150 | | 0.009 | | 0.700 | | 0.015 | | E2F transcription factor 4, p107/p130-binding | | |
| E2F6 | | 0.986 | | 0.629 | | 1.128 | | 0.024 | | 0.770 | | 0.014 | | E2F transcription factor 6 | | |
| EBF | | 0.837 | | 0.105 | | 1.119 | | 0.289 | | 0.667 | | 0.001 | | early B-cell factor | | |
| EGR1 | | 1.029 | | 0.531 | | 1.220 | | 0.038 | | 0.845 | | 0.175 | | early growth response 1 | | |
| EGR2 | | 0.981 | | 0.492 | | 0.995 | | 0.937 | | 1.479 | | 0.045 | | early growth response 2 (Krox-20 (Drosophila) homolog) | | |
| EGR3 | | 0.921 | | 0.008 | | 1.124 | | 0.139 | | 0.784 | | 2.51E-04 | | early growth response 3 | | |
| ELF2 | | 0.951 | | 0.048 | | 1.223 | | 0.046 | | 0.802 | | 0.037 | | E74-like factor 2 (ets domain transcription factor) | | |
| ELF3 | | 1.233 | | 2.28E-04 | | 1.128 | | 0.272 | | 1.250 | | 0.004 | | E74-like factor 3 (ets domain transcription factor, epithelial-specific ) |  | |
| ELK4 | | 0.975 | | 0.509 | | 1.056 | | 0.176 | | 1.216 | | 0.057 | | E74-like factor 4 (ets domain transcription factor) | | |
| EMX2 | | 0.916 | | 0.118 | | 0.971 | | 0.308 | | 0.734 | | 0.016 | | empty spiracles (Drosophila) homolog 2 | | |
| EN1 | | 1.053 | | 0.435 | | 0.986 | | 0.757 | | 1.438 | | 0.016 | | engrailed homolog 1 | | |
| EN2 | | 0.916 | | 0.379 | | 0.994 | | 0.901 | | 0.758 | | 4.27E-05 | | engrailed homolog 2 | | |
| EOMES | | 1.142 | | 0.032 | | 1.300 | | 0.001 | | 0.796 | | 0.048 | | eomesodermin (Xenopus laevis) homolog | | |
| EP300 | | 1.013 | | 0.683 | | 1.052 | | 0.207 | | 0.710 | | 0.005 | | E1A binding protein p300 | | |
| EPLIN | | 1.123 | | 0.015 | | 1.047 | | 0.310 | | 1.316 | | 0.039 | | epithelial protein lost in neoplasm beta | | |
| ERCC3 | | 1.045 | | 0.151 | | 1.128 | | 0.004 | | 0.807 | | 0.033 | | excision repair cross-complementing rodent repair deficiency, complementation group 3 | | |
| ERCC6 | | 0.925 | | 0.071 | | 1.293 | | 0.026 | | 0.829 | | 0.006 | | excision repair cross-complementing rodent repair deficiency, complementation group 6 | | |
| ESR1 | | 1.379 | | 0.024 | | 1.499 | | 3.14E-04 | | 1.250 | | 0.177 | | estrogen receptor 1 | | |
| ESRRG | | 1.016 | | 0.629 | | 1.124 | | 0.140 | | 0.817 | | 0.044 | | estrogen-related receptor gamma | | |
| ETS1 | | 0.902 | | 0.662 | | 0.853 | | 0.050 | | 1.219 | | 0.050 | | v-ets avian erythroblastosis virus E26 oncogene homolog 1 | | |
| EYA1 | | 1.035 | | 0.296 | | 1.033 | | 0.435 | | 1.322 | | 0.004 | | eyes absent (Drosophila) homolog 1 | | |
| EZH1 | | 0.915 | | 0.233 | | 1.006 | | 0.909 | | 0.729 | | 0.001 | | enhancer of zeste homolog 1 | | |
| EZH2 | | 0.969 | | 0.397 | | 1.135 | | 0.044 | | 0.781 | | 0.027 | | enhancer of zeste homolog 2 | | |
| FHL1 | | 0.825 | | 0.087 | | 0.802 | | 0.130 | | 0.736 | | 0.009 | | four and a half LIM domains 1 | | |
| FHL2 | | 0.973 | | 0.763 | | 0.868 | | 0.101 | | 0.829 | | 0.003 | | four and a half LIM domains 2 | | |
| FHX | | 1.043 | | 0.375 | | 1.181 | | 0.066 | | 0.770 | | 0.001 | | FOXJ2 forkhead factor | | |
| FKHL18 | | 0.941 | | 0.381 | | 0.810 | | 0.025 | | 0.721 | | 1.51E-05 | | forkhead (Drosophila)-like 18 | | |
| FLI1 | | 0.988 | | 0.667 | | 1.143 | | 0.046 | | 0.722 | | 0.013 | | friend leukemia virus integration 1 |  | |
| FLJ10142 | | 0.845 | | 0.212 | | 0.524 | | 0.007 | | 1.200 | | 0.069 | | likely ortholog of mouse and zebrafish forebrain embryonic zinc finger-like (FEZL) |  | |
| FLJ10697 | | 1.373 | | 0.029 | | 1.141 | | 0.021 | | 1.219 | | 0.018 | | zinc finger protein ZNF532 |  | |
| FLJ10759 | | 1.329 | | 0.009 | | 1.101 | | 0.104 | | 1.232 | | 0.073 | | hypothetical protein FLJ10759 |  | |
| FLJ11186 | | 1.262 | | 0.665 | | 0.730 | | 0.098 | | 1.531 | | 0.053 | | chromosome 14 open reading frame 106 |  | |
| FLJ20321 | | 0.897 | | 0.360 | | 0.957 | | 0.408 | | 0.817 | | 0.002 | | castor homolog 1, zinc finger (Drosophila) (CASZ1) |  | |
| FLJ20557 | | 1.041 | | 0.413 | | 1.064 | | 0.925 | | 0.817 | | 0.109 | | zinc finger protein 416 |  | |
| FLJ20595 | | 0.800 | | 0.017 | | 0.634 | | 0.102 | | 0.825 | | 0.067 | | likely ortholog of mouse zinc finger protein ZFP29 |  | |
| FLJ20729 | | 0.953 | | 0.453 | | 1.137 | | 0.046 | | 1.333 | | 0.009 | | chromosome 1 open reading frame 181 |  | |
| FLJ23309 | | 1.163 | | 0.157 | | 0.997 | | 0.934 | | 1.302 | | 0.023 | | hypothetical protein FLJ23309 |  | |
| FOG2 | | 0.803 | | 0.074 | | 0.589 | | 2.14E-05 | | 0.811 | | 0.341 | | friend of GATA2 |  | |
| FOSL1 | | 1.138 | | 0.167 | | 0.905 | | 0.122 | | 0.817 | | 0.075 | | FOS-like antigen 1 |  | |
| FOXC1 | | 1.034 | | 0.272 | | 1.319 | | 0.001 | | 0.890 | | 0.242 | | forkhead box C1 |  | |
| FOXC2 | | 1.235 | | 0.036 | | 0.955 | | 0.524 | | 1.035 | | 0.583 | | forkhead box C2 (MFH-1, mesenchyme forkhead 1) |  | |
| FOXD1 | | 1.086 | | 0.124 | | 1.056 | | 0.134 | | 1.221 | | 0.028 | | forkhead box D1 |  | |
| FOXE2 | | 1.031 | | 0.663 | | 0.927 | | 0.186 | | 0.789 | | 0.128 | | forkhead box E2 |  | |
| FOXF1 | | 0.974 | | 0.458 | | 0.981 | | 0.765 | | 0.758 | | 0.005 | | forkhead box F1 |  | |
| FOXF2 | | 1.121 | | 0.304 | | 1.224 | | 0.063 | | 0.682 | | 0.013 | | forkhead box F2 |  | |
| FOXH1 | | 0.678 | | 0.003 | | 0.732 | | 0.017 | | 0.835 | | 0.073 | | forkhead box H1 |  | |
| FOXM1 | | 0.992 | | 0.807 | | 0.962 | | 0.320 | | 0.782 | | 0.005 | | forkhead box M1 |  | |
| FOXO3A | | 1.134 | | 1.12E-04 | | 1.243 | | 0.013 | | 0.817 | | 0.004 | | forkhead box O3A |  | |
| FOXP1 | | 0.872 | | 0.346 | | 0.739 | | 3.13E-04 | | 0.950 | | 0.211 | | forkhead box P1 |  | |
| FUBP1 | | 0.989 | | 0.700 | | 1.068 | | 0.227 | | 1.408 | | 0.030 | | far upstream element-binding protein |  | |
| GABPA | | 1.057 | | 0.097 | | 1.090 | | 0.132 | | 0.720 | | 0.007 | | GA-binding protein transcription factor, alpha subunit (60kD) |  | |
| GABPB1 | | 1.081 | | 0.220 | | 1.233 | | 0.002 | | 0.793 | | 0.017 | | GA-binding protein transcription factor, beta subunit 1 (53kD) |  | |
| GAS41 | | 1.078 | | 0.064 | | 1.217 | | 0.035 | | 1.156 | | 0.053 | | glioma-amplified sequence-41 |  | |
| GATA6 | | 1.026 | | 0.617 | | 1.234 | | 4.37E-05 | | 0.898 | | 0.080 | | GATA-binding protein 6 |  | |
| GBX1 | | 1.002 | | 0.974 | | 1.208 | | 0.008 | | 0.848 | | 0.072 | | Gastrulation brain homeobox 1 |  | |
| GBX2 | | 0.457 | | 0.004 | | 0.496 | | 0.003 | | 0.867 | | 0.087 | | Gastrulation brain homeobox 2 |  | |
| GCN5L1 | | 1.037 | | 0.410 | | 0.987 | | 0.868 | | 0.759 | | 2.62E-04 | | GCN5 (general control of amino-acid synthesis, yeast, homolog)-like 1 |  | |
| GCN5L2 | | 0.989 | | 0.582 | | 1.060 | | 0.420 | | 0.721 | | 0.019 | | GCN5 (general control of amino-acid synthesis, yeast, homolog)-like 2 |  | |
| GFI1 | | 1.009 | | 0.842 | | 1.135 | | 0.265 | | 0.781 | | 3.56E-04 | | growth factor independent 1 |  | |
| GFI1B | | 0.907 | | 0.027 | | 1.162 | | 0.074 | | 0.789 | | 5.82E-04 | | growth factor independent 1B (potential regulator of CDKN1A, translocated in CML) |  | |
| GIOT-2 | | 0.850 | | 0.105 | | 0.733 | | 0.022 | | 0.955 | | 0.435 | | GIOT-2 for gonadotropin inducible transcription repressor-2 |  | |
| GLI | | 0.951 | | 0.370 | | 1.395 | | 0.019 | | 0.939 | | 0.604 | | glioma-associated oncogene homolog (zinc finger protein) |  | |
| GLI3 | | 1.072 | | 0.204 | | 1.157 | | 0.090 | | 0.821 | | 0.042 | | GLI-Kruppel family member GLI3 (Greig cephalopolysyndactyly syndrome) |  | |
| GLIS2 | | 1.149 | | 0.651 | | 0.925 | | 0.193 | | 0.819 | | 0.014 | | Kruppel-like zinc finger protein GLIS2 |  | |
| GTF2A1 | | 0.903 | | 0.726 | | 1.429 | | 0.028 | | 1.346 | | 0.109 | | general transcription factor IIA, 1 (37kD and 19kD subunits) |  | |
| GTF2B | | 1.286 | | 0.121 | | 1.060 | | 0.187 | | 0.843 | | 0.056 | | general transcription factor IIB |  | |
| GTF2E1 | | 0.763 | | 0.063 | | 1.055 | | 0.245 | | 0.821 | | 0.008 | | general transcription factor IIE, polypeptide 1 (alpha subunit, 56kD) |  | |
| GTF2E2 | | 0.750 | | 0.012 | | 0.781 | | 0.161 | | 0.837 | | 0.032 | | general transcription factor IIE, polypeptide 2 (beta subunit, 34kD) |  | |
| GTF2F1 | | 0.785 | | 0.018 | | 1.019 | | 0.759 | | 0.586 | | 6.01E-05 | | general transcription factor IIF, polypeptide 1 (74kD subunit) |  | |
| GTF2H2 | | 1.143 | | 0.237 | | 1.386 | | 0.008 | | 0.863 | | 0.166 | | general transcription factor IIH, polypeptide 2 (44kD subunit) |  | |
| GTF2H4 | | 1.149 | | 0.098 | | 1.348 | | 0.047 | | 0.777 | | 2.56E-04 | | general transcription factor IIH, polypeptide 4 (52kD subunit) |  | |
| GTF2I | | 1.026 | | 0.757 | | 0.802 | | 0.015 | | 0.733 | | 0.133 | | general transcription factor II, i |  | |
| GTF3C1 | | 0.874 | | 0.136 | | 1.092 | | 0.121 | | 0.682 | | 0.005 | | general transcription factor IIIC, polypeptide 1 (alpha subunit, 220kD ) |  | |
| GTF3C2 | | 1.003 | | 0.872 | | 1.023 | | 0.628 | | 0.819 | | 0.002 | | general transcription factor IIIC, polypeptide 2 (beta subunit, 110kD) |  | |
| GTF3C3 | | 1.018 | | 0.670 | | 1.132 | | 0.035 | | 0.822 | | 0.106 | | general transcription factor IIIC, polypeptide 3 (102kD) |  | |
| GTF3C4 | | 0.918 | | 0.090 | | 1.152 | | 0.270 | | 0.778 | | 0.010 | | general transcription factor IIIC, polypeptide 4 (90kD) |  | |
| H_GS165L15 | | 0.990 | | 0.843 | | 0.953 | | 0.521 | | 0.790 | | 0.001 | | cAMP response element-binding protein (CRE-Bpa) |  | |
| HAND2 | | 0.943 | | 0.072 | | 1.148 | | 0.004 | | 0.802 | | 0.044 | | basic helix-loop-helix transcription factor HAND2 |  | |
| HCNGP | | 0.651 | | 0.023 | | 0.923 | | 0.144 | | 0.918 | | 0.306 | | transcriptional regulator protein |  | |
| HDAC4 | | 1.137 | | 4.52E-05 | | 1.252 | | 1.63E-04 | | 0.802 | | 7.38E-04 | | histone deacetylase 4 |  | |
| HEY2 | | 1.067 | | 0.212 | | 1.446 | | 0.007 | | 1.062 | | 0.191 | | hairy/enhancer-of-split related with YRPW motif 2 |  | |
| HHEX | | 1.260 | | 0.009 | | 1.370 | | 0.003 | | 0.714 | | 0.005 | | hematopoietically expressed homeobox |  | |
| HIF1A | | 1.013 | | 0.784 | | 1.148 | | 0.073 | | 0.749 | | 0.032 | | hypoxia-inducible factor 1, alpha subunit (basic helix-loop-helix transcription factor) |  | |
| HIRA | | 1.023 | | 0.547 | | 1.164 | | 0.004 | | 0.790 | | 0.019 | | HIR (histone cell cycle regulation defective, S. cerevisiae) homolog A |  | |
| HKR3 | | 0.983 | | 0.795 | | 1.228 | | 0.023 | | 0.819 | | 1.90E-04 | | GLI-Kruppel family member HKR3 |  | |
| H-L(3)MBT | | 0.951 | | 0.273 | | 0.815 | | 0.084 | | 1.233 | | 0.010 | | lethal (3) malignant brain tumor l(3)mbt protein (Drosophila) homolog |  | |
| HLF | | 1.399 | | 0.229 | | 1.046 | | 0.399 | | 1.290 | | 0.049 | | hepatic leukemia factor |  | |
| HLXB9 | | 0.822 | | 0.129 | | 1.108 | | 0.026 | | 0.950 | | 0.171 | | homeo box HB9 |  | |
| HMG2 | | 0.960 | | 0.282 | | 0.715 | | 0.004 | | 1.177 | | 0.039 | | high-mobility group box 2 (HMGB2) |  | |
| HMX1 | | 1.027 | | 0.486 | | 1.250 | | 0.066 | | 1.258 | | 0.019 | | homeo box (H6 family) 1 |  | |
| HNF3A | | 0.901 | | 0.074 | | 1.014 | | 0.818 | | 0.750 | | 0.004 | | hepatocyte nuclear factor 3, alpha |  | |
| HNF3G | | 1.159 | | 0.081 | | 1.156 | | 0.045 | | 0.782 | | 0.017 | | forkhead box A3 (FOXA3) |  | |
| HOXA10 | | 0.988 | | 0.786 | | 1.021 | | 0.712 | | 1.263 | | 0.047 | | homeobox A10 |  | |
| HOXA13 | | 0.918 | | 0.124 | | 0.916 | | 0.936 | | 0.821 | | 0.015 | | homeo box A13 |  | |
| HOXA2 | | 1.086 | | 0.063 | | 1.035 | | 0.410 | | 0.729 | | 0.002 | | homeobox A2 |  | |
| HOXA3 | | 0.750 | | 0.113 | | 0.594 | | 1.94E-04 | | 1.006 | | 0.884 | | homeobox A3 |  | |
| HOXA4 | | 1.200 | | 0.037 | | 1.253 | | 0.051 | | 1.208 | | 0.093 | | homeobox A4 |  | |
| HOXA6 | | 0.952 | | 0.584 | | 1.316 | | 0.110 | | 0.764 | | 0.008 | | homeobox A6 |  | |
| HOXA7 | | 1.099 | | 0.183 | | 1.416 | | 0.001 | | 0.977 | | 0.635 | | homeobox A7 |  | |
| HOXA9 | | 1.128 | | 0.191 | | 0.921 | | 0.907 | | 0.757 | | 0.038 | | homeobox A9 |  | |
| HOXB1 | | 0.907 | | 0.077 | | 1.240 | | 0.093 | | 0.815 | | 0.021 | | homeobox B1 |  | |
| HOXB13 | | 0.907 | | 0.111 | | 1.052 | | 0.305 | | 0.793 | | 0.003 | | homeo box B13 |  | |
| HOXB3 | | 0.995 | | 0.793 | | 0.638 | | 0.010 | | 1.075 | | 0.172 | | homeobox B3 |  | |
| HOXB5 | | 1.018 | | 0.665 | | 1.177 | | 0.144 | | 1.357 | | 0.010 | | homeobox B5 |  | |
| HOXB7 | | 1.119 | | 0.109 | | 1.127 | | 0.401 | | 0.738 | | 0.001 | | homeo box B7 |  | |
| HOXB8 | | 1.265 | | 0.065 | | 1.197 | | 0.013 | | 0.952 | | 0.355 | | homeobox B8 |  | |
| HOXB9 | | 0.934 | | 0.142 | | 1.031 | | 0.306 | | 1.208 | | 0.033 | | homeobox B9 |  | |
| HOXC10 | | 0.980 | | 0.629 | | 1.273 | | 0.016 | | 1.492 | | 0.013 | | homeobox C10 |  | |
| HOXC13 | | 0.983 | | 0.572 | | 1.221 | | 0.033 | | 0.743 | | 0.005 | | homeobox C13 |  | |
| HOXC5 | | 0.923 | | 0.703 | | 1.210 | | 0.131 | | 0.827 | | 0.021 | | homeo box C5 |  | |
| HOXC6 | | 0.914 | | 0.737 | | 1.134 | | 0.140 | | 0.655 | | 0.001 | | homeo box C6 |  | |
| HOXC8 | | 1.001 | | 0.983 | | 1.225 | | 0.001 | | 0.722 | | 0.018 | | homeobox C8 |  | |
| HOXC9 | | 1.021 | | 0.373 | | 1.159 | | 0.046 | | 1.293 | | 0.031 | | homeobox C9 |  | |
| HOXD1 | | 1.092 | | 0.158 | | 1.275 | | 0.057 | | 0.795 | | 0.017 | | homeobox D1 |  | |
| HOXD12 | | 0.913 | | 0.049 | | 1.182 | | 0.003 | | 0.769 | | 0.002 | | homeo box D12 |  | |
| HOXD4 | | 1.097 | | 0.203 | | 1.217 | | 0.011 | | 1.035 | | 0.363 | | homeobox D4 |  | |
| HOXD8 | | 0.896 | | 0.377 | | 1.267 | | 0.027 | | 1.052 | | 0.532 | | homeobox D8 |  | |
| HRIHFB2122 | | 0.932 | | 0.290 | | 0.693 | | 0.008 | | 0.823 | | 0.197 | | TRIO and F-actin binding protein (TRIOBP or TARA) |  | |
| HRIHFB2436 | | 1.113 | | 0.069 | | 0.864 | | 0.062 | | 0.752 | | 0.022 | | endocrine regulator |  | |
| HSA275986 | | 1.145 | | 0.008 | | 1.108 | | 0.109 | | 1.215 | | 0.016 | | transcription factor SMIF |  | |
| HSAJ2425 | | 1.203 | | 0.164 | | 1.520 | | 0.017 | | 1.444 | | 0.024 | | p65 protein |  | |
| HSF2BP | | 1.091 | | 0.361 | | 0.798 | | 0.003 | | 1.000 | | 0.999 | | heat shock transcription factor 2 binding protein |  | |
| HSGT1 | | 1.029 | | 0.615 | | 0.904 | | 0.022 | | 1.232 | | 0.002 | | suppressor of S. cerevisiae gcr2 |  | |
| HSPC018 | | 1.056 | | 0.253 | | 1.325 | | 0.043 | | 1.189 | | 0.005 | | GTP binding protein 1 (GTPBP1) |  | |
| HSPC189 | | 1.018 | | 0.616 | | 1.067 | | 0.619 | | 0.826 | | 0.044 | | zinc finger protein ZNF581 |  | |
| HSPX153 | | 1.002 | | 0.938 | | 0.789 | | 0.012 | | 1.214 | | 0.018 | | HPX-153 homeobox |  | |
| ICBP90 | | 1.236 | | 0.027 | | 1.070 | | 0.082 | | 0.976 | | 0.605 | | ubiquitin-like, containing PHD and RING finger (UHRF1) |  | |
| ICSBP1 | | 1.025 | | 0.293 | | 1.026 | | 0.479 | | 0.766 | | 4.55E-05 | | interferon consensus sequence binding protein 1 |  | |
| ID3 | | 0.944 | | 0.261 | | 1.015 | | 0.721 | | 0.814 | | 0.009 | | inhibitor of DNA binding 3, dominant negative helix-loop-helix protein |  | |
| IGHMBP2 | | 1.000 | | 0.999 | | 1.009 | | 0.722 | | 0.749 | | 4.02E-04 | | immunoglobulin mu binding protein 2 |  | |
| ILF1 | | 1.057 | | 0.393 | | 0.947 | | 0.258 | | 0.672 | | 0.002 | | interleukin enhancer binding factor 1 |  | |
| ILF3 | | 0.919 | | 0.742 | | 1.065 | | 0.340 | | 1.328 | | 0.007 | | interleukin enhancer binding factor 3, 90 kDa |  | |
| DNAJ | | 1.054 | | 0.319 | | 0.933 | | 0.100 | | 0.740 | | 0.001 | | immune dysregulation, polyendocrinopathy, enteropathy, X-linked |  | |
| IRF1 | | 0.942 | | 0.694 | | 0.880 | | 0.461 | | 0.622 | | 0.009 | | interferon regulatory factor 1 |  | |
| IRF2 | | 0.800 | | 0.021 | | 0.754 | | 0.001 | | 0.521 | | 0.010 | | interferon regulatory factor 2 |  | |
| IRF5 | | 0.875 | | 0.028 | | 1.210 | | 0.002 | | 0.806 | | 4.40E-06 | | interferon regulatory factor 5 |  | |
| IRF6 | | 0.989 | | 0.896 | | 1.333 | | 0.095 | | 0.927 | | 0.205 | | interferon regulatory factor 6 |  | |
| IRLB | | 1.275 | | 0.089 | | 1.244 | | 0.001 | | 0.862 | | 0.040 | | c-myc promoter-binding protein |  | |
| IRX4 | | 0.929 | | 0.096 | | 1.040 | | 0.363 | | 0.649 | | 0.003 | | iroquois homeobox protein 4 |  | |
| IRX7 | | 0.901 | | 0.166 | | 0.757 | | 0.010 | | 0.858 | | 0.002 | | iroquois homeobox protein 7 |  | |
| ISGF3G | | 1.051 | | 0.208 | | 1.294 | | 0.083 | | 1.234 | | 0.037 | | interferon-stimulated transcription factor 3, gamma (48kD) |  | |
| JUND | | 0.929 | | 0.580 | | 0.710 | | 0.041 | | 0.734 | | 0.009 | | Jun D proto-oncogene |  | |
| KIAA0026 | | 0.824 | | 0.020 | | 1.024 | | 0.692 | | 1.206 | | 0.012 | | mortality factor 4 like 2 (MORF4L2) |  | |
| KIAA0071 | | 1.023 | | 0.386 | | 0.789 | | 0.010 | | 0.967 | | 0.600 | | REST corepressor (RCOR) |  | |
| KIAA0130 | | 0.710 | | 0.074 | | 0.755 | | 0.031 | | 0.671 | | 0.051 | | thyroid hormone receptor-associated protein, 100 kDa (TRAP100) |  | |
| KIAA0161 | | 1.205 | | 0.114 | | 0.966 | | 0.674 | | 0.838 | | 0.185 | | likely ortholog of mouse ubiquitin conjugating enzyme 7 interacting protein 4 (UBCE7IP4) |  | |
| KIAA0173 | | 0.412 | | 0.002 | | 0.948 | | 0.240 | | 0.786 | | 0.078 | | tubulin tyrosine ligase-like family, member 4 (TTLL4) |  | |
| KIAA0441 | | 0.929 | | 0.663 | | 1.000 | | 0.992 | | 0.792 | | 4.95E-04 | | zinc finger and BTB domain containing 24 (ZBTB24) |  | |
| KIAA0535 | | 0.975 | | 0.364 | | 1.001 | | 0.979 | | 1.210 | | 0.048 | | suppression of tumorigenicity 18 (breast carcinoma) (zinc finger protein) (ST18) |  | |
| KIAA0998 | | 1.026 | | 0.659 | | 1.262 | | 0.019 | | 1.269 | | 0.060 | | tubulin tyrosine ligase-like family, member 5 (TTLL5) |  | |
| KIAA1041 | | 0.767 | | 0.027 | | 0.827 | | 0.299 | | 0.861 | | 0.061 | | forkhead box J3 (FOXJ3) |  | |
| KIAA1442 | | 1.016 | | 0.654 | | 0.954 | | 0.437 | | 1.341 | | 0.004 | | KIAA1442 gene product |  | |
| KLF15 | | 0.997 | | 0.953 | | 0.979 | | 0.650 | | 0.790 | | 0.016 | | Kruppel-like factor 15 |  | |
| KLF5 | | 0.939 | | 0.439 | | 0.832 | | 0.027 | | 0.827 | | 0.021 | | Kruppel-like factor 5 |  | |
| KLHL4 | | 1.010 | | 0.892 | | 1.089 | | 0.247 | | 0.775 | | 0.040 | | kelch (Drosophila)-like 4 |  | |
| LAF4 | | 1.109 | | 0.708 | | 0.594 | | 0.003 | | 0.706 | | 0.025 | | lymphoid nuclear protein related to AF4 |  | |
| LDB1 | | 1.005 | | 0.931 | | 1.083 | | 0.063 | | 0.808 | | 0.023 | | LIM domain binding 1 |  | |
| LDB2 | | 0.966 | | 0.496 | | 0.911 | | 0.003 | | 1.262 | | 0.029 | | LIM domain binding 2 |  | |
| LDOC1 | | 0.939 | | 0.495 | | 0.747 | | 0.021 | | 0.748 | | 0.006 | | leucine zipper, down-regulated in cancer 1 |  | |
| LHX6 | | 1.076 | | 0.248 | | 1.548 | | 0.026 | | 1.278 | | 0.021 | | LIM homeobox protein 6 |  | |
| LHX9 | | 0.980 | | 0.547 | | 1.099 | | 0.107 | | 0.811 | | 0.604 | | LIM homeobox protein 9 |  | |
| LIM | | 1.107 | | 0.006 | | 0.778 | | 0.078 | | 0.806 | | 0.045 | | LIM protein (similar to rat protein kinase C-binding enigma) |  | |
| LMO1 | | 0.696 | | 0.012 | | 0.773 | | 0.010 | | 0.681 | | 0.003 | | LIM domain only 1 (rhombotin 1) |  | |
| LMO2 | | 0.901 | | 0.143 | | 1.038 | | 0.339 | | 1.298 | | 0.038 | | LIM domain only 2 (rhombotin-like 1) |  | |
| LMO6 | | 1.206 | | 0.006 | | 1.048 | | 0.508 | | 0.865 | | 0.011 | | LIM domain only 6 |  | |
| LMX1B | | 0.899 | | 0.514 | | 0.937 | | 0.251 | | 1.313 | | 0.017 | | LIM homeobox transcription factor 1, beta |  | |
| LOC51042 | | 1.007 | | 0.792 | | 0.979 | | 0.513 | | 0.819 | | 0.063 | | zinc finger protein |  | |
| LOC51043 | | 0.978 | | 0.638 | | 0.978 | | 0.485 | | 1.247 | | 0.005 | | zinc finger protein ZFP67 homolog (mouse) |  | |
| LOC51045 | | 0.992 | | 0.766 | | 0.982 | | 0.617 | | 1.201 | | 0.005 | | Kruppel-associated box protein |  | |
| LOC51058 | | 1.258 | | 0.008 | | 1.117 | | 0.314 | | 0.709 | | 0.033 | | hypothetical protein |  | |
| LOC56270 | | 1.054 | | 0.579 | | 1.294 | | 0.049 | | 1.213 | | 0.092 | | WDR45-like (WDR45L) or WIPI49-like protein (WIPI3) |  | |
| LOC57167 | | 0.816 | | 0.014 | | 1.393 | | 0.132 | | 1.125 | | 0.307 | | sal-like 4 (Drosophila) (SALL4) |  | |
| LOC57209 | | 0.868 | | 0.493 | | 1.412 | | 0.002 | | 1.385 | | 0.018 | | Kruppel-type zinc finger protein |  | |
| LOC58500 | | 1.219 | | 0.002 | | 0.947 | | 0.062 | | 1.033 | | 0.483 | | zinc finger protein (clone 647) |  | |
| LOC91120 | | 0.940 | | 0.241 | | 0.825 | | 0.014 | | 0.973 | | 0.699 | | similar to ZINC FINGER PROTEIN 85 (ZINC FINGER PROTEIN HPF4) (HTF1) |  | |
| M96 | | 0.999 | | 0.988 | | 1.235 | | 0.008 | | 0.926 | | 0.342 | | putative DNA binding protein |  | |
| MAD | | 1.236 | | 0.171 | | 0.880 | | 0.230 | | 1.089 | | 0.311 | | MAX dimerization protein 1 |  | |
| MAD4 | | 0.949 | | 0.461 | | 0.826 | | 7.90E-05 | | 1.039 | | 0.659 | | MAX dimerization protein 4 (MXD4) |  | |
| MADH9 | | 1.182 | | 0.244 | | 1.112 | | 0.058 | | 1.256 | | 0.047 | | MAD, mothers against decapentaplegic homolog 9 (Drosophila) |  | |
| MAF | | 0.993 | | 0.774 | | 1.210 | | 0.038 | | 0.857 | | 0.002 | | v-maf musculoaponeurotic fibrosarcoma oncogene homolog (avian) |  | |
| MAPK8IP1 | | 1.082 | | 0.223 | | 0.798 | | 0.004 | | 0.943 | | 0.384 | | mitogen-activated protein kinase 8 interacting protein 1 |  | |
| MAX | | 0.683 | | 0.002 | | 1.042 | | 0.898 | | 1.099 | | 0.041 | | MAX protein |  | |
| MDS1 | | 1.018 | | 0.791 | | 1.565 | | 0.005 | | 1.196 | | 0.072 | | myelodysplasia syndrome 1 |  | |
| MED6 | | 1.342 | | 0.006 | | 1.119 | | 0.586 | | 1.122 | | 0.173 | | RNA polymerase II transcriptional regulation mediator (Med6, S. cerevisiae, homolog of) |  | |
| MEF2B | | 0.468 | | 0.006 | | 0.915 | | 0.449 | | 1.158 | | 0.079 | | MADS box transcription enhancer factor 2, polypeptide B (myocyte enhancer factor 2B) |  | |
| MEOX1 | | 1.007 | | 0.826 | | 1.044 | | 0.177 | | 0.804 | | 0.006 | | mesenchyme homeobox 1 |  | |
| MGC16733 | | 0.932 | | 0.330 | | 0.939 | | 0.151 | | 1.217 | | 0.019 | | integrator complex subunit 4 (INT4) |  | |
| MGC2508 | | 0.994 | | 0.883 | | 0.989 | | 0.870 | | 0.717 | | 0.010 | | hypothetical protein MGC2508 |  | |
| MLLT2 | | 1.210 | | 0.101 | | 0.970 | | 0.671 | | 1.316 | | 0.019 | | myeloid/lymphoid or mixed-lineage leukemia (trithorax (Drosophila) homolog); translocated to 2 |  | |
| MLLT7 | | 1.060 | | 0.213 | | 0.974 | | 0.689 | | 0.774 | | 0.376 | | myeloid/lymphoid or mixed-lineage leukemia (trithorax (Drosophila) homolog); translocated to, 7 |  | |
| MORF | | 0.953 | | 0.476 | | 0.775 | | 0.030 | | 0.963 | | 0.441 | | histone acetyltransferase |  | |
| MSC | | 0.813 | | 0.100 | | 0.773 | | 0.022 | | 1.057 | | 0.398 | | musculin (activated B-cell factor-1) |  | |
| MSX2 | | 1.042 | | 0.174 | | 1.168 | | 0.058 | | 0.750 | | 0.014 | | homeo box, msh-like 2 |  | |
| MTA1 | | 0.747 | | 0.038 | | 0.681 | | 0.005 | | 0.856 | | 0.106 | | metastasis associated 1 |  | |
| MTA1L1 | | 1.177 | | 0.218 | | 1.218 | | 0.002 | | 1.281 | | 0.009 | | metastasis-associated 1-like 1 |  | |
| MYBL2 | | 1.131 | | 0.170 | | 0.844 | | 0.234 | | 0.726 | | 0.004 | | v-myb avian myeloblastosis viral oncogene homolog-like 2 |  | |
| MYCBP | | 1.058 | | 0.115 | | 0.792 | | 0.001 | | 1.266 | | 0.001 | | c-myc binding protein |  | |
| MYF5 | | 0.656 | | 0.017 | | 0.538 | | 0.007 | | 0.802 | | 0.053 | | myogenic factor 5 |  | |
| MYF6 | | 1.208 | | 0.092 | | 1.227 | | 0.075 | | 1.041 | | 0.517 | | myogenic factor 6 (herculin) |  | |
| MYOD1 | | 1.016 | | 0.815 | | 0.838 | | 0.076 | | 1.233 | | 0.001 | | myogenic factor 3 |  | |
| MYT1L | | 0.997 | | 0.968 | | 0.934 | | 0.218 | | 1.213 | | 0.044 | | myelin transcription factor 1-like |  | |
| MYT2 | | 0.856 | | 0.238 | | 0.771 | | 0.005 | | 0.836 | | 0.196 | | myelin transcription factor 2 |  | |
| NAB2 | | 1.039 | | 0.399 | | 1.039 | | 0.374 | | 0.766 | | 0.008 | | NGFI-A binding protein 2 (EGR1 binding protein 2) |  | |
| NCOA3 | | 0.957 | | 0.301 | | 0.878 | | 0.077 | | 0.818 | | 0.021 | | nuclear receptor coactivator 3 |  | |
| NCOR1 | | 0.906 | | 0.688 | | 0.766 | | 0.025 | | 1.327 | | 0.092 | | nuclear receptor co-repressor 1 |  | |
| NEUROD1 | | 0.854 | | 0.024 | | 1.061 | | 0.268 | | 0.774 | | 0.080 | | neurogenic differentiation 1 |  | |
| NEUROD6 | | 0.737 | | 0.025 | | 0.691 | | 1.20E-04 | | 0.736 | | 0.086 | | neurogenic differentiation 6 |  | |
| NEUROG1 | | 1.019 | | 0.647 | | 1.124 | | 0.292 | | 0.689 | | 0.007 | | neurogenin 1 |  | |
| NEUROG2 | | 0.995 | | 0.862 | | 0.813 | | 0.006 | | 1.002 | | 0.974 | | neurogenin 2 |  | |
| NFATC1 | | 0.992 | | 0.873 | | 1.427 | | 0.100 | | 1.662 | | 0.004 | | nuclear factor of activated T-cells, cytoplasmic, calcineurin-dependent 1 |  | |
| NFATC3 | | 1.021 | | 0.406 | | 1.055 | | 0.189 | | 0.778 | | 0.005 | | nuclear factor of activated T-cells, cytoplasmic, calcineurin-dependent 3 |  | |
| NFE2L1 | | 0.704 | | 0.048 | | 1.156 | | 0.315 | | 0.761 | | 0.017 | | nuclear factor (erythroid-derived 2)-like 1 |  | |
| NFE2L2 | | 1.010 | | 0.780 | | 1.071 | | 0.195 | | 0.785 | | 0.003 | | nuclear factor (erythroid-derived 2)-like 2 |  | |
| NFE2L3 | | 0.962 | | 0.319 | | 0.949 | | 0.214 | | 1.313 | | 0.094 | | nuclear factor (erythroid-derived 2)-like 3 |  | |
| NFIB | | 1.034 | | 0.610 | | 1.363 | | 0.027 | | 1.339 | | 0.202 | | nuclear factor I/B |  | |
| NFIC | | 1.123 | | 0.019 | | 0.917 | | 0.258 | | 1.236 | | 0.035 | | nuclear factor I/C (CCAAT-binding transcription factor) |  | |
| NFKB2 | | 1.027 | | 0.741 | | 1.565 | | 0.033 | | 0.970 | | 0.584 | | nuclear factor of kappa light polypeptide gene enhancer in B-cells 2 (p49/p100) |  | |
| NFKBIL1 | | 1.256 | | 0.047 | | 1.079 | | 0.376 | | 0.830 | | 0.007 | | nuclear factor of kappa light polypeptide gene enhancer in B-cells inhibitor-like 1 |  | |
| NFX1 | | 1.040 | | 0.328 | | 0.857 | | 0.373 | | 0.726 | | 0.004 | | nuclear transcription factor, X-box binding 1 |  | |
| NHLH2 | | 1.133 | | 0.256 | | 0.808 | | 0.019 | | 1.430 | | 0.014 | | nescient helix loop helix 2 |  | |
| NKX3A | | 1.075 | | 0.283 | | 0.672 | | 0.135 | | 0.829 | | 0.079 | | NK homeobox (Drosophila), family 3, A |  | |
| NMI | | 1.067 | | 0.119 | | 0.894 | | 0.017 | | 1.272 | | 0.003 | | N-myc (and STAT) interactor |  | |
| NR0B1 | | 1.005 | | 0.913 | | 1.075 | | 0.258 | | 0.777 | | 4.65E-04 | | nuclear receptor subfamily 0, group B, member 1 |  | |
| NR0B2 | | 0.995 | | 0.839 | | 1.694 | | 0.092 | | 1.253 | | 0.031 | | nuclear receptor subfamily 0, group B, member 2 |  | |
| NR1D1 | | 1.033 | | 0.374 | | 1.173 | | 0.014 | | 0.821 | | 0.260 | | nuclear receptor subfamily 1, group D, member 1 |  | |
| NR1H3 | | 1.173 | | 0.090 | | 0.927 | | 0.164 | | 0.772 | | 0.009 | | nuclear receptor subfamily 1, group H, member 3 |  | |
| NR1I3 | | 0.881 | | 0.351 | | 0.691 | | 0.050 | | 0.803 | | 0.001 | | nuclear receptor subfamily 1, group I, member 3 |  | |
| NR2C1 | | 0.993 | | 0.859 | | 1.116 | | 0.409 | | 0.668 | | 0.022 | | nuclear receptor subfamily 2, group C, member 1 |  | |
| NR2E3 | | 1.093 | | 0.556 | | 0.808 | | 0.024 | | 0.995 | | 0.875 | | nuclear receptor subfamily 2, group E, member 3 |  | |
| NR2F1 | | 1.067 | | 0.248 | | 0.869 | | 0.480 | | 1.398 | | 0.001 | | nuclear receptor subfamily 2, group F, member 1 |  | |
| NR2F2 | | 1.053 | | 0.267 | | 0.975 | | 0.802 | | 1.635 | | 0.001 | | nuclear receptor subfamily 2, group F, member 2 |  | |
| NR2F6 | | 1.049 | | 0.350 | | 1.039 | | 0.478 | | 0.792 | | 0.043 | | nuclear receptor subfamily 2, group F, member 6 |  | |
| NR4A1 | | 0.789 | | 0.005 | | 0.855 | | 0.155 | | 0.770 | | 0.021 | | nuclear receptor subfamily 4, group A, member 1 |  | |
| NR4A3 | | 1.159 | | 0.089 | | 1.012 | | 0.830 | | 1.806 | | 0.006 | | nuclear receptor subfamily 4, group A, member 3 |  | |
| NR5A1 | | 0.979 | | 0.258 | | 1.011 | | 0.774 | | 0.814 | | 0.012 | | nuclear receptor subfamily 5, group A, member 1 |  | |
| NR5A2 | | 1.040 | | 0.449 | | 1.120 | | 0.080 | | 1.242 | | 0.050 | | nuclear receptor subfamily 5, group A, member 2 |  | |
| NRIP1 | | 1.019 | | 0.645 | | 1.031 | | 0.443 | | 0.802 | | 4.46E-05 | | nuclear receptor interacting protein 1 |  | |
| NSEP1 | | 1.108 | | 0.080 | | 1.182 | | 0.006 | | 1.560 | | 0.001 | | nuclease sensitive element binding protein 1 |  | |
| ONECUT2 | | 0.839 | | 0.002 | | 0.899 | | 0.009 | | 0.770 | | 0.008 | | one cut domain, family member 2 |  | |
| P38IP | | 1.228 | | 0.008 | | 1.009 | | 0.863 | | 0.850 | | 0.005 | | transcription factor (p38 interacting protein) |  | |
| PAF65A | | 0.603 | | 0.041 | | 0.368 | | 1.48E-05 | | 0.741 | | 0.346 | | PCAF associated factor 65 alpha |  | |
| PAX1 | | 0.719 | | 1.00E-05 | | 0.822 | | 0.085 | | 1.007 | | 0.915 | | paired box gene 1 |  | |
| PAX3 | | 0.913 | | 0.204 | | 1.067 | | 0.180 | | 1.218 | | 0.238 | | paired box gene 3 (Waardenburg syndrome 1) |  | |
| PAX4 | | 0.860 | | 0.001 | | 1.057 | | 0.180 | | 0.807 | | 0.023 | | paired box gene 4 |  | |
| PAX5 | | 1.215 | | 0.026 | | 0.946 | | 0.247 | | 0.727 | | 0.047 | | paired box gene 5 (B-cell lineage specific activator protein) |  | |
| PAX7 | | 1.181 | | 0.001 | | 1.407 | | 0.011 | | 0.879 | | 0.066 | | paired box gene 7 |  | |
| PAX8 | | 1.278 | | 0.035 | | 1.053 | | 0.124 | | 0.818 | | 0.110 | | paired box gene 8 |  | |
| PAX9 | | 1.174 | | 1.05E-04 | | 1.197 | | 0.057 | | 0.790 | | 0.130 | | paired box gene 9 |  | |
| PBX1 | | 1.056 | | 0.178 | | 1.027 | | 0.617 | | 1.257 | | 0.003 | | pre-B-cell leukemia transcription factor 1 |  | |
| PBX3 | | 1.001 | | 0.979 | | 0.953 | | 0.189 | | 1.434 | | 0.001 | | pre-B-cell leukemia transcription factor 3 |  | |
| PC4 | | 1.081 | | 0.552 | | 0.875 | | 0.344 | | 1.356 | | 0.017 | | activated RNA polymerase II transcription cofactor 4 |  | |
| PCAR | | 1.059 | | 0.171 | | 0.876 | | 0.018 | | 1.280 | | 0.018 | | hypothetical protein I38022 |  | |
| PER1 | | 1.118 | | 0.012 | | 1.264 | | 0.040 | | 1.029 | | 0.657 | | period homolog 1 (Drosophila) |  | |
| PER2 | | 1.093 | | 0.123 | | 1.205 | | 0.131 | | 1.247 | | 0.020 | | period homolog 2 (Drosophila) |  | |
| PER3 | | 0.988 | | 0.808 | | 1.168 | | 0.078 | | 0.692 | | 0.025 | | period homolog 3 (Drosophila) |  | |
| PHAP1 | | 1.240 | | 0.067 | | 0.942 | | 0.143 | | 1.040 | | 0.538 | | putative human HLA class II associated protein I |  | |
| PIG7 | | 0.974 | | 0.816 | | 1.584 | | 0.066 | | 1.194 | | 0.290 | | LPS-induced TNF-alpha factor |  | |
| PITX2 | | 1.055 | | 0.285 | | 1.143 | | 0.137 | | 0.820 | | 0.065 | | paired-like homeodomain transcription factor 2 |  | |
| PKNOX1 | | 1.079 | | 0.105 | | 1.026 | | 0.705 | | 1.424 | | 0.007 | | PBX/knotted 1 homeobox 1 |  | |
| PMX1 | | 0.783 | | 0.040 | | 0.629 | | 0.002 | | 0.808 | | 0.164 | | paired mesoderm homeo box 1 |  | |
| POU2AF1 | | 0.941 | | 0.328 | | 0.926 | | 0.193 | | 0.830 | | 0.006 | | POU domain, class 2, associating factor 1 |  | |
| POU2F1 | | 0.943 | | 0.192 | | 1.276 | | 0.078 | | 0.823 | | 0.036 | | POU domain, class 2, transcription factor 1 |  | |
| POU2F2 | | 1.013 | | 0.659 | | 0.758 | | 0.139 | | 1.340 | | 0.012 | | POU domain, class 2, transcription factor 2 |  | |
| POU3F2 | | 0.945 | | 0.469 | | 1.062 | | 0.452 | | 1.441 | | 0.008 | | POU domain, class 3, transcription factor 2 |  | |
| POU3F4 | | 0.964 | | 0.463 | | 1.000 | | 0.991 | | 0.806 | | 0.004 | | POU domain, class 3, transcription factor 4 |  | |
| POU4F1 | | 1.383 | | 0.011 | | 1.033 | | 0.688 | | 1.324 | | 0.089 | | POU domain, class 4, transcription factor 1 |  | |
| POU4F2 | | 0.731 | | 0.011 | | 0.860 | | 0.133 | | 0.648 | | 0.301 | | POU domain, class 4, transcription factor 2 |  | |
| POU4F3 | | 1.029 | | 0.477 | | 1.089 | | 0.397 | | 0.613 | | 0.001 | | POU domain, class 4, transcription factor 3 |  | |
| POU5F1 | | 0.998 | | 0.967 | | 1.088 | | 0.560 | | 0.733 | | 0.002 | | POU domain, class 5, transcription factor 1 |  | |
| POU6F1 | | 0.729 | | 0.240 | | 0.916 | | 0.260 | | 0.786 | | 0.051 | | POU domain, class 6, transcription factor 1 |  | |
| PPARD | | 1.027 | | 0.524 | | 1.217 | | 0.007 | | 0.782 | | 0.005 | | peroxisome proliferative activated receptor, delta |  | |
| PPARG | | 1.061 | | 0.555 | | 1.535 | | 0.023 | | 1.040 | | 0.489 | | peroxisome proliferative activated receptor, gamma |  | |
| PPARGC1 | | 0.956 | | 0.367 | | 0.882 | | 0.349 | | 0.763 | | 0.213 | | peroxisome proliferative activated receptor, gamma, coactivator 1 |  | |
| PRDM10 | | 0.978 | | 0.658 | | 0.812 | | 0.062 | | 0.898 | | 0.055 | | PR domain containing 10 |  | |
| PRDM12 | | 1.009 | | 0.890 | | 1.036 | | 0.241 | | 0.809 | | 0.013 | | PR domain containing 12 |  | |
| PRDM13 | | 0.549 | | 0.019 | | 0.400 | | 0.013 | | 0.665 | | 0.104 | | PR domain containing 13 |  | |
| PRDM15 | | 0.926 | | 0.556 | | 0.830 | | 0.327 | | 0.680 | | 4.77E-04 | | PR domain containing 15 |  | |
| PRDM2 | | 1.047 | | 0.247 | | 1.351 | | 0.038 | | 0.859 | | 0.028 | | PR domain containing 2, with ZNF domain |  | |
| PRDM9 | | 1.090 | | 0.279 | | 1.069 | | 0.237 | | 1.249 | | 0.002 | | PR domain containing 9 |  | |
| PREB | | 0.542 | | 0.009 | | 1.090 | | 0.638 | | 0.819 | | 0.091 | | prolactin regulatory element binding |  | |
| PROP1 | | 0.991 | | 0.782 | | 1.047 | | 0.352 | | 1.271 | | 0.054 | | prophet of Pit1, paired-like homeodomain transcription factor |  | |
| PROX1 | | 1.020 | | 0.640 | | 0.912 | | 0.691 | | 0.784 | | 0.002 | | prospero-related homeobox 1 |  | |
| PTTG1IP | | 1.401 | | 0.040 | | 1.495 | | 0.001 | | 1.013 | | 0.864 | | pituitary tumor-transforming 1 interacting protein |  | |
| PURA | | 0.977 | | 0.658 | | 0.792 | | 0.044 | | 0.908 | | 0.185 | | purine-rich element binding protein A |  | |
| RARA | | 0.913 | | 0.334 | | 0.762 | | 0.044 | | 0.984 | | 0.560 | | retinoic acid receptor, alpha |  | |
| RARG | | 0.948 | | 0.465 | | 0.931 | | 0.254 | | 1.308 | | 0.013 | | retinoic acid receptor, gamma |  | |
| RBBP9 | | 1.067 | | 0.319 | | 1.312 | | 0.013 | | 0.770 | | 0.402 | | retinoblastoma binding protein 9 |  | |
| RBL1 | | 0.983 | | 0.665 | | 1.017 | | 0.598 | | 0.819 | | 0.001 | | retinoblastoma-like 1 (p107) |  | |
| RBL2 | | 1.057 | | 0.413 | | 1.088 | | 0.109 | | 0.678 | | 0.040 | | retinoblastoma-like 2 (p130) |  | |
| RBPSUHL | | 0.846 | | 0.080 | | 0.939 | | 0.189 | | 0.678 | | 0.008 | | recombining binding protein suppressor of hairless-like (Drosophila) |  | |
| REL | | 1.015 | | 0.706 | | 1.137 | | 0.581 | | 1.286 | | 0.056 | | v-rel reticuloendotheliosis viral oncogene homolog (avian) |  | |
| RELB | | 1.040 | | 0.341 | | 1.203 | | 0.015 | | 0.760 | | 0.015 | | v-rel reticuloendotheliosis viral oncogene homolog B, nuclear factor of kappa light polypeptide gene enhancer in B-cells 3 (avian) |  | |
| RFP2 | | 1.008 | | 0.776 | | 1.094 | | 0.099 | | 1.371 | | 0.001 | | ret finger protein 2 |  | |
| RFXANK | | 0.863 | | 0.085 | | 1.141 | | 0.770 | | 0.733 | | 0.001 | | regulatory factor X-associated ankyrin-containing protein |  | |
| RING1 | | 1.372 | | 0.164 | | 0.944 | | 0.626 | | 1.113 | | 0.054 | | ring finger protein 1 |  | |
| RNF10 | | 1.154 | | 0.050 | | 0.951 | | 0.280 | | 1.261 | | 0.005 | | ring finger protein 10 |  | |
| RNF13 | | 1.002 | | 0.961 | | 0.807 | | 0.002 | | 1.100 | | 0.421 | | ring finger protein 13 |  | |
| RNF14 | | 0.907 | | 0.413 | | 0.785 | | 0.031 | | 1.015 | | 0.835 | | ring finger protein 14 |  | |
| RNF22 | | 1.204 | | 0.020 | | 0.954 | | 0.090 | | 1.226 | | 0.001 | | ring finger protein 22 |  | |
| RNF24 | | 1.072 | | 0.166 | | 1.029 | | 0.736 | | 0.718 | | 0.009 | | ring finger protein 24 |  | |
| RNF4 | | 0.666 | | 0.001 | | 0.654 | | 0.023 | | 0.984 | | 0.936 | | ring finger protein 4 |  | |
| RORA | | 0.991 | | 0.832 | | 0.894 | | 0.141 | | 1.255 | | 0.014 | | RAR-related orphan receptor A |  | |
| RORC | | 1.042 | | 0.768 | | 0.726 | | 0.031 | | 0.603 | | 0.002 | | RAR-related orphan receptor C |  | |
| RPF-1 | | 1.009 | | 0.880 | | 0.860 | | 0.039 | | 1.230 | | 0.033 | | retina-derived POU-domain factor-1 |  | |
| RRN3 | | 1.125 | | 0.353 | | 0.865 | | 0.028 | | 1.273 | | 0.043 | | RNA polymerase I transcription factor RRN3 |  | |
| RUNX2 | | 1.049 | | 0.284 | | 0.911 | | 0.106 | | 1.217 | | 0.012 | | runt-related transcription factor 2 |  | |
| RXRB | | 1.270 | | 0.006 | | 0.970 | | 0.543 | | 0.705 | | 0.015 | | retinoid X receptor, beta |  | |
| SAFB | | 1.142 | | 0.196 | | 1.408 | | 0.001 | | 1.043 | | 0.582 | | scaffold attachment factor B |  | |
| SAP18 | | 1.190 | | 0.189 | | 1.001 | | 0.987 | | 1.310 | | 0.099 | | sin3-associated polypeptide, 18kD |  | |
| SATB1 | | 1.021 | | 0.624 | | 0.852 | | 0.155 | | 0.805 | | 0.010 | | special AT-rich sequence binding protein 1 (binds to nuclear matrix/scaffold-associating DNAs) |  | |
| SBB103 | | 1.166 | | 0.063 | | 0.810 | | 0.073 | | 0.802 | | 0.340 | | hypothetical SBBI03 protein |  | |
| SCML2 | | 1.106 | | 0.063 | | 1.137 | | 0.316 | | 1.309 | | 0.040 | | sex comb on midleg-like 2 (Drosophila) |  | |
| SDCCAG33 | | 0.827 | | 0.051 | | 0.816 | | 0.001 | | 0.930 | | 0.176 | | serologically defined colon cancer antigen 33 |  | |
| SETDB1 | | 0.953 | | 0.532 | | 1.330 | | 0.004 | | 0.975 | | 0.570 | | SET domain, bifurcated 1 |  | |
| SHOX | | 0.883 | | 0.001 | | 1.043 | | 0.376 | | 0.680 | | 0.001 | | short stature homeobox |  | |
| SIM2 | | 0.983 | | 0.669 | | 0.985 | | 0.701 | | 0.817 | | 0.022 | | single-minded homolog 2 (Drosophila) |  | |
| SIX1 | | 0.991 | | 0.766 | | 0.924 | | 0.434 | | 0.748 | | 2.14E-06 | | sine oculis homeobox homolog 1 (Drosophila) |  | |
| SIX2 | | 0.901 | | 0.002 | | 0.957 | | 0.254 | | 0.670 | | 3.94E-04 | | sine oculis homeobox homolog 2 (Drosophila) |  | |
| SIX3 | | 0.997 | | 0.949 | | 0.832 | | 0.025 | | 0.651 | | 0.002 | | sine oculis homeobox (Drosophila) homolog 3 |  | |
| SIX4 | | 0.621 | | 0.009 | | 0.681 | | 4.65E-04 | | 0.624 | | 0.002 | | sine oculis homeobox (Drosophila) homolog 4 |  | |
| SLB | | 1.054 | | 0.226 | | 1.168 | | 0.203 | | 1.284 | | 0.016 | | selective LIM binding factor |  | |
| SMARCC1 | | 1.219 | | 0.097 | | 1.185 | | 0.150 | | 1.131 | | 0.453 | | SWI/SNF related, matrix associated, actin dependent regulator of chromatin, subfamily c, member 1 |  | |
| SNAPC3 | | 1.021 | | 0.601 | | 1.109 | | 0.388 | | 0.521 | | 0.003 | | small nuclear RNA activating complex, polypeptide 3, 50kDa |  | |
| SOX14 | | 1.207 | | 0.089 | | 0.951 | | 0.587 | | 0.737 | | 0.055 | | SWI/SNF related, matrix associated, actin dependent regulator of chromatin, subfamily b, member 1 |  | |
| SOX2 | | 1.023 | | 0.501 | | 0.580 | | 0.013 | | 1.016 | | 0.773 | | SRY (sex determining region Y)-box 2 |  | |
| SPI1 | | 1.056 | | 0.460 | | 1.001 | | 0.989 | | 0.717 | | 1.43E-04 | | spleen focus forming virus (SFFV) proviral integration oncogene spi1 |  | |
| SPIB | | 1.107 | | 0.326 | | 1.006 | | 0.859 | | 0.809 | | 0.007 | | Spi-B transcription factor (Spi-1/PU.1 related) |  | |
| SRA1 | | 1.422 | | 0.134 | | 0.886 | | 4.29E-05 | | 1.333 | | 0.002 | | steroid receptor RNA activator 1 |  | |
| SREBF2 | | 0.835 | | 0.228 | | 1.033 | | 0.598 | | 0.703 | | 0.004 | | sterol regulatory element binding transcription factor 2 |  | |
| SRF | | 0.947 | | 0.212 | | 0.900 | | 0.037 | | 0.723 | | 0.011 | | serum response factor (c-fos serum response element-binding transcription factor) |  | |
| SSX1 | | 1.120 | | 0.204 | | 1.250 | | 0.020 | | 1.125 | | 0.432 | | synovial sarcoma, X breakpoint 1 |  | |
| SSX2 | | 1.137 | | 0.131 | | 1.546 | | 0.044 | | 1.157 | | 0.105 | | synovial sarcoma, X breakpoint 2 |  | |
| SSX3 | | 1.059 | | 0.396 | | 0.799 | | 0.063 | | 1.295 | | 0.022 | | synovial sarcoma, X breakpoint 3 |  | |
| STAT6 | | 0.995 | | 0.769 | | 0.971 | | 0.633 | | 0.772 | | 0.019 | | signal transducer and activator of transcription 6, interleukin-4 induced |  | |
| SUPT3H | | 0.991 | | 0.844 | | 0.988 | | 0.511 | | 0.784 | | 0.004 | | suppressor of Ty 3 homolog (S.cerevisiae) |  | |
| SUPT4H1 | | 0.920 | | 0.067 | | 0.936 | | 0.547 | | 0.614 | | 0.047 | | suppressor of Ty (S.cerevisiae) 4 homolog |  | |
| SURB7 | | 1.006 | | 0.798 | | 1.033 | | 0.415 | | 0.654 | | 0.021 | | SRB7 suppressor of RNA polymerase B homolog (yeast) |  | |
| TADA3L | | 1.222 | | 0.034 | | 0.807 | | 0.021 | | 0.924 | | 0.370 | | transcriptional adaptor 3 (ADA3 homolog, yeast)-like |  | |
| TAF-172 | | 0.745 | | 0.005 | | 0.838 | | 0.120 | | 0.846 | | 0.116 | | TBP-associated factor 172 |  | |
| TAF1A | | 1.106 | | 0.041 | | 1.124 | | 0.161 | | 1.200 | | 0.064 | | TATA box binding protein (TBP)-associated factor, RNA polymerase I, A, 48kDa |  | |
| TAF1C | | 1.044 | | 0.390 | | 1.025 | | 0.655 | | 1.260 | | 0.038 | | TATA box binding protein (TBP)-associated factor, RNA polymerase I, C, 110kD |  | |
| TAF2C1 | | 0.945 | | 0.169 | | 1.001 | | 0.986 | | 0.791 | | 0.005 | | TATA box binding protein (TBP)-associated factor, RNA polymerase II, C1, 130kD |  | |
| TAF2C2 | | 1.048 | | 0.422 | | 1.018 | | 0.727 | | 0.701 | | 0.027 | | TAF4b RNA polymerase II, TATA box binding protein (TBP)-associated factor, 105kDa |  | |
| TAF2D | | 0.929 | | 0.347 | | 1.245 | | 0.043 | | 1.492 | | 0.048 | | TAF5 RNA polymerase II, TATA box binding protein (TBP)-associated factor, 100kDa |  | |
| TAF2H | | 0.774 | | 0.190 | | 0.652 | | 4.26E-04 | | 0.641 | | 0.003 | | TATA box binding protein (TBP)-associated factor, RNA polymerase II, H, 30kD |  | |
| TAF2N | | 0.812 | | 0.018 | | 0.937 | | 0.319 | | 0.846 | | 0.040 | | TAF15 RNA polymerase II, TATA box binding protein (TBP)-associated factor, 68kDa |  | |
| TAL2 | | 0.771 | | 4.09E-04 | | 0.891 | | 0.267 | | 1.019 | | 0.823 | | T-cell acute lymphocytic leukemia 2 |  | |
| TBP | | 1.159 | | 0.279 | | 0.994 | | 0.918 | | 0.685 | | 0.003 | | TATA box binding protein |  | |
| TBPL1 | | 1.026 | | 0.618 | | 1.011 | | 0.759 | | 1.234 | | 0.062 | | TBP-like 1 |  | |
| TBR1 | | 1.050 | | 0.499 | | 1.174 | | 0.052 | | 1.339 | | 0.050 | | T-box, brain, 1 |  | |
| TBX10 | | 1.067 | | 0.761 | | 1.076 | | 0.212 | | 0.790 | | 1.56E-05 | | T-box 10 |  | |
| TBX15 | | 1.115 | | 0.071 | | 1.088 | | 0.198 | | 1.218 | | 0.004 | | T-box 15 |  | |
| TBX21 | | 1.170 | | 0.172 | | 1.400 | | 0.022 | | 1.044 | | 0.508 | | T-box 21 |  | |
| TBX3 | | 1.052 | | 0.727 | | 1.044 | | 0.251 | | 1.275 | | 0.013 | | T-box 3 (ulnar mammary syndrome) |  | |
| TBX5 | | 1.158 | | 0.056 | | 0.976 | | 0.756 | | 1.252 | | 0.005 | | T-box 5 (Holt-Oram syndrome) |  | |
| TBX6 | | 1.099 | | 0.248 | | 1.000 | | 0.988 | | 0.785 | | 0.012 | | T-box 6 |  | |
| TCEAL1 | | 1.072 | | 0.273 | | 1.306 | | 0.014 | | 0.854 | | 0.025 | | transcription elongation factor A (SII)-like 1 |  | |
| TCF12 | | 0.890 | | 0.223 | | 1.021 | | 0.491 | | 1.229 | | 0.019 | | transcription factor 12 (HTF4, helix-loop-helix transcription factors 4) |  | |
| TCF3 | | 1.015 | | 0.718 | | 1.015 | | 0.779 | | 0.758 | | 3.60E-04 | | transcription factor 3 (E2A immunoglobulin enhancer binding factors E12/E47) |  | |
| TCF8 | | 0.804 | | 0.064 | | 1.000 | | 0.998 | | 1.279 | | 0.031 | | transcription factor 8 (represses interleukin 2 expression) |  | |
| TCFL5 | | 0.806 | | 0.052 | | 1.761 | | 0.052 | | 1.060 | | 0.240 | | transcription factor-like 5 (basic helix-loop-helix) |  | |
| TEAD1 | | 0.729 | | 0.187 | | 1.010 | | 0.908 | | 1.083 | | 0.424 | | TEA domain family member 1 (SV40 transcriptional enhancer factor) |  | |
| TEAD3 | | 0.823 | | 0.014 | | 1.007 | | 0.882 | | 0.916 | | 0.047 | | TEA domain family member 3 |  | |
| TEAD4 | | 0.903 | | 0.021 | | 1.132 | | 0.619 | | 1.288 | | 0.106 | | TEA domain family member 4 |  | |
| TEF | | 0.902 | | 0.247 | | 1.226 | | 0.039 | | 0.717 | | 0.022 | | thyrotrophic embryonic factor |  | |
| TFAP2A | | 0.897 | | 0.298 | | 0.944 | | 0.119 | | 0.808 | | 0.015 | | transcription factor AP-2 alpha (activating enhancer binding protein 2 alpha) |  | |
| TFAP4 | | 0.896 | | 0.027 | | 0.940 | | 0.234 | | 0.725 | | 0.003 | | transcription factor AP-4 (activating enhancer binding protein 4) |  | |
| TFCP2 | | 0.970 | | 0.758 | | 0.784 | | 0.006 | | 1.047 | | 0.438 | | transcription factor CP2 |  | |
| THRA | | 0.827 | | 0.017 | | 1.039 | | 0.763 | | 1.045 | | 0.539 | | thyroid hormone receptor, alpha (erythroblastic leukemia viral (v-erb-a) oncogene homolog, avian) |  | |
| TIEG2 | | 0.990 | | 0.842 | | 0.818 | | 0.121 | | 0.820 | | 0.385 | | TGFB inducible early growth response 2 |  | |
| TIMELESS | | 1.300 | | 0.034 | | 1.378 | | 0.006 | | 1.358 | | 0.047 | | timeless homolog (Drosophila) |  | |
| TITF1 | | 0.975 | | 0.832 | | 0.821 | | 0.004 | | 0.923 | | 0.156 | | thyroid transcription factor 1 |  | |
| TMF1 | | 0.956 | | 0.231 | | 1.033 | | 0.454 | | 1.455 | | 0.023 | | TATA element modulatory factor 1 |  | |
| TNRC3 | | 0.984 | | 0.693 | | 0.783 | | 0.009 | | 1.009 | | 0.819 | | trinucleotide repeat containing 3 |  | |
| TNRC4 | | 1.104 | | 0.079 | | 1.007 | | 0.884 | | 1.277 | | 1.47E-04 | | trinucleotide repeat containing 4 |  | |
| TNRC5 | | 0.623 | | 0.026 | | 0.454 | | 0.005 | | 1.000 | | 0.997 | | trinucleotide repeat containing 5 |  | |
| TNRC6 | | 1.168 | | 0.020 | | 0.917 | | 0.039 | | 1.257 | | 0.008 | | trinucleotide repeat containing 6 |  | |
| TRAP150 | | 0.997 | | 0.918 | | 0.929 | | 0.260 | | 1.219 | | 0.004 | | thyroid hormone receptor-associated protein, 150 kDa subunit |  | |
| TRIM15 | | 0.795 | | 0.036 | | 0.708 | | 0.001 | | 0.965 | | 0.497 | | tripartite motif-containing 15 |  | |
| TRIM22 | | 1.035 | | 0.640 | | 1.009 | | 0.854 | | 1.234 | | 0.021 | | tripartite motif-containing 22 |  | |
| TRIP11 | | 1.018 | | 0.653 | | 0.906 | | 0.152 | | 1.250 | | 0.011 | | thyroid hormone receptor interactor 11 |  | |
| TRIP15 | | 0.682 | | 0.003 | | 1.131 | | 0.101 | | 0.962 | | 0.455 | | thyroid receptor interacting protein 15 |  | |
| TRIP6 | | 0.955 | | 0.281 | | 1.608 | | 0.062 | | 1.617 | | 0.034 | | thyroid hormone receptor interactor 6 |  | |
| TZFP | | 1.083 | | 0.260 | | 1.062 | | 0.324 | | 1.610 | | 0.044 | | testis zinc finger protein |  | |
| UTF1 | | 1.246 | | 0.093 | | 0.869 | | 0.332 | | 1.350 | | 0.015 | | undifferentiated embryonic cell transcription factor 1 |  | |
| VSX1 | | 1.012 | | 0.791 | | 1.047 | | 0.287 | | 0.816 | | 0.330 | | visual system homeobox 1 homolog, CHX10-like (zebrafish) |  | |
| WHN | | 0.979 | | 0.512 | | 1.017 | | 0.716 | | 1.551 | | 0.002 | | winged-helix nude |  | |
| WHSC1 | | 1.275 | | 0.026 | | 1.060 | | 0.230 | | 1.098 | | 0.075 | | Wolf-Hirschhorn syndrome candidate 1 |  | |
| YAF2 | | 1.038 | | 0.547 | | 1.046 | | 0.452 | | 1.476 | | 0.033 | | YY1 associated factor 2 |  | |
| YY1 | | 0.866 | | 0.009 | | 0.968 | | 0.625 | | 1.603 | | 0.013 | | YY1 transcription factor |  | |
| ZF5128 | | 0.921 | | 0.861 | | 0.873 | | 0.005 | | 1.499 | | 0.018 | | Zinc finger protein |  | |
| ZFHX1B | | 0.903 | | 0.022 | | 1.230 | | 0.083 | | 1.344 | | 0.034 | | zinc finger homeobox 1B |  | |
| ZFP103 | | 0.959 | | 0.363 | | 0.930 | | 0.339 | | 1.234 | | 0.031 | | zinc finger protein 103 homolog (mouse) |  | |
| ZFP106 | | 1.055 | | 0.307 | | 0.878 | | 0.044 | | 1.550 | | 0.032 | | zinc finger protein 106 |  | |
| ZFP161 | | 1.007 | | 0.850 | | 0.881 | | 0.275 | | 1.491 | | 0.011 | | zinc finger protein 161 homolog (mouse) |  | |
| ZFX | | 1.091 | | 0.125 | | 0.895 | | 0.062 | | 1.251 | | 0.038 | | zinc finger protein, X-linked |  | |
| ZFY | | 1.272 | | 0.013 | | 1.127 | | 0.152 | | 1.396 | | 0.001 | | zinc finger protein, Y-linked |  | |
| ZIC1 | | 0.940 | | 0.318 | | 0.775 | | 0.008 | | 1.128 | | 0.841 | | Zic family member 1 (odd-paired homolog, Drosophila) |  | |
| ZIC4 | | 1.117 | | 0.059 | | 1.304 | | 0.106 | | 1.161 | | 0.040 | | zinc family member 4 protein HZIC4 |  | |
| ZID | | 1.205 | | 0.038 | | 0.977 | | 0.670 | | 1.102 | | 0.160 | | zinc finger protein with interaction domain |  | |
| ZIM2 | | 1.015 | | 0.742 | | 0.737 | | 0.003 | | 0.978 | | 0.530 | | zinc finger, imprinted 2 |  | |
| ZNF10 | | 0.324 | | 0.003 | | 0.271 | | 0.002 | | 0.604 | | 0.028 | | Zinc finger protein 10 (KOX 1) |  | |
| ZNF123 | | 0.986 | | 0.738 | | 1.024 | | 0.479 | | 1.481 | | 0.014 | | zinc finger protein 123 (HZF-1) |  | |
| ZNF135 | | 1.003 | | 0.936 | | 1.122 | | 0.152 | | 1.608 | | 0.011 | | zinc finger protein 135 (clone pHZ-17) |  | |
| ZNF136 | | 1.001 | | 0.988 | | 1.105 | | 0.548 | | 1.316 | | 0.038 | | zinc finger protein 136 (clone pHZ-20) |  | |
| ZNF137 | | 1.049 | | 0.334 | | 1.175 | | 0.153 | | 1.401 | | 0.049 | | zinc finger protein 137 (clone pHZ-30) |  | |
| ZNF140 | | 1.019 | | 0.701 | | 1.023 | | 0.680 | | 1.657 | | 0.004 | | zinc finger protein 140 (clone pHZ-39) |  | |
| ZNF142 | | 0.983 | | 0.747 | | 0.830 | | 0.021 | | 1.038 | | 0.745 | | zinc finger protein 142 (clone pHZ-49) |  | |
| ZNF144 | | 1.131 | | 0.048 | | 0.889 | | 0.212 | | 1.214 | | 0.045 | | zinc finger protein 144 (Mel-18) |  | |
| ZNF146 | | 1.030 | | 0.393 | | 0.978 | | 0.504 | | 1.222 | | 0.017 | | zinc finger protein 146 |  | |
| ZNF148 | | 0.998 | | 0.959 | | 1.044 | | 0.699 | | 1.638 | | 0.044 | | zinc finger protein 148 (pHZ-52) |  | |
| ZNF154 | | 1.017 | | 0.655 | | 0.961 | | 0.466 | | 1.305 | | 0.042 | | zinc finger protein 154 (pHZ-92) |  | |
| ZNF174 | | 0.716 | | 0.055 | | 0.596 | | 0.005 | | 1.151 | | 0.230 | | zinc finger protein 174 |  | |
| ZNF175 | | 0.946 | | 0.568 | | 1.091 | | 0.155 | | 1.581 | | 0.040 | | zinc finger protein 175 |  | |
| ZNF177 | | 0.924 | | 0.253 | | 1.064 | | 0.333 | | 1.811 | | 0.045 | | zinc finger protein 177 |  | |
| ZNF180 | | 1.024 | | 0.372 | | 1.034 | | 0.510 | | 1.366 | | 0.007 | | zinc finger protein 180 (HHZ168) |  | |
| ZNF184 | | 0.912 | | 0.323 | | 1.101 | | 0.247 | | 1.666 | | 0.036 | | zinc finger protein 184 (Kruppel-like) |  | |
| ZNF189 | | 0.903 | | 0.015 | | 1.082 | | 0.020 | | 1.332 | | 0.088 | | zinc finger protein 189 |  | |
| ZNF192 | | 0.891 | | 0.014 | | 0.970 | | 0.606 | | 1.575 | | 0.044 | | zinc finger protein 192 |  | |
| ZNF200 | | 1.003 | | 0.943 | | 1.383 | | 0.050 | | 1.470 | | 0.045 | | zinc finger protein 200 |  | |
| ZNF202 | | 0.884 | | 0.014 | | 1.396 | | 0.023 | | 1.200 | | 0.133 | | zinc finger protein 202 |  | |
| ZNF205 | | 0.983 | | 0.691 | | 1.036 | | 0.270 | | 0.803 | | 0.041 | | zinc finger protein 205 |  | |
| ZNF208 | | 1.030 | | 0.501 | | 1.114 | | 0.023 | | 1.361 | | 0.052 | | zinc finger protein 208 |  | |
| ZNF211 | | 1.184 | | 0.031 | | 1.413 | | 0.038 | | 1.074 | | 0.123 | | zinc finger protein 211 |  | |
| ZNF213 | | 0.945 | | 0.224 | | 1.011 | | 0.813 | | 1.382 | | 0.032 | | zinc finger protein 213 |  | |
| ZNF214 | | 1.001 | | 0.977 | | 1.169 | | 0.049 | | 1.576 | | 0.029 | | zinc finger protein 214 |  | |
| ZNF215 | | 0.905 | | 0.011 | | 1.164 | | 0.368 | | 1.613 | | 0.034 | | zinc finger protein 215 |  | |
| ZNF217 | | 0.962 | | 0.214 | | 1.214 | | 0.111 | | 1.387 | | 0.027 | | zinc finger protein 217 |  | |
| ZNF219 | | 0.968 | | 0.436 | | 0.945 | | 0.343 | | 1.728 | | 0.004 | | zinc finger protein 219 |  | |
| ZNF220 | | 1.076 | | 0.043 | | 0.888 | | 0.031 | | 1.348 | | 0.129 | | zinc finger protein 220 |  | |
| ZNF221 | | 1.088 | | 0.190 | | 0.824 | | 0.046 | | 1.341 | | 0.003 | | zinc finger protein 221 |  | |
| ZNF225 | | 1.014 | | 0.734 | | 0.873 | | 0.519 | | 1.648 | | 0.026 | | zinc finger protein 225 |  | |
| ZNF226 | | 1.123 | | 0.064 | | 1.263 | | 0.028 | | 1.616 | | 0.022 | | zinc finger protein 226 |  | |
| ZNF234 | | 1.005 | | 0.801 | | 1.151 | | 0.005 | | 1.244 | | 0.026 | | zinc finger protein 234 |  | |
| ZNF237 | | 1.014 | | 0.687 | | 0.920 | | 0.016 | | 1.256 | | 0.014 | | zinc finger protein 237 |  | |
| ZNF24 | | 0.883 | | 0.110 | | 0.959 | | 0.481 | | 1.312 | | 0.032 | | zinc finger protein 24 (KOX 17) |  | |
| ZNF254 | | 0.999 | | 0.976 | | 0.797 | | 0.122 | | 1.191 | | 0.026 | | zinc finger protein 254 |  | |
| ZNF256 | | 0.971 | | 0.526 | | 0.942 | | 0.360 | | 1.375 | | 0.033 | | zinc finger protein 256 |  | |
| ZNF258 | | 1.018 | | 0.674 | | 0.874 | | 0.814 | | 1.527 | | 0.026 | | zinc finger protein 258 |  | |
| ZNF262 | | 0.885 | | 0.045 | | 1.070 | | 0.571 | | 1.342 | | 0.020 | | zinc finger protein 262 |  | |
| ZNF263 | | 0.937 | | 0.152 | | 1.049 | | 0.449 | | 1.376 | | 0.032 | | zinc finger protein 263 |  | |
| ZNF265 | | 0.883 | | 0.214 | | 0.995 | | 0.882 | | 1.240 | | 0.014 | | zinc finger protein 265 |  | |
| ZNF268 | | 1.063 | | 0.281 | | 1.570 | | 0.037 | | 0.947 | | 0.193 | | zinc finger protein 268 |  | |
| ZNF271 | | 1.014 | | 0.632 | | 1.014 | | 0.712 | | 1.269 | | 0.021 | | zinc finger protein 271 |  | |
| ZNF272 | | 1.033 | | 0.315 | | 1.059 | | 0.225 | | 1.355 | | 0.003 | | zinc finger protein 272 |  | |
| ZNF273 | | 0.425 | | 0.274 | | 1.388 | | 0.103 | | 1.175 | | 0.127 | | zinc finger protein 273 (HZF9) |  | |
| ZNF277 | | 1.040 | | 0.312 | | 0.933 | | 0.181 | | 1.392 | | 0.007 | | zinc finger protein (C2H2 type) 277 |  | |
| ZNF282 | | 1.055 | | 0.157 | | 1.078 | | 0.151 | | 1.328 | | 0.007 | | zinc finger protein 282 |  | |
| ZNF287 | | 1.245 | | 0.012 | | 1.002 | | 0.958 | | 1.295 | | 0.016 | | zinc finger protein 287 |  | |
| ZNF294 | | 1.154 | | 0.190 | | 0.746 | | 0.020 | | 1.000 | | 0.997 | | zinc finger protein 294 |  | |
| ZNF295 | | 1.024 | | 0.405 | | 1.148 | | 0.217 | | 1.446 | | 0.043 | | zinc finger protein 295 |  | |
| ZNF297 | | 0.978 | | 0.420 | | 0.829 | | 0.062 | | 1.387 | | 0.050 | | zinc finger protein 297 |  | |
| ZNF3 | | 0.968 | | 0.345 | | 1.085 | | 0.207 | | 1.503 | | 0.019 | | zinc finger protein 3 (A8-51) |  | |
| ZNF304 | | 1.156 | | 0.086 | | 1.051 | | 0.376 | | 1.226 | | 0.035 | | zinc finger protein 304 |  | |
| ZNF33A | | 0.969 | | 0.637 | | 0.970 | | 0.453 | | 1.425 | | 0.028 | | zinc finger protein 33a (KOX 31) |  | |
| ZNF361 | | 0.994 | | 0.864 | | 0.876 | | 0.035 | | 1.374 | | 0.006 | | zinc finger protein 361 |  | |
| ZNF38 | | 0.702 | | 0.080 | | 0.598 | | 0.045 | | 0.727 | | 0.015 | | zinc finger protein 38 (KOX 25) |  | |
| ZNF46 | | 1.018 | | 0.655 | | 0.997 | | 0.954 | | 1.212 | | 0.099 | | zinc finger protein 46 (KUP) |  | |
| ZNF6 | | 1.089 | | 0.580 | | 1.106 | | 0.426 | | 1.706 | | 0.003 | | zinc finger protein 6 (CMPX1) |  | |
| ZNF7 | | 1.084 | | 0.032 | | 0.862 | | 0.030 | | 1.461 | | 0.021 | | zinc finger protein 7 (KOX 4, clone HF.16) |  | |
| ZNF73 | | 1.077 | | 0.187 | | 0.795 | | 0.372 | | 1.154 | | 0.196 | | zinc finger protein 73 (Cos12) |  | |
| ZNF75A | | 1.318 | | 4.24E-04 | | 1.729 | | 0.080 | | 1.100 | | 0.164 | | zinc finger protein 75a |  | |
| ZNF76 | | 0.687 | | 0.012 | | 0.973 | | 0.583 | | 0.932 | | 0.436 | | zinc finger protein 76 (expressed in testis) |  | |
| ZNF79 | | 1.251 | | 0.077 | | 1.088 | | 0.064 | | 1.313 | | 0.039 | | zinc finger protein 79 (pT7) |  | |
| ZNF8 | | 0.983 | | 0.319 | | 0.989 | | 0.790 | | 1.434 | | 0.039 | | zinc-finger protein 8 (clone HF.18) |  | |
| ZNF80 | | 0.824 | | 1.30E-04 | | 0.999 | | 0.987 | | 0.919 | | 0.116 | | zinc finger protein 80 (pT17) |  | |
| ZNF84 | | 0.890 | | 0.091 | | 0.932 | | 0.307 | | 1.280 | | 0.046 | | zinc finger protein 84 (HPF2) |  | |
| ZNF90 | | 1.210 | | 0.006 | | 1.042 | | 0.305 | | 1.244 | | 0.025 | | zinc finger protein 90 (HTF9) |  | |
| ZNF93 | | 1.251 | | 0.008 | | 1.015 | | 0.786 | | 1.038 | | 0.522 | | zinc finger protein 93 (HTF34) |  | |
| ZNFN1A3 | | 0.993 | | 0.845 | | 0.932 | | 0.323 | | 1.316 | | 0.047 | | zinc finger protein, subfamily 1A, 3 (Aiolos) |  | |
