# Supplementary material for: Large Scale Gene Expression Profiles of Regenerating Inner Ear Sensory Epithelia
Source: PLoS One. 2007 Jun 13;2(6):e525. doi: 10.1371/journal.pone.0000525 (PMC1888727; doi:10.1371/journal.pone.0000525)
Supplement: Table S6 — Cochlea Neomycin Differential Expression>1.2-fold and P< = 0.05 (0.10 MB PDF) [file pone.0000525.s007.pdf]

Supplemental Table S6

Gene ID	Entrez	O hr Fold change	O hr P-value	24 hr Fold Change	24 hr P-value	48 hr Fold Change	48 hr P-value	Updated syml	Updated description
TIMELESS	8914	1.3	0.034	1.378	0.006	1.358	0.047	TIMELESS	timeless homolog (Drosophila)
BAPX1	579	1.446	0.009	1.426	0.103	2.122	0.005	BAPX1	Bagpipe homeobox homolog 1 (Drosophila)
FLJ10697	55205	1.373	0.029	1.141	0.021	1.219	0.018	ZNF532	Zinc finger protein 532
ELF3	1999	1.233	2.28E-04	1.128	0.272	1.25	0.004	ELF3	E74-like factor 3 (ets domain transcription factor, epithelial-specific)
ZFY	7544	1.272	0.013	1.127	0.152	1.396	0.001	ZFY	Zinc finger protein, Y-linked
ZNF90	7643	1.21	0.006	1.042	0.305	1.244	0.025	ZNF90	zinc finger protein 90
ZNF287	57336	1.245	0.012	1.002	0.958	1.295	0.016	ZNF287	zinc finger protein 287
BRD7	29117	1.221	0.01	0.98	0.566	1.374	0.005	BRD7	Bromodomain containing 7
RNF22	10612	1.204	0.02	0.954	0.09	1.226	0.001	TRIM3	Tripartite motif-containing 3
LOC57209	57209	0.868	0.493	1.412	0.002	1.385	0.018	ZNF248	Zinc finger protein 248
MTA1L1	9219	1.177	0.218	1.218	0.002	1.281	0.009	MTA2	Metastasis associated 1 family, member 2
DFKZP434E0:	112398	1.294	0.1	1.758	0.011	1.352	0.021	EGLN2	egl nine homolog 2 (C. elegans)
HOXC10	3226	0.98	0.629	1.273	0.016	1.492	0.013	HOXC10	homeobox C10
HSAJ2425	55566	1.203	0.164	1.52	0.017	1.444	0.024	HSAJ2425	p65 protein
LHX6	26468	1.076	0.248	1.548	0.026	1.278	0.021	LHX6	LIM homeobox 6
ZNF226	7769	1.123	0.064	1.263	0.028	1.616	0.022	ZNF226	zinc finger protein 226
TAF2D	6877	0.929	0.347	1.245	0.043	1.492	0.048	TAF5	TAF5 RNA polymerase II, TATA box binding protein (TBP)-associated fact
ZNF200	7752	1.003	0.943	1.383	0.05	1.47	0.045	ZNF200	zinc finger protein 200
TRIP6	7205	0.955	0.281	1.608	0.062	1.617	0.034	TRIP6	thyroid hormone receptor interactor 6
DKFZp547H2	56917	1.078	0.166	1.635	0.064	1.272	0.001	MEIS3	Meis1, myeloid ecotropic viral integration site 1 homolog 3 (mouse)
HMX1	3166	1.027	0.486	1.25	0.066	1.258	0.019	HMX1	Homeobox (H6 family) 1
ISGF3G	10379	1.051	0.208	1.294	0.083	1.234	0.037	ISGF3G	interferon-stimulated transcription factor 3, gamma 48kDa
ZFHX1B	9839	0.903	0.022	1.23	0.083	1.344	0.034	ZFHX1B	Zinc finger homeobox 1b
NR0B2	8431	0.995	0.839	1.694	0.092	1.253	0.031	NR0B2	nuclear receptor subfamily 0, group B, member 2
NFATC1	4772	0.992	0.873	1.427	0.1	1.662	0.004	NFATC1	nuclear factor of activated T-cells, cytoplasmic, calcineurin-dependent 1
ZNF217	7764	0.962	0.214	1.214	0.111	1.387	0.027	ZNF217	zinc finger protein 217
PER2	8864	1.093	0.123	1.205	0.131	1.247	0.02	PER2	Period homolog 2 (Drosophila)
NSEP1	4904	1.108	0.08	1.182	0.006	1.56	0.001	YBX1	Y box binding protein 1
HOXB5	3215	1.018	0.665	1.177	0.144	1.357	0.01	HOXB5	homeobox B5
ZNF137	7696	1.049	0.334	1.175	0.153	1.401	0.049	ZNF137	zinc finger protein 137
TBR1	10716	1.05	0.499	1.174	0.052	1.339	0.05	TBR1	T-box, brain, 1
ZNF214	7761	1.001	0.977	1.169	0.049	1.576	0.029	ZNF214	Zinc finger protein 214
SLB	26160	1.054	0.226	1.168	0.203	1.284	0.016	IFT172	Intraflagellar transport 172 homolog (Chlamydomonas) /// selective LIM bin
ZNF215	7762	0.905	0.011	1.164	0.368	1.613	0.034	ZNF215	zinc finger protein 215
HOXC9	3225	1.021	0.373	1.159	0.046	1.293	0.031	HOXC9	homeobox C9
ZNF234	10780	1.005	0.801	1.151	0.005	1.244	0.026	ZNF234	zinc finger protein 234
ZNF295	49854	1.024	0.405	1.148	0.217	1.446	0.043	ZNF295	Zinc finger protein 295
CEBPB	1051	0.988	0.821	1.146	0.234	1.277	0.043	CEBPB	CCAAT/enhancer binding protein (C/EBP), beta
FLJ20729	54680	0.953	0.453	1.137	0.046	1.333	0.009	C1orf181	chromosome 1 open reading frame 181
SCML2	10389	1.106	0.063	1.137	0.316	1.309	0.04	SCML2	sex comb on midleg-like 2 (Drosophila)
ZNF135	7694	1.003	0.936	1.122	0.152	1.608	0.011	ZNF135	Zinc finger protein 135
NR5A2	2494	1.04	0.449	1.12	0.08	1.242	0.05	NR5A2	nuclear receptor subfamily 5, group A, member 2
ZNF208	7757	1.03	0.501	1.114	0.023	1.361	0.052	ZNF208	zinc finger protein 208
MADH9	4093	1.182	0.244	1.112	0.058	1.256	0.047	SMAD9	SMAD, mothers against DPP homolog 9 (Drosophila)
DR1	1810	0.995	0.821	1.109	0.044	1.242	0.033	DR1	down-regulator of transcription 1, TBP-binding (negative cofactor 2)
HSA275986	55802	1.145	0.008	1.108	0.109	1.215	0.016	DCP1A	DCP1 decapping enzyme homolog A (S. cerevisiae)
ZNF6	7552	1.089	0.58	1.106	0.426	1.706	0.003	ZNF711	Zinc finger protein 711

ZNF136	7695	1.001	0.988	1.105	0.548	1.316	0.038	ZNF136	zinc finger protein 136
ZNF184	7738	0.912	0.323	1.101	0.247	1.666	0.036	ZNF184	zinc finger protein 184
RFP2	10206	1.008	0.776	1.094	0.099	1.371	0.001	RFP2	ret finger protein 2
ZNF175	7728	0.946	0.568	1.091	0.155	1.581	0.04	ZNF175	zinc finger protein 175
ZNF79	7633	1.251	0.077	1.088	0.064	1.313	0.039	ZNF79	zinc finger protein 79
TBX15	6913	1.115	0.071	1.088	0.198	1.218	0.004	TBX15	T-box 15
ZNF3	7551	0.968	0.345	1.085	0.207	1.503	0.019	ZNF3	zinc finger protein 3
ZNF282	8427	1.055	0.157	1.078	0.151	1.328	0.007	ZNF282	zinc finger protein 282
BLZF1	8548	1.124	0.162	1.075	0.157	1.374	0.034	BLZF1	Basic leucine zipper nuclear factor 1 (JEM-1)
ZNF262	9202	0.885	0.045	1.07	0.571	1.342	0.02	ZMYM4	zinc finger, MYM-type 4
PRDM9	56979	1.09	0.279	1.069	0.237	1.249	0.002	PRDM9	PR domain containing 9
FUBP1	8880	0.989	0.7	1.068	0.227	1.408	0.03	FUBP1	far upstream element (FUSE) binding protein 1
DLX1	1745	0.974	0.326	1.066	0.271	1.258	0.036	DLX1	distal-less homeobox 1
ILF3	3609	0.919	0.742	1.065	0.34	1.328	0.007	ILF3	interleukin enhancer binding factor 3, 90kDa
ZNF177	7730	0.924	0.253	1.064	0.333	1.811	0.045	ZNF177	zinc finger protein 177
TZFP	27033	1.083	0.26	1.062	0.324	1.61	0.044	ZBTB32	zinc finger and BTB domain containing 32
POU3F2	5454	0.945	0.469	1.062	0.452	1.441	0.008	POU3F2	POU domain, class 3, transcription factor 2
ZNF272	10794	1.033	0.315	1.059	0.225	1.355	0.003	ZNF272	Zinc finger protein 272
FOXD1	2297	1.086	0.124	1.056	0.134	1.221	0.028	FOXD1	forkhead box D1
ZNF304	57343	1.156	0.086	1.051	0.376	1.226	0.035	ZNF304	zinc finger protein 304
ZNF263	10127	0.937	0.152	1.049	0.449	1.376	0.032	ZNF263	zinc finger protein 263
EPLIN	51474	1.123	0.015	1.047	0.31	1.316	0.039	LIMA1	LIM domain and actin binding 1
PROP1	5626	0.991	0.782	1.047	0.352	1.271	0.054	PROP1	prophet of Pit1, paired-like homeodomain transcription factor
YAF2	10138	1.038	0.547	1.046	0.452	1.476	0.033	YAF2	YY1 associated factor 2
HLF	3131	1.399	0.229	1.046	0.399	1.29	0.049	HLF	hepatic leukemia factor
ZNF148	7707	0.998	0.959	1.044	0.699	1.638	0.044	ZNF148	zinc finger protein 148
TBX3	6926	1.052	0.727	1.044	0.251	1.275	0.013	TBX3	T-box 3 (ulnar mammary syndrome)
LMO2	4005	0.901	0.143	1.038	0.339	1.298	0.038	LMO2	LIM domain only 2 (rhombotin-like 1)
ZNF180	7733	1.024	0.372	1.034	0.51	1.366	0.007	ZNF180	zinc finger protein 180
TMF1	7110	0.956	0.231	1.033	0.454	1.455	0.023	TMF1	TATA element modulatory factor 1
EYA1	2138	1.035	0.296	1.033	0.435	1.322	0.004	EYA1	eyes absent homolog 1 (Drosophila)
HOXB9	3219	0.934	0.142	1.031	0.306	1.208	0.033	HOXB9	homeobox B9
PBX1	5087	1.056	0.178	1.027	0.617	1.257	0.003	PBX1	Pre-B-cell leukemia transcription factor 1
PKNOX1	5316	1.079	0.105	1.026	0.705	1.424	0.007	PKNOX1	PBX/knotted 1 homeobox 1
TAF1C	9013	1.044	0.39	1.025	0.655	1.26	0.038	TAF1C	TATA box binding protein (TBP)-associated factor, RNA polymerase I, C, 1
ZNF123	7677	0.986	0.738	1.024	0.479	1.481	0.014	ZNF123	zinc finger protein 123
ZNF140	7699	1.019	0.701	1.023	0.68	1.657	0.004	ZNF140	zinc finger protein 140
HOXA10	3206	0.988	0.786	1.021	0.712	1.263	0.047	HOXA10	homeobox A10
TCF12	6938	0.89	0.223	1.021	0.491	1.229	0.019	TCF12	transcription factor 12 (HTF4, helix-loop-helix transcription factors 4)
WHN	8456	0.979	0.512	1.017	0.716	1.551	0.002	FOXN1	Forkhead box N1
ZNF271	10778	1.014	0.632	1.014	0.712	1.269	0.021	ZNF271	zinc finger protein 271
NR4A3	8013	1.159	0.089	1.012	0.83	1.806	0.006	NR4A3	nuclear receptor subfamily 4, group A, member 3
ZNF213	7760	0.945	0.224	1.011	0.813	1.382	0.032	ZNF213	zinc finger protein 213
TRIM22	10346	1.035	0.64	1.009	0.854	1.234	0.021	TRIM22	tripartite motif-containing 22
TNRC4	11189	1.104	0.079	1.007	0.884	1.277	1.47E-04	TNRC4	trinucleotide repeat containing 4
KIAA0535	9705	0.975	0.364	1.001	0.979	1.21	0.048	ST18	suppression of tumorigenicity 18 (breast carcinoma) (zinc finger protein)
TCF8	6935	0.804	0.064	1	0.998	1.279	0.031	TCF8	transcription factor 8 (represses interleukin 2 expression)
FLJ23309	79956	1.163	0.157	0.997	0.934	1.302	0.023	KIAA1815	KIAA1815
EGR2	1959	0.981	0.492	0.995	0.937	1.479	0.045	EGR2	early growth response 2 (Krox-20 homolog, Drosophila)
ZNF265	9406	0.883	0.214	0.995	0.882	1.24	0.014	ZRANB2	zinc finger, RAN-binding domain containing 2
ZNF8	7554	0.983	0.319	0.989	0.79	1.434	0.039	ZNF8	zinc finger protein 8

EN1	2019	1.053	0.435	0.986	0.757	1.438	0.016	EN1	engrailed homolog 1
LOC51045	51045	0.992	0.766	0.982	0.617	1.201	0.005	LOC51045	Discontinued
ARNTL	406	1.002	0.946	0.98	0.457	1.219	0.003	ARNTL	aryl hydrocarbon receptor nuclear translocator-like
LOC51043	51043	0.978	0.638	0.978	0.485	1.247	0.005	ZBTB7B	zinc finger and BTB domain containing 7B
ZNF146	7705	1.03	0.393	0.978	0.504	1.222	0.017	ZNF146	zinc finger protein 146
TBX5	6910	1.158	0.056	0.976	0.756	1.252	0.005	TBX5	T-box 5
NR2F2	7026	1.053	0.267	0.975	0.802	1.635	0.001	NR2F2	nuclear receptor subfamily 2, group F, member 2
AIB3	497657	1.063	0.109	0.973	0.49	1.214	0.031	ANIB3	aneurysm, intracranial berry 3
ZNF192	7745	0.891	0.014	0.97	0.606	1.575	0.044	ZNF192	zinc finger protein 192
ZNF33A	7581	0.969	0.637	0.97	0.453	1.425	0.028	ZNF33A	zinc finger protein 33A
MLLT2	4299	1.21	0.101	0.97	0.671	1.316	0.019	AFF1	AF4/FMR2 family, member 1
YY1	7528	0.866	0.009	0.968	0.625	1.603	0.013	YY1	YY1 transcription factor
ZNF154	7710	1.017	0.655	0.961	0.466	1.305	0.042	ZNF154	zinc finger protein 154
ZNF24	7572	0.883	0.11	0.959	0.481	1.312	0.032	ZNF24	zinc finger protein 24
KIAA1442	57593	1.016	0.654	0.954	0.437	1.341	0.004	RP5-860F19.3	KIAA1442 protein
PBX3	5090	1.001	0.979	0.953	0.189	1.434	0.001	PBX3	pre-B-cell leukemia transcription factor 3
BCL11A	53335	1.136	0.172	0.952	0.364	1.437	0.009	BCL11A	B-cell CLL/lymphoma 11A (zinc finger protein)
RNF10	9921	1.154	0.05	0.951	0.28	1.261	0.005	RNF10	ring finger protein 10
ZNF219	51222	0.968	0.436	0.945	0.343	1.728	0.004	ZNF219	zinc finger protein 219
ZNF256	10172	0.971	0.526	0.942	0.36	1.375	0.033	ZNF256	zinc finger protein 256
MGC16733	92105	0.932	0.33	0.939	0.151	1.217	0.019	INTS4	integrator complex subunit 4
LMX1B	4010	0.899	0.514	0.937	0.251	1.313	0.017	LMX1B	LIM homeobox transcription factor 1, beta
CREG	8804	1.138	0.106	0.934	0.086	1.252	0.039	CREG1	Cellular repressor of E1A-stimulated genes 1
MYT1L	23040	0.997	0.968	0.934	0.218	1.213	0.044	MYT1L	myelin transcription factor 1-like
ZNF277	11179	1.04	0.312	0.933	0.181	1.392	0.007	ZNF277	zinc finger protein 277
ZNFN1A3	22806	0.993	0.845	0.932	0.323	1.316	0.047	IKZF3	IKAROS family zinc finger 3 (Aiolos)
ZNF84	7637	0.89	0.091	0.932	0.307	1.28	0.046	ZNF84	zinc finger protein 84
RARG	5916	0.948	0.465	0.931	0.254	1.308	0.013	RARG	retinoic acid receptor, gamma
ZFP103	7844	0.959	0.363	0.93	0.339	1.234	0.031	RNF103	ring finger protein 103
TRAP150	9967	0.997	0.918	0.929	0.26	1.219	0.004	THRAP3	thyroid hormone receptor associated protein 3
ZNF237	9205	1.014	0.687	0.92	0.016	1.256	0.014	ZMYM5	zinc finger, MYM-type 5
TNRC6	27327	1.168	0.02	0.917	0.039	1.257	0.008	TNRC6A	Trinucleotide repeat containing 6A
NFIC	4782	1.123	0.019	0.917	0.258	1.236	0.035	NFIC	nuclear factor I/C (CCAAT-binding transcription factor)
LDB2	9079	0.966	0.496	0.911	0.003	1.262	0.029	LDB2	LIM domain binding 2
RUNX2	860	1.049	0.284	0.911	0.106	1.217	0.012	RUNX2	runt-related transcription factor 2
TRIP11	9321	1.018	0.653	0.906	0.152	1.25	0.011	TRIP11	thyroid hormone receptor interactor 11
HSGT1	11319	1.029	0.615	0.904	0.022	1.232	0.002	ECD	ecdysoneless homolog (Drosophila)
ZFX	7543	1.091	0.125	0.895	0.062	1.251	0.038	ZFX	Zinc finger protein, X-linked
NMI	9111	1.067	0.119	0.894	0.017	1.272	0.003	NMI	N-myc (and STAT) interactor
RORA	6095	0.991	0.832	0.894	0.141	1.255	0.014	RORA	RAR-related orphan receptor A
ZNF144	7703	1.131	0.048	0.889	0.212	1.214	0.045	PCGF2	Polycomb group ring finger 2 /// zinc finger protein 144
SRA1	10011	1.422	0.134	0.886	4.29E-05	1.333	0.002	SRA1	Steroid receptor RNA activator 1
ZFP161	7541	1.007	0.85	0.881	0.275	1.491	0.011	ZFP161	zinc finger protein 161 homolog (mouse)
ZFP106	64397	1.055	0.307	0.878	0.044	1.55	0.032	ZFP106	zinc finger protein 106 homolog (mouse)
ZNF361	55422	0.994	0.864	0.876	0.035	1.374	0.006	ZNF331	zinc finger protein 331
PCAR	AF174394	1.059	0.171	0.876	0.018	1.28	0.018	PCAR	Hypothetical protein I38022
PC4	3475	1.081	0.552	0.875	0.344	1.356	0.017	IFRD1	Interferon-related developmental regulator 1 /// nerve growth factor-inducibl
ZNF258	9204	1.018	0.674	0.874	0.814	1.527	0.026	ZMYM6	zinc finger, MYM-type 6
ZF5128	25799	0.921	0.861	0.873	0.005	1.499	0.018	ZNF324	Zinc finger protein 324
ZNF225	7768	1.014	0.734	0.873	0.519	1.648	0.026	ZNF225	zinc finger protein 225
NR2F1	7025	1.067	0.248	0.869	0.48	1.398	0.001	NR2F1	Nuclear receptor subfamily 2, group F, member 1

UTF1	8433	1.246	0.093	0.869	0.332	1.35	0.015	UTF1	undifferentiated embryonic cell transcription factor 1
RRN3	54700	1.125	0.353	0.865	0.028	1.273	0.043	RRN3	RRN3 RNA polymerase I transcription factor homolog (S. cerevisiae)
ZNF7	7553	1.084	0.032	0.862	0.03	1.461	0.021	ZNF7	zinc finger protein 7
RPF-1	11281	1.009	0.88	0.86	0.039	1.23	0.033	POU6F2	POU domain, class 6, transcription factor 2
ETS1	2113	0.902	0.662	0.853	0.05	1.219	0.05	ETS1	v-ets erythroblastosis virus E26 oncogene homolog 1 (avian)
MYOD1	4654	1.016	0.815	0.838	0.076	1.233	0.001	MYOD1	myogenic differentiation 1
MYCBP	26292	1.058	0.115	0.792	0.001	1.266	0.001	MYCBP	c-myc binding protein
HSPX153	54729	1.002	0.938	0.789	0.012	1.214	0.018	NKX1-1	NK1 transcription factor related, locus 1 (Drosophila)
NHLH2	4808	1.133	0.256	0.808	0.019	1.43	0.014	NHLH2	nescient helix loop helix 2
ZNF221	7638	1.088	0.19	0.824	0.046	1.341	0.003	ZNF221	zinc finger protein 221
ZNF297	9278	0.978	0.42	0.829	0.062	1.387	0.05	ZBTB22	zinc finger and BTB domain containing 22
SSX3	10214	1.059	0.396	0.799	0.063	1.295	0.022	SSX3	synovial sarcoma, X breakpoint 3
H-L(3)MBT	83746	0.951	0.273	0.815	0.084	1.233	0.01	L3MBTL2	L(3)mbt-like 2 (Drosophila)
FLJ11186	55320	1.262	0.665	0.73	0.098	1.531	0.053	C14orf106	Chromosome 14 open reading frame 106
ZNF254	399655	0.999	0.976	0.797	0.122	1.191	0.026	ZNF254	zinc finger protein 254 /// zinc finger protein 539
POU2F2	5452	1.013	0.659	0.758	0.139	1.34	0.012	POU2F2	POU domain, class 2, transcription factor 2 /// POU domain, class 2, transcri
CTNNB1	1499	1.18	0.304	0.76	0.336	1.374	0.001	CTNNB1	catenin (cadherin-associated protein), beta 1, 88kDa
KIAA0026	9643	0.824	0.02	1.024	0.692	1.206	0.012	MORF4L2	mortality factor 4 like 2
ESR1	2099	1.379	0.024	1.499	3.14E-04	1.25	0.177	ESR1	estrogen receptor 1
PTTG1IP	754	1.401	0.04	1.495	0.001	1.013	0.864	PTTG1IP	pituitary tumor-transforming 1 interacting protein
HOXA4	3201	1.2	0.037	1.253	0.051	1.208	0.093	HOXA4	homeobox A4
ZNF75A	7627	1.318	4.24E-04	1.729	0.08	1.1	0.164	ZNF75A	Zinc finger protein 75a
CART1	8092	1.5	0.013	1.383	0.095	1.002	0.957	CART1	cartilage paired-class homeoprotein 1
MED6	10001	1.342	0.006	1.119	0.586	1.122	0.173	MED6	Mediator of RNA polymerase II transcription, subunit 6 homolog (S. cerevis
FLJ10759	55223	1.329	0.009	1.101	0.104	1.232	0.073	TRIM62	tripartite motif-containing 62
CUTL1	1523	1.211	0.014	1.079	0.587	1.007	0.944	CUTL1	Cut-like 1, CCAAT displacement protein (Drosophila)
ICBP90	29128	1.236	0.027	1.07	0.082	0.976	0.605	UHRF1	ubiquitin-like, containing PHD and RING finger domains, 1
WHSC1	7468	1.275	0.026	1.06	0.23	1.098	0.075	WHSC1	Wolf-Hirschhorn syndrome candidate 1
PAX8	7849	1.278	0.035	1.053	0.124	0.818	0.11	PAX8	paired box gene 8
LMO6	4007	1.206	0.006	1.048	0.508	0.865	0.011	LMO6	LIM domain only 6
POU4F1	5457	1.383	0.011	1.033	0.688	1.324	0.089	POU4F1	POU domain, class 4, transcription factor 1
ZNF93	81931	1.251	0.008	1.015	0.786	1.038	0.522	ZNF93	Zinc finger protein 93
P38IP	55578	1.228	0.008	1.009	0.863	0.85	0.005	FAM48A	family with sequence similarity 48, member A
CHD3	1107	1.232	0.048	0.98	0.758	0.762	0.106	CHD3	Chromodomain helicase DNA binding protein 3
ZID	10773	1.205	0.038	0.977	0.67	1.102	0.16	ZBTB6	Zinc finger and BTB domain containing 6
FOXC2	2303	1.235	0.036	0.955	0.524	1.035	0.583	FOXC2	forkhead box C2 (MFH-1, mesenchyme forkhead 1)
LOC58500	58500	1.219	0.002	0.947	0.062	1.033	0.483	ZNF250	Zinc finger protein 250
CRSP6	9440	1.21	0.004	0.888	0.334	1.033	0.692	CRSP6	cofactor required for Sp1 transcriptional activation, subunit 6, 77kDa
TADA3L	10474	1.222	0.034	0.807	0.021	0.924	0.37	TADA3L	transcriptional adaptor 3 (NGG1 homolog, yeast)-like
GATA6	2627	1.026	0.617	1.234	4.37E-05	0.898	0.08	GATA6	GATA binding protein 6
CBX6	23466	1.089	0.043	1.2	8.07E-05	1.047	0.506	CBX6	chromobox homolog 6
HOXA7	3204	1.099	0.183	1.416	0.001	0.977	0.635	HOXA7	Homeobox A7
SAFB	6294	1.142	0.196	1.408	0.001	1.043	0.582	SAFB	scaffold attachment factor B
FOXC1	2296	1.034	0.272	1.319	0.001	0.89	0.242	FOXC1	forkhead box C1
IRLB	10260	1.275	0.089	1.244	0.001	0.862	0.04	DENND4A	DENN/MADD domain containing 4A
SETDB1	9869	0.953	0.532	1.33	0.004	0.975	0.57	SETDB1	SET domain, bifurcated 1
MDS1	4197	1.018	0.791	1.565	0.005	1.196	0.072	MDS1	Myelodysplasia syndrome 1
CDK8	1024	1.114	0.073	1.241	0.005	1.013	0.844	CDK8	cyclin-dependent kinase 8
HEY2	23493	1.067	0.212	1.446	0.007	1.062	0.191	HEY2	Hairy/enhancer-of-split related with YRPW motif 2
GTF2H2	2966	1.143	0.237	1.386	0.008	0.863	0.166	GTF2H2	General transcription factor IIH, polypeptide 2, 44kDa
M96	22823	0.999	0.988	1.235	0.008	0.926	0.342	MTF2	Metal response element binding transcription factor 2

GBX1	2636	1.002	0.974	1.208	0.008	0.848	0.072	GBX1	gastrulation brain homeobox 1
PAX7	5081	1.181	0.001	1.407	0.011	0.879	0.066	PAX7	paired box gene 7
HOXD4	3233	1.097	0.203	1.217	0.011	1.035	0.363	HOXD4	homeobox D4
RBBP9	10741	1.067	0.319	1.312	0.013	0.77	0.402	RBBP9	retinoblastoma binding protein 9
HOXB8	3218	1.265	0.065	1.197	0.013	0.952	0.355	HOXB8	homeobox B8
TCEAL1	9338	1.072	0.273	1.306	0.014	0.854	0.025	TCEAL1	transcription elongation factor A (SII)-like 1
GLI	2735	0.951	0.37	1.395	0.019	0.939	0.604	GLI	glioma-associated oncogene homolog 1 (zinc finger protein)
KIAA0998	23093	1.026	0.659	1.262	0.019	1.269	0.06	TTLL5	Tubulin tyrosine ligase-like family, member 5
SSX1	652630	1.12	0.204	1.25	0.02	1.125	0.432	SSX1	synovial sarcoma, X breakpoint 1 /// similar to synovial sarcoma, X breakpo
TBX21	30009	1.17	0.172	1.4	0.022	1.044	0.508	TBX21	T-box 21
PPARG	5468	1.061	0.555	1.535	0.023	1.04	0.489	PPARG	peroxisome proliferative activated receptor, gamma
ZNF202	7753	0.884	0.014	1.396	0.023	1.2	0.133	ZNF202	zinc finger protein 202
NFIB	4781	1.034	0.61	1.363	0.027	1.339	0.202	NFIB	nuclear factor I/B
HOXD8	3234	0.896	0.377	1.267	0.027	1.052	0.532	HOXD8	Homeobox D8
GTF2A1	2957	0.903	0.726	1.429	0.028	1.346	0.109	GTF2A1	general transcription factor IIA, 1, 19/37kDa
NFKB2	4791	1.027	0.741	1.565	0.033	0.97	0.584	NFKB2	nuclear factor of kappa light polypeptide gene enhancer in B-cells 2 (p49/p1
GAS41	8089	1.078	0.064	1.217	0.035	1.156	0.053	YEATS4	YEATS domain containing 4
ZNF268	10795	1.063	0.281	1.57	0.037	0.947	0.193	ZNF268	Zinc finger protein 268
ZNF211	10520	1.184	0.031	1.413	0.038	1.074	0.123	ZNF211	zinc finger protein 211
PRDM2	7799	1.047	0.247	1.351	0.038	0.859	0.028	PRDM2	PR domain containing 2, with ZNF domain
EGR1	1958	1.029	0.531	1.22	0.038	0.845	0.175	EGR1	early growth response 1
MAF	4094	0.993	0.774	1.21	0.038	0.857	0.002	MAF	V-maf musculoaponeurotic fibrosarcoma oncogene homolog (avian)
PER1	5187	1.118	0.012	1.264	0.04	1.029	0.657	PER1	period homolog 1 (Drosophila)
HSPC018	9567	1.056	0.253	1.325	0.043	1.189	0.005	GTPBP1	GTP binding protein 1
SSX2	6757	1.137	0.131	1.546	0.044	1.157	0.105	SSX2	synovial sarcoma, X breakpoint 2
LOC56270	56270	1.054	0.579	1.294	0.049	1.213	0.092	WDR45L	WDR45-like
FOG2	23414	0.803	0.074	0.589	2.14E-05	0.811	0.341	ZFPM2	Friend of GATA2 /// zinc finger protein, multitype 2
MAD4	10608	0.949	0.461	0.826	7.90E-05	1.039	0.659	MXD4	MAX dimerization protein 4
HOXA3	3200	0.75	0.113	0.594	1.94E-04	1.006	0.884	HOXA3	homeobox A3 /// homeobox A3
FOXP1	27086	0.872	0.346	0.739	3.13E-04	0.95	0.211	FOXP1	Forkhead box P1
C21orf18	54093	0.75	0.529	0.675	0.001	0.713	0.09	SETD4	SET domain containing 4
BRD1	23774	0.913	0.288	0.579	0.001	0.909	0.27	BRD1	bromodomain containing 1
RNF13	11342	1.002	0.961	0.807	0.002	1.1	0.421	RNF13	ring finger protein 13
HSF2BP	11077	1.091	0.361	0.798	0.003	1	0.999	HSF2BP	Heat shock transcription factor 2 binding protein
ZIM2	23619	1.015	0.742	0.737	0.003	0.978	0.53	ZIM2	zinc finger, imprinted 2
BTF3L1	690	0.835	0.125	0.666	0.003	1.054	0.561	BTF3L1	basic transcription factor 3, like 1
TITF1	7080	0.975	0.832	0.821	0.004	0.923	0.156	TITF1	thyroid transcription factor 1
MAPK8IP1	9479	1.082	0.223	0.798	0.004	0.943	0.384	MAPK8IP1	mitogen-activated protein kinase 8 interacting protein 1
HMG2	3148	0.96	0.282	0.715	0.004	1.177	0.039	HMGB2	high-mobility group box 2
MYT2	8827	0.856	0.238	0.771	0.005	0.836	0.196	MYT2	Myelin transcription factor 2
ZNF174	7727	0.716	0.055	0.596	0.005	1.151	0.23	ZNF174	zinc finger protein 174
NEUROG2	63973	0.995	0.862	0.813	0.006	1.002	0.974	NEUROG2	neurogenin 2
TFCP2	7024	0.97	0.758	0.784	0.006	1.047	0.438	TFCP2	transcription factor CP2
FLJ10142	55079	0.845	0.212	0.524	0.007	1.2	0.069	FEZF2	FEZ family zinc finger 2
ZIC1	7545	0.94	0.318	0.775	0.008	1.128	0.841	ZIC1	Zic family member 1 (odd-paired homolog, Drosophila)
HRIHFB2122	11078	0.932	0.29	0.693	0.008	0.823	0.197	TRIOBP	TRIO and F-actin binding protein
TNRC3	55534	0.984	0.693	0.783	0.009	1.009	0.819	MAML3	mastermind-like 3 (Drosophila)
KIAA0071	23186	1.023	0.386	0.789	0.01	0.967	0.6	RCOR1	REST corepressor 1
IRX7	79190	0.901	0.166	0.757	0.01	0.858	0.002	IRX6	iroquois homeobox protein 6
HOXB3	3213	0.995	0.793	0.638	0.01	1.075	0.172	HOXB3	homeobox B3
SOX2	6657	1.023	0.501	0.58	0.013	1.016	0.773	SOX2	SRY (sex determining region Y)-box 2

LOC91120	91120	0.94	0.241	0.825	0.014	0.973	0.699	ZNF682	Zinc finger protein 682
GTF2I	2969	1.026	0.757	0.802	0.015	0.733	0.133	GTF2I	General transcription factor II, i
ZNF294	26046	1.154	0.19	0.746	0.02	1	0.997	ZNF294	zinc finger protein 294
ATF4	468	0.983	0.565	0.556	0.02	1.062	0.533	ATF4	activating transcription factor 4 (tax-responsive enhancer element B67)
ZNF142	7701	0.983	0.747	0.83	0.021	1.038	0.745	ZNF142	zinc finger protein 142
MSC	9242	0.813	0.1	0.773	0.022	1.057	0.398	MSC	Musculin (activated B-cell factor-1)
GIOT-2	51710	0.85	0.105	0.733	0.022	0.955	0.435	ZNF44	Zinc finger protein 44
NR2E3	10002	1.093	0.556	0.808	0.024	0.995	0.875	NR2E3	nuclear receptor subfamily 2, group E, member 3
BCL11B	64919	0.88	0.037	0.786	0.025	0.985	0.84	BCL11B	B-cell CLL/lymphoma 11B (zinc finger protein)
NCOR1	9611	0.906	0.688	0.766	0.025	1.327	0.092	NCOR1	Nuclear receptor co-repressor 1
MORF	23522	0.953	0.476	0.775	0.03	0.963	0.441	MYST4	MYST histone acetyltransferase (monocytic leukemia) 4
RNF14	9604	0.907	0.413	0.785	0.031	1.015	0.835	RNF14	ring finger protein 14
PURA	5813	0.977	0.658	0.792	0.044	0.908	0.185	PURA	purine-rich element binding protein A
RARA	5914	0.913	0.334	0.762	0.044	0.984	0.56	RARA	retinoic acid receptor, alpha
TCFL5	10732	0.806	0.052	1.761	0.052	1.06	0.24	TCFL5	Transcription factor-like 5 (basic helix-loop-helix)
LOC57167	57167	0.816	0.014	1.393	0.132	1.125	0.307	SALL4	Sal-like 4 (Drosophila)
TRIP15	9318	0.682	0.003	1.131	0.101	0.962	0.455	COPS2	COP9 constitutive photomorphogenic homolog subunit 2 (Arabidopsis)
PREB	10113	0.542	0.009	1.09	0.638	0.819	0.091	PREB	Prolactin regulatory element binding
MAX	4149	0.683	0.002	1.042	0.898	1.099	0.041	MAX	MYC associated factor X
THRA	7067	0.827	0.017	1.039	0.763	1.045	0.539	THRA	thyroid hormone receptor, alpha (erythroblastic leukemia viral (v-erb-a) onc
TEAD3	7005	0.823	0.014	1.007	0.882	0.916	0.047	TEAD3	TEA domain family member 3
ZNF80	7634	0.824	1.30E-04	0.999	0.987	0.919	0.116	ZNF80	Zinc finger protein 80
DRPLA	1822	0.817	0.054	0.975	0.517	0.881	0.348	ATN1	Atrophin 1
ZNF76	7629	0.687	0.012	0.973	0.583	0.932	0.436	ZNF76	zinc finger protein 76 (expressed in testis)
KIAA0173	9654	0.412	0.002	0.948	0.24	0.786	0.078	TTLL4	Tubulin tyrosine ligase-like family, member 4
TAF2N	8148	0.812	0.018	0.937	0.319	0.846	0.04	TAF15	TAF15 RNA polymerase II, TATA box binding protein (TBP)-associated fa
HCNGP	29115	0.651	0.023	0.923	0.144	0.918	0.306	SAP30BP	SAP30 binding protein
MEF2B	4207	0.468	0.006	0.915	0.449	1.158	0.079	MEF2B	MADS box transcription enhancer factor 2, polypeptide B (myocyte enhance
TAL2	6887	0.771	4.09E-04	0.891	0.267	1.019	0.823	TAL2	T-cell acute lymphocytic leukemia 2
POU4F2	5458	0.731	0.011	0.86	0.133	0.648	0.301	POU4F2	POU domain, class 4, transcription factor 2
TAF-172	9044	0.745	0.005	0.838	0.12	0.846	0.116	BTAF1	BTAF1 RNA polymerase II, B-TFIID transcription factor-associated, 170kD
PAF65A	10629	0.603	0.041	0.368	1.48E-05	0.741	0.346	TAF6L	TAF6-like RNA polymerase II, p300/CBP-associated factor (PCAF)-associa
NEUROD6	63974	0.737	0.025	0.691	1.20E-04	0.736	0.086	NEUROD6	neurogenic differentiation 6
SDCCAG33	10194	0.827	0.051	0.816	0.001	0.93	0.176	TSHZ1	teashirt family zinc finger 1
TRIM15	89870	0.795	0.036	0.708	0.001	0.965	0.497	TRIM15	tripartite motif-containing 15
PMX1	5396	0.783	0.04	0.629	0.002	0.808	0.164	PRRX1	Paired related homeobox 1
GBX2	2637	0.457	0.004	0.496	0.003	0.867	0.087	GBX2	gastrulation brain homeobox 2
MTA1	9112	0.747	0.038	0.681	0.005	0.856	0.106	MTA1	Metastasis associated 1
TNRC5	10695	0.623	0.026	0.454	0.005	1	0.997	TNRC5	trinucleotide repeat containing 5
ARIX	401	0.805	0.004	0.785	0.007	0.959	0.669	PHOX2A	Paired-like (aristaless) homeobox 2a
PRDM13	59336	0.549	0.019	0.4	0.013	0.665	0.104	PRDM13	PR domain containing 13
FOXH1	8928	0.678	0.003	0.732	0.017	0.835	0.073	FOXH1	Forkhead box H1
RNF4	6047	0.666	0.001	0.654	0.023	0.984	0.936	RNF4	ring finger protein 4
PAX1	5075	0.719	1.00E-05	0.822	0.085	1.007	0.915	PAX1	Paired box gene 1
FLJ20595	54993	0.8	0.017	0.634	0.102	0.825	0.067	ZSCAN2	Zinc finger and SCAN domain containing 2
DEAF1	10522	0.72	0.004	0.555	0.127	0.976	0.6	DEAF1	deformed epidermal autoregulatory factor 1 (Drosophila)
KIAA1041	22887	0.767	0.027	0.827	0.299	0.861	0.061	FOXJ3	Forkhead box J3
HHEX	3087	1.26	0.009	1.37	0.003	0.714	0.005	HHEX	homeobox, hematopoietically expressed
LOC51058	51058	1.258	0.008	1.117	0.314	0.709	0.033	ZNF691	Zinc finger protein 691
NFKBIL1	4795	1.256	0.047	1.079	0.376	0.83	0.007	NFKBIL1	nuclear factor of kappa light polypeptide gene enhancer in B-cells inhibitor-l
BTEB1	687	1.245	0.048	0.977	0.588	0.829	0.012	KLF9	Kruppel-like factor 9

RXRB	6257	1.27	0.006	0.97	0.543	0.705	0.015	RXRB	retinoid X receptor, beta
PAX5	5079	1.215	0.026	0.946	0.247	0.727	0.047	PAX5	Paired box gene 5 (B-cell lineage specific activator)
HDAC4	9759	1.137	4.52E-05	1.252	1.63E-04	0.802	7.38E-04	HDAC4	histone deacetylase 4
EOMES	8320	1.142	0.032	1.3	0.001	0.796	0.048	EOMES	eomesodermin homolog (Xenopus laevis)
HOXC8	3224	1.001	0.983	1.225	0.001	0.722	0.018	HOXC8	homeobox C8
GABPB1	2553	1.081	0.22	1.233	0.002	0.793	0.017	GABPB1	GA binding protein transcription factor, beta subunit 1, 53kDa
IRF5	3663	0.875	0.028	1.21	0.002	0.806	4.40E-06	IRF5	interferon regulatory factor 5
ARNT	405	1.004	0.918	1.204	0.003	0.776	0.001	ARNT	aryl hydrocarbon receptor nuclear translocator
PPARD	5467	1.027	0.524	1.217	0.007	0.782	0.005	PPARD	peroxisome proliferative activated receptor, delta
FOXO3A	2309	1.134	1.12E-04	1.243	0.013	0.817	0.004	FOXO3A	Forkhead box O3A
RELB	5971	1.04	0.341	1.203	0.015	0.76	0.015	RELB	v-rel reticuloendotheliosis viral oncogene homolog B, nuclear factor of kapp
HKR3	3104	0.983	0.795	1.228	0.023	0.819	1.90E-04	HKR3	GLI-Kruppel family member HKR3
ERCC6	2074	0.925	0.071	1.293	0.026	0.829	0.006	ERCC6	excision repair cross-complementing rodent repair deficiency, complementat
HOXC13	3229	0.983	0.572	1.221	0.033	0.743	0.005	HOXC13	homeobox C13
TEF	7008	0.902	0.247	1.226	0.039	0.717	0.022	TEF	Thyrotrophic embryonic factor
ELF2	1998	0.951	0.048	1.223	0.046	0.802	0.037	ELF2	E74-like factor 2 (ets domain transcription factor)
GTF2H4	2968	1.149	0.098	1.348	0.047	0.777	2.56E-04	GTF2H4	general transcription factor IIH, polypeptide 4, 52kDa
HOXD1	3231	1.092	0.158	1.275	0.057	0.795	0.017	HOXD1	homeobox D1
FOXF2	2295	1.121	0.304	1.224	0.063	0.682	0.013	FOXF2	forkhead box F2
POU2F1	5451	0.943	0.192	1.276	0.078	0.823	0.036	POU2F1	POU domain, class 2, transcription factor 1
HOXB1	3211	0.907	0.077	1.24	0.093	0.815	0.021	HOXB1	homeobox B1
HOXA6	3203	0.952	0.584	1.316	0.11	0.764	0.008	HOXA6	Homeobox A6
HOXC5	3222	0.923	0.703	1.21	0.131	0.827	0.021	HOXC5	Homeobox C5
HOXD12	3238	0.913	0.049	1.182	0.003	0.769	0.002	HOXD12	homeobox D12
FHX	55810	1.043	0.375	1.181	0.066	0.77	0.001	FOXJ2	forkhead box J2
DRIL1	1820	0.901	0.32	1.173	0.312	0.715	0.002	ARID3A	AT rich interactive domain 3A (BRIGHT- like)
MSX2	4488	1.042	0.174	1.168	0.058	0.75	0.014	MSX2	msh homeobox homolog 2 (Drosophila)
PER3	8863	0.988	0.808	1.168	0.078	0.692	0.025	PER3	period homolog 3 (Drosophila)
HIRA	7290	1.023	0.547	1.164	0.004	0.79	0.019	HIRA	HIR histone cell cycle regulation defective homolog A (S. cerevisiae)
GFI1B	8328	0.907	0.027	1.162	0.074	0.789	5.82E-04	GFI1B	Growth factor independent 1B (potential regulator of CDKN1A, translocatec
GLI3	2737	1.072	0.204	1.157	0.09	0.821	0.042	GLI3	GLI-Kruppel family member GLI3 (Greig cephalopolysyndactyly syndrome
HNF3G	3171	1.159	0.081	1.156	0.045	0.782	0.017	FOXA3	forkhead box A3
GTF3C4	9329	0.918	0.09	1.152	0.27	0.778	0.01	GTF3C4	general transcription factor IIIC, polypeptide 4, 90kDa
E2F4	1874	1.128	0.07	1.15	0.009	0.7	0.015	E2F4	E2F transcription factor 4, p107/p130-binding
HAND2	9464	0.943	0.072	1.148	0.004	0.802	0.044	HAND2	heart and neural crest derivatives expressed 2
HIF1A	3091	1.013	0.784	1.148	0.073	0.749	0.032	HIF1A	hypoxia-inducible factor 1, alpha subunit (basic helix-loop-helix transcriptio
FLI1	2313	0.988	0.667	1.143	0.046	0.722	0.013	FLI1	Friend leukemia virus integration 1
RFXANK	8625	0.863	0.085	1.141	0.77	0.733	0.001	RFXANK	regulatory factor X-associated ankyrin-containing protein
GFI1	2672	1.009	0.842	1.135	0.265	0.781	3.56E-04	GFI1	growth factor independent 1
EZH2	2146	0.969	0.397	1.135	0.044	0.781	0.027	EZH2	enhancer of zeste homolog 2 (Drosophila)
HOXC6	3223	0.914	0.737	1.134	0.14	0.655	0.001	HOXC6	Homeobox C6
ERCC3	2071	1.045	0.151	1.128	0.004	0.807	0.033	ERCC3	excision repair cross-complementing rodent repair deficiency, complementat
E2F6	1876	0.986	0.629	1.128	0.024	0.77	0.014	E2F6	E2F transcription factor 6
HOXB7	3217	1.119	0.109	1.127	0.401	0.738	0.001	HOXB7	homeobox B7
ATF3	467	1.02	0.654	1.127	0.011	0.607	0.013	ATF3	activating transcription factor 3
ESRRG	2104	1.016	0.629	1.124	0.14	0.817	0.044	ESRRG	estrogen-related receptor gamma
E2F3	1871	1.074	0.197	1.124	0.117	0.785	0.002	E2F3	E2F transcription factor 3
EGR3	1960	0.921	0.008	1.124	0.139	0.784	2.51E-04	EGR3	early growth response 3
NEUROG1	4762	1.019	0.647	1.124	0.292	0.689	0.007	NEUROG1	neurogenin 1
EBF	1879	0.837	0.105	1.119	0.289	0.667	0.001	EBF	early B-cell factor
NR2C1	7181	0.993	0.859	1.116	0.409	0.668	0.022	NR2C1	nuclear receptor subfamily 2, group C, member 1

SNAPC3	6619	1.021	0.601	1.109	0.388	0.521	0.003	SNAPC3	small nuclear RNA activating complex, polypeptide 3, 50kDa
DSIPI	1831	1.154	0.387	1.106	0.075	0.813	0.003	TSC22D3	TSC22 domain family, member 3
GTF3C1	2975	0.874	0.136	1.092	0.121	0.682	0.005	GTF3C1	general transcription factor IIIC, polypeptide 1, alpha 220kDa
GABPA	2551	1.057	0.097	1.09	0.132	0.72	0.007	GABPA	GA binding protein transcription factor, alpha subunit 60kDa /// GA binding
ATF5	22809	0.964	0.187	1.09	0.182	0.524	0.011	ATF5	activating transcription factor 5
KLHL4	56062	1.01	0.892	1.089	0.247	0.775	0.04	KLHL4	Kelch-like 4 (Drosophila)
POU4F3	5459	1.029	0.477	1.089	0.397	0.613	0.001	POU4F3	POU domain, class 4, transcription factor 3
POU5F1	5460	0.998	0.967	1.088	0.56	0.733	0.002	POU5F1	POU domain, class 5, transcription factor 1 /// POU domain, class 5, transcri
RBL2	5934	1.057	0.413	1.088	0.109	0.678	0.04	RBL2	retinoblastoma-like 2 (p130)
LDB1	8861	1.005	0.931	1.083	0.063	0.808	0.023	LDB1	LIM domain binding 1
TBX10	347853	1.067	0.761	1.076	0.212	0.79	1.56E-05	TBX10	T-box 10
NR0B1	190	1.005	0.913	1.075	0.258	0.777	4.65E-04	NR0B1	nuclear receptor subfamily 0, group B, member 1
DLX3	1747	1.024	0.57	1.074	0.33	0.763	2.32E-04	DLX3	Distal-less homeobox 3
NFE2L2	4780	1.01	0.78	1.071	0.195	0.785	0.003	NFE2L2	nuclear factor (erythroid-derived 2)-like 2
HSPC189	51545	1.018	0.616	1.067	0.619	0.826	0.044	ZNF581	zinc finger protein 581
GCN5L2	2648	0.989	0.582	1.06	0.42	0.721	0.019	GCN5L2	GCN5 general control of amino-acid synthesis 5-like 2 (yeast)
PAX4	5078	0.86	0.001	1.057	0.18	0.807	0.023	PAX4	paired box gene 4
GTF2E1	2960	0.763	0.063	1.055	0.245	0.821	0.008	GTF2E1	general transcription factor IIE, polypeptide 1, alpha 56kDa
NFATC3	4775	1.021	0.406	1.055	0.189	0.778	0.005	NFATC3	nuclear factor of activated T-cells, cytoplasmic, calcineurin-dependent 3
ATF6	22926	1.028	0.554	1.053	0.343	0.814	0.048	ATF6	activating transcription factor 6
HOXB13	10481	0.907	0.111	1.052	0.305	0.793	0.003	HOXB13	Homeobox B13
EP300	2033	1.013	0.683	1.052	0.207	0.71	0.005	EP300	E1A binding protein p300
MEOX1	4222	1.007	0.826	1.044	0.177	0.804	0.006	MEOX1	mesenchyme homeobox 1
SHOX	6473	0.883	0.001	1.043	0.376	0.68	0.001	SHOX	short stature homeobox
IRX4	50805	0.929	0.096	1.04	0.363	0.649	0.003	IRX4	iroquois homeobox protein 4
NR2F6	2063	1.049	0.35	1.039	0.478	0.792	0.043	NR2F6	nuclear receptor subfamily 2, group F, member 6
NAB2	4665	1.039	0.399	1.039	0.374	0.766	0.008	NAB2	NGFI-A binding protein 2 (EGR1 binding protein 2)
PRDM12	59335	1.009	0.89	1.036	0.241	0.809	0.013	PRDM12	PR domain containing 12
ZNF205	7755	0.983	0.691	1.036	0.27	0.803	0.041	ZNF205	Zinc finger protein 205 /// zinc finger protein 205
CBX1	10951	0.977	0.494	1.036	0.754	0.595	0.006	CBX1	chromobox homolog 1 (HP1 beta homolog Drosophila)
HOXA2	3199	1.086	0.063	1.035	0.41	0.729	0.002	HOXA2	homeobox A2
SREBF2	6721	0.835	0.228	1.033	0.598	0.703	0.004	SREBF2	sterol regulatory element binding transcription factor 2
SURB7	9412	1.006	0.798	1.033	0.415	0.654	0.021	SURB7	SRB7 suppressor of RNA polymerase B homolog (yeast)
NRIP1	8204	1.019	0.645	1.031	0.443	0.802	4.46E-05	NRIP1	nuclear receptor interacting protein 1
CHD4	1108	0.911	0.224	1.03	0.293	0.582	0.005	CHD4	Chromodomain helicase DNA binding protein 4
RNF24	11237	1.072	0.166	1.029	0.736	0.718	0.009	RNF24	Ring finger protein 24
CREBBP	1387	0.882	0.258	1.028	0.49	0.822	0.033	CREBBP	CREB binding protein (Rubinstein-Taybi syndrome)
ICSBP1	3394	1.025	0.293	1.026	0.479	0.766	4.55E-05	IRF8	interferon regulatory factor 8 /// interferon regulatory factor 8
CREM	1390	0.976	0.321	1.025	0.802	0.652	0.017	CREM	cAMP responsive element modulator
GTF3C2	2976	1.003	0.872	1.023	0.628	0.819	0.002	GTF3C2	General transcription factor IIIC, polypeptide 2, beta 110kDa
BARX2	8538	1.103	0.302	1.021	0.528	0.728	0.005	BARX2	BarH-like homeobox 2
TAF2C2	6875	1.048	0.422	1.018	0.727	0.701	0.027	TAF4B	TAF4b RNA polymerase II, TATA box binding protein (TBP)-associated fa
RBL1	5933	0.983	0.665	1.017	0.598	0.819	0.001	RBL1	retinoblastoma-like 1 (p107)
ID3	3399	0.944	0.261	1.015	0.721	0.814	0.009	ID3	inhibitor of DNA binding 3, dominant negative helix-loop-helix protein
TCF3	6929	1.015	0.718	1.015	0.779	0.758	3.60E-04	TCF3	transcription factor 3 (E2A immunoglobulin enhancer binding factors E12/E
HNF3A	3169	0.901	0.074	1.014	0.818	0.75	0.004	FOXA1	Forkhead box A1
NR5A1	2516	0.979	0.258	1.011	0.774	0.814	0.012	NR5A1	nuclear receptor subfamily 5, group A, member 1
IGHMBP2	3508	1	0.999	1.009	0.722	0.749	4.02E-04	IGHMBP2	immunoglobulin mu binding protein 2
SPIB	6689	1.107	0.326	1.006	0.859	0.809	0.007	SPIB	Spi-B transcription factor (Spi-1/PU.1 related) /// Spi-B transcription factor (
EZH1	2145	0.915	0.233	1.006	0.909	0.729	0.001	EZH1	Enhancer of zeste homolog 1 (Drosophila)
AHR	196	1.023	0.335	1.006	0.911	0.691	0.013	AHR	aryl hydrocarbon receptor

BCL6	604	0.997	0.919	1.002	0.958	0.82	0.015	BCL6	B-cell CLL/lymphoma 6 (zinc finger protein 51) /// B-cell CLL/lymphoma 6
TAF2C1	6874	0.945	0.169	1.001	0.986	0.791	0.005	TAF4	TAF4 RNA polymerase II, TATA box binding protein (TBP)-associated fac
SPI1	6688	1.056	0.46	1.001	0.989	0.717	1.43E-04	SPI1	spleen focus forming virus (SFFV) proviral integration oncogene spi1
POU3F4	5456	0.964	0.463	1	0.991	0.806	0.004	POU3F4	POU domain, class 3, transcription factor 4
KIAA0441	9841	0.929	0.663	1	0.992	0.792	4.95E-04	ZBTB24	zinc finger and BTB domain containing 24
TBX6	6911	1.099	0.248	1	0.988	0.785	0.012	TBX6	T-box 6
BRPF3	27154	1.06	0.22	0.997	0.949	0.793	0.004	BRPF3	bromodomain and PHD finger containing, 3
EN2	2020	0.916	0.379	0.994	0.901	0.758	4.27E-05	EN2	Engrailed homolog 2
TBP	6908	1.159	0.279	0.994	0.918	0.685	0.003	TBP	TATA box binding protein /// spinocerebellar ataxia 17
MGC2508	79177	0.994	0.883	0.989	0.87	0.717	0.01	ZNF576	Zinc finger protein 576
SUPT3H	8464	0.991	0.844	0.988	0.511	0.784	0.004	SUPT3H	suppressor of Ty 3 homolog (S. cerevisiae)
GCN5L1	2647	1.037	0.41	0.987	0.868	0.759	2.62E-04	BLOC1S1	Biogenesis of lysosome-related organelles complex-1, subunit 1
SIM2	6493	0.983	0.669	0.985	0.701	0.817	0.022	SIM2	single-minded homolog 2 (Drosophila)
ATF7	11016	1.243	0.103	0.984	0.824	0.65	0.006	ATF7	Activating transcription factor 7
FOXF1	2294	0.974	0.458	0.981	0.765	0.758	0.005	FOXF1	forkhead box F1
KLF15	28999	0.997	0.953	0.979	0.65	0.79	0.016	KLF15	Kruppel-like factor 15
STAT6	6778	0.995	0.769	0.971	0.633	0.772	0.019	STAT6	signal transducer and activator of transcription 6, interleukin-4 induced
EMX2	2018	0.916	0.118	0.971	0.308	0.734	0.016	EMX2	empty spiracles homolog 2 (Drosophila)
FOXM1	2305	0.992	0.807	0.962	0.32	0.782	0.005	FOXM1	forkhead box M1
CSDA	8531	1.126	0.227	0.961	0.342	0.68	0.001	CSDA	cold shock domain protein A
FLJ20321	54897	0.897	0.36	0.957	0.408	0.817	0.002	CASZ1	Castor homolog 1, zinc finger (Drosophila)
SIX2	10736	0.901	0.002	0.957	0.254	0.67	3.94E-04	SIX2	sine oculis homeobox homolog 2 (Drosophila)
H_GS165L15	9586	0.99	0.843	0.953	0.521	0.79	0.001	CREB5	cAMP responsive element binding protein 5
CTCF	10664	0.968	0.323	0.951	0.266	0.644	0.003	CTCF	CCCTC-binding factor (zinc finger protein)
ILF1	3607	1.057	0.393	0.947	0.258	0.672	0.002	FOXK2	Forkhead box K2
TFAP2A	7020	0.897	0.298	0.944	0.119	0.808	0.015	TFAP2A	Transcription factor AP-2 alpha (activating enhancer binding protein 2 alpha
TFAP4	7023	0.896	0.027	0.94	0.234	0.725	0.003	TFAP4	transcription factor AP-4 (activating enhancer binding protein 4)
RBPSUHL	11317	0.846	0.08	0.939	0.189	0.678	0.008	RBPSUHL	recombining binding protein suppressor of hairless (Drosophila)-like
BRF2	55290	1.079	0.468	0.938	0.095	0.525	0.045	BRF2	BRF2, subunit of RNA polymerase III transcription initiation factor, BRF1-l
SUPT4H1	6827	0.92	0.067	0.936	0.547	0.614	0.047	SUPT4H1	suppressor of Ty 4 homolog 1 (S. cerevisiae)
DNAJ	10294	1.054	0.319	0.933	0.1	0.74	0.001	DNAJA2	DnaJ (Hsp40) homolog, subfamily A, member 2
NR1H3	10062	1.173	0.09	0.927	0.164	0.772	0.009	NR1H3	nuclear receptor subfamily 1, group H, member 3
POU2AF1	5450	0.941	0.328	0.926	0.193	0.83	0.006	POU2AF1	POU domain, class 2, associating factor 1
GLIS2	84662	1.149	0.651	0.925	0.193	0.819	0.014	GLIS2	GLIS family zinc finger 2
SIX1	6495	0.991	0.766	0.924	0.434	0.748	2.14E-06	SIX1	sine oculis homeobox homolog 1 (Drosophila)
ATF2	1386	1.133	0.298	0.923	0.479	0.654	0.023	ATF2	activating transcription factor 2
HOXA9	3205	1.128	0.191	0.921	0.907	0.757	0.038	HOXA9	homeobox A9
CREB1	1385	1.055	0.404	0.918	0.175	0.707	0.004	CREB1	cAMP responsive element binding protein 1
HOXA13	3209	0.918	0.124	0.916	0.936	0.821	0.015	HOXA13	homeobox A13
POU6F1	5463	0.729	0.24	0.916	0.26	0.786	0.051	POU6F1	POU domain, class 6, transcription factor 1
DLX6	1750	0.978	0.63	0.913	0.041	0.708	0.001	DLX6	distal-less homeobox 6
PROX1	5629	1.02	0.64	0.912	0.691	0.784	0.002	PROX1	prospero-related homeobox 1
SRF	6722	0.947	0.212	0.9	0.037	0.723	0.011	SRF	Serum response factor (c-fos serum response element-binding transcription f
CBX4	8535	0.985	0.634	0.884	0.003	0.716	0.004	CBX4	chromobox homolog 4 (Pc class homolog, Drosophila)
IRF1	3659	0.942	0.694	0.88	0.461	0.622	0.009	IRF1	interferon regulatory factor 1
NCOA3	8202	0.957	0.301	0.878	0.077	0.818	0.021	NCOA3	Nuclear receptor coactivator 3
FHL2	2274	0.973	0.763	0.868	0.101	0.829	0.003	FHL2	four and a half LIM domains 2
HRIHFB2436	24149	1.113	0.069	0.864	0.062	0.752	0.022	ZNF318	Zinc finger protein 318
BACH1	571	1.015	0.799	0.861	0.201	0.618	0.005	BACH1	BTB and CNC homology 1, basic leucine zipper transcription factor 1
NFX1	4799	1.04	0.328	0.857	0.373	0.726	0.004	NFX1	nuclear transcription factor, X-box binding 1
SATB1	6304	1.021	0.624	0.852	0.155	0.805	0.01	SATB1	special AT-rich sequence binding protein 1 (binds to nuclear matrix/scaffold

MYBL2	4605	1.131	0.17	0.844	0.234	0.726	0.004	MYBL2	v-myb myeloblastosis viral oncogene homolog (avian)-like 2
TAF2H	6881	0.774	0.19	0.652	4.26E-04	0.641	0.003	TAF10	TAF10 RNA polymerase II, TATA box binding protein (TBP)-associated fa
LAF4	3899	1.109	0.708	0.594	0.003	0.706	0.025	AFF3	AF4/FMR2 family, member 3
ATBF1	463	1.142	0.186	0.83	0.02	0.593	0.001	ATBF1	AT-binding transcription factor 1
LDOC1	23641	0.939	0.495	0.747	0.021	0.748	0.006	LDOC1	Leucine zipper, down-regulated in cancer 1
SIX3	6496	0.997	0.949	0.832	0.025	0.651	0.002	SIX3	sine oculis homeobox homolog 3 (Drosophila)
FKHL18	2307	0.941	0.381	0.81	0.025	0.721	1.51E-05	FKHL18	forkhead-like 18 (Drosophila)
KLF5	688	0.939	0.439	0.832	0.027	0.827	0.021	KLF5	Kruppel-like factor 5 (intestinal)
KIAA0130	9862	0.71	0.074	0.755	0.031	0.671	0.051	THRAP4	Thyroid hormone receptor associated protein 4
RORC	6097	1.042	0.768	0.726	0.031	0.603	0.002	RORC	RAR-related orphan receptor C
JUND	3727	0.929	0.58	0.71	0.041	0.734	0.009	JUND	jun D proto-oncogene
ZNF38	7589	0.702	0.08	0.598	0.045	0.727	0.015	ZNF38	Zinc finger protein 38
NR1I3	9970	0.881	0.351	0.691	0.05	0.803	0.001	NR1I3	Nuclear receptor subfamily 1, group I, member 3
LIM	10611	1.107	0.006	0.778	0.078	0.806	0.045	PDLIM5	PDZ and LIM domain 5
FHL1	2273	0.825	0.087	0.802	0.13	0.736	0.009	FHL1	four and a half LIM domains 1
PRDM15	63977	0.926	0.556	0.83	0.327	0.68	4.77E-04	PRDM15	PR domain containing 15 /// similar to PR domain containing 15
NFE2L1	4779	0.704	0.048	1.156	0.315	0.761	0.017	NFE2L1	nuclear factor (erythroid-derived 2)-like 1
GTF2F1	2962	0.785	0.018	1.019	0.759	0.586	6.01E-05	GTF2F1	general transcription factor IIF, polypeptide 1, 74kDa
CITED1	4435	0.617	0.003	0.954	0.263	0.718	0.002	CITED1	Cbp/p300-interacting transactivator, with Glu/Asp-rich carboxy-terminal do
ONECUT2	9480	0.839	0.002	0.899	0.009	0.77	0.008	ONECUT2	one cut domain, family member 2
CL469780	27246	0.814	0.007	0.863	0.049	0.822	0.004	ZNF364	zinc finger protein 364
NR4A1	3164	0.789	0.005	0.855	0.155	0.77	0.021	NR4A1	nuclear receptor subfamily 4, group A, member 1
SIX4	51804	0.621	0.009	0.681	4.65E-04	0.624	0.002	SIX4	Sine oculis homeobox homolog 4 (Drosophila)
IRF2	3660	0.8	0.021	0.754	0.001	0.521	0.01	IRF2	Interferon regulatory factor 2
ZNF10	7556	0.324	0.003	0.271	0.002	0.604	0.028	ZNF10	zinc finger protein 10
CIAO1	9391	0.622	0.023	0.458	0.003	0.691	0.034	CIAO1	Cytosolic iron-sulfur protein assembly 1 homolog (S. cerevisiae)
MYF5	4617	0.656	0.017	0.538	0.007	0.802	0.053	MYF5	myogenic factor 5
LMO1	4004	0.696	0.012	0.773	0.01	0.681	0.003	LMO1	LIM domain only 1 (rhombotin 1)
GTF2E2	2961	0.75	0.012	0.781	0.161	0.837	0.032	GTF2E2	General transcription factor IIE, polypeptide 2, beta 34kDa

ctor, 100kDa

nding factor homolog

110kDa

ole protein PC4

ription factor 2

siae)

oint 9

100)

:ogene homolog, avian)

actor, 68kDa

:er factor 2B)

Da (Mot1 homolog, S. cerevisia
ated factor, 65kDa

-like 1

pa light polypeptide gene enhan

ation group 6

ed in CML)
e)

on factor)

ation group 3 (xeroderma pigme

g protein transcription factor, al

:iption factor 1 pseudogene 1 ///

actor, 105kDa

Ξ47)

(Spi-1/PU.1 related)

6 (zinc finger protein 51)
ctor, 135kDa

a)

-like

factor)

d-associating DNA's)

actor, 30kDa

omain, 1
