# Supplementary material for: Large Scale Gene Expression Profiles of Regenerating Inner Ear Sensory Epithelia
Source: PLoS One. 2007 Jun 13;2(6):e525. doi: 10.1371/journal.pone.0000525 (PMC1888727; doi:10.1371/journal.pone.0000525)
Supplement: Table S7 — Cochlea Laser Differentially Expressed Genes (386 genes total). This listing shows all genes that exhibited>1.2-fold changes in expression, irrespective of P-value. For p-value filtered data see Table S8. (0.53 MB DOC) [file pone.0000525.s008.doc]

Supplemental Table S7

|  | | **30min** | | | | **1hr** | | | | **2hr** | | | | **3hr** | |  |  | |
| --- | --- | --- | --- | --- | --- | --- | --- | --- | --- | --- | --- | --- | --- | --- | --- | --- | --- | --- |
| **Gene ID** | | **Fold change** | | **P-value** | | **Fold Change** | | **P-value** | | **Fold Change** | | **P-value** | | **Fold Change** | | **P-value** | **Notes/Description** | |
| AF093680 | 1.279 | | 0.014 | | 1.011 | | 0.826 | | 0.908 | | 0.287 | | 0.952 | | 0.331 | | | similar to mouse Glt3 or D. malanogaster transcription factor IIB |
| AF5Q31 | 1.377 | | 0.425 | | 1.133 | | 0.470 | | 0.992 | | 0.954 | | 1.289 | | 0.008 | | | ALL1 fused gene from 5q31 |
| ALX3 | 1.011 | | 0.889 | | 1.206 | | 0.202 | | 1.019 | | 0.376 | | 0.943 | | 0.287 | | | aristaless-like homeobox 3 |
| ARC | 0.812 | | 0.036 | | 0.885 | | 0.163 | | 1.078 | | 0.448 | | 1.148 | | 0.009 | | | activity-regulated cytoskeleton-associated protein |
| ARNTL | 1.276 | | 0.046 | | 1.059 | | 0.572 | | 0.813 | | 0.151 | | 1.018 | | 0.696 | | | aryl hydrocarbon receptor nuclear translocator-like |
| ATF5 | 0.767 | | 0.026 | | 0.704 | | 0.113 | | 0.918 | | 0.352 | | 0.953 | | 0.658 | | | activating transcription factor 5 |
| ATRX | 0.783 | | 0.314 | | 1.000 | | 0.998 | | 1.178 | | 0.142 | | 1.050 | | 0.300 | | | alpha thalassemia/mental retardation syndrome X-linked (RAD54 (S. cerevisiae) homolog) |
| BACH2 | 0.990 | | 0.900 | | 1.005 | | 0.861 | | 0.985 | | 0.811 | | 0.770 | | 0.116 | | | BTB and CNC homology 1, basic leucine zipper transcription factor 2 |
| BAZ2B | 1.200 | | 0.020 | | 0.873 | | 0.042 | | 1.120 | | 0.401 | | 1.058 | | 0.343 | | | bromodomain adjacent to zinc finger domain, 2B |
| BCL11A | 1.003 | | 0.959 | | 0.690 | | 0.166 | | 1.088 | | 0.382 | | 1.197 | | 0.005 | | | B-cell CLL/lymphoma 11A (zinc finger protein) |
| BCL11B | 1.225 | | 0.046 | | 0.873 | | 0.316 | | 0.950 | | 0.460 | | 0.989 | | 0.787 | | | B-cell CLL/lymphoma 11B (zinc finger protein) |
| BHLHB2 | 0.721 | | 0.053 | | 0.903 | | 0.373 | | 0.799 | | 0.132 | | 1.209 | | 0.141 | | | basic helix-loop-helix domain containing, class B, 2 |
| BHLHB3 | 0.865 | | 0.263 | | 0.805 | | 0.069 | | 0.978 | | 0.696 | | 1.222 | | 0.016 | | | basic helix-loop-helix domain containing, class B, 3 |
| BLZF1 | 0.763 | | 0.056 | | 0.737 | | 0.038 | | 0.898 | | 0.172 | | 1.170 | | 0.004 | | | basic leucine zipper nuclear factor 1 (JEM-1) |
| BMI1 | 1.017 | | 0.826 | | 0.916 | | 0.317 | | 0.691 | | 0.148 | | 0.896 | | 0.216 | | | murine leukemia viral (bmi-1) oncogene homolog |
| BRF2 | 1.461 | | 0.049 | | 0.770 | | 0.296 | | 0.895 | | 0.458 | | 1.033 | | 0.776 | | | zinc finger protein 36, C3H type-like 2 |
| BRPF1 | 0.799 | | 0.076 | | 0.923 | | 0.379 | | 0.918 | | 0.495 | | 1.058 | | 0.612 | | | bromodomain and PHD finger containing, 1 |
| BRPF3 | 1.241 | | 0.129 | | 1.023 | | 0.811 | | 1.378 | | 0.106 | | 1.021 | | 0.698 | | | bromodomain and PHD finger containing, 3 |
| BTF3L1 | 1.005 | | 0.981 | | 1.055 | | 0.505 | | 1.357 | | 0.177 | | 0.971 | | 0.448 | | | basic transcription factor 3, like 1 |
| C11orf9 | 0.853 | | 0.085 | | 0.829 | | 0.104 | | 0.979 | | 0.534 | | 1.097 | | 0.072 | | | chromosome 11open reading frame 9 |
| C21orf18 | 0.775 | | 0.132 | | 0.877 | | 0.179 | | 0.947 | | 0.587 | | 1.131 | | 0.139 | | | chromosome 21 open reading frame 18 |
| CART1 | 0.879 | | 0.151 | | 0.854 | | 0.322 | | 0.775 | | 0.102 | | 1.149 | | 0.185 | | | cartilage paired-class homeoprotein 1 |
| CBFA2T3 | 1.083 | | 0.488 | | 1.114 | | 0.330 | | 0.924 | | 0.247 | | 1.226 | | 0.007 | | | core-binding factor, runt domain, alpha subunit 2; translocated to, 3 |
| CBX8 | 0.997 | | 0.979 | | 0.960 | | 0.552 | | 0.788 | | 0.022 | | 1.187 | | 0.105 | | | chromobox homolog 8 (Drosophila Pc class) |
| CDX2 | 0.943 | | 0.436 | | 0.997 | | 0.962 | | 1.115 | | 0.460 | | 1.226 | | 0.001 | | | caudal-type homeobox transcription factor 2 |
| CDX4 | 1.410 | | 0.021 | | 0.945 | | 0.540 | | 1.015 | | 0.858 | | 0.912 | | 0.086 | | | caudal-type homeobox transcription factor 4 |
| CERD4 | 0.827 | | 0.010 | | 0.853 | | 0.014 | | 1.005 | | 0.912 | | 1.060 | | 0.275 | | | D4, zinc and double PHD fingers, family 3 |
| CEZANNE | 0.782 | | 0.060 | | 0.916 | | 0.307 | | 0.866 | | 0.077 | | 1.240 | | 0.020 | | | cellular zinc finger anti-NF-kappaB Cezanne |
| CHD3 | 1.530 | | 0.213 | | 0.961 | | 0.413 | | 0.963 | | 0.546 | | 1.209 | | 0.002 | | | chromodomain helicase DNA binding protein 3 |
| CHD4 | 1.005 | | 0.919 | | 0.876 | | 0.156 | | 1.403 | | 0.157 | | 0.858 | | 0.017 | | | chromodomain helicase DNA binding protein 4 |
| CIR | 0.910 | | 0.329 | | 1.749 | | 0.273 | | 0.681 | | 0.291 | | 1.013 | | 0.874 | | | CBF1 interacting corepressor |
| CITED2 | 1.352 | | 0.018 | | 0.974 | | 0.795 | | 1.130 | | 0.207 | | 1.315 | | 0.017 | | | Cbp/p300-interacting transactivator, with Glu/Asp-rich carboxy-terminal domain, 2 |
| CLOCK | 1.597 | | 0.286 | | 1.292 | | 0.050 | | 0.929 | | 0.514 | | 0.865 | | 0.144 | | | clock (mouse) homolog |
| CNOT3 | 0.793 | | 0.143 | | 0.820 | | 0.165 | | 0.911 | | 0.287 | | 1.031 | | 0.415 | | | CCR4-NOT transcription complex, subunit 3 |
| CNOT8 | 0.964 | | 0.269 | | 0.926 | | 0.051 | | 0.808 | | 0.598 | | 1.020 | | 0.732 | | | CCR4-NOT transcription complex, subunit 8 |
| COPEB | 1.121 | | 0.168 | | 1.035 | | 0.716 | | 1.074 | | 0.550 | | 0.821 | | 0.004 | | | core promoter element binding protein |
| COPS5 | 0.868 | | 0.386 | | 1.201 | | 0.119 | | 1.130 | | 0.413 | | 1.050 | | 0.176 | | | COP9 constitutive photomorphogenic homolog subunit 5 (Arabidopsis) |
| CREB1 | 1.200 | | 0.057 | | 0.992 | | 0.954 | | 1.088 | | 0.621 | | 1.134 | | 0.183 | | | cAMP responsive element binding protein-like 1 |
| CRIP1 | 1.057 | | 0.549 | | 0.893 | | 0.193 | | 1.277 | | 0.144 | | 0.927 | | 0.146 | | | cysteine-rich protein 1 (intestinal) |
| CROC4 | 0.623 | | 0.234 | | 0.994 | | 0.957 | | 1.249 | | 0.019 | | 0.825 | | 0.103 | | | transcriptional activator of the c-fos promoter |
| CRSP6 | 1.339 | | 0.160 | | 0.940 | | 0.555 | | 1.095 | | 0.416 | | 0.903 | | 0.061 | | | cofactor required for Sp1 transcriptional activation, subunit 6 (77kD) |
| CRSP9 | 0.982 | | 0.911 | | 0.845 | | 0.011 | | 1.229 | | 0.093 | | 1.003 | | 0.942 | | | cofactor required for Sp1 transcriptional activation, subunit 9, 33kD |
| CRX | 0.830 | | 0.313 | | 1.315 | | 0.418 | | 0.618 | | 0.223 | | 0.858 | | 0.151 | | | cone-rod homeobox |
| CSEN | 0.897 | | 0.234 | | 1.104 | | 0.508 | | 0.985 | | 0.917 | | 0.801 | | 0.007 | | | Calsenilin, presenilin-binding protein, EF hand transcription factor |
| CTCF | 0.821 | | 0.109 | | 0.990 | | 0.929 | | 1.020 | | 0.843 | | 0.854 | | 0.054 | | | CCCTC-binding factor (zinc finger protein) |
| CUTL1 | 0.669 | | 0.298 | | 1.239 | | 0.217 | | 1.035 | | 0.695 | | 0.802 | | 2.61E-04 | | | cut (Drosophila)-like 1 (CCAAT displacement protein) |
| CXorf6 | 0.764 | | 0.219 | | 0.926 | | 0.714 | | 0.906 | | 0.258 | | 1.234 | | 0.005 | | | chromosome X open reading frame 6 |
| DDIT3 | 0.821 | | 0.222 | | 1.009 | | 0.953 | | 0.917 | | 0.177 | | 0.937 | | 0.385 | | | DNA-damage-inducible transcript 3 |
| DFKZP434E026 | 1.407 | | 0.002 | | 1.064 | | 0.586 | | 1.411 | | 0.074 | | 1.090 | | 0.220 | | | egl nine homolog 2 (C. elegans) (EGLN2) |
| DKFZP434B0335 | 0.788 | | 0.019 | | 1.103 | | 0.586 | | 0.874 | | 0.384 | | 0.852 | | 0.020 | | | DKFZP434B0335 protein |
| DKFZP434P1750 | 1.371 | | 0.008 | | 1.111 | | 0.682 | | 1.027 | | 0.911 | | 0.878 | | 0.162 | | | TBC1 domain family, member 10B (TBC1D10B) |
| DKFZp547H236 | 0.957 | | 0.767 | | 0.965 | | 0.734 | | 1.161 | | 0.254 | | 1.497 | | 0.032 | | | myeloid ecotropic viral integration site 1 homolog 3 (MEIS3) |
| DKFZp762K2015 | 1.061 | | 0.567 | | 1.005 | | 0.962 | | 0.830 | | 0.069 | | 1.011 | | 0.803 | | | SWI/SNF-related, matrix-associated actin-dependent regulator of chromatin, subfamily a, containing DEAD/H box 1 (SMARCAD1) |
| DLX5 | 0.968 | | 0.644 | | 0.951 | | 0.413 | | 1.257 | | 0.032 | | 0.854 | | 0.027 | | | distal-less homeobox 5 |
| DMRT2 | 1.185 | | 0.817 | | 1.300 | | 0.167 | | 1.125 | | 0.287 | | 0.921 | | 0.269 | | | doublesex and mab-3 related transcription factor 2 |
| DSIPI | 0.667 | | 0.068 | | 0.989 | | 0.936 | | 1.299 | | 0.294 | | 0.895 | | 0.217 | | | delta sleep inducing peptide, immunoreactor |
| DUX2 | 0.798 | | 0.426 | | 0.814 | | 0.241 | | 1.045 | | 0.540 | | 0.971 | | 0.255 | | | double homeobox 2 |
| DUX4 | 0.995 | | 0.940 | | 0.991 | | 0.882 | | 1.218 | | 0.124 | | 0.967 | | 0.393 | | | double homeobox, 4 |
| E2F2 | 0.653 | | 0.072 | | 1.177 | | 0.288 | | 1.083 | | 0.582 | | 0.865 | | 0.013 | | | E2F transcription factor 2 |
| E2F5 | 1.274 | | 0.208 | | 0.986 | | 0.829 | | 0.944 | | 0.348 | | 0.883 | | 0.091 | | | E2F transcription factor 5, p130-binding |
| E2F6 | 1.054 | | 0.339 | | 1.166 | | 0.935 | | 1.235 | | 0.117 | | 0.950 | | 0.436 | | | E2F transcription factor 6 |
| E4F1 | 1.162 | | 0.014 | | 0.880 | | 0.101 | | 1.219 | | 0.227 | | 1.036 | | 0.421 | | | E4F transcription factor 1 |
| EBF | 1.365 | | 0.091 | | 1.137 | | 0.379 | | 1.410 | | 0.139 | | 0.952 | | 0.438 | | | early B-cell factor |
| EGR1 | 1.775 | | 0.001 | | 0.992 | | 0.945 | | 0.947 | | 0.358 | | 1.048 | | 0.651 | | | early growth response 1 |
| EHF | 1.207 | | 0.102 | | 0.815 | | 0.242 | | 1.056 | | 0.424 | | 0.965 | | 0.396 | | | ets homologous factor |
| ELF1 | 1.034 | | 0.575 | | 0.945 | | 0.541 | | 1.317 | | 0.147 | | 1.036 | | 0.299 | | | E74-like factor 1 (ets domain transcription factor) |
| ELK1 | 1.209 | | 0.022 | | 0.980 | | 0.693 | | 1.025 | | 0.756 | | 0.999 | | 0.986 | | | ELK1, member of ETS oncogene family |
| ELK3 | 0.923 | | 0.244 | | 0.991 | | 0.916 | | 0.794 | | 0.005 | | 0.901 | | 0.116 | | | ELK3, ETS-domain protein (SRF accessory protein 2) |
| ELK4 | 1.271 | | 0.034 | | 1.052 | | 0.603 | | 0.860 | | 0.025 | | 1.066 | | 0.031 | | | ELK4, ETS-domain protein (SRF accessory protein 1) |
| EP300 | 1.439 | | 0.019 | | 1.069 | | 0.523 | | 0.873 | | 0.660 | | 0.870 | | 0.090 | | | E1A binding protein p300 |
| EPAS1 | 0.626 | | 0.039 | | 0.869 | | 0.089 | | 0.766 | | 0.153 | | 1.299 | | 0.009 | | | endothelial PAS domain protein 1 |
| ERCC3 | 0.866 | | 0.140 | | 1.042 | | 0.587 | | 1.227 | | 0.442 | | 1.052 | | 0.413 | | | excision repair cross-complementing rodent repair deficiency, complementation group 3 |
| ERCC6 | 1.271 | | 0.274 | | 0.899 | | 0.319 | | 0.885 | | 0.842 | | 1.078 | | 0.247 | | | excision repair cross-complementing rodent repair deficiency, complementation group 6 |
| ERG | 0.918 | | 0.360 | | 1.141 | | 0.093 | | 1.257 | | 0.061 | | 1.109 | | 0.244 | | | v-ets avian erythroblastosis virus E26 oncogene related |
| ESR1 | 0.724 | | 0.095 | | 0.905 | | 0.504 | | 0.970 | | 0.813 | | 1.820 | | 0.001 | | | estrogen receptor 1 |
| ESRRA | 0.715 | | 0.108 | | 0.791 | | 0.112 | | 0.983 | | 0.684 | | 1.012 | | 0.750 | | | estrogen-related receptor alpha |
| ESRRG | 0.855 | | 0.164 | | 0.857 | | 0.140 | | 1.281 | | 0.064 | | 1.071 | | 0.233 | | | estrogen-related receptor gamma |
| ETV1 | 1.419 | | 0.286 | | 1.035 | | 0.895 | | 1.067 | | 0.833 | | 0.540 | | 0.005 | | | ets variant gene 1 |
| EZH2 | 1.203 | | 0.052 | | 1.016 | | 0.830 | | 1.119 | | 0.031 | | 0.991 | | 0.866 | | | enhancer of zeste homolog 2 |
| FKHL18 | 0.927 | | 0.717 | | 1.099 | | 0.732 | | 1.184 | | 0.581 | | 0.641 | | 0.007 | | | forkhead (Drosophila)-like 18 |
| FLJ10759 | 1.260 | | 5.46E-05 | | 1.042 | | 0.450 | | 0.926 | | 0.357 | | 1.143 | | 0.012 | | | hypothetical protein FLJ10759 |
| FLJ11186 | 0.562 | | 0.136 | | 0.588 | | 0.294 | | 0.855 | | 0.345 | | 2.043 | | 0.007 | | | chromosome 14 open reading frame 106 |
| FLJ11191 | 0.853 | | 0.151 | | 0.884 | | 0.496 | | 0.775 | | 0.156 | | 1.236 | | 0.024 | | | zinc finger protein ZNF415 |
| FLJ12457 | 0.767 | | 0.131 | | 0.992 | | 0.928 | | 0.956 | | 0.734 | | 1.001 | | 0.950 | | | RNA-binding protein LIN-28 |
| FLJ12517 | 1.333 | | 0.052 | | 0.996 | | 0.920 | | 1.239 | | 0.026 | | 0.953 | | 0.177 | | | jumonji domain containing 4 (JMJD4) |
| FLJ12606 | 0.897 | | 0.249 | | 0.824 | | 0.187 | | 0.915 | | 0.128 | | 0.959 | | 0.219 | | | zinc finger proteins ZNF669 or ZNF670 |
| FLJ12644 | 1.247 | | 0.078 | | 0.969 | | 0.681 | | 1.059 | | 0.412 | | 1.037 | | 0.312 | | | zinc finger protein ZNF649 |
| FLJ12827 | 0.997 | | 0.954 | | 0.837 | | 0.005 | | 1.314 | | 0.264 | | 0.980 | | 0.713 | | | zinc finger protein ZNF408 |
| FLJ13222 | 1.213 | | 0.011 | | 1.142 | | 0.092 | | 1.045 | | 0.246 | | 0.796 | | 0.003 | | | testis expressed sequence 27 (TEX27) |
| FLJ13659 | 1.223 | | 0.017 | | 0.839 | | 0.023 | | 1.213 | | 0.254 | | 1.067 | | 0.222 | | | zinc finger protein ZNF430 |
| FLJ20321 | 1.188 | | 0.244 | | 0.975 | | 0.546 | | 1.202 | | 0.114 | | 0.874 | | 0.085 | | | castor homolog 1, zinc finger (Drosophila) (CASZ1) |
| FLJ21603 | 0.714 | | 0.065 | | 1.111 | | 0.342 | | 1.016 | | 0.873 | | 1.317 | | 0.006 | | | zinc finger protein ZNF552 |
| FLJ22301 | 1.357 | | 0.039 | | 1.035 | | 0.581 | | 1.183 | | 0.332 | | 1.059 | | 0.126 | | | hypothetical protein FLJ22301 |
| FLJ22332 | 0.885 | | 0.216 | | 0.848 | | 0.084 | | 0.815 | | 0.133 | | 1.271 | | 6.28E-06 | | | zinc finger and BTB domain containing 38 (ZBTB38) |
| FOG2 | 1.061 | | 0.628 | | 1.339 | | 0.067 | | 0.850 | | 0.723 | | 1.385 | | 0.011 | | | friend of GATA2 |
| FOS | 1.501 | | 1.93E-04 | | 1.132 | | 0.616 | | 1.079 | | 0.368 | | 1.136 | | 0.167 | | | v-fos FBJ murine osteosarcoma viral oncogene homolog |
| FOXB1 | 0.970 | | 0.640 | | 0.820 | | 0.068 | | 1.204 | | 0.359 | | 0.936 | | 0.281 | | | forkhead box B1 |
| FOXC2 | 1.499 | | 0.022 | | 1.219 | | 0.266 | | 1.153 | | 0.177 | | 0.971 | | 0.568 | | | forkhead box C2 (MFH-1, mesenchyme forkhead 1) |
| FOXE1 | 0.979 | | 0.851 | | 0.997 | | 0.974 | | 1.255 | | 0.308 | | 0.890 | | 0.010 | | | forkhead box E1 (thyroid transcription factor 2) |
| FOXH1 | 0.915 | | 0.411 | | 0.916 | | 0.362 | | 0.936 | | 0.342 | | 1.282 | | 0.001 | | | forkhead box H1 |
| FOXI1 | 0.942 | | 0.210 | | 0.999 | | 0.992 | | 0.775 | | 0.105 | | 0.909 | | 0.106 | | | forkhead box I1 |
| FOXL2 | 1.161 | | 0.192 | | 1.021 | | 0.864 | | 1.260 | | 0.255 | | 0.959 | | 0.423 | | | forkhead box L2 |
| FOXM1 | 0.990 | | 0.782 | | 0.881 | | 0.304 | | 0.817 | | 0.130 | | 0.953 | | 0.133 | | | forkhead box M1 |
| GAS41 | 0.674 | | 0.260 | | 0.904 | | 0.152 | | 0.776 | | 0.044 | | 1.186 | | 0.004 | | | glioma-amplified sequence-41 |
| GASC1 | 0.837 | | 0.082 | | 0.778 | | 0.210 | | 0.954 | | 0.601 | | 1.177 | | 0.158 | | | gene amplified in squamous cell carcinoma 1 |
| GATA1 | 0.770 | | 0.510 | | 0.975 | | 0.823 | | 0.828 | | 0.293 | | 0.984 | | 0.714 | | | GATA-binding protein 1 (globin transcription factor 1) |
| GATA3 | 0.817 | | 0.027 | | 0.836 | | 0.520 | | 0.771 | | 0.043 | | 0.694 | | 2.16E-05 | | | GATA-binding protein 3 |
| GCMB | 1.273 | | 0.020 | | 1.047 | | 0.484 | | 0.915 | | 0.357 | | 1.028 | | 0.731 | | | glial cells missing (Drosophila) homolog b |
| GCN5L1 | 1.078 | | 0.275 | | 0.919 | | 0.508 | | 1.035 | | 0.750 | | 0.701 | | 0.001 | | | GCN5 (general control of amino-acid synthesis, yeast, homolog)-like 1 |
| GLI2 | 1.107 | | 0.338 | | 1.359 | | 0.153 | | 0.913 | | 0.405 | | 0.948 | | 0.194 | | | GLI-Kruppel family member GLI2 |
| GLI3 | 1.327 | | 0.081 | | 1.055 | | 0.368 | | 1.368 | | 0.249 | | 1.047 | | 0.232 | | | GLI-Kruppel family member GLI3 (Greig cephalopolysyndactyly syndrome) |
| GLIS2 | 1.295 | | 0.275 | | 1.167 | | 0.196 | | 1.015 | | 0.850 | | 0.979 | | 0.409 | | | Kruppel-like zinc finger protein GLIS2 |
| GTF2A1 | 0.979 | | 0.747 | | 1.025 | | 0.757 | | 0.728 | | 0.122 | | 1.096 | | 0.072 | | | general transcription factor IIA, 1 (37kD and 19kD subunits) |
| GTF2B | 1.219 | | 0.145 | | 1.268 | | 0.037 | | 1.063 | | 0.263 | | 0.799 | | 0.007 | | | general transcription factor IIB |
| GTF2H3 | 1.102 | | 0.265 | | 1.084 | | 0.231 | | 0.828 | | 0.173 | | 1.093 | | 0.051 | | | general transcription factor IIH, polypeptide 3 (34kD subunit) |
| GTF2I | 0.680 | | 0.168 | | 0.925 | | 0.788 | | 1.173 | | 0.418 | | 0.909 | | 0.092 | | | general transcription factor II, i |
| GTF3A | 1.024 | | 0.719 | | 1.054 | | 0.296 | | 0.744 | | 0.052 | | 1.084 | | 0.039 | | | general transcription factor IIIA |
| HBOA | 1.225 | | 0.006 | | 1.099 | | 0.282 | | 0.971 | | 0.727 | | 0.983 | | 0.625 | | | histone acetyltransferase |
| HCF2 | 0.749 | | 2.68E-04 | | 1.009 | | 0.894 | | 0.800 | | 0.258 | | 1.076 | | 0.173 | | | host cell factor 2 |
| HDAC4 | 1.206 | | 0.829 | | 1.071 | | 0.153 | | 0.999 | | 0.996 | | 0.996 | | 0.911 | | | histone deacetylase 4 |
| HES2 | 1.454 | | 0.003 | | 1.029 | | 0.751 | | 0.890 | | 0.221 | | 0.675 | | 0.011 | | | hairy and enhancer of split 2 (Drosophila) |
| HES7 | 1.388 | | 0.012 | | 0.983 | | 0.752 | | 0.962 | | 0.681 | | 0.722 | | 0.001 | | | hairy and enhancer of split 7 (Drosophila) |
| HEYL | 1.294 | | 0.054 | | 1.002 | | 0.971 | | 1.160 | | 0.135 | | 1.024 | | 0.726 | | | hairy/enhancer-of-split related with YRPW motif-like wa |
| HIRA | 1.129 | | 0.339 | | 0.728 | | 0.197 | | 0.929 | | 0.590 | | 1.069 | | 0.235 | | | HIR (histone cell cycle regulation defective, S. cerevisiae) homolog A |
| HKR2 | 0.801 | | 0.001 | | 0.969 | | 0.643 | | 0.923 | | 0.424 | | 1.258 | | 0.013 | | | GLI-Kruppel family member HKR2 |
| HLX1 | 0.809 | | 0.038 | | 1.033 | | 0.853 | | 1.096 | | 0.567 | | 0.706 | | 0.014 | | | H2.0 (Drosophila)-like homeo box 1 |
| HMG20B | 1.099 | | 0.036 | | 0.982 | | 0.814 | | 0.996 | | 0.910 | | 1.198 | | 0.002 | | | high-mobility group 20B |
| HMGIY | 1.098 | | 0.331 | | 1.190 | | 0.227 | | 1.263 | | 0.077 | | 1.001 | | 0.976 | | | high-mobility group (nonhistone chromosomal) protein isoforms I and Y |
| HNF3B | 1.377 | | 0.124 | | 1.094 | | 0.453 | | 0.857 | | 0.030 | | 0.806 | | 0.002 | | | hepatocyte nuclear factor 3, beta |
| HNF4A | 1.563 | | 0.059 | | 0.959 | | 0.458 | | 1.178 | | 0.179 | | 1.111 | | 0.022 | | | hepatocyte nuclear factor 4, alpha |
| HNF4G | 1.140 | | 0.872 | | 0.831 | | 0.096 | | 1.232 | | 0.204 | | 1.077 | | 0.374 | | | hepatocyte nuclear factor 4, gamma |
| HOX11 | 0.713 | | 0.009 | | 1.015 | | 0.879 | | 0.894 | | 0.166 | | 0.980 | | 0.506 | | | T-cell leukemia, homeobox 1 (TLX1) |
| HOXA4 | 1.307 | | 0.007 | | 0.960 | | 0.711 | | 1.113 | | 0.276 | | 1.276 | | 0.006 | | | homeobox A4 |
| HOXA6 | 0.824 | | 0.142 | | 1.080 | | 0.324 | | 1.157 | | 0.445 | | 0.889 | | 0.059 | | | homeobox A6 |
| HOXB1 | 1.065 | | 0.463 | | 0.758 | | 0.157 | | 0.928 | | 0.632 | | 1.025 | | 0.462 | | | homeobox B1 |
| HOXC10 | 0.857 | | 0.220 | | 0.984 | | 0.748 | | 0.878 | | 0.249 | | 1.260 | | 0.017 | | | homeobox C10 |
| HOXC11 | 1.869 | | 0.045 | | 1.274 | | 0.104 | | 0.986 | | 0.830 | | 0.848 | | 0.011 | | | homeobox C11 |
| HOXC12 | 1.020 | | 0.842 | | 0.809 | | 0.875 | | 0.857 | | 0.559 | | 1.051 | | 0.224 | | | homeoboxC12 |
| HOXC13 | 0.763 | | 0.173 | | 0.758 | | 0.137 | | 1.042 | | 0.546 | | 0.949 | | 0.353 | | | homeobox C13 |
| HOXC9 | 1.243 | | 0.613 | | 1.027 | | 0.629 | | 0.884 | | 0.094 | | 0.998 | | 0.961 | | | homeobox C9 |
| HOXD1 | 1.289 | | 0.044 | | 0.941 | | 0.494 | | 1.272 | | 0.303 | | 1.061 | | 0.077 | | | homeobox D1 |
| HOXD11 | 0.863 | | 0.217 | | 0.857 | | 0.181 | | 0.945 | | 0.569 | | 1.200 | | 0.001 | | | homeobox D11 |
| HOXD8 | 0.769 | | 0.251 | | 1.021 | | 0.744 | | 1.110 | | 0.372 | | 1.272 | | 0.002 | | | homeobox D8 |
| HR | 0.802 | | 0.012 | | 0.974 | | 0.529 | | 0.904 | | 0.016 | | 1.306 | | 0.094 | | | hairless |
| HS747E2A | 0.900 | | 0.190 | | 0.791 | | 0.124 | | 1.134 | | 0.472 | | 1.046 | | 0.088 | | | hypothetical protein (RING domain) |
| HSAJ2425 | 1.109 | | 0.372 | | 1.192 | | 0.689 | | 1.287 | | 0.231 | | 1.313 | | 3.89E-04 | | | p65 protein |
| HSF2 | 0.979 | | 0.645 | | 1.160 | | 0.276 | | 1.244 | | 0.166 | | 0.946 | | 0.462 | | | heat shock transcription factor 2 |
| HSF4 | 1.249 | | 0.011 | | 1.086 | | 0.131 | | 0.975 | | 0.897 | | 0.972 | | 0.705 | | | heat shock transcription factor 4 |
| HSPC189 | 1.469 | | 0.039 | | 0.994 | | 0.912 | | 0.918 | | 0.061 | | 1.028 | | 0.420 | | | zinc finger protein ZNF581 |
| HSU90653 | 0.644 | | 0.064 | | 0.806 | | 0.030 | | 0.791 | | 0.230 | | 1.217 | | 0.011 | | | zinc finger, DHHC domain containing 1 (ZDHHC1) |
| ID3 | 0.911 | | 0.455 | | 1.005 | | 0.964 | | 0.812 | | 0.181 | | 1.223 | | 0.002 | | | inhibitor of DNA binding 3, dominant negative helix-loop-helix protein |
| ILF2 | 1.177 | | 0.076 | | 1.078 | | 0.263 | | 1.063 | | 0.241 | | 0.763 | | 0.005 | | | interleukin enhancer binding factor 2 |
| DNAJ | 1.032 | | 0.870 | | 1.219 | | 0.302 | | 1.305 | | 0.300 | | 0.740 | | 0.015 | | | immune dysregulation, polyendocrinopathy, enteropathy, X-linked |
| IRF1 | 0.652 | | 0.036 | | 1.461 | | 0.149 | | 1.066 | | 0.702 | | 0.849 | | 0.011 | | | interferon regulatory factor 1 |
| IRF6 | 0.962 | | 0.789 | | 1.087 | | 0.791 | | 0.631 | | 0.060 | | 0.696 | | 0.012 | | | interferon regulatory factor 6 |
| IRLB | 0.965 | | 0.713 | | 1.290 | | 0.007 | | 1.123 | | 0.600 | | 0.891 | | 0.038 | | | c-myc promoter-binding protein |
| JUND | 0.994 | | 0.962 | | 1.161 | | 0.295 | | 1.166 | | 0.223 | | 1.308 | | 0.019 | | | Jun D proto-oncogene |
| KIAA0014 | 1.811 | | 0.055 | | 0.866 | | 0.394 | | 0.953 | | 0.818 | | 1.288 | | 0.051 | | | leucine rich repeat containing 14 (LRRC14) |
| KIAA0026 | 0.967 | | 0.700 | | 0.987 | | 0.732 | | 1.215 | | 0.073 | | 1.018 | | 0.506 | | | mortality factor 4 like 2 (MORF4L2) |
| KIAA0132 | 0.956 | | 0.452 | | 1.055 | | 0.434 | | 0.774 | | 0.155 | | 0.846 | | 0.020 | | | Kelch-like ECH-associated protein 1 (KEAP1) |
| KIAA0173 | 0.991 | | 0.845 | | 1.054 | | 0.432 | | 1.089 | | 0.705 | | 1.247 | | 3.33E-04 | | | tubulin tyrosine ligase-like family, member 4 (TTLL4) |
| KIAA0293 | 1.270 | | 0.013 | | 1.201 | | 0.159 | | 1.139 | | 0.371 | | 0.916 | | 0.033 | | | cut-like 2 (Drosophila) (CUTL2) |
| KIAA0296 | 0.992 | | 0.935 | | 1.015 | | 0.830 | | 1.413 | | 0.244 | | 0.987 | | 0.724 | | | zinc fingerprotein ZNF646 |
| KIAA0395 | 0.722 | | 0.037 | | 0.834 | | 0.246 | | 0.934 | | 0.344 | | 1.186 | | 0.032 | | | triple homeobox 1 (TIX1) |
| KIAA0414 | 0.911 | | 0.267 | | 0.856 | | 0.002 | | 0.804 | | 0.178 | | 1.122 | | 0.011 | | | zinc finger protein ZNF297B |
| KIAA0441 | 1.184 | | 0.001 | | 1.102 | | 0.097 | | 0.773 | | 0.122 | | 0.868 | | 0.074 | | | zinc finger and BTB domain containing 24 (ZBTB24) |
| KIAA0535 | 0.721 | | 0.046 | | 0.996 | | 0.953 | | 0.916 | | 0.372 | | 1.054 | | 0.234 | | | suppression of tumorigenicity 18 (breast carcinoma) (zinc finger protein) (ST18) |
| KIAA0943 | 1.157 | | 0.242 | | 1.393 | | 0.140 | | 1.242 | | 0.015 | | 1.025 | | 0.715 | | | ATG4 autophagy related 4 homolog B (S. cerevisiae) (ATG4B or APG4B) |
| KIAA0961 | 0.962 | | 0.559 | | 1.103 | | 0.200 | | 0.797 | | 0.218 | | 1.013 | | 0.692 | | | zinc finger protein ZFP30 |
| KIAA0972 | 0.768 | | 0.066 | | 0.828 | | 0.143 | | 0.944 | | 0.693 | | 1.143 | | 0.092 | | | zinc finger protein ZNF510 |
| KIAA0998 | 0.929 | | 0.388 | | 1.111 | | 0.526 | | 0.948 | | 0.472 | | 1.250 | | 0.002 | | | tubulin tyrosine ligase-like family, member 5 (TTLL5) |
| KIAA1190 | 0.829 | | 0.040 | | 0.834 | | 0.088 | | 0.801 | | 0.008 | | 1.250 | | 0.003 | | | hypothetical protein KIAA1190 |
| KIAA1388 | 0.823 | | 0.003 | | 0.847 | | 0.066 | | 1.083 | | 0.367 | | 1.031 | | 0.484 | | | zinc finger protein ZNF319 |
| KIAA1528 | 1.226 | | 0.270 | | 0.803 | | 0.044 | | 1.055 | | 0.487 | | 1.164 | | 2.08E-04 | | | deltex homolog 2 (Drosophila) (DTX2) |
| KIAA1542 | 0.801 | | 0.361 | | 0.967 | | 0.348 | | 1.011 | | 0.914 | | 0.847 | | 0.006 | | | KIAA1542 protein |
| KLF13 | 0.971 | | 0.422 | | 1.051 | | 0.661 | | 0.795 | | 0.061 | | 0.898 | | 0.078 | | | Kruppel-like factor 13 |
| KLF15 | 0.978 | | 0.829 | | 1.211 | | 0.156 | | 1.095 | | 0.067 | | 0.885 | | 0.058 | | | Kruppel-like factor 15 |
| KLF5 | 1.002 | | 0.970 | | 1.147 | | 0.364 | | 0.789 | | 0.260 | | 0.934 | | 0.325 | | | Kruppel-like factor 5 |
| KLF7 | 0.804 | | 0.336 | | 0.837 | | 0.648 | | 1.004 | | 0.966 | | 1.007 | | 0.850 | | | Kruppel-like factor 7 |
| KLHL4 | 0.834 | | 0.318 | | 0.732 | | 0.100 | | 1.099 | | 0.520 | | 0.888 | | 0.057 | | | kelch (Drosophila)-like 4 |
| KRML | 1.018 | | 0.917 | | 1.191 | | 0.239 | | 0.974 | | 0.733 | | 0.786 | | 0.001 | | | v-maf musculoaponeurotic fibrosarcoma oncogene homolog B (avian) (MAFB) |
| LDB1 | 1.295 | | 0.001 | | 1.287 | | 0.028 | | 0.948 | | 0.540 | | 0.886 | | 0.014 | | | LIM domain binding 1 |
| LDB2 | 1.273 | | 0.168 | | 0.975 | | 0.711 | | 0.989 | | 0.850 | | 1.031 | | 0.559 | | | LIM domain binding 2 |
| LHX9 | 0.993 | | 0.935 | | 1.265 | | 0.103 | | 0.968 | | 0.770 | | 0.935 | | 0.170 | | | LIM homeobox protein 9 |
| LMO2 | 0.818 | | 0.092 | | 1.058 | | 0.245 | | 1.007 | | 0.856 | | 0.968 | | 0.388 | | | LIM domain only 2 (rhombotin-like 1) |
| LMO4 | 0.823 | | 0.331 | | 0.979 | | 0.786 | | 1.061 | | 0.638 | | 0.766 | | 0.004 | | | LIM domain only 4 |
| LOC51270 | 1.070 | | 0.366 | | 1.258 | | 0.056 | | 1.005 | | 0.951 | | 1.019 | | 0.563 | | | E2F-like protein |
| LOC51652 | 1.229 | | 0.036 | | 1.015 | | 0.828 | | 0.839 | | 0.081 | | 1.105 | | 0.070 | | | CGI-149 protein |
| LOC55885 | 1.027 | | 0.710 | | 0.802 | | 0.086 | | 1.028 | | 0.762 | | 0.952 | | 0.178 | | | neuronal specific transcription factor DAT1 |
| LOC56270 | 1.107 | | 0.283 | | 0.891 | | 0.514 | | 1.231 | | 0.202 | | 0.907 | | 0.044 | | | WDR45-like (WDR45L) or WIPI49-like protein (WIPI3) |
| LOC56930 | 0.738 | | 0.035 | | 1.201 | | 0.379 | | 0.848 | | 0.151 | | 1.171 | | 0.043 | | | hypothetical protein |
| LOC57209 | 1.043 | | 0.764 | | 0.767 | | 0.298 | | 1.149 | | 0.251 | | 1.210 | | 0.012 | | | Kruppel-type zinc finger protein |
| LZTS1 | 0.855 | | 0.289 | | 0.781 | | 0.061 | | 0.989 | | 0.903 | | 1.029 | | 0.571 | | | leucine zipper, putative tumor suppressor 1 |
| M96 | 0.783 | | 0.062 | | 1.139 | | 0.320 | | 1.233 | | 0.022 | | 1.006 | | 0.858 | | | putative DNA binding protein |
| MAD | 1.035 | | 0.675 | | 1.151 | | 0.463 | | 0.791 | | 0.132 | | 0.807 | | 0.041 | | | MAX dimerization protein 1 |
| MAD4 | 1.434 | | 0.099 | | 1.000 | | 0.996 | | 0.960 | | 0.421 | | 1.045 | | 0.086 | | | MAX dimerization protein 4 (MXD4) |
| MADH2 | 1.068 | | 0.620 | | 1.086 | | 0.574 | | 1.152 | | 0.064 | | 1.227 | | 0.023 | | | MAD (mothers against decapentaplegic, Drosophila) homolog 2 |
| MADH5 | 0.817 | | 0.141 | | 0.832 | | 0.013 | | 0.960 | | 0.476 | | 1.035 | | 0.138 | | | MAD (mothers against decapentaplegic, Drosophila) homolog 5 |
| MADH7 | 0.945 | | 0.334 | | 0.821 | | 0.127 | | 0.958 | | 0.861 | | 1.049 | | 0.241 | | | MAD (mothers against decapentaplegic, Drosophila) homolog 7 |
| MAF | 1.004 | | 0.962 | | 1.039 | | 0.278 | | 0.801 | | 0.144 | | 1.071 | | 0.132 | | | v-maf musculoaponeurotic fibrosarcoma oncogene homolog (avian) |
| MAFF | 1.273 | | 0.010 | | 1.155 | | 0.050 | | 1.175 | | 0.067 | | 0.964 | | 0.463 | | | v-maf musculoaponeurotic fibrosarcoma (avian) oncogene family, protein F |
| MAFG | 0.626 | | 0.125 | | 0.742 | | 0.054 | | 0.786 | | 0.082 | | 1.120 | | 0.229 | | | v-maf musculoaponeurotic fibrosarcoma oncogene family, protein G (avian) |
| MAPK8IP1 | 1.021 | | 0.690 | | 0.793 | | 0.014 | | 1.144 | | 0.749 | | 1.063 | | 0.239 | | | mitogen-activated protein kinase 8 interacting protein 1 |
| MEF2A | 0.881 | | 0.276 | | 1.219 | | 0.052 | | 0.752 | | 0.497 | | 0.912 | | 0.096 | | | MADS box transcription enhancer factor 2, polypeptide A (myocyte enhancer factor 2A) |
| MEF2B | 1.356 | | 1.94E-04 | | 0.908 | | 0.638 | | 0.979 | | 0.716 | | 1.089 | | 0.037 | | | MADS box transcription enhancer factor 2, polypeptide B (myocyte enhancer factor 2B) |
| MEIS3 | 0.737 | | 0.011 | | 0.979 | | 0.855 | | 1.160 | | 0.205 | | 1.086 | | 0.076 | | | meis1-related protein 2 aka MRG2 |
| MEOX2 | 1.066 | | 0.275 | | 1.057 | | 0.531 | | 1.255 | | 0.052 | | 0.975 | | 0.698 | | | mesenchyme homeo box 2 (growth arrest-specific homeo box) |
| MHC2TA | 1.361 | | 0.050 | | 0.918 | | 0.567 | | 0.887 | | 0.321 | | 1.062 | | 0.447 | | | MHC class II transactivator |
| MORF | 1.016 | | 0.873 | | 1.130 | | 0.007 | | 1.327 | | 0.114 | | 0.939 | | 0.051 | | | histone acetyltransferase |
| MYB | 1.219 | | 0.039 | | 0.933 | | 0.473 | | 1.080 | | 0.339 | | 1.016 | | 0.781 | | | v-myb myeloblastosis viral oncogene homolog (avian) |
| MYBL2 | 1.155 | | 0.196 | | 1.028 | | 0.769 | | 0.790 | | 0.150 | | 0.869 | | 0.023 | | | v-myb avian myeloblastosis viral oncogene homolog-like 2 |
| MYF6 | 0.944 | | 0.386 | | 0.985 | | 0.590 | | 0.786 | | 0.045 | | 1.141 | | 0.046 | | | myogenic factor 6 (herculin) |
| MYT2 | 0.942 | | 0.501 | | 1.207 | | 0.044 | | 1.136 | | 0.041 | | 1.030 | | 0.287 | | | myelin transcription factor 2 |
| NAB1 | 1.067 | | 0.483 | | 0.979 | | 0.757 | | 1.303 | | 0.154 | | 0.953 | | 0.238 | | | NGFI-A binding protein 1 (EGR1 binding protein 1) |
| NCOA3 | 0.749 | | 0.159 | | 0.876 | | 0.005 | | 0.916 | | 0.354 | | 1.092 | | 0.309 | | | nuclear receptor coactivator 3 |
| NCOA4 | 1.210 | | 0.236 | | 1.043 | | 0.458 | | 1.097 | | 0.280 | | 0.891 | | 0.004 | | | nuclear receptor coactivator 4 |
| NCOR2 | 1.178 | | 0.077 | | 1.114 | | 0.347 | | 1.291 | | 0.034 | | 1.106 | | 0.194 | | | nuclear receptor co-repressor 2 |
| NEUROD6 | 0.828 | | 0.097 | | 1.013 | | 0.678 | | 1.002 | | 0.968 | | 0.949 | | 0.101 | | | neurogenic differentiation 6 |
| NEUROG1 | 1.976 | | 0.045 | | 1.244 | | 0.036 | | 1.014 | | 0.726 | | 1.117 | | 0.006 | | | neurogenin 1 |
| NFATC2 | 1.253 | | 0.015 | | 0.786 | | 0.406 | | 1.249 | | 0.203 | | 1.160 | | 0.156 | | | nuclear factor of activated T-cells, cytoplasmic, calcineurin-dependent 2 |
| NFIB | 1.146 | | 0.476 | | 1.179 | | 0.196 | | 1.257 | | 0.037 | | 1.086 | | 0.021 | | | nuclear factor I/B |
| NFIC | 1.383 | | 0.097 | | 0.793 | | 0.312 | | 1.021 | | 0.861 | | 1.192 | | 0.033 | | | nuclear factor I/C (CCAAT-binding transcription factor) |
| NFIL3 | 2.150 | | 0.008 | | 1.319 | | 0.009 | | 0.801 | | 0.051 | | 0.968 | | 0.473 | | | nuclear factor, interleukin 3 regulated |
| NFIX | 0.717 | | 0.442 | | 1.196 | | 0.362 | | 0.933 | | 0.526 | | 1.167 | | 0.045 | | | nuclear factor I/X (CCAAT-binding transcription factor) |
| NFKBIE | 1.320 | | 0.068 | | 0.959 | | 0.479 | | 1.061 | | 0.380 | | 1.046 | | 0.289 | | | nuclear factor of kappa light polypeptide gene enhancer in B-cells inhibitor, epsilon |
| NFX1 | 1.171 | | 0.277 | | 1.184 | | 0.039 | | 1.223 | | 0.217 | | 0.766 | | 0.001 | | | nuclear transcription factor, X-box binding 1 |
| NHLH2 | 1.757 | | 0.037 | | 0.944 | | 0.725 | | 0.971 | | 0.828 | | 0.883 | | 0.175 | | | nescient helix loop helix 2 |
| NKX2B | 1.447 | | 0.024 | | 1.290 | | 0.164 | | 1.135 | | 0.467 | | 0.782 | | 0.021 | | | NK2 transcription factor related, locus 2 (Drosophila) (NKX2-2) |
| NMI | 1.051 | | 0.558 | | 0.908 | | 0.224 | | 1.235 | | 0.325 | | 0.965 | | 0.550 | | | N-myc (and STAT) interactor |
| NPAS1 | 1.289 | | 0.001 | | 1.050 | | 0.356 | | 1.128 | | 0.234 | | 1.015 | | 0.625 | | | neuronal PAS domain protein 1 |
| NR1H3 | 0.991 | | 0.935 | | 1.207 | | 0.041 | | 1.491 | | 0.240 | | 0.847 | | 0.009 | | | nuclear receptor subfamily 1, group H, member 3 |
| NR1I3 | 1.039 | | 0.586 | | 0.803 | | 0.035 | | 1.047 | | 0.817 | | 1.124 | | 0.077 | | | nuclear receptor subfamily 1, group I, member 3 |
| NR2C2 | 1.273 | | 0.156 | | 1.233 | | 0.023 | | 1.080 | | 0.262 | | 0.957 | | 0.207 | | | nuclear receptor subfamily 2, group C, member 2 |
| NR2F2 | 1.233 | | 0.005 | | 1.152 | | 0.010 | | 1.212 | | 0.017 | | 0.924 | | 0.154 | | | nuclear receptor subfamily 2, group F, member 2 |
| NR2F6 | 1.220 | | 0.015 | | 1.192 | | 0.027 | | 1.140 | | 0.424 | | 0.858 | | 0.002 | | | nuclear receptor subfamily 2, group F, member 6 |
| NR3C2 | 1.222 | | 0.623 | | 1.191 | | 0.185 | | 1.189 | | 0.059 | | 0.943 | | 0.229 | | | nuclear receptor subfamily 3, group C, member 2 |
| NR5A1 | 0.907 | | 0.250 | | 1.284 | | 0.118 | | 1.289 | | 0.123 | | 0.997 | | 0.950 | | | nuclear receptor subfamily 5, group A, member 1 |
| NR5A2 | 1.205 | | 0.154 | | 0.975 | | 0.763 | | 0.849 | | 0.434 | | 1.044 | | 0.158 | | | nuclear receptor subfamily 5, group A, member 2 |
| OAZ | 0.792 | | 0.014 | | 0.806 | | 0.484 | | 1.029 | | 0.576 | | 1.050 | | 0.402 | | | OLF-1/EBF associated zinc finger gene |
| OCT11 | 0.762 | | 0.175 | | 0.992 | | 0.905 | | 1.096 | | 0.281 | | 1.069 | | 0.089 | | | POU domain, class 2, transcription factor 3 (POU2F3) |
| OG2x | 0.804 | | 0.485 | | 1.067 | | 0.372 | | 1.079 | | 0.410 | | 0.972 | | 0.457 | | | OG2x OG2 homeobox (mouse) homolog |
| ONECUT1 | 0.706 | | 0.252 | | 1.142 | | 0.055 | | 1.054 | | 0.478 | | 0.873 | | 0.008 | | | one cut domain, family member 1 |
| ONECUT2 | 1.099 | | 0.035 | | 1.288 | | 0.096 | | 1.226 | | 0.002 | | 0.930 | | 0.128 | | | one cut domain, family member 2 |
| P1P373C6 | 1.282 | | 0.034 | | 0.975 | | 0.795 | | 1.212 | | 0.004 | | 0.922 | | 0.166 | | | hypothetical protein P1 p373c6 |
| P38IP | 0.901 | | 0.177 | | 0.789 | | 0.171 | | 1.170 | | 0.246 | | 1.100 | | 0.052 | | | transcription factor (p38 interacting protein) |
| PAF65A | 0.739 | | 0.027 | | 1.024 | | 0.882 | | 1.117 | | 0.394 | | 1.003 | | 0.965 | | | PCAF associated factor 65 alpha |
| PAX3 | 1.289 | | 0.319 | | 1.325 | | 0.048 | | 0.970 | | 0.681 | | 1.003 | | 0.931 | | | paired box gene 3 (Waardenburg syndrome 1) |
| PAX4 | 1.228 | | 0.096 | | 1.043 | | 0.448 | | 0.921 | | 0.299 | | 0.755 | | 0.025 | | | paired box gene 4 |
| PAX6 | 0.852 | | 0.137 | | 0.805 | | 0.104 | | 1.018 | | 0.842 | | 1.098 | | 0.024 | | | paired box gene 6 (aniridia, keratitis) |
| PAX7 | 0.762 | | 0.003 | | 0.979 | | 0.805 | | 0.951 | | 0.715 | | 0.936 | | 0.520 | | | paired box gene 7 |
| PAX8 | 1.355 | | 0.066 | | 1.137 | | 0.493 | | 0.839 | | 0.052 | | 0.910 | | 0.242 | | | paired box gene 8 |
| PAX9 | 1.339 | | 0.073 | | 1.137 | | 0.058 | | 0.812 | | 0.043 | | 0.778 | | 8.71E-05 | | | paired box gene 9 |
| PBX1 | 0.787 | | 0.174 | | 1.018 | | 0.815 | | 0.909 | | 0.502 | | 1.115 | | 0.021 | | | pre-B-cell leukemia transcription factor 1 |
| PBX2 | 0.960 | | 0.788 | | 1.209 | | 0.025 | | 0.994 | | 0.949 | | 0.937 | | 0.125 | | | pre-B-cell leukemia transcription factor 2 |
| PER1 | 1.243 | | 0.035 | | 1.096 | | 0.249 | | 1.236 | | 0.109 | | 0.991 | | 0.862 | | | period homolog 1 (Drosophila) |
| PER2 | 1.144 | | 0.546 | | 1.214 | | 0.036 | | 1.236 | | 0.308 | | 1.138 | | 0.069 | | | period (Drosophila) homolog 2 |
| PIG7 | 0.827 | | 0.117 | | 1.222 | | 0.645 | | 1.028 | | 0.767 | | 1.058 | | 0.342 | | | LPS-induced TNF-alpha factor |
| PILB | 1.256 | | 0.107 | | 1.000 | | 0.996 | | 1.012 | | 0.924 | | 1.123 | | 0.021 | | | pilin-like transcription factor |
| PLAGL1 | 0.966 | | 0.586 | | 1.168 | | 0.342 | | 1.293 | | 0.227 | | 0.728 | | 0.026 | | | pleiomorphic adenoma gene-like 1 |
| PLRG1 | 0.823 | | 0.108 | | 1.002 | | 0.984 | | 0.988 | | 0.855 | | 0.977 | | 0.430 | | | pleiotropic regulator 1 (PRL1 homolog, Arabidopsis) |
| POU1F1 | 1.207 | | 0.170 | | 1.159 | | 0.063 | | 1.082 | | 0.323 | | 0.805 | | 0.012 | | | POU domain, class 1, transcription factor 1 (Pit1, growth hormone factor 1) |
| POU2F1 | 0.757 | | 0.070 | | 1.125 | | 0.319 | | 1.120 | | 0.435 | | 0.931 | | 0.103 | | | POU domain, class 2, transcription factor 1 |
| POU3F4 | 0.945 | | 0.387 | | 1.006 | | 0.947 | | 1.210 | | 0.336 | | 0.954 | | 0.260 | | | POU domain, class 3, transcription factor 4 |
| PPARBP | 0.978 | | 0.766 | | 1.030 | | 0.687 | | 1.383 | | 0.111 | | 0.908 | | 0.138 | | | peroxisome proliferator activated receptor binding protein |
| PPARD | 0.925 | | 0.634 | | 1.156 | | 0.148 | | 0.819 | | 0.007 | | 0.978 | | 0.600 | | | peroxisome proliferative activated receptor, delta |
| PPARG | 0.788 | | 0.381 | | 1.070 | | 0.595 | | 1.599 | | 0.056 | | 0.988 | | 0.658 | | | peroxisome proliferative activated receptor, gamma |
| PRDM11 | 1.725 | | 0.016 | | 1.100 | | 0.149 | | 1.098 | | 0.600 | | 0.974 | | 0.663 | | | PR domain containing 11 |
| PRDM13 | 0.749 | | 0.002 | | 1.146 | | 0.410 | | 0.791 | | 0.514 | | 2.048 | | 0.006 | | | PR domain containing 13 |
| PRDM14 | 1.571 | | 0.152 | | 1.086 | | 0.239 | | 0.901 | | 0.399 | | 1.095 | | 0.060 | | | PR domain containing 14 |
| PRDM15 | 0.814 | | 0.037 | | 1.316 | | 0.060 | | 1.149 | | 0.448 | | 0.853 | | 0.032 | | | PR domain containing 15 |
| PRDM7 | 1.333 | | 0.066 | | 1.116 | | 0.957 | | 1.158 | | 0.030 | | 0.954 | | 0.245 | | | PR domain containing 7 |
| PROX1 | 1.019 | | 0.723 | | 1.204 | | 0.056 | | 1.153 | | 0.082 | | 0.965 | | 0.371 | | | prospero-related homeobox 1 |
| RARA | 0.741 | | 0.006 | | 1.047 | | 0.426 | | 1.127 | | 0.372 | | 1.060 | | 0.240 | | | retinoic acid receptor, alpha |
| RARG | 1.318 | | 0.015 | | 1.193 | | 0.269 | | 1.061 | | 0.801 | | 1.017 | | 0.643 | | | retinoic acid receptor, gamma |
| RBBP9 | 1.001 | | 0.986 | | 0.933 | | 0.563 | | 1.309 | | 0.045 | | 1.129 | | 0.008 | | | retinoblastoma binding protein 9 |
| RBPSUHL | 1.232 | | 0.536 | | 1.122 | | 0.188 | | 1.033 | | 0.432 | | 0.813 | | 0.008 | | | recombining binding protein suppressor of hairless-like (Drosophila) |
| RELA | 1.241 | | 0.008 | | 1.138 | | 0.425 | | 1.002 | | 0.965 | | 0.936 | | 0.086 | | | v-rel reticuloendotheliosis viral oncogene homolog A, nuclear factor of kappa light polypeptide gene enhancer in B-cells 3, p65 (avian) |
| RELB | 0.743 | | 0.015 | | 1.031 | | 0.590 | | 1.093 | | 0.473 | | 1.009 | | 0.873 | | | v-rel reticuloendotheliosis viral oncogene homolog B, nuclear factor of kappa light polypeptide gene enhancer in B-cells 3 (avian) |
| RFP | 0.812 | | 0.201 | | 0.828 | | 0.102 | | 1.131 | | 0.421 | | 1.176 | | 0.015 | | | ret finger protein |
| RFX2 | 0.768 | | 0.192 | | 0.843 | | 0.137 | | 0.974 | | 0.649 | | 1.067 | | 0.214 | | | regulatory factor X, 2 (influences HLA class II expression) |
| RFX5 | 1.073 | | 0.445 | | 1.003 | | 0.964 | | 0.805 | | 0.196 | | 1.011 | | 0.855 | | | regulatory factor X, 5 (influences HLA class II expression) |
| RFXANK | 0.742 | | 0.048 | | 1.005 | | 0.940 | | 1.134 | | 0.127 | | 0.867 | | 0.004 | | | regulatory factor X-associated ankyrin-containing protein |
| RGC32 | 1.009 | | 0.891 | | 0.889 | | 0.019 | | 1.205 | | 0.119 | | 1.160 | | 0.001 | | | RGC32 protein |
| RLF | 0.708 | | 0.087 | | 0.910 | | 0.350 | | 0.968 | | 0.656 | | 1.007 | | 0.905 | | | rearranged L-myc fusion sequence |
| RNF4 | 1.511 | | 0.027 | | 1.348 | | 0.198 | | 0.954 | | 0.768 | | 0.873 | | 0.231 | | | ring finger protein 4 |
| RORB | 1.289 | | 0.286 | | 1.296 | | 0.144 | | 1.425 | | 0.203 | | 0.832 | | 0.002 | | | RAR-related orphan receptor B |
| RRN3 | 1.018 | | 0.889 | | 0.826 | | 0.087 | | 1.079 | | 0.453 | | 1.033 | | 0.212 | | | RNA polymerase I transcription factor RRN3 |
| RXRB | 1.399 | | 0.015 | | 0.990 | | 0.857 | | 0.936 | | 0.258 | | 0.889 | | 0.010 | | | retinoid X receptor, beta |
| SAFB | 1.238 | | 0.307 | | 1.208 | | 0.277 | | 1.298 | | 0.175 | | 1.026 | | 0.667 | | | scaffold attachment factor B |
| SALL3 | 0.976 | | 0.753 | | 0.812 | | 0.122 | | 1.321 | | 0.191 | | 1.077 | | 0.391 | | | sal-like 3 (Drosophila) |
| SATB1 | 0.783 | | 0.072 | | 0.933 | | 0.070 | | 1.091 | | 0.156 | | 0.902 | | 0.004 | | | special AT-rich sequence binding protein 1 (binds to nuclear matrix/scaffold-associating DNAs) |
| SCML2 | 0.736 | | 0.279 | | 0.967 | | 0.649 | | 0.910 | | 0.081 | | 1.067 | | 0.147 | | | sex comb on midleg-like 2 (Drosophila) |
| SETBP1 | 0.989 | | 0.825 | | 0.922 | | 0.966 | | 0.824 | | 0.051 | | 0.984 | | 0.695 | | | SET binding protein 1 |
| SHARP | 0.730 | | 0.068 | | 0.971 | | 0.611 | | 0.900 | | 0.059 | | 0.956 | | 0.232 | | | SMART/HDAC1 associated repressor protein |
| SHOX2 | 1.221 | | 0.028 | | 0.899 | | 0.299 | | 1.032 | | 0.784 | | 1.009 | | 0.833 | | | short stature homeobox 2 |
| SIAH1 | 0.782 | | 0.073 | | 0.950 | | 0.641 | | 0.747 | | 0.129 | | 1.052 | | 0.572 | | | seven in absentia (Drosophila) homolog 1 |
| SIM1 | 1.206 | | 0.013 | | 1.043 | | 0.585 | | 0.969 | | 0.548 | | 0.992 | | 0.885 | | | single-minded homolog 1 (Drosophila) |
| SIX1 | 0.661 | | 0.039 | | 1.194 | | 0.012 | | 1.146 | | 0.468 | | 0.805 | | 0.001 | | | sine oculis homeobox homolog 1 (Drosophila) |
| SLUG | 1.081 | | 0.537 | | 1.125 | | 0.255 | | 1.147 | | 0.286 | | 0.785 | | 0.002 | | | snail homolog 2 (Drosophila) (SNAI2) |
| SMARCB1 | 1.104 | | 0.504 | | 1.225 | | 0.007 | | 1.054 | | 0.538 | | 0.913 | | 0.149 | | | SWI/SNF related, matrix associated, actin dependent regulator of chromatin, subfamily b, member 1 |
| SMARCC1 | 1.062 | | 0.553 | | 1.416 | | 0.270 | | 1.454 | | 0.009 | | 1.106 | | 0.016 | | | SWI/SNF related, matrix associated, actin dependent regulator of chromatin, subfamily c, member 1 |
| SNAPC4 | 0.922 | | 0.472 | | 1.088 | | 0.639 | | 1.210 | | 0.139 | | 1.290 | | 0.002 | | | small nuclear RNA activating complex, polypeptide 4, 190kD |
| SNAPC5 | 1.002 | | 0.955 | | 1.016 | | 0.837 | | 1.211 | | 0.050 | | 1.027 | | 0.565 | | | small nuclear RNA activating complex, polypeptide 5, 19kDa |
| SOX5 | 0.903 | | 0.154 | | 1.002 | | 0.980 | | 0.716 | | 0.041 | | 0.847 | | 0.005 | | | SRY (sex determining region Y)-box 5 |
| SOX9 | 0.879 | | 0.111 | | 0.783 | | 0.160 | | 0.971 | | 0.468 | | 0.951 | | 0.477 | | | SRY (sex determining region Y)-box 9 |
| SP4 | 0.964 | | 0.583 | | 1.249 | | 0.058 | | 0.878 | | 0.386 | | 0.953 | | 0.434 | | | Sp4 transcription factor |
| SPIB | 0.947 | | 0.618 | | 1.316 | | 0.093 | | 1.054 | | 0.595 | | 0.891 | | 0.048 | | | Spi-B transcription factor (Spi-1/PU.1 related) |
| SOX general | 0.828 | | 0.105 | | 0.893 | | 0.330 | | 0.961 | | 0.692 | | 0.827 | | 0.014 | | | sex determining region Y type genes |
| SSX1 | 1.306 | | 0.035 | | 1.022 | | 0.612 | | 1.058 | | 0.236 | | 1.299 | | 0.013 | | | synovial sarcoma, X breakpoint 1 |
| SSX2 | 1.228 | | 0.003 | | 0.981 | | 0.867 | | 1.091 | | 0.318 | | 1.373 | | 4.86E-04 | | | synovial sarcoma, X breakpoint 2 |
| STAT6 | 0.824 | | 0.366 | | 1.240 | | 0.174 | | 1.037 | | 0.436 | | 0.813 | | 0.020 | | | signal transducer and activator of transcription 6, interleukin-4 induced |
| TADA2L | 1.257 | | 0.404 | | 1.060 | | 0.356 | | 0.992 | | 0.951 | | 0.858 | | 0.019 | | | transcriptional adaptor 2 (ADA2 homolog, yeast)-like |
| TADA3L | 0.899 | | 0.335 | | 1.103 | | 0.314 | | 0.824 | | 0.333 | | 1.085 | | 0.102 | | | transcriptional adaptor 3 (ADA3 homolog, yeast)-like |
| TAF1B | 0.608 | | 0.068 | | 0.960 | | 0.735 | | 1.013 | | 0.913 | | 1.253 | | 0.240 | | | TATA box binding protein (TBP)-associated factor, RNA polymerase I, B, 63kD |
| TAF2E | 1.011 | | 0.883 | | 0.946 | | 0.919 | | 1.299 | | 0.084 | | 0.991 | | 0.765 | | | TAF6 RNA polymerase II, TATA box binding protein (TBP)-associated factor, 80kDa |
| TAF2G | 1.260 | | 0.030 | | 0.874 | | 0.196 | | 0.926 | | 0.432 | | 0.951 | | 0.130 | | | TAF9 RNA polymerase II, TATA box binding protein (TBP)-associated factor, 32kDa |
| TBPL1 | 0.908 | | 0.484 | | 0.802 | | 0.107 | | 0.944 | | 0.681 | | 0.956 | | 0.567 | | | TBP-like 1 |
| TBX1 | 0.820 | | 0.010 | | 0.802 | | 0.105 | | 0.863 | | 0.199 | | 1.143 | | 0.053 | | | T-box 1 |
| TBX18 | 1.197 | | 0.033 | | 1.106 | | 0.047 | | 1.281 | | 0.034 | | 0.869 | | 0.203 | | | T-box 18 |
| TBX19 | 1.023 | | 0.493 | | 1.200 | | 0.191 | | 0.888 | | 0.484 | | 1.009 | | 0.897 | | | T-box 19 |
| TBX22 | 1.237 | | 0.019 | | 1.196 | | 0.511 | | 1.239 | | 0.079 | | 1.094 | | 0.539 | | | T-box 22 |
| TBX5 | 0.967 | | 0.757 | | 0.888 | | 0.055 | | 0.955 | | 0.367 | | 1.201 | | 0.017 | | | T-box 5 (Holt-Oram syndrome) |
| TCEAL1 | 1.402 | | 0.065 | | 1.292 | | 0.007 | | 1.252 | | 0.042 | | 1.086 | | 0.053 | | | transcription elongation factor A (SII)-like 1 |
| TCF21 | 0.993 | | 0.950 | | 1.231 | | 0.056 | | 1.114 | | 0.581 | | 0.887 | | 0.055 | | | Transcription factor 21 |
| TCF7L2 | 1.186 | | 0.094 | | 1.200 | | 0.020 | | 1.189 | | 0.212 | | 0.736 | | 0.008 | | | transcription factor 7-like 2 (T-cell specific, HMG-box) |
| TCFL1 | 1.115 | | 0.124 | | 0.829 | | 0.023 | | 1.092 | | 0.475 | | 1.039 | | 0.410 | | | transcription factor-like 1 |
| TEAD1 | 0.789 | | 0.327 | | 1.056 | | 0.743 | | 0.945 | | 0.583 | | 0.994 | | 0.920 | | | TEA domain family member 1 (SV40 transcriptional enhancer factor) |
| TEAD4 | 0.754 | | 0.028 | | 0.944 | | 0.598 | | 0.737 | | 0.149 | | 1.067 | | 0.408 | | | TEA domain family member 4 |
| TFAP2B | 1.121 | | 0.102 | | 1.330 | | 0.029 | | 0.835 | | 0.372 | | 0.786 | | 0.014 | | | transcription factor AP-2 beta (activating enhancer binding protein 2 beta) |
| THRB | 0.764 | | 0.085 | | 1.022 | | 0.709 | | 1.035 | | 0.785 | | 0.899 | | 0.031 | | | thyroid hormone receptor, beta (erythroblastic leukemia viral (v-erb-a) oncogene homolog 2, avian) |
| TIEG | 1.313 | | 0.023 | | 1.246 | | 0.097 | | 0.943 | | 0.414 | | 1.034 | | 0.614 | | | TGFB inducible early growth response |
| TIEG2 | 0.958 | | 0.704 | | 0.935 | | 0.366 | | 0.825 | | 0.083 | | 0.837 | | 0.085 | | | TGFB inducible early growth response 2 |
| TIMELESS | 1.377 | | 0.075 | | 1.283 | | 0.006 | | 0.985 | | 0.851 | | 0.889 | | 0.107 | | | timeless homolog (Drosophila) |
| TITF1 | 0.760 | | 0.018 | | 1.078 | | 0.506 | | 0.838 | | 0.364 | | 1.166 | | 0.150 | | | thyroid transcription factor 1 |
| TMF1 | 1.161 | | 0.154 | | 1.082 | | 0.498 | | 0.779 | | 0.037 | | 1.073 | | 0.437 | | | TATA element modulatory factor 1 |
| TNRC12 | 0.785 | | 0.016 | | 0.987 | | 0.730 | | 0.946 | | 0.187 | | 0.978 | | 0.467 | | | trinucleotide repeat containing 12 |
| TNRC5 | 1.265 | | 0.004 | | 0.957 | | 0.685 | | 0.795 | | 0.313 | | 1.162 | | 0.001 | | | trinucleotide repeat containing 5 |
| TNRC6 | 1.292 | | 0.137 | | 1.035 | | 0.686 | | 1.028 | | 0.804 | | 1.031 | | 0.460 | | | trinucleotide repeat containing 6 |
| TNRC9 | 0.778 | | 0.082 | | 0.895 | | 0.029 | | 0.947 | | 0.772 | | 1.057 | | 0.035 | | | trinucleotide repeat containing 9 |
| TP53 | 1.096 | | 0.096 | | 1.168 | | 0.332 | | 1.227 | | 0.078 | | 0.980 | | 0.722 | | | tumor protein p53 (Li-Fraumeni syndrome) |
| TRIM15 | 1.239 | | 0.924 | | 0.838 | | 0.214 | | 0.953 | | 0.378 | | 1.044 | | 0.219 | | | tripartite motif-containing 15 |
| TRIP11 | 0.968 | | 0.848 | | 0.912 | | 0.150 | | 0.976 | | 0.860 | | 1.203 | | 0.001 | | | thyroid hormone receptor interactor 11 |
| TRIP15 | 1.210 | | 0.087 | | 1.117 | | 0.041 | | 0.990 | | 0.898 | | 0.789 | | 0.011 | | | thyroid receptor interacting protein 15 |
| USF1 | 1.055 | | 0.224 | | 1.256 | | 0.141 | | 0.910 | | 0.455 | | 0.954 | | 0.260 | | | upstream transcription factor 1 |
| VAX2 | 0.747 | | 0.101 | | 1.219 | | 0.046 | | 0.945 | | 0.184 | | 1.090 | | 0.450 | | | homeobox protein VAX2 |
| VDR | 1.239 | | 0.055 | | 0.923 | | 0.451 | | 0.796 | | 0.137 | | 1.051 | | 0.186 | | | vitamin D (1,25- dihydroxyvitamin D3) receptor |
| VENTX2 | 0.976 | | 0.782 | | 0.756 | | 0.007 | | 0.786 | | 0.181 | | 1.056 | | 0.244 | | | VENT-like homeobox 2 |
| VSX1 | 0.850 | | 0.004 | | 0.936 | | 0.575 | | 0.823 | | 0.250 | | 1.008 | | 0.902 | | | visual system homeobox 1 homolog, CHX10-like (zebrafish) |
| WHN | 1.706 | | 0.066 | | 0.959 | | 0.417 | | 0.893 | | 0.487 | | 0.893 | | 0.226 | | | winged-helix nude |
| XBP1 | 1.228 | | 0.227 | | 0.922 | | 0.513 | | 0.770 | | 0.390 | | 0.719 | | 0.008 | | | X-box binding protein 1 |
| ZF5128 | 0.915 | | 0.187 | | 0.819 | | 0.146 | | 0.831 | | 0.255 | | 0.968 | | 0.560 | | | zinc finger protein |
| ZFHX1B | 0.774 | | 0.107 | | 1.093 | | 0.329 | | 0.907 | | 0.478 | | 0.784 | | 0.015 | | | zinc finger homeobox 1B |
| ZFP103 | 0.962 | | 0.721 | | 0.820 | | 0.152 | | 0.956 | | 0.774 | | 1.154 | | 0.040 | | | zinc finger protein 103 homolog (mouse) |
| ZFP106 | 0.790 | | 0.017 | | 0.777 | | 0.147 | | 0.949 | | 0.707 | | 1.248 | | 0.006 | | | zinc finger protein 106 |
| ZFP161 | 0.870 | | 0.873 | | 0.890 | | 0.285 | | 0.827 | | 0.104 | | 1.048 | | 0.251 | | | zinc finger protein 161 homolog (mouse) |
| ZFP289 | 0.780 | | 0.202 | | 0.829 | | 0.029 | | 0.910 | | 0.568 | | 1.075 | | 0.287 | | | zinc finger protein 289, ID1 regulated |
| ZIC4 | 1.301 | | 0.124 | | 1.101 | | 0.304 | | 1.175 | | 0.095 | | 1.080 | | 0.195 | | | zinc family member 4 protein HZIC4 |
| ZID | 0.812 | | 0.023 | | 0.809 | | 0.321 | | 1.069 | | 0.592 | | 1.125 | | 0.018 | | | zinc finger protein with interaction domain |
| ZNF11B | 1.154 | | 0.290 | | 0.826 | | 0.040 | | 0.960 | | 0.484 | | 1.053 | | 0.039 | | | zinc finger protein 11b (KOX 2) |
| ZNF123 | 0.880 | | 0.188 | | 1.405 | | 0.130 | | 0.849 | | 0.308 | | 1.279 | | 0.005 | | | zinc finger protein 123 (HZF-1) |
| ZNF134 | 0.896 | | 0.487 | | 1.042 | | 0.519 | | 1.121 | | 0.413 | | 1.297 | | 0.058 | | | zinc finger protein 134 (clone pHZ-15) |
| ZNF140 | 0.815 | | 0.103 | | 0.908 | | 0.802 | | 0.751 | | 0.153 | | 1.065 | | 0.346 | | | zinc finger protein 140 (clone pHZ-39) |
| ZNF143 | 0.716 | | 0.025 | | 1.045 | | 0.627 | | 0.859 | | 0.157 | | 1.118 | | 0.041 | | | zinc finger protein 143 (clone pHZ-1) |
| ZNF165 | 0.959 | | 0.669 | | 0.986 | | 0.652 | | 0.854 | | 0.224 | | 1.210 | | 0.244 | | | zinc finger protein 165 |
| ZNF174 | 1.041 | | 0.648 | | 0.820 | | 0.244 | | 1.016 | | 0.630 | | 1.426 | | 0.006 | | | zinc finger protein 174 |
| ZNF175 | 1.432 | | 0.006 | | 1.070 | | 0.377 | | 0.859 | | 0.287 | | 0.948 | | 0.455 | | | zinc finger protein 175 |
| ZNF195 | 0.852 | | 0.168 | | 0.773 | | 0.116 | | 0.922 | | 0.333 | | 1.035 | | 0.463 | | | zinc finger protein 195 |
| ZNF219 | 1.146 | | 0.241 | | 0.935 | | 0.449 | | 0.810 | | 0.291 | | 0.961 | | 0.242 | | | zinc finger protein 219 |
| ZNF230 | 1.286 | | 0.010 | | 0.993 | | 0.971 | | 0.952 | | 0.659 | | 0.994 | | 0.894 | | | zinc finger protein 230 |
| ZNF239 | 0.976 | | 0.769 | | 0.885 | | 0.400 | | 1.298 | | 0.080 | | 0.821 | | 0.008 | | | zinc finger protein (C2H2) homologous to mouse MOK-2 (aka HOK-2, MOK2) |
| ZNF25 | 0.817 | | 0.010 | | 0.838 | | 0.061 | | 0.920 | | 0.351 | | 1.010 | | 0.810 | | | zinc finger protein 25 (KOX 19) |
| ZNF258 | 0.921 | | 0.321 | | 0.846 | | 0.887 | | 0.809 | | 0.191 | | 1.287 | | 0.027 | | | zinc finger protein 258 |
| ZNF273 | 1.082 | | 0.239 | | 0.753 | | 0.146 | | 0.807 | | 0.169 | | 1.020 | | 0.737 | | | zinc finger protein 273 (HZF9) |
| ZNF3 | 0.988 | | 0.848 | | 1.270 | | 0.306 | | 1.060 | | 0.966 | | 1.092 | | 0.060 | | | zinc finger protein 3 (A8-51) |
| ZNF306 | 0.722 | | 0.059 | | 0.943 | | 0.287 | | 0.999 | | 0.981 | | 1.055 | | 0.385 | | | zinc finger protein 306 |
| ZNF31 | 0.818 | | 0.162 | | 0.805 | | 0.179 | | 0.826 | | 0.090 | | 1.002 | | 0.956 | | | zinc finger protein 31 (KOX 29) |
| ZNF36 | 0.738 | | 0.004 | | 0.928 | | 0.434 | | 0.956 | | 0.689 | | 1.084 | | 0.221 | | | zinc finger protein 36 (KOX 18) |
| ZNF73 | 0.891 | | 0.197 | | 0.770 | | 0.230 | | 0.780 | | 0.049 | | 1.203 | | 0.115 | | | zinc finger protein 73 (Cos12) |
| ZNF75A | 1.005 | | 0.953 | | 0.808 | | 0.228 | | 1.159 | | 0.275 | | 1.325 | | 1.07E-04 | | | zinc finger protein 75a |
| ZNF76 | 2.205 | | 0.016 | | 1.127 | | 0.598 | | 1.204 | | 0.534 | | 0.728 | | 0.022 | | | zinc finger protein 76 (expressed in testis) |
| ZNF85 | 0.893 | | 0.255 | | 0.741 | | 0.127 | | 0.712 | | 0.187 | | 1.169 | | 0.135 | | | zinc finger protein 85 (HPF4, HTF1) |
| ZNF9 | 0.816 | | 0.024 | | 1.104 | | 0.357 | | 0.995 | | 0.963 | | 1.012 | | 0.599 | | | zinc finger protein 9 (a cellular retroviral nucleic acid binding protein) |
| ZNF93 | 0.847 | | 0.018 | | 0.739 | | 0.060 | | 1.131 | | 0.320 | | 1.153 | | 0.002 | | | zinc finger protein 93 (HTF34) |
| ZNF-kaiso | 0.843 | | 0.315 | | 0.896 | | 0.306 | | 1.005 | | 0.962 | | 0.828 | | 0.010 | | | Kaiso |
| ZNF-U69274 | 0.974 | | 0.774 | | 0.803 | | 0.284 | | 0.791 | | 0.225 | | 1.053 | | 0.254 | | | zinc finger protein |
| ZXDA/B | 0.783 | | 0.156 | | 1.076 | | 0.240 | | 1.191 | | 0.103 | | 0.933 | | 0.130 | | | zinc finger, X-linked, duplicated B |
