# Supplementary material for: Large Scale Gene Expression Profiles of Regenerating Inner Ear Sensory Epithelia
Source: PLoS One. 2007 Jun 13;2(6):e525. doi: 10.1371/journal.pone.0000525 (PMC1888727; doi:10.1371/journal.pone.0000525)
Supplement: Table S8 — Cochlea Laser Differential Expression>1.2-fold and P< = 0.05 (0.07 MB PDF) [file pone.0000525.s009.pdf]

Gene ID	Entrez	30min Fold change	30min P-value	1hr Fold Change	1hr P-value	2hr Fold Change	2hr P-value	3hr Fold Change	3hr P-value	Updated syml	Updated description
CITED2	10370	1.352	0.018	0.974	0.795	1.13	0.207	1.315	0.017	CITED2	Cbp/p300-interacting transactivator, with Glu
HOXA4	3201	1.307	0.007	0.96	0.711	1.113	0.276	1.276	0.006	HOXA4	homeobox A4
SSX2	10214	1.228	0.003	0.981	0.867	1.091	0.318	1.373	4.86E-04	SSX2	synovial sarcoma, X breakpoint 2 /// synovial
SSX1	652630	1.306	0.035	1.022	0.612	1.058	0.236	1.299	0.013	SSX1	synovial sarcoma, X breakpoint 1 /// similar to
HSAJ2425	55566	1.109	0.372	1.192	0.689	1.287	0.231	1.313	3.89E-04	HSAJ2425	p65 protein
SNAPC4	6621	0.922	0.472	1.088	0.639	1.21	0.139	1.29	0.002	SNAPC4	Small nuclear RNA activating complex, polyp
JUND	3727	0.994	0.962	1.161	0.295	1.166	0.223	1.308	0.019	JUND	jun D proto-oncogene
DKFZp547H2	56917	0.957	0.767	0.965	0.734	1.161	0.254	1.497	0.032	MEIS3	Meis1, myeloid ecotropic viral integration site
ZNF75A	7627	1.005	0.953	0.808	0.228	1.159	0.275	1.325	1.07E-04	ZNF75A	Zinc finger protein 75a
MADH2	4087	1.068	0.62	1.086	0.574	1.152	0.064	1.227	0.023	SMAD2	SMAD family member 2 /// mothers against d
LOC57209	57209	1.043	0.764	0.767	0.298	1.149	0.251	1.21	0.012	ZNF248	Zinc finger protein 248
CDX2	1045	0.943	0.436	0.997	0.962	1.115	0.46	1.226	0.001	CDX2	caudal type homeobox transcription factor 2
HOXD8	3234	0.769	0.251	1.021	0.744	1.11	0.372	1.272	0.002	HOXD8	Homeobox D8
KIAA0173	9654	0.991	0.845	1.054	0.432	1.089	0.705	1.247	3.33E-04	TTLL4	Tubulin tyrosine ligase-like family, member 4
BCL11A	53335	1.003	0.959	0.69	0.166	1.088	0.382	1.197	0.005	BCL11A	B-cell CLL/lymphoma 11A (zinc finger prote
NFIC	4782	1.383	0.097	0.793	0.312	1.021	0.861	1.192	0.033	NFIC	nuclear factor I/C (CCAAT-binding transcript
FLJ21603	79818	0.714	0.065	1.111	0.342	1.016	0.873	1.317	0.006	ZNF552	Zinc finger protein 552
ZNF174	7727	1.041	0.648	0.82	0.244	1.016	0.63	1.426	0.006	ZNF174	zinc finger protein 174
HMG20B	10362	1.099	0.036	0.982	0.814	0.996	0.91	1.198	0.002	HMG20B	high-mobility group 20B
AF5Q31	27125	1.377	0.425	1.133	0.47	0.992	0.954	1.289	0.008	AFF4	AF4/FMR2 family, member 4
BHLHB3	79365	0.865	0.263	0.805	0.069	0.978	0.696	1.222	0.016	BHLHB3	basic helix-loop-helix domain containing, clas
TRIP11	9321	0.968	0.848	0.912	0.15	0.976	0.86	1.203	0.001	TRIP11	thyroid hormone receptor interactor 11
ESR1	2099	0.724	0.095	0.905	0.504	0.97	0.813	1.82	0.001	ESR1	estrogen receptor 1
CHD3	1107	1.53	0.213	0.961	0.413	0.963	0.546	1.209	0.002	CHD3	Chromodomain helicase DNA binding protein
TBX5	6910	0.967	0.757	0.888	0.055	0.955	0.367	1.201	0.017	TBX5	T-box 5
KIAA0014	9684	1.811	0.055	0.866	0.394	0.953	0.818	1.288	0.051	LRRC14	Leucine rich repeat containing 14
KIAA0998	23093	0.929	0.388	1.111	0.526	0.948	0.472	1.25	0.002	TTLL5	Tubulin tyrosine ligase-like family, member 5
HOXD11	3237	0.863	0.217	0.857	0.181	0.945	0.569	1.2	0.001	HOXD11	homeobox D11
FOXH1	8928	0.915	0.411	0.916	0.362	0.936	0.342	1.282	0.001	FOXH1	Forkhead box H1
CBFA2T3	863	1.083	0.488	1.114	0.33	0.924	0.247	1.226	0.007	CBFA2T3	core-binding factor, runt domain, alpha subun
CXorf6	10046	0.764	0.219	0.926	0.714	0.906	0.258	1.234	0.005	CXorf6	chromosome X open reading frame 6
HOXC10	3226	0.857	0.22	0.984	0.748	0.878	0.249	1.26	0.017	HOXC10	homeobox C10
CEZANNE	56957	0.782	0.06	0.916	0.307	0.866	0.077	1.24	0.02	OTUD7B	OTU domain containing 7B /// Zinc finger, A
FLJ11186	55320	0.562	0.136	0.588	0.294	0.855	0.345	2.043	0.007	C14orf106	Chromosome 14 open reading frame 106
FOG2	23414	1.061	0.628	1.339	0.067	0.85	0.723	1.385	0.011	ZFPM2	Friend of GATA2 /// zinc finger protein, mult
ZNF123	7677	0.88	0.188	1.405	0.13	0.849	0.308	1.279	0.005	ZNF123	zinc finger protein 123
FLJ22332	253461	0.885	0.216	0.848	0.084	0.815	0.133	1.271	6.28E-06	ZBTB38	zinc finger and BTB domain containing 38
FLJ11191	55786	0.853	0.151	0.884	0.496	0.775	0.156	1.236	0.024	ZNF415	zinc finger protein 415
ID3	3399	0.911	0.455	1.005	0.964	0.812	0.181	1.223	0.002	ID3	inhibitor of DNA binding 3, dominant negativ
ZNF258	9204	0.921	0.321	0.846	0.887	0.809	0.191	1.287	0.027	ZMYM6	zinc finger, MYM-type 6
ZFP106	64397	0.79	0.017	0.777	0.147	0.949	0.707	1.248	0.006	ZFP106	zinc finger protein 106 homolog (mouse)
HKR2	342945	0.801	0.001	0.969	0.643	0.923	0.424	1.258	0.013	HKR2	GLI-Kruppel family member HKR2
EPAS1	2034	0.626	0.039	0.869	0.089	0.766	0.153	1.299	0.009	EPAS1	endothelial PAS domain protein 1
PRDM13	59336	0.749	0.002	1.146	0.41	0.791	0.514	2.048	0.006	PRDM13	PR domain containing 13
HSU90653	29800	0.644	0.064	0.806	0.03	0.791	0.23	1.217	0.011	ZDHHC1	zinc finger, DHHC-type containing 1
KIAA1190	92999	0.829	0.04	0.834	0.088	0.801	0.008	1.25	0.003	ZNF651	zinc finger protein 651
TCEAL1	9338	1.402	0.065	1.292	0.007	1.252	0.042	1.086	0.053	TCEAL1	transcription elongation factor A (SII)-like 1
P1P373C6	387032	1.282	0.034	0.975	0.795	1.212	0.004	0.922	0.166	ZNF307	zinc finger protein 307
NR2F2	7026	1.233	0.005	1.152	0.01	1.212	0.017	0.924	0.154	NR2F2	nuclear receptor subfamily 2, group F, membe

FLJ12517	116841	1.333	0.052	0.996	0.92	1.239	0.026	0.953	0.177	C1orf142	Chromosome 1 open reading frame 142
TBX18	9096	1.197	0.033	1.106	0.047	1.281	0.034	0.869	0.203	TBX18	T-box 18
ONECUT2	9480	1.099	0.035	1.288	0.096	1.226	0.002	0.93	0.128	ONECUT2	one cut domain, family member 2
SMARCC1	6599	1.062	0.553	1.416	0.27	1.454	0.009	1.106	0.016	SMARCC1	SWI/SNF related, matrix associated, actin dep
KIAA0943	23192	1.157	0.242	1.393	0.14	1.242	0.015	1.025	0.715	ATG4B	ATG4 autophagy related 4 homolog B (S. cer
M96	22823	0.783	0.062	1.139	0.32	1.233	0.022	1.006	0.858	MTF2	Metal response element binding transcription
DLX5	1749	0.968	0.644	0.951	0.413	1.257	0.032	0.854	0.027	DLX5	distal-less homeobox 5
NCOR2	9612	1.178	0.077	1.114	0.347	1.291	0.034	1.106	0.194	NCOR2	Nuclear receptor co-repressor 2
NFIB	4781	1.146	0.476	1.179	0.196	1.257	0.037	1.086	0.021	NFIB	nuclear factor I/B
RBBP9	10741	1.001	0.986	0.933	0.563	1.309	0.045	1.129	0.008	RBBP9	retinoblastoma binding protein 9
SNAPC5	10302	1.002	0.955	1.016	0.837	1.211	0.05	1.027	0.565	SNAPC5	small nuclear RNA activating complex, polyp
MEOX2	4223	1.066	0.275	1.057	0.531	1.255	0.052	0.975	0.698	MEOX2	mesenchyme homeobox 2
NR2F6	2063	1.22	0.015	1.192	0.027	1.14	0.424	0.858	0.002	NR2F6	nuclear receptor subfamily 2, group F, membe
NEUROG1	4762	1.976	0.045	1.244	0.036	1.014	0.726	1.117	0.006	NEUROG1	neurogenin 1
LDB1	8861	1.295	0.001	1.287	0.028	0.948	0.54	0.886	0.014	LDB1	LIM domain binding 1
NR1H3	10062	0.991	0.935	1.207	0.041	1.491	0.24	0.847	0.009	NR1H3	nuclear receptor subfamily 1, group H, memb
PER2	8864	1.144	0.546	1.214	0.036	1.236	0.308	1.138	0.069	PER2	Period homolog 2 (Drosophila)
MYT2	8827	0.942	0.501	1.207	0.044	1.136	0.041	1.03	0.287	MYT2	Myelin transcription factor 2
IRLB	10260	0.965	0.713	1.29	0.007	1.123	0.6	0.891	0.038	DENND4A	DENN/MADD domain containing 4A
NR2C2	7182	1.273	0.156	1.233	0.023	1.08	0.262	0.957	0.207	NR2C2	nuclear receptor subfamily 2, group C, memb
SMARCB1	6598	1.104	0.504	1.225	0.007	1.054	0.538	0.913	0.149	SMARCB1	SWI/SNF related, matrix associated, actin dep
PBX2	5089	0.96	0.788	1.209	0.025	0.994	0.949	0.937	0.125	PBX2	pre-B-cell leukemia transcription factor 2
TIMELESS	8914	1.377	0.075	1.283	0.006	0.985	0.851	0.889	0.107	TIMELESS	timeless homolog (Drosophila)
PAX3	5077	1.289	0.319	1.325	0.048	0.97	0.681	1.003	0.931	PAX3	paired box gene 3 (Waardenburg syndrome 1)
VAX2	25806	0.747	0.101	1.219	0.046	0.945	0.184	1.09	0.45	VAX2	Ventral anterior homeobox 2
CLOCK	9575	1.597	0.286	1.292	0.05	0.929	0.514	0.865	0.144	CLOCK	clock homolog (mouse)
MEF2A	4205	0.881	0.276	1.219	0.052	0.752	0.497	0.912	0.096	MEF2A	MADS box transcription enhancer factor 2, po
DFKZP434E0	112398	1.407	0.002	1.064	0.586	1.411	0.074	1.09	0.22	EGLN2	egl nine homolog 2 (C. elegans)
TBX22	50945	1.237	0.019	1.196	0.511	1.239	0.079	1.094	0.539	TBX22	T-box 22
PER1	5187	1.243	0.035	1.096	0.249	1.236	0.109	0.991	0.862	PER1	period homolog 1 (Drosophila)
NFATC2	4773	1.253	0.015	0.786	0.406	1.249	0.203	1.16	0.156	NFATC2	nuclear factor of activated T-cells, cytoplasmi
HOXD1	3231	1.289	0.044	0.941	0.494	1.272	0.303	1.061	0.077	HOXD1	homeobox D1
FLJ22301	79894	1.357	0.039	1.035	0.581	1.183	0.332	1.059	0.126	ZNF672	zinc finger protein 672
MAFF	23764	1.273	0.01	1.155	0.05	1.175	0.067	0.964	0.463	MAFF	V-maf musculoaponeurotic fibrosarcoma onco
HEYL	26508	1.294	0.054	1.002	0.971	1.16	0.135	1.024	0.726	HEYL	hairy/enhancer-of-split related with YRPW m
FOXC2	2303	1.499	0.022	1.219	0.266	1.153	0.177	0.971	0.568	FOXC2	forkhead box C2 (MFH-1, mesenchyme forkh
KIAA0293	23316	1.27	0.013	1.201	0.159	1.139	0.371	0.916	0.033	CUTL2	cut-like 2 (Drosophila)
NPAS1	4861	1.289	0.001	1.05	0.356	1.128	0.234	1.015	0.625	NPAS1	neuronal PAS domain protein 1
BAZ2B	29994	1.2	0.02	0.873	0.042	1.12	0.401	1.058	0.343	BAZ2B	bromodomain adjacent to zinc finger domain,
EZH2	2146	1.203	0.052	1.016	0.83	1.119	0.031	0.991	0.866	EZH2	enhancer of zeste homolog 2 (Drosophila)
PRDM11	56981	1.725	0.016	1.1	0.149	1.098	0.6	0.974	0.663	PRDM11	PR domain containing 11
MYB	4602	1.219	0.039	0.933	0.473	1.08	0.339	1.016	0.781	MYB	V-myb myeloblastosis viral oncogene homolo
FOS	2353	1.501	1.93E-04	1.132	0.616	1.079	0.368	1.136	0.167	FOS	v-fos FBJ murine osteosarcoma viral oncogen
RARG	5916	1.318	0.015	1.193	0.269	1.061	0.801	1.017	0.643	RARG	retinoic acid receptor, gamma
SHOX2	6474	1.221	0.028	0.899	0.299	1.032	0.784	1.009	0.833	SHOX2	short stature homeobox 2
DKFZP434P1	26000	1.371	0.008	1.111	0.682	1.027	0.911	0.878	0.162	TBC1D10B	TBC1 domain family, member 10B
ELK1	2002	1.209	0.022	0.98	0.693	1.025	0.756	0.999	0.986	ELK1	ELK1, member of ETS oncogene family
CDX4	1046	1.41	0.021	0.945	0.54	1.015	0.858	0.912	0.086	CDX4	caudal type homeobox transcription factor 4
RELA	5970	1.241	0.008	1.138	0.425	1.002	0.965	0.936	0.086	RELA	v-rel reticuloendotheliosis viral oncogene hon
HOXC11	3227	1.869	0.045	1.274	0.104	0.986	0.83	0.848	0.011	HOXC11	homeobox C11
MEF2B	4207	1.356	1.94E-04	0.908	0.638	0.979	0.716	1.089	0.037	MEF2B	MADS box transcription enhancer factor 2, po

HSF4	3299	1.249	0.011	1.086	0.131	0.975	0.897	0.972	0.705	HSF4	heat shock transcription factor 4
HBOA	11143	1.225	0.006	1.099	0.282	0.971	0.727	0.983	0.625	MYST2	MYST histone acetyltransferase 2 /// MYST h
NHLH2	4808	1.757	0.037	0.944	0.725	0.971	0.828	0.883	0.175	NHLH2	nescient helix loop helix 2
SIM1	6492	1.206	0.013	1.043	0.585	0.969	0.548	0.992	0.885	SIM1	single-minded homolog 1 (Drosophila)
RNF4	6047	1.511	0.027	1.348	0.198	0.954	0.768	0.873	0.231	RNF4	ring finger protein 4
ZNF230	7773	1.286	0.01	0.993	0.971	0.952	0.659	0.994	0.894	ZNF230	Zinc finger protein 230
BCL11B	64919	1.225	0.046	0.873	0.316	0.95	0.46	0.989	0.787	BCL11B	B-cell CLL/lymphoma 11B (zinc finger protei
EGR1	1958	1.775	0.001	0.992	0.945	0.947	0.358	1.048	0.651	EGR1	early growth response 1
TIEG	7071	1.313	0.023	1.246	0.097	0.943	0.414	1.034	0.614	KLF10	Kruppel-like factor 10
RXRB	6257	1.399	0.015	0.99	0.857	0.936	0.258	0.889	0.01	RXRB	retinoid X receptor, beta
FLJ10759	55223	1.26	5.46E-05	1.042	0.45	0.926	0.357	1.143	0.012	TRIM62	tripartite motif-containing 62
TAF2G	6880	1.26	0.03	0.874	0.196	0.926	0.432	0.951	0.13	TAF9	TAF9 RNA polymerase II, TATA box binding
HSPC189	51545	1.469	0.039	0.994	0.912	0.918	0.061	1.028	0.42	ZNF581	zinc finger protein 581
GCMB	9247	1.273	0.02	1.047	0.484	0.915	0.357	1.028	0.731	GCM2	glial cells missing homolog 2 (Drosophila)
AF093680	29105	1.279	0.014	1.011	0.826	0.908	0.287	0.952	0.331	C16orf80	chromosome 16 open reading frame 80
BRF2	55290	1.461	0.049	0.77	0.296	0.895	0.458	1.033	0.776	BRF2	BRF2, subunit of RNA polymerase III transcr
MHC2TA	4261	1.361	0.05	0.918	0.567	0.887	0.321	1.062	0.447	CIITA	Class II, major histocompatibility complex, tr
EP300	2033	1.439	0.019	1.069	0.523	0.873	0.66	0.87	0.09	EP300	E1A binding protein p300
ELK4	2005	1.271	0.034	1.052	0.603	0.86	0.025	1.066	0.031	ELK4	ELK4, ETS-domain protein (SRF accessory p
ZNF175	7728	1.432	0.006	1.07	0.377	0.859	0.287	0.948	0.455	ZNF175	zinc finger protein 175
LOC51652	51652	1.229	0.036	1.015	0.828	0.839	0.081	1.105	0.07	VPS24	vacuolar protein sorting 24 homolog (S. cerev
ARNTL	406	1.276	0.046	1.059	0.572	0.813	0.151	1.018	0.696	ARNTL	aryl hydrocarbon receptor nuclear translocator
TNRC5	10695	1.265	0.004	0.957	0.685	0.795	0.313	1.162	0.001	TNRC5	trinucleotide repeat containing 5
MEIS3	56917	0.737	0.011	0.979	0.855	1.16	0.205	1.086	0.076	MEIS3	Meis1, myeloid ecotropic viral integration site
PRDM15	63977	0.814	0.037	1.316	0.06	1.149	0.448	0.853	0.032	PRDM15	PR domain containing 15 /// similar to PR dor
RFXANK	8625	0.742	0.048	1.005	0.94	1.134	0.127	0.867	0.004	RFXANK	regulatory factor X-associated ankyrin-contain
RARA	5914	0.741	0.006	1.047	0.426	1.127	0.372	1.06	0.24	RARA	retinoic acid receptor, alpha
PAF65A	10629	0.739	0.027	1.024	0.882	1.117	0.394	1.003	0.965	TAF6L	TAF6-like RNA polymerase II, p300/CBP-ass
RELB	5971	0.743	0.015	1.031	0.59	1.093	0.473	1.009	0.873	RELB	v-rel reticuloendotheliosis viral oncogene hon
KIAA1388	57567	0.823	0.003	0.847	0.066	1.083	0.367	1.031	0.484	ZNF319	zinc finger protein 319
ARC	23237	0.812	0.036	0.885	0.163	1.078	0.448	1.148	0.009	ARC	activity-regulated cytoskeleton-associated pro
ZID	10773	0.812	0.023	0.809	0.321	1.069	0.592	1.125	0.018	ZBTB6	Zinc finger and BTB domain containing 6
IRF1	3659	0.652	0.036	1.461	0.149	1.066	0.702	0.849	0.011	IRF1	interferon regulatory factor 1
OAZ	23090	0.792	0.014	0.806	0.484	1.029	0.576	1.05	0.402	ZNF423	zinc finger protein 423
CERD4	8110	0.827	0.01	0.853	0.014	1.005	0.912	1.06	0.275	DPF3	D4, zinc and double PHD fingers, family 3
ZNF9	7555	0.816	0.024	1.104	0.357	0.995	0.963	1.012	0.599	CNBP	CCHC-type zinc finger, nucleic acid binding p
ZNF36	7586	0.738	0.004	0.928	0.434	0.956	0.689	1.084	0.221	ZKSCAN1	zinc finger with KRAB and SCAN domains 1
PAX7	5081	0.762	0.003	0.979	0.805	0.951	0.715	0.936	0.52	PAX7	paired box gene 7
TNRC12	57634	0.785	0.016	0.987	0.73	0.946	0.187	0.978	0.467	EP400	E1A binding protein p400
KIAA0395	23051	0.722	0.037	0.834	0.246	0.934	0.344	1.186	0.032	ZHX3	Zinc fingers and homeoboxes 3
ZNF25	219749	0.817	0.01	0.838	0.061	0.92	0.351	1.01	0.81	ZNF25	zinc finger protein 25
ATF5	22809	0.767	0.026	0.704	0.113	0.918	0.352	0.953	0.658	ATF5	activating transcription factor 5
KIAA0535	9705	0.721	0.046	0.996	0.953	0.916	0.372	1.054	0.234	ST18	suppression of tumorigenicity 18 (breast carci
HR	55806	0.802	0.012	0.974	0.529	0.904	0.016	1.306	0.094	HR	hairless homolog (mouse)
HOX11	3195	0.713	0.009	1.015	0.879	0.894	0.166	0.98	0.506	TLX1	T-cell leukemia homeobox 1
DKFZP434B0	25851	0.788	0.019	1.103	0.586	0.874	0.384	0.852	0.02	DKFZP434B0	DKFZP434B0335 protein
TBX1	6899	0.82	0.01	0.802	0.105	0.863	0.199	1.143	0.053	TBX1	T-box 1
ZNF143	7702	0.716	0.025	1.045	0.627	0.859	0.157	1.118	0.041	ZNF143	zinc finger protein 143
LOC56930	4298	0.738	0.035	1.201	0.379	0.848	0.151	1.171	0.043	MLLT1	MLLT1 myeloid/lymphoid or mixed-lineage l
BHLHB2	8553	0.721	0.053	0.903	0.373	0.799	0.132	1.209	0.141	BHLHB2	basic helix-loop-helix domain containing, clas
TEAD4	7004	0.754	0.028	0.944	0.598	0.737	0.149	1.067	0.408	TEAD4	TEA domain family member 4

HCF2	29915	0.749	2.68E-04	1.009	0.894	0.8	0.258	1.076	0.173	HCFC2	host cell factor C2
TITF1	7080	0.76	0.018	1.078	0.506	0.838	0.364	1.166	0.15	TITF1	thyroid transcription factor 1
FLJ13659	80264	1.223	0.017	0.839	0.023	1.213	0.254	1.067	0.222	ZNF430	zinc finger protein 430
FLJ12827	79797	0.997	0.954	0.837	0.005	1.314	0.264	0.98	0.713	FLJ12827	Zinc finger protein 408
MAPK8IP1	9479	1.021	0.69	0.793	0.014	1.144	0.749	1.063	0.239	MAPK8IP1	mitogen-activated protein kinase 8 interacting
TCFL1	6944	1.115	0.124	0.829	0.023	1.092	0.475	1.039	0.41	VPS72	Vacuolar protein sorting 72 homolog (S. cerev
KIAA1528	113878	1.226	0.27	0.803	0.044	1.055	0.487	1.164	2.08E-04	DTX2	Deltex homolog 2 (Drosophila)
NR1I3	9970	1.039	0.586	0.803	0.035	1.047	0.817	1.124	0.077	NR1I3	Nuclear receptor subfamily 1, group I, membe
MADH5	4090	0.817	0.141	0.832	0.013	0.96	0.476	1.035	0.138	SMAD5	Mothers against decapentaplegic homolog 5
ZNF11B	7582	1.154	0.29	0.826	0.04	0.96	0.484	1.053	0.039	ZNF33B	zinc finger protein 33B
ZNF289	84364	0.78	0.202	0.829	0.029	0.91	0.568	1.075	0.287	ZNF289	zinc finger protein 289, ID1 regulated
BLZF1	8548	0.763	0.056	0.737	0.038	0.898	0.172	1.17	0.004	BLZF1	Basic leucine zipper nuclear factor 1 (JEM-1)
MAFG	4097	0.626	0.125	0.742	0.054	0.786	0.082	1.12	0.229	MAFG	v-maf musculoaponeurotic fibrosarcoma onco
VENTX2	27287	0.976	0.782	0.756	0.007	0.786	0.181	1.056	0.244	VENTX	VENT homeobox homolog (Xenopus laevis)
NFIL3	4783	2.15	0.008	1.319	0.009	0.801	0.051	0.968	0.473	NFIL3	nuclear factor, interleukin 3 regulated
ELK3	2004	0.923	0.244	0.991	0.916	0.794	0.005	0.901	0.116	ELK3	ELK3, ETS-domain protein (SRF accessory p
PPARD	5467	0.925	0.634	1.156	0.148	0.819	0.007	0.978	0.6	PPARD	peroxisome proliferative activated receptor, de
CBX8	57332	0.997	0.979	0.96	0.552	0.788	0.022	1.187	0.105	CBX8	chromobox homolog 8 (Pc class homolog, Dro
TMF1	7110	1.161	0.154	1.082	0.498	0.779	0.037	1.073	0.437	TMF1	TATA element modulatory factor 1
SOX5	6660	0.903	0.154	1.002	0.98	0.716	0.041	0.847	0.005	SOX5	SRY (sex determining region Y)-box 5
GAS41	8089	0.674	0.26	0.904	0.152	0.776	0.044	1.186	0.004	YEATS4	YEATS domain containing 4
MYF6	4618	0.944	0.386	0.985	0.59	0.786	0.045	1.141	0.046	MYF6	myogenic factor 6 (herculin)
ZNF73	7624	0.891	0.197	0.77	0.23	0.78	0.049	1.203	0.115	ZNF73	zinc finger protein 73
SETBP1	26040	0.989	0.825	0.922	0.966	0.824	0.051	0.984	0.695	SETBP1	SET binding protein 1
PAX8	7849	1.355	0.066	1.137	0.493	0.839	0.052	0.91	0.242	PAX8	paired box gene 8
GTF3A	2971	1.024	0.719	1.054	0.296	0.744	0.052	1.084	0.039	GTF3A	general transcription factor IIIA
CROC4	10485	0.623	0.234	0.994	0.957	1.249	0.019	0.825	0.103	CROC4	Transcriptional activator of the c-fos promoter
TCF7L2	6934	1.186	0.094	1.2	0.02	1.189	0.212	0.736	0.008	TCF7L2	Transcription factor 7-like 2 (T-cell specific, H
GTF2B	2959	1.219	0.145	1.268	0.037	1.063	0.263	0.799	0.007	GTF2B	general transcription factor IIB /// general tran
TFAP2B	7021	1.121	0.102	1.33	0.029	0.835	0.372	0.786	0.014	TFAP2B	transcription factor AP-2 beta (activating enha
SIX1	6495	0.661	0.039	1.194	0.012	1.146	0.468	0.805	0.001	SIX1	sine oculis homeobox homolog 1 (Drosophila
ZNF76	7629	2.205	0.016	1.127	0.598	1.204	0.534	0.728	0.022	ZNF76	zinc finger protein 76 (expressed in testis)
NKX2B	4821	1.447	0.024	1.29	0.164	1.135	0.467	0.782	0.021	NKX2-2	NK2 transcription factor related, locus 2 (Dro
FLJ13222	60685	1.213	0.011	1.142	0.092	1.045	0.246	0.796	0.003	ZFAND3	Zinc finger, AN1-type domain 3
HES7	84667	1.388	0.012	0.983	0.752	0.962	0.681	0.722	0.001	HES7	Hairy and enhancer of split 7 (Drosophila)
HES2	54626	1.454	0.003	1.029	0.751	0.89	0.221	0.675	0.011	HES2	hairy and enhancer of split 2 (Drosophila)
ZNF239	8187	0.976	0.769	0.885	0.4	1.298	0.08	0.821	0.008	ZNF239	zinc finger protein 239
RORB	6096	1.289	0.286	1.296	0.144	1.425	0.203	0.832	0.002	RORB	RAR-related orphan receptor B
NFX1	4799	1.171	0.277	1.184	0.039	1.223	0.217	0.766	0.001	NFX1	nuclear transcription factor, X-box binding 1
PLAGL1	5325	0.966	0.586	1.168	0.342	1.293	0.227	0.728	0.026	PLAGL1	Pleiomorphic adenoma gene-like 1
DNAJ	10294	1.032	0.87	1.219	0.302	1.305	0.3	0.74	0.015	DNAJA2	DnaJ (Hsp40) homolog, subfamily A, membe
FKHL18	2307	0.927	0.717	1.099	0.732	1.184	0.581	0.641	0.007	FKHL18	forkhead-like 18 (Drosophila)
SLUG	6591	1.081	0.537	1.125	0.255	1.147	0.286	0.785	0.002	SNAI2	snail homolog 2 (Drosophila)
POU1F1	5449	1.207	0.17	1.159	0.063	1.082	0.323	0.805	0.012	POU1F1	POU domain, class 1, transcription factor 1 (P
COPEB	1316	1.121	0.168	1.035	0.716	1.074	0.55	0.821	0.004	KLF6	Kruppel-like factor 6
ETV1	2115	1.419	0.286	1.035	0.895	1.067	0.833	0.54	0.005	ETV1	Ets variant gene 1
ILF2	3608	1.177	0.076	1.078	0.263	1.063	0.241	0.763	0.005	ILF2	interleukin enhancer binding factor 2, 45kDa /
LMO4	8543	0.823	0.331	0.979	0.786	1.061	0.638	0.766	0.004	LMO4	LIM domain only 4
STAT6	6778	0.824	0.366	1.24	0.174	1.037	0.436	0.813	0.02	STAT6	signal transducer and activator of transcription
CUTL1	1523	0.669	0.298	1.239	0.217	1.035	0.695	0.802	2.61E-04	CUTL1	Cut-like 1, CCAAT displacement protein (Dro
GCN5L1	2647	1.078	0.275	0.919	0.508	1.035	0.75	0.701	0.001	BLOC1S1	Biogenesis of lysosome-related organelles com

RBPSUHL	11317	1.232	0.536	1.122	0.188	1.033	0.432	0.813	0.008	RBPSUHL	recombining binding protein suppressor of hai
ZNF-kaiso	10009	0.843	0.315	0.896	0.306	1.005	0.962	0.828	0.01	ZBTB33	zinc finger and BTB domain containing 33
TRIP15	9318	1.21	0.087	1.117	0.041	0.99	0.898	0.789	0.011	COPS2	COP9 constitutive photomorphogenic homolo
CSEN	30818	0.897	0.234	1.104	0.508	0.985	0.917	0.801	0.007	KCNIP3	Kv channel interacting protein 3, calsenilin
KRML	9935	1.018	0.917	1.191	0.239	0.974	0.733	0.786	0.001	MAFB	v-maf musculoaponeurotic fibrosarcoma onco
SOX general	6736	0.828	0.105	0.893	0.33	0.961	0.692	0.827	0.014	SOX general	sex determining region Y type genes
PAX4	5078	1.228	0.096	1.043	0.448	0.921	0.299	0.755	0.025	PAX4	paired box gene 4
ZFHX1B	9839	0.774	0.107	1.093	0.329	0.907	0.478	0.784	0.015	ZFHX1B	Zinc finger homeobox 1b
HNF3B	3170	1.377	0.124	1.094	0.453	0.857	0.03	0.806	0.002	FOXA2	Forkhead box A2
IRF6	3664	0.962	0.789	1.087	0.791	0.631	0.06	0.696	0.012	IRF6	interferon regulatory factor 6
MAD	4084	1.035	0.675	1.151	0.463	0.791	0.132	0.807	0.041	MXD1	MAX dimerization protein 1 /// MAD protein
XBP1	7494	1.228	0.227	0.922	0.513	0.77	0.39	0.719	0.008	XBP1	X-box binding protein 1
HLX1	3142	0.809	0.038	1.033	0.853	1.096	0.567	0.706	0.014	HLX1	H2.0-like homeobox 1 (Drosophila)
PAX9	5083	1.339	0.073	1.137	0.058	0.812	0.043	0.778	8.71E-05	PAX9	paired box gene 9
GATA3	2625	0.817	0.027	0.836	0.52	0.771	0.043	0.694	2.16E-05	GATA3	GATA binding protein 3

ı/Asp-rich carboxy-terminal doı

l sarcoma, X breakpoint 4 /// syı
to synovial sarcoma, X breakpoı

ʻpeptide 4, 190kDa

te 1 homolog 3 (mouse)

decapentaplegic homolog 2

4
ein)
ɔtion factor)

ıss B, 3

ın 3

5

nit 2; translocated to, 3

ı20 domain containing 1 /// cellı

titype 2

ve helix-loop-helix protein

ɔer 2

pendent regulator of chromatin,
revisiae)
ı factor 2

peptide 5, 19kDa

ɔer 6

ɔer 3

ɔer 2
pendent regulator of chromatin,

)

ɔolypeptide A (myocyte enhance

ıic, calcineurin-dependent 2

cogene homolog F (avian)
notif-like
head 1)

, 2B

og (avian)
ne homolog

molog A, nuclear factor of kapp

ɔolypeptide B (myocyte enhance

histone acetyltransferase 2

ein)

ng protein (TBP)-associated fact

ription initiation factor, BRF1-l
ransactivator

protein 1)

visiae)
or-like

te 1 homolog 3 (mouse)
omain containing 15
ining protein

ssociated factor (PCAF)-associa
molog B, nuclear factor of kapp

otein

protein
1

inoma) (zinc finger protein)

leukemia (trithorax homolog, [illegible]
ass B, 2

g protein 1
:visiae) /// transcription factor-li

)er 3

)
ogene homolog G (avian)

protein 2)
delta
rosophila)

er
HMG-box)
nscription factor IIB
hancer binding protein 2 beta)
a)

osophila)

er 2

Pit1, growth hormone factor 1)

. /// interleukin enhancer binding

on 6, interleukin-4 induced
rosophila)
omplex-1, subunit 1

airless (Drosophila)-like

og subunit 2 (Arabidopsis)

ogene homolog B (avian)

ı (MAX-binding protein)
