# Supplementary material for: Large Scale Gene Expression Profiles of Regenerating Inner Ear Sensory Epithelia
Source: PLoS One. 2007 Jun 13;2(6):e525. doi: 10.1371/journal.pone.0000525 (PMC1888727; doi:10.1371/journal.pone.0000525)
Supplement: Table S9 — A listing of genes that exhibit similar patterns of expression across the four timecourses. utr-neo = utricle neomycin timecourse , coch-lsr = cochlea laser timecourse and so on. 1 = up-regulation, 0 = no change and −1 = down-regulation relative to untreated controls. (0.04 MB PDF) [file pone.0000525.s010.pdf]

SUPPLEMENTAL TABLE S9 .

Gene ID	UTR-NEO			COCH-NEO			UTR-LSR				COCH-LSR					
	**0hr**	**24hr**	**48hr**	**0hr**	**24hr**	**48hr**	**30min**	**1hr**	**2hr**	**3hr**	**30min**	**1hr**	**2hr**	**3hr**	**Updated symbo**	**Updated description**
TRIP15	-1	0	0	-1	0	0	0	1	0	0	0	0	0	-1	COPS2	COP9 constitutive photomorphogenic homolog s
GTF2F1	-1	0	0	-1	0	-1	--	--	--	--	--	--	--	--	GTF2F1	general transcription factor IIF, polypeptide 1, 74
IRF2	-1	0	0	-1	-1	-1	0	0	0	0	--	--	--	--	IRF2	Interferon regulatory factor 2
KIAA1041	-1	0	0	-1	0	0	--	--	--	--	--	--	--	--	FOXJ3	Forkhead box J3
NFE2L1	-1	0	0	-1	0	-1	--	--	--	--	--	--	--	--	NFE2L1	nuclear factor (erythroid-derived 2)-like 1
ZNF93	1	1	1	1	0	0	-1	0	0	1	0	0	0	0	ZNF93	Zinc finger protein 93
ZNF90	1	0	0	1	0	1	0	0	0	0	--	--	--	--	ZNF90	zinc finger protein 90
GIOT-2	0	-1	-1	0	-1	0	0	0	0	1	--	--	--	--	ZNF44	Zinc finger protein 44
TITF1	0	-1	0	0	-1	0	0	0	0	0	-1	0	0	0	TITF1	thyroid transcription factor 1
MAPK8IP1	1	-1	0	0	-1	0	0	0	0	0	0	-1	0	0	MAPK8IP1	mitogen-activated protein kinase 8 interacting pr
TAF2H	0	0	-1	0	-1	-1	0	0	0	0	--	--	--	--	TAF10	TAF10 RNA polymerase II, TATA box binding pr
NR1H3	0	0	-1	0	0	-1	0	1	0	0	0	1	0	0	NR1H3	nuclear receptor subfamily 1, group H, member 3
FHL1	0	0	-1	0	0	-1	0	0	0	1	--	--	--	--	FHL1	four and a half LIM domains 1
HNF3A	0	1	-1	0	0	-1	0	0	0	0	--	--	--	--	FOXA1	Forkhead box A1
MYCBP	0	0	1	0	-1	1	0	0	0	1	--	--	--	--	MYCBP	c-myc binding protein
BCL11A	0	0	1	0	0	1	0	0	0	1	0	0	0	1	BCL11A	B-cell CLL/lymphoma 11A (zinc finger protein)
CTNNB1	0	0	1	0	0	1	--	--	--	--	--	--	--	--	CTNNB1	catenin (cadherin-associated protein), beta 1, 88
ZF5128	0	0	1	0	0	1	--	--	--	--	0	0	0	0	ZNF324	Zinc finger protein 324
TBX15	1	0	1	0	0	1	0	0	0	1	--	--	--	--	TBX15	T-box 15
ZNF79	1	0	1	0	0	1	0	0	0	0	--	--	--	--	ZNF79	zinc finger protein 79
LOC57209	1	0	1	0	1	1	0	0	0	1	0	0	0	1	ZNF248	Zinc finger protein 248
ZID	--	--	--	1	0	0	-1	0	0	1	-1	0	0	0	ZBTB6	Zinc finger and BTB domain containing 6
FLJ12517	--	--	--	--	--	--	1	0	0	0	1	0	1	0	C1orf142	Chromosome 1 open reading frame 142
NR1H3	0	0	-1	0	0	-1	0	1	0	0	0	1	0	0	NR1H3	nuclear receptor subfamily 1, group H, member 3
MYT2	--	--	--	0	-1	0	0	1	-1	0	0	1	0	0	MYT2	Myelin transcription factor 2
JUND	0	0	0	0	-1	-1	0	0	0	1	0	0	0	1	JUND	jun D proto-oncogene
KIAA0173	1	0	0	-1	0	0	0	0	0	1	0	0	0	1	TTLL4	Tubulin tyrosine ligase-like family, member 4
HOXD8	1	0	0	0	1	0	0	0	0	1	0	0	0	1	HOXD8	Homeobox D8
BCL11A	0	0	1	0	0	1	0	0	0	1	0	0	0	1	BCL11A	B-cell CLL/lymphoma 11A (zinc finger protein)
LOC57209	1	0	1	0	1	1	0	0	0	1	0	0	0	1	ZNF248	Zinc finger protein 248
ZNF75A	--	--	--	1	0	0	0	0	0	1	0	0	0	1	ZNF75A	Zinc finger protein 75a
FLJ11186	--	--	--	0	0	1	0	0	0	1	0	0	0	1	C14orf106	Chromosome 14 open reading frame 106
TBX5	--	--	--	0	0	1	0	0	0	1	0	0	0	1	TBX5	T-box 5
