# Supplementary material for: Large Scale Gene Expression Profiles of Regenerating Inner Ear Sensory Epithelia
Source: PLoS One. 2007 Jun 13;2(6):e525. doi: 10.1371/journal.pone.0000525 (PMC1888727; doi:10.1371/journal.pone.0000525)
Supplement: Table S10 — Transforming Growth Factor-Beta Signaling. CN = Cochlea Neomycin timecourse. CL = Cochlea Laser timecourse. UN = Utricle Neomycin timecourse. UL = Utricle Laser timecourse. (0.03 MB DOC) [file pone.0000525.s011.doc]

Supplementary Table S10 Transforming Growth Factor-Beta Signaling

| **GeneID** | **Function** | **Reference** | **Diff Expr Timecourse** |
| --- | --- | --- | --- |
| DLX3 | Activated by BMP-2 through Smad1 and Smad4 | Park and Morasso, 2002 | CN |
| ELF3 | Activates TGF-beta RII | Chang et al., 2000 | UL, CN |
| ETV1 | Induces Smad7 expression  Activated by TAK1 via the p38 mitogen-activated protein kinase pathway | Dowdy et al., 2003 | CL |
| FOXH1 | Forms a complex with Smad2 and Smad4 which can activate Nodal | Zhou et al., 1998, Osada et al., 2000 | CL, CN |
| ID3 | Induced by BMP-2 signaling | Nakashima et al., 2001 | CL, CN |
| MADH2 | Phosphorylated by TGF-beta RI and RII  Transducer of TGF-β signaling | Macias-Silva et al., 1996 | UN, CL |
| MADH5 | Phosphorylated and activated by BMP-2 signaling | Nishimura et al., 1998 | CL |
| TCF8 | Binds p300 with Smads | Postigo et al., 2003 | UL, UN, CN |
| TITF1 | Interacts with Smad3 | Li et al., 2002 | UN, CL, CN |

Chang J, Lee C, Hahm KB, Yi Y, Choi SG, Kim SJ. Over-expression of ERT(ESX/ESE-1/ELF3), an ets-related transcription factor, induces endogenous TGF-beta type II receptor expression and restores the TGF-beta signaling pathway in Hs578t human breast cancer cells. Oncogene. 2000, 19:151-4.

Dowdy SC, Mariani A, Janknecht R. HER2/Neu- and TAK1-mediated up-regulation of the transforming growth factor beta inhibitor Smad7 via the ETS protein ER81. J Biol Chem. 2003, 278:44377-84.

Li C, Zhu NL, Tan RC, Ballard PL, Derynck R, Minoo P. Transforming growth factor-beta inhibits pulmonary surfactant protein B gene transcription through SMAD3 interactions with NKX2.1 and HNF-3 transcription factors. J Biol Chem. 2002, 277:38399-408.

Macias-Silva M, Abdollah S, Hoodless PA, Pirone R, Attisano L, Wrana JL. MADR2 is a substrate of the TGFbeta receptor and its phosphorylation is required for nuclear accumulation and signaling. Cell. 1996, 87:1215-24.

Nakashima K, Takizawa T, Ochiai W, Yanagisawa M, Hisatsune T, Nakafuku M, Miyazono K, Kishimoto T, Kageyama R, Taga T. BMP2-mediated alteration in the developmental pathway of fetal mouse brain cells from neurogenesis to astrocytogenesis. Proc Natl Acad Sci U S A. 2001, 98:5868-73.

Nishimura R, Kato Y, Chen D, Harris SE, Mundy GR, Yoneda T. Smad5 and DPC4 are key molecules in mediating BMP-2-induced osteoblastic differentiation of the pluripotent mesenchymal precursor cell line C2C12. J Biol Chem. 1998, 273:1872-9.

Osada SI, Saijoh Y, Frisch A, Yeo CY, Adachi H, Watanabe M, Whitman M, Hamada H, Wright CV. Activin/nodal responsiveness and asymmetric expression of a Xenopus nodal-related gene converge on a FAST-regulated module in intron 1. Development. 2000, 127:2503-14.

Park GT, Morasso MI. Bone morphogenetic protein-2 (BMP-2) transactivates Dlx3 through Smad1 and Smad4: alternative mode for Dlx3 induction in mouse keratinocytes. Nucleic Acids Res. 2002, 30:515-22.

Postigo AA, Depp JL, Taylor JJ, Kroll KL. Regulation of Smad signaling through a differential recruitment of coactivators and corepressors by ZEB proteins. EMBO J. 2003, 22:2453-62.

Zhou S, Zawel L, Lengauer C, Kinzler KW, Vogelstein B. Characterization of human FAST-1, a TGF beta and activin signal transducer. Mol Cell. 1998, 2:121-7.
