# Supplementary material for: Large Scale Gene Expression Profiles of Regenerating Inner Ear Sensory Epithelia
Source: PLoS One. 2007 Jun 13;2(6):e525. doi: 10.1371/journal.pone.0000525 (PMC1888727; doi:10.1371/journal.pone.0000525)
Supplement: Table S11 — The PAX-EYA-SIX pathway. CN = Cochlea Neomycin timecourse. CL = Cochlea Laser timecourse. UN = Utricle Neomycin timecourse. UL = Utricle Laser timecourse. (0.03 MB DOC) [file pone.0000525.s012.doc]

Supplementary table S11**.**

| **GeneID** | **Function** | **Reference** | **Diff Expr Timecourse** |
| --- | --- | --- | --- |
| BAPX1 | Middle ear pattern regulation, enhanced by Pax9 | Tucker et al., 2004 | CN |
| EYA1 | Dephosphorylates Six1 altering its function from repressor to activator | Li et al., 2003 | CN |
| HHEX | Induced by PAX8 | Puppin et al., 2004 | UN,CN |
| MSX2 | Represses Six3 | Lengler and Graw, 2001 | CN |
| PAX1 | Induces BAPX1; repressed by BMP4 and BMP2 during limb development | Rodrigo et al., 2003  Hofmann et al., 1998 | CN |
| PAX3 | Synergizes with SOX10 to activate MITF transcription  Mutation results in Waardenburg syndrome (sensorineural deafness due to lack of melanogenesis) | Bondurand et al., 2000  Watanabe et al., 1998 | CL |
| PAX4 | Possible role in beta cell regenerative capacity | Biason-Lauber et al., 2005 | CL, CN |
| PAX5 | Promotes B-cell specific genes  Functionally equivalent to PAX2 in the inner ear | Delogu et al., 2006  Bouchard et al., 2000 | CN |
| PAX7 | Role in renewal of muscle satellite cells (stem cells) | Olguin and Olwin, 2004 | CL, CN |
| PAX8 | Involved in otic specification | Hans et al., 2004 | CL, CN |
| PAX9 | Interacts with Msx1 during tooth development | Ogawa et al., 2005 | CL |
| PROX1 | Activates Six3 | Lengler and Graw, 2001 | CN |
| SIX1 | Cell cycle regulation  Interacts with EYA1-crucial for morphogenesis of cochlea | Ford et al., 1998  Coletta et al., 2004  Zheng et al., 2003 | CL, CN |
| SIX2 | Induces translocation of Eya proteins | Ohto et al., 1999 | CN |
| SIX3 | Cell proliferation and differentiation. Regulated by Pax6, Prox1 and Msx2 | Li et al., 2003  Lengler and Graw, 2001 | UN, CN |
| SIX4 | Expressed in all sensorineural placodes | Ozaki et al., 2001 | CN |
| SIX6 | With DACH corepressors, directly represses p27Kip1 | Li et al., 2002 | UN |
| TITF1 | Directly interacts with PAX8 | DiPalma et al., 2003 | UN, CL, CN |

Biason-Lauber A, Boehm B, Lang-Muritano M, Gauthier BR, Brun T, Wollheim CB, Schoenle EJ. Association of childhood type 1 diabetes mellitus with a variant of PAX4: possible link to beta cell regenerative capacity. Diabetologia. 2005, 48:900-5.

Bondurand N, Pingault V, Goerich DE, Lemort N, Sock E, Caignec CL, Wegner M, Goossens M. Interaction among SOX10, PAX3 and MITF, three genes altered in Waardenburg syndrome. Hum Mol Genet. 2000, 9:1907-17.

Bouchard M, Pfeffer P, Busslinger M. Functional equivalence of the transcription factors Pax2 and Pax5 in mouse development. Development. 2000, 127:3703-13.

Coletta RD, Christensen K, Reichenberger KJ, Lamb J, Micomonaco D, Huang L, Wolf DM, Muller-Tidow C, Golub TR, Kawakami K, Ford HL. The Six1 homeoprotein stimulates tumorigenesis by reactivation of cyclin A1. Proc Natl Acad Sci U S A. 2004, 101:6478-83.

Delogu A, Schebesta A, Sun Q, Aschenbrenner K, Perlot T, Busslinger M. Gene repression by Pax5 in B cells is essential for blood cell homeostasis and is reversed in plasma cells. Immunity. 2006, 24:269-81.

Di Palma T, Nitsch R, Mascia A, Nitsch L, Di Lauro R, Zannini M. The paired domain-containing factor Pax8 and the homeodomain-containing factor TTF-1 directly interact and synergistically activate transcription.
J Biol Chem. 2003, 278:3395-402.

Ford HL, Kabingu EN, Bump EA,Mutter GL, Pardee AB. Abrogation of the G_2_ cell cycle checkpoint associates with overexpression of HSIX1: A possible mechanism of breast carcinogenesis. Proc Natl Acad Sci. 1998, 96:12608-12613

Hans S, Liu D, Westerfield M. Pax8 and Pax2a function synergistically in otic specification, downstream of the Foxi1 and Dlx3b transcription factors. Development. 2004, 131:5091-102.

Hofmann C, Drossopoulou G, McMahon A, Balling R, Tickle C. Inhibitory action of BMPs on Pax1 expression and on shoulder girdle formation during limb development. Dev Dyn. 1998, 213:199-206.

Lengler J, Graw J. Regulation of the human SIX3 gene promoter.Biochem Biophys Res Commun. 2001, 287:372-6.

Li X, Perissi V, Liu F, Rose DW, Rosenfeld MG. Tissue-specific regulation of retinal and pituitary precursor cell proliferation. Science. 2002, 297:1180-3.

Li K, Oghi A, Zhang J, Krones A, Bush KT, Glass CK, Nigam SK, Aggarwal AK, Maas R, Rose DW, Rosenfield MG. Eya protein phosphatase activity regulates Six1-Dach-Eya transcriptional effects in mammalian organogenesis. Nature. 2003, 426:247-54.

Ogawa T, Kapadia H, Wang B, D'Souza RN. Studies on Pax9-Msx1 protein interactions. Arch Oral Biol. 2005, 50:141-5.

Ohto H, Kamada S, Tago K, Tominaga SI, Ozaki H, Sato S, Kawakami K. Cooperation of six and eya in activation of their target genes through nuclear translocation of Eya. Mol Cell Biol. 1999, 19:6815-24.

Olguin HC, Olwin BB. Pax-7 up-regulation inhibits myogenesis and cell cycle progression in satellite cells: a potential mechanism for self-renewal. Dev Biol. 2004, 275:375-88.

Ozaki H, Watanabe Y, Takahashi K, Kitamura K, Tanaka A, Urase K, Momoi T, Sudo K, Sakagami J, Asano M, Iwakura Y, Kawakami K. Six4, a putative myogenin gene regulator, is not essential for mouse embryonal development. Mol Cell Biol. 2001, 21:3343-50.

Puppin C, Presta I, D'Elia AV, Tell G, Arturi F, Russo D, Filetti S, Damante G. Functional interaction among thyroid-specific transcription factors: Pax8 regulates the activity of Hex promoter. Mol Cell Endocrinol. 2004, 214:117-25.

Rodrigo I, Hill RE, Balling R, Munsterberg A, Imai K. Pax1 and Pax9 activate Bapx1 to induce chondrogenic differentiation in the sclerotome. Development. 2003, 130:473-82.

Tucker A, Watson RP, Lettice LA, Yamada G, Hill RE. Bapx1 regulates patterning in the middle ear: altered regulatory role in the transition from the proximal jaw during vertebrate evolution. Development. 2004, 131:1235-1245

Watanabe A, Takeda K, Ploplis B, Tachibana M. Epistatic relationship between Waardenburg syndrome genes MITF and PAX3. Nat Genet. 1998, 18:283-6.

Zheng W, Huang L, Wei ZB, Silvius D, Tang B, Xu PX. The role of Six1 in mammalian auditory system development. Development. 2003, 130:3989-4000.
