# Supplementary material for: Large Scale Gene Expression Profiles of Regenerating Inner Ear Sensory Epithelia
Source: PLoS One. 2007 Jun 13;2(6):e525. doi: 10.1371/journal.pone.0000525 (PMC1888727; doi:10.1371/journal.pone.0000525)
Supplement: Table S12 — Notch signaling. CN = Cochlea Neomycin timecourse. CL = Cochlea Laser timecourse. UN = Utricle Neomycin timecourse. UL = Utricle Laser timecourse. (0.02 MB DOC) [file pone.0000525.s013.doc]

Supplementary Table S12

| **GeneID** | **Function** | **Reference** | **Diff Expr Timecourse** |
| --- | --- | --- | --- |
| FHL1 | Interacts with RBP-J and modulates Notch signaling. | Li and Zhang, 2003 | UL, UN, CN |
| HES7 | Expression controlled by Notch | Bessho et al., 2001 | UL, CL |
| HEY1 | Upregulated in response to Notch via RBP-Jkappa | Maier and Gessler, 2000  Fischer et al., 2004 | UN |
| HEY2 | Transducer of Notch signals | Fischer et al., 2004 | UL, CN |
| HEYL | Upregulated in response to Notch via RBP-Jkappa | Maier and Gessler, 2000 | CL |

Bessho Y, Miyoshi G, Sakata R, Kageyama R. Hes7: a bHLH-type repressor gene regulated by Notch and expressed in the presomitic mesoderm. Genes Cells. 2001, 6:175-85.

Fischer A, Schumacher N, Maier M, Sendtner M, Gessler M. The Notch target genes Hey1 and Hey2 are required for embryonic vascular development. Genes Dev. 2004, 18:901-11.

Li R, Zhang R. The interactions of LIM protein KyoT with polycomb proteins. Sheng Wu Hua Xue Yu Sheng Wu Wu Li Xue Bao (Shanghai). 2003, 35:113-6.

Maier MM, Gessler M. Comparative analysis of the human and mouse Hey1 promoter: Hey genes are new Notch target genes. Biochem Biophys Res Commun. 2000, 275:652-60.
