# Supplementary material for: Large Scale Gene Expression Profiles of Regenerating Inner Ear Sensory Epithelia
Source: PLoS One. 2007 Jun 13;2(6):e525. doi: 10.1371/journal.pone.0000525 (PMC1888727; doi:10.1371/journal.pone.0000525)
Supplement: Table S13 — WNT signaling. CN = Cochlea Neomycin timecourse. CL = Cochlea Laser timecourse. UN = Utricle Neomycin timecourse. UL = Utricle Laser timecourse. (0.03 MB DOC) [file pone.0000525.s014.doc]

Supplementary Table S13

| **GeneID** | **Function** | **Reference** | **Diff Expr Timecourse** |
| --- | --- | --- | --- |
| CBX4 | Enhances sumoylation of CtBP (repressor of TCF/LEF) | Kagey et al., 2003 | UN, CN |
| CTNNB1 | Downstream of WNT receptor. Activates TCF/LEF transcription factors. | Willert and Nusse 1998 | UN, CN |
| PPARG | Supresses WNT/β-catenin signaling | Moldes et al., 2003 | CN |
| PPARGC1 | PPARG coactivator | Feilchenfeldt et al., 2004 | UN |
| TCF3 | Activated by CTNNB1. Activates downstream targets of WNT signaling pathway | Merrill et al., 2004 | CN |
| TCF7L2 | Activated by CTNNB1. Activates downstream targets of WNT signaling pathway | Douglas et al., 2001 | CL |
| TCF8 | Transcriptional repressor of E-cadherin | Guaita et al., 2002 | UL,UN, CN |

Douglas KR, Brinkmeier ML, Kennell JA, Eswara P, Harrison TA, Patrianakos AI, Sprecher BS, Potok MA, Lyons RH Jr, MacDougald OA, Camper SA. Identification of members of the Wnt signaling pathway in the embryonic pituitary gland. Mamm Genome. 2001, 12:843-51.

Feilchenfeldt J, Brundler MA, Soravia C, Totsch M, Meier CA. Peroxisome proliferator-activated receptors (PPARs) and associated transcription factors in colon cancer: reduced expression of PPARgamma-coactivator 1 (PGC-1).Cancer Lett. 2004, 203:25-33.

Guaita S, Puig I, Franci C, Garrido M, Dominguez D, Batlle E, Sancho E, Dedhar S, De Herreros AG, Baulida J. Snail induction of epithelial to mesenchymal transition in tumor cells is accompanied by MUC1 repression and ZEB1 expression. J Biol Chem. 2002, 277:39209-16.

Kagey MH, Melhuish TA, Wotton D. The polycomb protein Pc2 is a SUMO E3. Cell. 2003, 113:127-137.

Merrill BJ, Pasolli HA, Polak L, Rendl M, Garcia-Garcia MJ, Anderson KV, Fuchs E. Tcf3: a transcriptional regulator of axis induction in the early embryo. Development. 2004, 131:263-74.

Moldes M, Zuo Y, Morrison RF, Silva D, Park BH, Liu J, Farmer SR. Peroxisome-proliferator-activated receptor gamma suppresses Wnt/beta-catenin signalling during adipogenesis. Biochem J. 2003, 376:607-13.

Willert K, Nusse R. β-catenin: a key mediator of Wnt signaling. Current Opinion in Genetics and Development. 1998, 8:96-102.
