# Supplementary material for: Large Scale Gene Expression Profiles of Regenerating Inner Ear Sensory Epithelia
Source: PLoS One. 2007 Jun 13;2(6):e525. doi: 10.1371/journal.pone.0000525 (PMC1888727; doi:10.1371/journal.pone.0000525)
Supplement: Table S14 — NFKappaB. CN = Cochlea Neomycin timecourse. CL = Cochlea Laser timecourse. UN = Utricle Neomycin timecourse. UL = Utricle Laser timecourse. (0.02 MB DOC) [file pone.0000525.s015.doc]

Supplementary Table S14

| **GeneID** | **Function** | **Reference** | **Diff Expr Timecourse** |
| --- | --- | --- | --- |
| HIF1A | Induced by NFκB | Jung et al., 2003 | UN, CN |
| LDOC1 | Inhibits NFκB | Nagasaki et al., 2003 | CN |
| NFKB2 (p100) | Induced by IκB kinase α phosphorylation | Xiao et al., 2004 | CN |

Jung Y-J, Isaacs JS, Lee S, Trepel J, Neckers L. Microtubule disruption utilizes an NFκB-dependent pathway to stabilize HIF-1α protein. J Biol Chem. 2003, 278:7445-52.

Nagasaki K, Schem C, von Kaisenberg C, Biallek M, Rosel F, Jonat W, Maass N. Leucine-zipper protein, LDOC1, inhibits NF-kappaB activation and sensitizes pancreatic cancer cells to apoptosis. Int J Cancer. 2003, 105:454-8.

Xiao G, Fong A, Sun S-C. Induction of p100 processing by NF-kappaB-inducing kinase involves docking IkappaB kinase alpha (IKKalpha) to p100 and IKKalpha-mediated phosphorylation. J Biol Chem. 2004, 279:30099-105.
