# Supplementary material for: Large Scale Gene Expression Profiles of Regenerating Inner Ear Sensory Epithelia
Source: PLoS One. 2007 Jun 13;2(6):e525. doi: 10.1371/journal.pone.0000525 (PMC1888727; doi:10.1371/journal.pone.0000525)
Supplement: Table S15 — Insulin/IGF signaling. CN = Cochlea Neomycin timecourse. CL = Cochlea Laser timecourse. UN = Utricle Neomycin timecourse. UL = Utricle Laser timecourse. (0.02 MB DOC) [file pone.0000525.s016.doc]

Supplementary Table S15

| **GeneID** | **Function** | **Reference** | **Diff Expr Timecourse** |
| --- | --- | --- | --- |
| EGR1 | Expression induced by insulin | Harada et al., 1995  Jhun et al., 1995 | CL, CN |
| ELK1 | Phosphorylated by MAPK , promotes growth and division | Sharrocks, 2002  Langlais et al., 2004 | CL |
| FOS | Expression induced by insulin | Harada et al., 1995  Jhun et al., 1995 | CL |
| HIF1A | Expression induced by IGF-1 | Fukuda et al., 2002 | UN, CN |
| MYBL2 | Activates IGFBP1 | Tanno et al., 2002 | UN, CN |
| SRF | Regulates many immediate-early genes including FOS and ELK1 | Murai and Treisman, 2002 | CN |

Fukuda R, Hirota K, Fan F, Jung YD, Ellis LM, Semenza GL. Insulin-like growth factor 1 induces hypoxia-inducible factor 1-mediated vascular endothelial growth factor expression, which is dependent on MAP kinase and phosphatidylinositol 3-kinase signaling in colon cancer cells. J Biol Chem. 2002, 277:38205-11.

Harada S, Smith RM, Smith JA, Shah N, Hu DQ, Jarett L. Insulin-induced egr-1 expression in Chinese hamster ovary cells is insulin receptor and insulin receptor substrate-1 phosphorylation-independent. Evidence of an alternative signal transduction pathway.J Biol Chem. 1995, 270:26632-8.

Jhun BH, Haruta T, Meinkoth JL, Leitner W, Draznin B, Saltiel AR, Pang L, Sasaoka T. Olefsky JM. Signal transduction pathways leading to insulin-induced early gene induction. Biochemistry. 1995, 34:7996-8004.

Langlais P, Dong LQ, Ramos FJ, Hu D, Li Y, Quon MJ, Liu F. Negative regulation of insulin-stimulated protein kinase signaling by Grb10. Mol Endocrin. 2004, 18:350-8.

Murai K, Treisman R. Interaction of serum response factor (SRF) with the Elk-1 B box inhibits RhoA-actin signaling to SRF and potentiates transcriptional activation by Elk-1. Mol Cell Biol. 2002, 22:7083-92.

Sharrocks AD. Complexities in ETS-domain transcription factor function and regulation: Lessons from the TCF (Ternary Complex Factor) subfamily. Biol Society Transaction. 2002, 30:1-9.

Tanno B, Negroni A, Vitali R, Pirozzoli MC, Cesi V, Mancini C, Calabretta B, Raschella G. Expression of insulin-like growth factor-binding protein 5 in neuroblastoma cells is regulated at the transcription level by c-Myb and B-Myb via direst and indirect mechanisms. J Biol Chem. 2002, 227:23172-80.
