# Supplementary material for: Large Scale Gene Expression Profiles of Regenerating Inner Ear Sensory Epithelia
Source: PLoS One. 2007 Jun 13;2(6):e525. doi: 10.1371/journal.pone.0000525 (PMC1888727; doi:10.1371/journal.pone.0000525)
Supplement: Table S16 — AP-1 Signaling. CN = Cochlea Neomycin timecourse. CL = Cochlea Laser timecourse. UN = Utricle Neomycin timecourse. UL = Utricle Laser timecourse. (0.03 MB DOC) [file pone.0000525.s017.doc]

Supplementary Table S16

| **GeneID** | **Function** | **Reference** | **Diff Expr Timecourse** |
| --- | --- | --- | --- |
| ATF2 | Histone acetyltransferase that forms a homodimer or heterodimer with c-Jun to stimulate transcription of cAMP response elements (CREs) | Kawasaki et al., 2000 | UN, CN |
| ATF7 | Homodimerizes to bind CRE elements | Peters et al., 2001 | CN |
| CEBPG | Downstream of JNK and JUND | Hawkins et al., unpublished | UL, UN |
| CROC4 | Involved in c-fos signaling | Jeffrey et al., 2000 | UN, CL |
| DRPLA | Phosphorylated by JNK | Okamura-Oho et al., 2003 | CN |
| ELK4 | Phosporylated by MAP kinases; binds the c-fos enhancer in combination with serum response factor (SRF) | Wang and Prywes, 2000 | CL |
| FOS | Heterodimerizes with JUN proteins to promote cell cycle progression by inducing cyclin D1 | Shaulian and Karin, 2002 (Review) | CL |
| JUN | Forms homodimers or heterodimers with Fos proteins; generally promotes cell cycle progression activating cyclin D1 and repressing p21^Cip1^ | Shaulian and Karin, 2002 (Review) | UL |
| JUND | Forms homodimers or heterodimers with Fos proteins; generally inhibits cell cycle progression repressing cyclin D1 and inducing p16^INK4a^ | Shaulian and Karin, 2002 (Review) | UL, CL, CN |
| MAPK8IP1 | Scaffold for MKP7 to reduce the activity of JNK, decreasing phosphorylation of c-Jun | Willoughby et al., 2003 | UN, CL, CN |

Jeffrey PL, Capes-Davis A, Dunn JM, Tolhurst O, Seeto G, Hannan AJ, Lin SL. CROC-4: a novel brain specific transcriptional activator of c-fos expressed from proliferation through to maturation of multiple neuronal cell types. Mol Cell Neurosci. 2000, 16:185-96.

Kawasaki H, Schiltz L, Chiu R, Itakura K, Taira K, Nakatani Y, Yokoyama KK. ATF-2 has intrinsic histone acetyltransferase activity which is modulated by phosphorylation. Nature. 2000, 405:195-200.

Okamura-Oho Y, Miyashita T, Nagao K, Shima S, Ogata Y, Katada T, Nishina H, Yamada M. Dentatorubral-pallidoluysian atrophy protein is phosphorylated by c-Jun NH2-terminal kinase. Hum Mol Genet. 2003, 12:1535-42.

Peters CS, Liang X, Li S, Kannan S, Peng Y, Taub R, Diamond RH. ATF-7, a novel bZIP protein, interacts with the PRL-1 protein-tyrosine phosphatase. J Biol Chem. 2001, 276:13718-26.

Shaulian E, Karin M. AP-1 as a regulator of cell life and death. Nat Cell Biol. 2002, 4:E131-6.

Wang Y, Prywes R. Activation of the c-fos enhancer by the erk MAP kinase pathway through two sequence elements: the c-fos AP-1 and p62TCF sites. Oncogene. 2000, 19:1379-85.

Willoughby EA, Perkins GR, Collins MK, Whitmarsh AJ. The JNK-interacting protein-1 scaffold protein targets MAPK phosphatase-7 to dephosphorylate JNK. J Biol Chem. 2003, 278:10731-6.
