# Supplementary material for: Large Scale Gene Expression Profiles of Regenerating Inner Ear Sensory Epithelia
Source: PLoS One. 2007 Jun 13;2(6):e525. doi: 10.1371/journal.pone.0000525 (PMC1888727; doi:10.1371/journal.pone.0000525)
Supplement: Table S17 — Cell Cycle/Apoptosis. CN = Cochlea Neomycin timecourse. CL = Cochlea Laser timecourse. UN = Utricle Neomycin timecourse. UL = Utricle Laser timecourse. (0.03 MB DOC) [file pone.0000525.s018.doc]

Supplementary Table S17

| **GeneID** | **Function** | **Reference** | **Diff Expr Timecourse** |
| --- | --- | --- | --- |
| BCL11B | Negative regulation of cell proliferation | Wakabayashi et al., 2003 | UN, CL, CN |
| BRD7 | Inhibits G1-S progression through ras/MEK/ERK & Rb/E2F pathways. | Zhou et al., 2004 | CN |
| CDK7 | Member of TFIIH complex.  Cell cycle progression. | Serizawa et al., 1995  Akoulitchev and Reinberg, 1998 | UN |
| CUTL1 | Plays a role in cell cycle progression via p27  Functions as the DNA binding subunit of the HiNT-D complex | Ledford et al., 2002  Gupta et al., 2003 | UN, CL, CN |
| DEAF1 | Inhibitor of cell cycle entry. | Manne et al., 2001 | UN, CN |
| HOX11 | Disrupts a G2/M cell cycle checkpoint by interaction with PP2A and PP1 phosphatases | Kawabe et al., 1997 | CL |
| MYBL2 | Phosphorylated by cyclinA/CDK2 during S-phase. | Saville and Watson, 1998 | UN, CN |
| MYCBP | Interacts with c-MYC. | Taira et al., 1998 | UL, UN, CN |
| PURA | Development coupled cell proliferation. | Khalili et al., 2003 | UN, CN |
| RGC32 | Promotes entry into S-phase through increase in p34CDC2 kinase activity | Badea et al., 2002 | UL |
| SMARCA2 | Negative regulation of cell proliferation | Reyes et al., 1998 | UL |
| TRIP15 | Member of COP9 complex regulating p27 | Yang et al., 2002 | UL, UN, CL, CN |
| FOXO3A | Targets TRAIL(TNF apoptosis inducing ligand).  Downstream target of Akt promoting apoptosis via FLIP downregulation. | Modur et al., 2002  Skurk et al., 2004 | CN |
| HIF1A | Hypoxic cell death | Lee et al., 2004 | UN, CN |
| HOXA5 | Can induce apoptosis through Caspase 2 & 8 | Chen et al., 2004 | UL |
| ID3 | Induces activation of caspase-2 and apoptosis in lymphocytes  Induces activation of caspase-3 and -9-dependent apoptosis in keratinocytes | Kee 2005  Simbulan-Rosenthal et al., 2006 | CL, CN |
| RORC | Negative regulator of apoptosis. | Kurebayashi et al., 2000 | CN |

Akoulitchev S, Reinberg D. The molecular mechanism of mitotic inhibition of TFIIH is mediated by phosphorylation of CDK7. Genes Dev. 1998, 12:3541-50.

Badea T, Niculescu F, Soane L, Fosbrink M, Sorana H, Rus V, Shin ML, Rus H. RGC-32 increases p34CDC2 kinase activity and entry of aortic smooth muscle cells into S-phase. J Biol Chem. 2002, 277:502-8.

Chen H, Chung S, Sukumar S. HOXA5-induced apoptosis in breast cancer cells is mediated by caspases 2 and 8. Mol Cell Biol. 2004, 24:924-35.

Gupta S, Luong MX, Bleuming SA, Miele A, Luong M, Young D, Knudsen ES, Van Wijnen AJ, Stein JL, Stein GS.Tumor suppressor pRB functions as a co-repressor of the CCAAT displacement protein (CDP/cut) to regulate cell cycle controlled histone H4 transcription.J Cell Physiol. 2003, 196:541-56.

Kawabe T, Muslin AJ, Korsmeyer SJ. HOX11 interacts with protein phosphatases PP2A and PP1 and disrupts a G2/M cell-cycle checkpoint. Nature. 1997, 385:454-8.

Kee BL. Id3 induces growth arrest and caspase-2-dependent apoptosis in B lymphocyte progenitors.
J Immunol. 2005, 175:4518-27.

Khalili K, Del Valle L, Muralidharan V, Gault WJ, Darbinian N, Otte J, Meier E, Johnson EM, Daniel DC, Kinoshita Y, Amini S, Gordon J. Puralpha is essential for postnatal brain development and developmentally coupled cellular proliferation as revealed by genetic inactivation in the mouse. Mol Cell Biol. 2003, 23:6857-75.

Kurebayashi S, Ueda E, Sakaue M, Patel DD, Medvedev A, Zhang F, Jetten AM. Retinoid-related orphan receptor gamma (RORgamma) is essential for lymphoid organogenesis and controls apoptosis during thymopoiesis. Proc Natl Acad Sci U S A. 2000, 97:10132-7.

Ledford AW, Brantley JG, Kemeny G, Foreman TL, Quaggin SE, Igarashi P, Oberhaus SM, Rodova M, Calvet JP, Vanden Heuvel GB. Deregulated expression of the homeobox gene Cux-1 in transgenic mice results in downregulation of p27(kip1) expression during nephrogenesis, glomerular abnormalities, and multiorgan hyperplasia. Dev Biol. 2002, 245:157-71.

Lee MJ, Kim JY, Suk K, Park JH. Identification of the hypoxia-inducible factor 1 alpha-responsive HGTD-P gene as a mediator in the mitochondrial apoptotic pathway. Mol Cell Biol. 2004, 24:3918-27.

Manne U, Gary BD, Oelschlager DK, Weiss HL, Frost AR, Grizzle WE. Altered subcellular localization of suppressin, a novel inhibitor of cell-cycle entry, is an independent prognostic factor in colorectal adenocarcinomas. Clin Cancer Res. 2001, 7:3495-503.

Modur V, Nagarajan R, Evers BM, Milbrandt J. FOXO proteins regulate tumor necrosis factor-related apoptosis inducing ligand expression. Implications for PTEN mutation in prostate cancer. J Biol Chem. 2002, 277:47928-37.

Reyes JC, Barra J, Muchardt C, Camus A, Babinet C, Yaniv M. Altered control of cellular proliferation in the absence of mammalian brahma (SNF2alpha). EMBO J. 1998, 17:6979-91.

Saville MK, Watson RJ. The cell-cycle regulated transcription factor B-Myb is phosphorylated by cyclin A/Cdk2 at sites that enhance its transactivation properties. Oncogene. 1998, 17:2679-89.

Serizawa H, Makela TP, Conaway JW, Conaway RC, Weinberg RA, Young RA. Association of Cdk-activating kinase subunits with transcription factor TFIIH. Nature. 1995, 374:280-2.

Simbulan-Rosenthal CM, Daher A, Trabosh V, Chen WC, Gerstel D, Soeda E, Rosenthal DS. Id3 induces a caspase-3- and -9-dependent apoptosis and mediates UVB sensitization of HPV16 E6/7 immortalized human keratinocytes.
Oncogene. 2006, 25: 3649-60.

Skurk C, Maatz H, Kim HS, Yang J, Abid MR, Aird WC, Walsh K. The Akt-regulated forkhead transcription factor FOXO3a controls endothelial cell viability through modulation of the caspase-8 inhibitor FLIP. J Biol Chem. 2004, 279:1513-25.

Taira T, Maeda J, Onishi T, Kitaura H, Yoshida S, Kato H, Ikeda M, Tamai K, Iguchi-Ariga SM, Ariga H. AMY-1, a novel C-MYC binding protein that stimulates transcription activity of C-MYC. Genes Cells. 1998, 3:549-65.

Wakabayashi Y, Inoue J, Takahashi Y, Matsuki A, Kosugi-Okano H, Shinbo T, Mishima Y, Niwa O, Kominami R. Homozygous deletions and point mutations of the Rit1/Bcl11b gene in gamma-ray induced mouse thymic lymphomas.

Biochem Biophys Res Commun. 2003, 301:598-603.

Yang X, Menon S, Lykke-Andersen K, Tsuge T, Di Xiao, Wang X, Rodriguez-Suarez RJ, Zhang H, Wei N. The COP9 signalosome inhibits p27(kip1) degradation and impedes G1-S phase progression via deneddylation of SCF Cul1. Curr Biol. 2002, 12:667-72.

Zhou J, Ma J, Zhang BC, Li XL, Shen SR, Zhu SG, Xiong W, Liu HY, Huang H, Zhou M, Li GY. BRD7, a novel bromodomain gene, inhibits G1-S progression by transcriptionally regulating some important molecules involved in ras/MEK/ERK and Rb/E2F pathways. J Cell Physiol. 2004, 200:89-98.
