# Supplementary material for: Large Scale Gene Expression Profiles of Regenerating Inner Ear Sensory Epithelia
Source: PLoS One. 2007 Jun 13;2(6):e525. doi: 10.1371/journal.pone.0000525 (PMC1888727; doi:10.1371/journal.pone.0000525)
Supplement: Table S18 — Utricle Self Organizing Map Centroid Groups shown in Figure 3A. (0.03 MB DOC) [file pone.0000525.s019.doc]

Supplementary Table S18:

**Centroid 0**

NR1I3 PURA SIX3 VENTX2

**Centroid 1**

CBX4 CREG DNAJ E2F2

HSF1 KIAA1041 MTF1 MYT1

PBX4 PRDM13 RBL2 RNF10

RNF15 SMARCB1 SREBF1 STAT3

TAF1B TAF2I TAF2K TCF8

**Centroid 2**

AF5Q31 ATF2 CSRP2 DKFZP434B0335

DLX6 FOG2 NEUROD6 RFX3

SOX general TAF-172 ZNF274 ZXDA

**Centroid 3**

BRD1 C21orf18 CEBPG FHL1

FOXP1 HES7 HNF3A HOXA13

IRF2 JUND MYT2 PRDM16

TAF2H TCF21

**Centroid 4**

CRX ELF3 HLX1 JUN

LOC51637 M96 MTA1L1

**Centroid 5**

CDK7 CROC4 HOXA5 ISGF3G

MAFF TAF1C ZNF135 ZNF214

**Centroid 6**

ASH2L BCL11B ETV5 GIOT-2

HSF2BP KIAA0669 MAPK8IP1 MLLT6

PMF1 TGFB1I1 TITF1 TNRC9

TNRC12 ZNF76

**Centroid 7**

CUTL1 E2F5 GTF2F1 HEY1

HIF1A ILF1 MYBL2 NFE2L1

PPARBP PSMC5 SOX14 TRIP15

ZNF288 ZNF7

**Centroid 8**

CREBL1 CSDA EBF EOMES

ERCC6 EZH2 GTF2H1 GTF2H3

HHEX HIVEP1 HIRA HOXD12

POU4F2 POU4F3 ZF5128 ZNF286

**Centroid 9**

CBX3 CITED1 DUX2 FLJ10251

FLJ10891 FLJ12517 FLJ12827 FLJ13590

FLJ20595 GTF3C4 H-L(3)MBT KIAA0130

LHX4 MEIS2 MYCL2 NRF

PDEF PILB PLAG1 PROP1

SIX6 ZNF10 ZNF21 ZNF281

**Centroid 10**

FLJ13222 LOC92283 PHAP1 RFP

RGC32 SAP30 SMARCA2 ZNF20

**Centroid 11**

CRSP6 MORF NFIB NR1H3

**Centroid 12**

CTNNB1 PPARGC1 RNF14 HNF3B

**Centroid 13**

BLZF1 GTF2E1 HOXB7 HSAJ2425

HSPC018 KIAA0173 KIAA0395 KIAA1528

LOC51058 LOC51131 LZLP MADH2

MYCBP POU4F1 RERE SSX4

ZNF174

**Centroid 14**

BCL11A FLJ10697 GTF2A1 HOXD8

LOC57209 TBX15 TBX5 WHSC1

ZFY ZNF79 ZNF90 ZNF93

**Centroid 15**

DEAF1 FLJ11186 HEY2 KIAA0040

KIAA0414 LOC51036 TFE3 ZHX1

ZID ZNF187 ZNF212 ZNF75A
