# Supplementary material for: Large Scale Gene Expression Profiles of Regenerating Inner Ear Sensory Epithelia
Source: PLoS One. 2007 Jun 13;2(6):e525. doi: 10.1371/journal.pone.0000525 (PMC1888727; doi:10.1371/journal.pone.0000525)
Supplement: Table S19 — Cochlea Self Organizing Map Centroid Groups shown in Figure 3B. (0.04 MB DOC) [file pone.0000525.s020.doc]

Supplementary Table S19.

**Centroid 0**

ARIX BACH1 CBX4 CROC4

DLX6 DNAJ DRIL1 EMX2

FHL1 FKHL18 IGHMBP2 ILF1

IRF1 IRF2 KLF15 KRML

LAF4 LIM MORF MYT2

NAB2 NFX1 NR0B1 NR1H3

NR5A1 ONECUT2 PAX5 PLAGL1

PMX1 PRDM15 PROX1 PURA

RNF13 RNF14 RORC SDCCAG33

SIX1 SIX2 SIX3 SIX4

SPIB SRF STAT6 TAF2H

TAF2N TCF3 TFAP4

**Centroid 1**

BAZ2B BRPF3 CHD4 CITED1

CL469780 E2F6 EBF ELK1

EN2 EZH1 EZH2 FLJ12827

FLJ13659 FLJ20321 FLJ22301 FOXF1

GLI3 GTF2E1 HCNGP HEYL

HOXD1 IRX4 MEOX1 MEOX2

MYB NCOR2 NFE2L1 P1P373C6

POU3F4 POU6F1 PREB RBL1

SNAPC5 TAF2C1 TBX10 TBX18

TCFL1 TEAD3 ZNF239 NFATC2

**Centroid 2**

AF093680 ATBF1 BCL6 COPEB

DKFZP434P1750 EP300 ETV1 FHX

FLJ13222 GFI1 GLIS2 GTF2B

GTF2H4 HBOA HDAC4 HES7

HNF3B HOXA2 ILF2 KIAA0293

KIAA0441 KIAA0943 LDB1 LOC51058

MAFF NFATC3 NKX2B NR2C2

NR2F6 PAX4 PAX8 PAX9

POU1F1 RBPSUHL RELA RORB

RXRB SLUG SMARCB1 SPI1

TCEAL1 TCF7L2 TFAP2A TFAP2B

TRIP15

**Centroid 3**

BRF2 CHD3 CLOCK CREB1

EGR1 FOXC2 GTF2F1 HES2

HOXC11 HSPC189 KIAA0014 LDOC1

MGC2508 NEUROG1 NFIL3 NHLH2

PRDM11 RNF4 ZNF76

**Centroid 4**

AHR ATF2 ATF3 ATF5

ATF6 ATF7 BARX2 CBX1

CREBBP CREM CSDA CTCF

DLX3 E2F3 E2F4 EGR3

ELF2 ELK3 ERCC6 FLI1

FOXF2 GABPA GBX1 GCN5L1

GCN5L2 GFI1B GTF3C1 GTF3C2

GRF3C4 HAND2 HHEX HIF1A

HIRA HNF3A HNF3G HOXB1

HOXB13 HOXB7 HOXC6 ICSBP1

IRF5 KLHL4 MSX2 NFE2L2

NFKBIL1 NR2C1 NRIP1 PER3

POU4F3 POU5F1 RBL2 RFXANK

RNF24 SHOX SNAPC3 SREBF2

SUPT4H1 SURB7 TAF2C2 TBP

TBX6 TEF ZNF205

**Centroid 5**

ARNT DLX5 DSIPI EOMES

ERCC3 ESRRG FOXC1 FOXO3A

GABPB1 GATA6 HKR3 HOXA6

HOXC13 HOXC5 HOXC8 HOXD12

ICBP90 IRLB LMO6 M96

MEIS3 P38IP PAX7 POU2F1

PPARG PRDM2 RBBP9 RELB

SETDB1

**Centroid 6**

CBX6 CITED2 DFKZP434E026 DKFZP547H236

FLJ12517 GTF2H2 HEY2 HOXA4

HOXA7 HSAJ2425 HSPC018 ISGF3G

LHX6 LOC56270 LOC57167 LOC57209

MADH2 MDS1 NFIB NFKB2

NR0B2 PER1 PER2 PTTG1IP

SAFB SMARCC1 SNAPC4 SSX1

SSX2 TBX21 TBX22 TCFL5

TIMELESS ZNF211 ZNF268 ZNF75A

**Centroid 7**

ARNTL CDX4 CRSP6 DR1

ELK4 FLJ10759 FOS FOXD1

GCMB HMG20B HOXA10 HOXD4

HSF4 KIAA1528 LDB2 LOC51045

LOC51652 MAD MAD4 MHC2TA

NFIC NPAS1 NR5A2 PAX3

RARG SHOX2 SIM1 TAF2G

THRA TIEG TNRC6 TRIM22

UTF1 WHN WHSC1 XBP1

ZIC1 ZNF11B ZNF142 ZNF230

ZNF294

**Centroid 8**

BTEB1 DRPLA FHL2 FOXM1

GTF21 HOXA13 HOXA9 ID3

IRX7 KLF5 MYBL2 NCOA3

NEUROG2 POU2AF1 PRDM12 RARA

SATB1 SIM2 SUPT3H TADA3L

TITF1 TNRC3 ZNF80 HRIHFB2436

**Centroid 9**

CDK8 CSEN CUTL1 DKFZP434B0335

GATA3 GLI GTF3A HLX1

HOX11 HOXB8 IRF6 LMO4

MAF MAX MEF2A PBX2

PPARD SETBP1 SOX5 ZNF9

**Centroid 10**

ARC BHLHB2 BHLHB3 BLZF1

CART1 CDX2 CEZANNE EPAS1

ESR1 FLJ10697 FLJ11191 FLJ21603

GAS41 HKR2 HOXB5 HOXD8

HSA275986 HSU90653 KIAA0535 KIAA0998

LOC56930 MAFG MED6 MYF6

PBX1 SCML2 TAF1C TEAD4

VAX2 ZNF143 ZNF202 ZNF25

CBX8

**Centroid 11**

CBFA2T3 CEBPB DLX1 EPLIN

EYA1 FLJ20729 FUBP1 GTF2A1

HMX1 HOXB9 HOXC9 LMX1B

MADH9 MTA1L1 NR2F1 NR2F2

PBX3 PKNOX1 PROP1 RFP2

SLB TAF2D TBR1 TBX3

TCF8 TMF1 TRIP6 TZFP

YAF2 ZFHX1B ZFY ZNF136

ZNF137 ZNF154 ZNF175 ZNF180

ZNF200 ZNF208 ZNF214 ZNF215

ZNF217 ZNF234 ZNF262 ZNF272

ZNF282 ZNF289 ZNF295 ZNF3

ZNF304 ZNF33A ZNF8

**Centroid 12**

AF5Q31 ATF4 BCL11B BRD1

BTF3L1 C21orf18 CIAO1 DEAF1

FLJ10142 FLJ20595 FOG2 FOXH1

FOXP1 GBX2 GIOT-2 GTF2E2

HOXA3 HRIHFB2122 JUND KIAA0130

KIAA0173 KIAA1041 LMO1 MEF2B

MSC MTA1 MYF5 NEUROD6

NR1I3 NR4A1 PAF65A PAX1

POU4F2 PRDM13 SOX2 TAF-172

TNRC5 TRIM15 ZNF10 ZNF174

ZNF38

**Centroid 13**

CERD4 CXorf6 FLJ22332 HCF2

HMG2 HOXB3 HOXD11 HSF2BP

KIAA0071 KIAA0395 KIAA1190 KIAA1388

LOC58500 LOC91120 MADH5 MAPK8IP1

NR2E3 OAZ SOX general TAL2

TBX1 TFCP2 VENTX2 ZID

ZIM2 ZNF36 ZNF73 ZNF93

ZNF-kaiso

**Centroid 14**

AIB3 BCL11A BRD7 COPS5

CREG CRSP8 CTNNB1 ELF3

EN1 ETS1 FLJ23309 H-L(3)MBT

HLF HR HSGT1 HSPX153

ILF3 KIAA0026 KIAA1442 LMO2

LOC51043 MGC16733 MLLT2 MYCBP

MYOD1 MYT1L NCOR1 NMI

PC4 PCAR POU2F2 POU4F1

PRDM9 RNF10 RNF22 RORA

RPF-1 RRN3 RUNX2 SRA1

SSX3 TBPL1 TBX15 TBX5

TCF12 TNRC12 TNRC4 TRAP150

TRIP11 ZF5128 ZFP103 ZFX

ZNF144 ZNF146 ZNF213 ZNF221

ZNF237 ZNF24 ZNF254 ZNF256

ZNF263 ZNF265 ZNF271 ZNF277

ZNF287 ZNF297 ZNF361 ZNF7

ZNF79 ZNF84 ZNF90 ZNFN1A3

**Centroid 15**

BAPX1 EGR2 FLJ11186 HOXC10

NFATC1 NR4A3 NSEP1 POU3F2

YY1 ZFP106 ZFP161 ZNF123

ZNF135 ZNF140 ZNF148 ZNF177

ZNF184 ZNF192 ZNF219 ZNF225

ZNF226 ZNF258 ZNF6
