# Supplementary material for: Large Scale Gene Expression Profiles of Regenerating Inner Ear Sensory Epithelia
Source: PLoS One. 2007 Jun 13;2(6):e525. doi: 10.1371/journal.pone.0000525 (PMC1888727; doi:10.1371/journal.pone.0000525)
Supplement: Table S20 — Utricle Detectably Expressed Genes (0.23 MB DOC) [file pone.0000525.s021.doc]

Supplementary Table S20.

1. Utricle Neomycin Venn Diagram Gene List

| O HR EXPRESSION - 87 GENES | | | | | |
| --- | --- | --- | --- | --- | --- |
| AIB3 | GCN5L2 | MYF6 | STAT6 | ZNF145 | |
| ATF3 | GLI3 | NFKBIE | TAF2A | ZNF151 | |
| CBX6 | HIVEP1 | NKX2B | TAF2E | ZNF162 | |
| CEZANNE | HMX1 | NR2F2 | TAF2F | ZNF193 | |
| CHD4 | HOXC9 | NR4A2 | TAF2J | ZNF197 | |
| CNOT8 | HYPH | POU1F1 | TAF2K | ZNF202 | |
| CSRP3 | IRF4 | PPARA | TBX19 | ZNF262 | |
| DACH | ISL1 | RBPSUHL | TBX22 | ZNF274 | |
| DMRT1 | KIAA0130 | RERE | TCF12 | ZNF278 | |
| DR1 | KIAA0426 | RFX4 | TEAD2 | ZNF32 | |
| DRAP1 | LEF1 | RFXANK | TEF | ZNF38 | |
| DSIPI | LOC56270 | RUNX1 | TGIF | ZNF41 | |
| EYA4 | LZTR1 | RXRA | TIEG | ZNF8 | |
| FLJ13590 | MAFF | SAFB | TIMELESS | ZNF81 | |
| FLJ20557 | MDS032 | SALF | TP53 | ZNF84 | |
| FLJ20729 | MLLT1 | SNAPC1 | TRIP4 |  | |
| FLJ22332 | MNDA | SOX13 | WHN |  | |
| FOXC1 | MYCL1 | STAT5A | ZNF134 |  | |
|  |  |  |  |  | |
| 24 HR EXPRESSION - 55 GENES | | | | | |
| AF093680 | DLX5 | GLI | MEF2A | SIX3 | |
| AHR | DLX6 | GTF2E1 | MEIS1 | SLUG | |
| ALY | E2F4 | HDAC1 | MID1 | SSRP1 | |
| ATF5 | E2F6 | HNF4G | MYT1 | TBX20 | |
| BHLHB2 | EGR2 | HOX11L | NFKB1 | TRIM28 | |
| BMI1 | EMX2 | HRY | NHLH2 | ZFP26 | |
| CL469780 | EP300 | IRF5 | PPARD | ZNF208 | |
| COPEB | FOSL2 | KIAA0222 | RB1 | ZNF220 | |
| CREM | FOXF2 | KIAA0293 | RELA | ZNF232 | |
| CSEN | GATA3 | LDB1 | RORB | ZNF35 | |
| DLX2 | GFI1 | MADH9 | RXRG | ZNF7 | |
|  |  |  |  |  | |
| 48 HR EXPRESSION - 101 GENES | | | | | |
| ATOH1 | FLJ20321 | KIAA1388 | PBX3 | ZIC1 | |
| BAZ2B | FOG2 | LOC51637 | PEGASUS | ZNF125 | |
| BC002881 | FOXI1 | LOC55885 | PLRG1 | ZNF132 | |
| BRF2 | FOXO1A | M96 | PMF1 | ZNF161 | |
| BRPF1 | GATA2 | MADH3 | POU5F1 | ZNF173 | |
| C5orf7 | GCN5L1 | MAZ | PRDM8 | ZNF175 | |
| CDX2 | GSH2 | MDS1 | RFX2 | ZNF185 | |
| CRIP1 | HDAC4 | MED6 | RNF14 | ZNF221 | |
| CTNNB1 | HKR3 | MILD1 | SAP18 | ZNF258 | |
| DBP | HLF | MLLT7 | SDCCAG33 | ZNF259 | |
| DKFZp547H236 | HOXA3 | MYOG | SMARCA3 | ZNF26 | |
| DLX4 | HOXD11 | NAB1 | SNW1 | ZNF261 | |
| DRPLA | HOXD9 | NFIX | TAF2B | ZNF264 | |
| DUX4 | HR | NFRKB | TAL2 | ZNF265 | |
| ELK4 | HRIHFB2436 | NMI | TCF3 | ZNF268 | |
| ESR2 | HSPC189 | NR1D1 | TFE3 | ZNF286 | |
| ETV2 | ICBP90 | NR2E3 | THRA | ZNF83 | |
| EYA2 | ID4 | NR3C2 | TITF1 |  | |
| EZH2 | IRF7 | NR5A2 | TRIP11 |  | |
| FLJ125 | IRX5 | ONECUT1 | TRPS1 |  | |
| FLJ12827 | KIAA0952 | OVOL1 | ZFP289 |  | |
|  |  |  |  |  | |
| O HR - 24 HR CO-EXPRESSION - 87 GENES | | | | | |
| ALX3 | ELK1 | HSF2 | NCOR2 | SOX10 | |
| ARC | ERG | ICSBP1 | NEUROD1 | SOX5 | |
| ARNT | ETV6 | ID2 | NFATC2 | SRY | |
| ATF1 | FKHL18 | IGHMBP2 | NFATC4 | SURB7 | |
| ATF7 | FLJ11186 | ILF1 | NPAS1 | TBPL1 | |
| BARX1 | FLJ12457 | JUN | NPAS2 | TBX10 | |
| BATF | FLJ12525 | KLF12 | ONECUT2 | ZFP93 | |
| BAZ2A | GATA1 | KLF5 | OTX2 | ZNF142 | |
| CART1 | GTF2F2 | KRML | PAX5 | ZNF146 | |
| CBFA2T1 | GTF2H3 | LMO4 | PAX8 | ZNF16 | |
| CEBPE | HIRA | LOC57862 | PCAR | ZNF234 | |
| CHD2 | HOXA10 | MAD | PGR | ZNF256 | |
| CREBBP | HOXA2 | MAF | POU2F1 | ZNF45 | |
| CROC4 | HOXA7 | MGC12942 | PRDM1 | ZNF46 | |
| CRX | HOXC8 | MNAT1 | PRDM11 | ZNF80 | |
| DDIT3 | HOXD10 | MSX2 | R32184_3 |  | |
| EGR1 | HOXD4 | MYT1L | RFP2 |  | |
| ELF1 | HRIHFB2122 | NCOA2 | SHOX |  | |
|  |  |  |  |  | |
| O HR - 48 HR CO-EXPRESSION - 245 GENES | | | | | |
| ARIX | GTF2F1 | LOC92283 | PRDM9 | TIEG2 | |
| ARNT2 | GTF2H1 | LW-1 | RARA | TMF1 | |
| ATF4 | GTF3C5 | LYL1 | RARG | TNRC3 | |
| AWP1 | HCF2 | LZTS1 | RBBP9 | TNRC6 | |
| BANP | HDAC2 | M6A | RBL2 | TRIP15 | |
| BAPX1 | HEY2 | MADH4 | RELB | UTF1 | |
| BRD7 | HEYL | MADH5 | REST | VAX2 | |
| C1orf2 | HKR2 | MADH7 | RGC32 | VSX1 | |
| CBFA2T3 | HNF3B | MEF2B | RLF | ZFHX1B | |
| CBX3 | HNF3G | MEF2C | RNF13 | ZFP | |
| CLOCK | HOXA13 | MEF2D | RNF2 | ZFP91 | |
| CNOT3 | HOXA6 | MGC16733 | RNF3 | ZFP95 | |
| CRSP7 | HOXB13 | MGC2508 | RNF8 | ZIC4 | |
| DFKZP434E026 | HOXB3 | MHC2TA | RORA | ZNF123 | |
| DKFZP434B195 | HOXD1 | MITF | RREB1 | ZNF131 | |
| DKFZP434N043 | HOXD13 | MLL | SALL3 | ZNF135 | |
| DKFZP564F1422 | HOXD3 | MLLT10 | SBB103 | ZNF137 | |
| DUX2 | HSA275986 | MTA1 | SCML2 | ZNF14 | |
| E4F1 | HSAJ2425 | MYBL1 | SETDB1 | ZNF141 | |
| EHF | HSF2BP | MYCN | SHOX2 | ZNF15L1 | |
| ELF5 | HSPC018 | MYF5 | SIM1 | ZNF165 | |
| EMX1 | HSPX153 | NCOA3 | SIX6 | ZNF177 | |
| EN1 | KIAA0040 | NEUD4 | SMARCA1 | ZNF179 | |
| EPAS1 | KIAA0071 | NEUROD6 | SMARCC1 | ZNF18 | |
| ERCC2 | KIAA0156 | NEUROG1 | SNAPC3 | ZNF184 | |
| ERF | KIAA0164 | NFATC1 | SNAPC4 | ZNF192 | |
| ESR1 | KIAA0244 | NFE2L2 | SNAPC5 | ZNF20 | |
| ESRRA | KIAA0306 | NFIB | SOX3 | ZNF200 | |
| ETV5 | KIAA0469 | NFKBIL1 | SOX30 | ZNF207 | |
| FHL2 | KIAA0535 | NFYA | SP2 | ZNF21 | |
| FLJ10469 | KIAA0943 | NR1H2 | SP3 | ZNF214 | |
| FLJ10697 | KIAA0972 | NR6A1 | SRF | ZNF217 | |
| FLJ11191 | KIAA0998 | NSEP1 | STAT1 | ZNF22 | |
| FLJ12606 | KIAA1528 | NYCM | STAT2 | ZNF229 | |
| FLJ13222 | KLF4 | OAZ | STAT3 | ZNF230 | |
| FLJ13659 | KLF7 | p100 | TAF1B | ZNF267 | |
| FLJ20039 | KLHL4 | P1P373C6 | TAL1 | ZNF271 | |
| FLJ20595 | LDB2 | PAF65B | TBR1 | ZNF275 | |
| FMR2 | LDOC1 | PAX6 | TCF7L2 | ZNF277 | |
| FOXC2 | LHX6 | PER3 | TCFL4 | ZNF281 | |
| FOXE2 | LMO2 | PFDN5 | TEAD1 | ZNF288 | |
| FOXP1 | LMO6 | PHTF1 | TEL2 | ZNF29 | |
| GABPB1 | LMO7 | PIASX-BETA | TFAP2A | ZNF294 | |
| GAS41 | LOC51045 | PMX1 | TFAP2B | ZNF6 | |
| GBX2 | LOC51087 | PPARG | TFAP2C | ZNF75A | |
| GCMB | LOC51131 | PRDM12 | TFDP1 | ZNF9 | |
| GIOT-3 | LOC51132 | PRDM16 | TFEB | ZNF90 | |
| GLI2 | LOC51290 | PRDM4 | TGFB1I1 | ZNF93 | |
| GTF2A2 | LOC65243 | PRDM5 | THRB | ZXDA | |
|  |  |  |  |  | |
| 24 HR - 48 HR CO-EXPRESSION - 70 GENES | | | | |  |
| ARNTL | EYA3 | HSSOX6 | NFKBIL2 | TBX2 | |
| BARHL1 | FLJ10734 | HTLF | NFX1 | YAF2 | |
| BHLHB3 | FLJ14549 | H_GS165L15 | NR4A1 | ZFP92 | |
| BRD3 | FLJ20531 | ID1 | NRIP1 | ZFPL1 | |
| BRPF3 | FLJ21603 | ILF3 | PBX2 | ZFX | |
| BTF3L2 | FLJ22252 | IRF3 | PIG7 | ZNF-kaiso | |
| CBX5 | FLJ23309 | KIAA0161 | RING1 | ZNF169 | |
| CE1 | FOS | KIAA1041 | SCAND2 | ZNF19 | |
| CHD3 | FOXJ1 | KIAA1542 | SIAH1 | ZNF211 | |
| CRSP3 | GLP | KLF3 | SIX2 | ZNF213 | |
| CXorf6 | HLXB9 | LHX2 | SSX5 | ZNF216 | |
| DLX3 | HOXA9 | LMO1 | SUPT6H | ZNF236 | |
| EDR1 | HOXB1 | MYCBP | TAF2C2 | ZNF254 | |
| ELF2 | HOXC6 | NFKBIA | TBX18 | ZNF273 | |
|  |  |  |  |  | |
| 0 HR - 24 HR - 48 HR CO-EXPRESSION - 367 GENES | | | | |  |
| ADNP | FLJ12644 | IRX7 | NFYB | TAF-172 | |
| AF020591 | FLJ14967 | JMJ | NFYC | TAF2C1 | |
| AF5Q31 | FLJ20244 | JUND | NKX3A | TAF2H | |
| AR | FLJ20392 | KIAA0014 | NR1H3 | TAF2S | |
| ASH1 | FLJ22301 | KIAA0026 | NR1I2 | TAF3B2 | |
| ASH2L | FOSB | KIAA0173 | NR1I3 | TBP | |
| ATBF1 | FOSL1 | KIAA0237 | NR2E1 | TBX15 | |
| ATRX | FOXB1 | KIAA0326 | NRF | TBX21 | |
| BACH2 | FOXE1 | KIAA0352 | NRL | TBX5 | |
| BAZ1B | FOXH1 | KIAA0395 | OCT11 | TBX6 | |
| BCL11A | FOXL2 | KIAA0414 | P38IP | TCF-3 | |
| BCL11B | FOXM1 | KIAA0478 | PAF65A | TCF19 | |
| BLZF1 | FOXO3A | KIAA0602 | PAX1 | TCF21 | |
| BRD1 | GABPA | KIAA0669 | PAX2 | TCF4 | |
| BRD2 | GATA4 | KIAA0798 | PBX1 | TCF8 | |
| BRD4 | GATA6 | KIAA1190 | PBX4 | TCFL1 | |
| BRDT | GBX1 | KIAA1321 | PDEF | TCFL5 | |
| BS69 | GCMA | KIAA1431 | PER2 | TEAD3 | |
| BTF3 | GFI1B | LAF4 | PHAP1 | TFCP2 | |
| BTF3L1 | GIOT-2 | LBP-9 | PITX2 | TFDP2 | |
| C11orf9 | GLIS2 | LBX1 | PKNOX2 | TFEC | |
| C21orf18 | GTF2A1 | LHX5 | PLAGL2 | THG-1 | |
| CBX4 | GTF2B | LIM | PMX2B | TNRC12 | |
| CCT4 | GTF2E2 | LMX1B | POU2AF1 | TNRC18 | |
| CDK7 | GTF2H2 | LOC51036 | POU3F2 | TNRC4 | |
| CDK8 | GTF2H4 | LOC51042 | POU4F1 | TNRC5 | |
| CDX1 | GTF3A | LOC51043 | POU4F2 | TNRC9 | |
| CE4 | GTF3C1 | LOC51058 | POU6F1 | TONDU | |
| CEBPB | GTF3C3 | LOC51088 | PP3501 | TRIM15 | |
| CEBPG | GTF3C4 | LOC51186 | PPARBP | TRIM22 | |
| CIAO1 | H-L(3)MBT | LOC51193 | PRDM10 | TRIP6 | |
| CITED1 | HAND2 | LOC51270 | PRDM13 | TZFP | |
| CITED2 | HBOA | LOC51652 | PRDM2 | UBTF | |
| CNOT4 | HCNGP | LOC55893 | PRDM7 | USF1 | |
| CORO1A | HES2 | LOC57167 | PREB | VENTX2 | |
| CREB1 | HES7 | LOC57209 | PROP1 | WHSC1 | |
| CREB3 | HESX1 | LOC58500 | PSMC5 | XBP1 | |
| CREBL2 | HEY1 | LOC91120 | PTTG1IP | ZFP36 | |
| CRIP2 | HHEX | LOC91614 | PURA | ZFR | |
| CRSP9 | HIF1A | LZLP | RAI15 | ZFY | |
| CSDA | HIS1 | MAD4 | RBBP5 | ZHX1 | |
| CSRP1 | HIVEP2 | MADH1 | REL | ZIC2 | |
| CSRP2 | HKR1 | MADH2 | REQ | ZIC3 | |
| CUTL1 | HLX1 | MADH6 | RFP | ZIC5 | |
| DEAF1 | HMG2 | MAFG | RFX3 | ZID | |
| DKFZP434B0335 | HMG20B | MAPK8IP1 | RNF4 | ZIM2 | |
| DKFZP434P1750 | HMGIC | MAX | RORC | ZNF10 | |
| DKFZp762K2015 | HMGIY | MBLL | SALL1 | ZNF133 | |
| DKFZp762M136 | HMX2 | MECP2 | SALL2 | ZNF138 | |
| DLX1 | HNF3A | MEIS2 | SAP30 | ZNF147 | |
| DXYS155E | HNF4A | MEIS3 | SATB1 | ZNF174 | |
| E2F2 | HOX11 | MEOX2 | SETBP1 | ZNF187 | |
| EBF | HOXA11 | MGC11349 | SIAH2 | ZNF195 | |
| EED | HOXA4 | MGC15716 | SIM2 | ZNF205 | |
| EGR4 | HOXA5 | MLL2 | SIX4 | ZNF212 | |
| ELF3 | HOXB2 | MLLT2 | SLB | ZNF226 | |
| ELK3 | HOXB5 | MLLT6 | SMARCA2 | ZNF23 | |
| EN2 | HOXB6 | MNT | SMARCA4 | ZNF239 | |
| EOMES | HOXB7 | MORF | SMARCE1 | ZNF297 | |
| EPLIN | HOXB8 | MSC | SMCX | ZNF304 | |
| ERCC3 | HOXB9 | MTA1L1 | SNAI1 | ZNF306 | |
| ESRRB | HOXC13 | MTF1 | SOX11 | ZNF31 | |
| ESRRG | HOXC4 | MYBL2 | SOX2 | ZNF361 | |
| ETV3 | HOXC5 | MYC | SOX4 | ZNF37A | |
| ETV4 | HOXD12 | MYCL2 | SPI1 | ZNF43 | |
| EZH1 | HOXD8 | MYT2 | SREBF1 | ZNF44 | |
| FALZ | HS747E2A | NCOA1 | SSX1 | ZNF74 | |
| FHL1 | HSF1 | NEUROG2 | SSX2 | ZNF76 | |
| FLJ10142 | ID3 | NFE2L1 | SSX3 | ZNF79 | |
| FLJ10251 | ILF2 | NFIA | SSX4 | ZNF92 | |
| FLJ10298 | DNAJ | NFIC | SUPT4H1 | ZXDA/B | |
| FLJ10759 | IPF1 | NFIL3 | T |  | |
| FLJ10891 | IRF2 | NFKB2 | TADA2L |  | |
| FLJ12517 | IRX4 | NFKBIB | TADA3L |  | |
|  |  |  |  |  | |

Supplementary Table S20.

2 Utricle Laser Venn Diagram Gene List

| 30 MIN EXPRESSION - 61 GENES | | | | |
| --- | --- | --- | --- | --- |
| AF093680 | ID2 | RFX5 | UBP1 | ZNF225 |
| ARNTL | ILF3 | RNF10 | VDR | ZNF234 |
| ATF2 | ISGF3G | SIM1 | YY1 | ZNF256 |
| ATF6 | KLF15 | SP1 | ZFP289 | ZNF259 |
| BNC | LOC51173 | SPIB | ZNF124 | ZNF263 |
| BTF3 | MAFK | STAT4 | ZNF136 | ZNF265 |
| CBX8 | MEIS1 | TAF2D | ZNF157 | ZNF266 |
| CSRP3 | MYB | TAF2G | ZNF160 | ZNF272 |
| DMRT2 | NFE2 | TEAD4 | ZNF180 | ZNF33A |
| DRIL1 | NRF1 | TFAP4 | ZNF189 |  |
| ETS1 | PAX4 | TIF1 | ZNF195 |  |
| ETS2 | PML | TP73 | ZNF202 |  |
| FOXD1 | RARB | TRAP150 | ZNF215 |  |
|  |  |  |  |  |
| 1 HR EXPRESSION - 80 GENES | | | | |
| AF020591 | FLJ22252 | MAZ | PRDM9 | TCF12 |
| ARNT | GABPB1 | MNDA | RBL2 | TMF1 |
| AWP1 | GTF2F2 | MSX2 | REST | TRIM28 |
| BANP | HKR2 | MYF5 | RFX2 | TRIP4 |
| BARX1 | HNF4A | NCOA2 | RUNX1 | VSX1 |
| CBFA2T3 | HOXA11 | NFATC4 | RXRA | ZFP95 |
| CBX6 | HOXA2 | NFE2L1 | RXRG | ZNF135 |
| CE1 | HOXC10 | NFIX | SALF | ZNF137 |
| CREB3 | HOXD10 | NR3C1 | SOX10 | ZNF143 |
| CREG | HOXD3 | NR4A2 | SOX5 | ZNF145 |
| CRX | IRF4 | NR6A1 | STAT3 | ZNF184 |
| DATF1 | IRF6 | NYCM | STAT5A | ZNF19 |
| DMTF1 | KIAA0194 | PAX2 | STAT6 | ZNF192 |
| E2F6 | LBX1 | PLAGL1 | TAF2F | ZNF22 |
| ELF1 | LOC51132 | POU2F1 | TBX19 | ZNF229 |
| EYA2 | LOC56930 | PPARA | TBX22 | ZNF264 |
|  |  |  |  |  |
| 2 HR EXPRESSION - 21 GENES | | | | |
| CHES1 | HLF | POU3F4 | ZNF193 | ZNFN1A1 |
| CRSP8 | HSAJ2425 | RLF | ZNF216 |  |
| FACTP140 | LOC51087 | TAF1C | ZNF287 |  |
| FIP2 | MGC10772 | ZFP103 | ZNF295 |  |
| FLJ20531 | NFATC1 | ZNF133 | ZNF85 |  |
|  |  |  |  |  |
| 3 HR EXPRESSION - 17 GENES | | | | |
| BACH1 | E2F3 | LHX1 | PER1 | ZNF84 |
| BAZ1A | ELF2 | NCOA4 | ZNF219 |  |
| BTEB1 | HNF3G | NFAT5 | ZNF232 |  |
| DLX6 | IRF1 | NFATC3 | ZNF30 |  |
|  |  |  |  |  |
| 30 MIN - 1 HR CO-EXPRESSION - 23 GENES | | | | |
| ASH1 | FLJ20729 | PER3 | SMARCA1 | ZFP26 |
| CBFA2T1 | ISL1 | PIAS3 | TADA2L | ZNF278 |
| CBX1 | KIAA0462 | PRDM1 | TCF7 | ZNF35 |
| CSEN | LEF1 | RNF22 | ZF5128 |  |
| DR1 | NFKBIE | SHOX | ZFP106 |  |
|  |  |  |  |  |
| 1 HR - 2 HR CO-EXPRESSION - 28 GENES | | | | |
| C11orf9 | FKHL18 | HSU90653 | R32184_3 | TRIP11 |
| CART1 | FLJ10688 | ICSBP1 | RORA | ZNF177 |
| COPS5 | FLJ12457 | NKX2B | SIAH1 | ZNF200 |
| CUTL1 | FLJ22332 | NR2F6 | SNAPC1 | ZNF7 |
| EN1 | FOXB1 | PAX6 | SNW1 |  |
| EPLIN | HSGT1 | PPARGC1 | SOX14 |  |
|  |  |  |  |  |
| 2 HR - 3 HR CO-EXPRESSION - 10 GENES | | | | |
| GFI1 | GTF3C2 | KIAA0211 | MLL | REQ |
| GIOT-3 | HOXC12 | KIAA1388 | NR4A3 | ZNF91 |
|  |  |  |  |  |
| 30 MIN - 3 HR CO-EXPRESSION - 36 GENES | | | | |
| ATF1 | FHX | PGR | TEL2 | ZNF26 |
| ATF3 | IRF3 | PIASX-BETA | THG-1 | ZNF8 |
| CEZANNE | KIAA1041 | PRDM8 | TIF1GAMMA | ZNF81 |
| CTCF | KLF13 | SP4 | USF2 | ZNFN1A3 |
| DKFZP564F1422 | MEOX2 | SREBF2 | ZNF123 |  |
| E2F4 | MID1 | TAF2I | ZNF132 |  |
| E2F5 | MLLT3 | TAF2K | ZNF144 |  |
| EZH2 | NR3C2 | TBX18 | ZNF258 |  |
|  |  |  |  |  |
| 30 MIN - 2 HR CO-EXPRESSION - 37 GENES | | | | |
| ARC | GATA2 | PAX3 | TAF1A | ZNF213 |
| BRPF1 | HMX1 | PKNOX1 | TBX20 | ZNF25 |
| CDR2 | KIAA0014 | POU2F2 | TBX4 | ZNF261 |
| EGR2 | KIAA0961 | PRDM14 | TCF2 | ZNF73 |
| ELF4 | KIAA1442 | RBPSUHL | TCF8 | ZNF83 |
| ETV2 | LOC56270 | RFP2 | ZNF-U69274 |  |
| EYA1 | NFKB1 | SLUG | ZNF138 |  |
| FLJ10211 | NR0B1 | SUPT5H | ZNF147 |  |
|  |  |  |  |  |
| 1 HR - 3 HR CO-EXPRESSION - 59 GENES | | | | |
| AIB3 | GCN5L2 | HOXC5 | MYBL1 | RUNX2 |
| ATOH1 | GTF2H1 | HOXC8 | NFIA | SIM2 |
| ERCC2 | GTF3C1 | IGHMBP2 | NFKBIA | SRF |
| ERG | HESX1 | KIAA0535 | NPAS2 | TCF19 |
| ESRRG | HIRA | KLF12 | NR5A2 | TIEG |
| EYA3 | HMG20B | LMO7 | NSEP1 | WT1 |
| FLI1 | HOX11 | LOC51045 | PAX8 | ZFR |
| FLJ125 | HOXA9 | LYL1 | PBX1 | ZNF151 |
| FOXM1 | HOXB1 | MAF | PHTF1 | ZNF16 |
| FOXO1A | HOXB2 | MEF2A | PPARBP | ZNF286 |
| GABPA | HOXB7 | MLLT1 | PROX1 | ZNF29 |
| GATA6 | HOXB8 | MXI1 | RFX4 |  |
|  |  |  |  |  |
| 30 MIN - 1 HR - 2 HR CO-EXPRESSION - 65 GENES | | | | |
| AHR | GAS41 | KIAA1668 | RNF24 | TRPS1 |
| BHLHB2 | GASC1 | LOC57862 | RRN3 | YAF2 |
| CDX2 | GCN5L1 | M6A | SAP18 | ZFX |
| CIR | GRLF1 | MED6 | SNAPC5 | ZIC1 |
| CRIP1 | GSH2 | MEFV | SOX3 | ZNF11B |
| CRSP6 | GTF2I | MLLT7 | SRA1 | ZNF169 |
| DFKZP434E026 | HLX1 | NAB1 | SUPT3H | ZNF208 |
| DLX4 | HOXC11 | NR0B2 | TAF2A | ZNF221 |
| DSIPI | HOXC13 | PFDN5 | TAF2C2 | ZNF254 |
| ELF5 | HOXC9 | PLRG1 | TAF2J | ZNF288 |
| ELK4 | HSF4 | PMF1 | TAL1 | ZNF306 |
| EOMES | IPF1 | PPARG | THRA | ZNF9 |
| FUBP1 | IRF5 | RBBP9 | TRIP13 | ZNF92 |
|  |  |  |  |  |
| 1 HR - 2 HR - 3 HR CO-EXPRESSION - 86 GENES | | | | |
| ATF5 | FLJ20557 | KLF4 | p100 | TEAD1 |
| BRD2 | FOXF2 | LHX4 | PEGASUS | TEF |
| BRD3 | FOXI1 | LHX5 | POU4F2 | TFCP2 |
| BRPF3 | GATA4 | LMO2 | PRDM12 | TGFB1I1 |
| BS69 | GLI2 | LMO6 | PRDM15 | ZFP |
| C1orf2 | GTF2H2 | MDS1 | PRDM4 | ZHX1 |
| CREBBP | GTF2H4 | MEIS3 | PRDM7 | ZIC5 |
| CREBL1 | GTF3C4 | MGC11349 | REL | ZNF-kaiso |
| DKFZp762M136 | HCF2 | MILD1 | SETDB1 | ZNF14 |
| DLX2 | HNF4G | MTA1 | SIX1 | ZNF141 |
| DUX2 | HOXA1 | MYBBP1A | SIX2 | ZNF207 |
| EBF | HOXA10 | MYOG | SNAPC3 | ZNF267 |
| EGR1 | HOXA7 | MYT1L | SRCAP | ZNF268 |
| EGR3 | HRIHFB2436 | NEUROD1 | SSX1 | ZNF37A |
| ELK3 | HRY | NEUROD6 | TAF2E |  |
| EPAS1 | HSSOX6 | NPAS1 | TBX1 |  |
| ERF | KIAA0478 | NR1H2 | TBX3 |  |
| ESRRA | KIAA0943 | NR2F1 | TCFL4 |  |
|  |  |  |  |  |
| 30 MIN - 2 HR - 3 HR CO-EXPRESSION 62 GENES | | | | |
| ABT1 | DBP | HNF3B | NFE2L2 | SMARCB1 |
| ATBF1 | DKFZP434B195 | HOXB9 | NFE2L3 | SOX11 |
| BRF2 | ESR2 | HSPC189 | NFIL3 | SUPT6H |
| BTF3L2 | FLJ12517 | HYPH | NFKBIB | T |
| CBX5 | FLJ12525 | H_GS165L15 | PAX1 | TCF7L2 |
| CDX1 | FLJ13222 | KIAA0304 | PBX2 | TEAD2 |
| CE3 | FLJ20039 | LHX2 | PBX3 | ZNF173 |
| CEBPE | FLJ23309 | LHX9 | POU6F1 | ZNF297 |
| CHD2 | GATA1 | LOC51186 | PROP1 | ZNF45 |
| CLOCK | GTF2E1 | LOC51637 | RARG | ZNF76 |
| COPEB | HBOA | MADH3 | RELA |  |
| CREM | HEY1 | NCOA3 | SIX3 |  |
| DACH | HIVEP1 | NCOR1 | SLB |  |
|  |  |  |  |  |
| 30 MIN - 1 HR - 3 HR CO-EXPRESSION - 67 GENES | | | | |
| BARHL1 | FLJ20392 | KRML | NRL | TFAP2C |
| BRD7 | FOSL2 | LHX6 | ONECUT2 | TIMELESS |
| BRDT | GTF2B | LMO4 | PMX1 | USF1 |
| CE4 | GTF2H3 | LOC51042 | POU1F1 | ZFHX1B |
| CRSP3 | GTF3C5 | LOC51290 | POU3F2 | ZIC3 |
| CSDA | HHEX | LOC91614 | R28830_2 | ZNF20 |
| CXorf6 | HOXD12 | MAD | RFXANK | ZNF23 |
| DDIT3 | HOXD4 | MGC12942 | RNF2 | ZNF262 |
| DLX1 | IRX7 | MYT1 | SCAND2 | ZNF275 |
| EDR1 | KIAA0132 | NFATC2 | SMARCC2 | ZNF304 |
| ESRRB | KIAA0222 | NFYA | SREBF1 | ZNF74 |
| ETV3 | KIAA0469 | NFYB | SSRP1 |  |
| FALZ | KIAA0798 | NR1D1 | TBX10 |  |
| FLJ10734 | KLF3 | NR2C1 | TCF4 |  |
|  |  |  |  |  |
| 30 MIN - 1 HR - 2 HR - 3 HR CO-EXPRESSION - 535 GENES | | | | |
| ADNP | FOS | KIAA0414 | NR1I2 | TAF1B |
| AF5Q31 | FOSB | KIAA0602 | NR1I3 | TAF2B |
| ALY | FOSL1 | KIAA0669 | NR2E1 | TAF2C1 |
| AR | FOXC1 | KIAA0700 | NR2E3 | TAF2H |
| ARIX | FOXC2 | KIAA0952 | NR2F2 | TAF2N |
| ARNT2 | FOXE1 | KIAA0972 | NR4A1 | TAF2S |
| ASH2L | FOXE2 | KIAA0998 | NR5A1 | TAF3B2 |
| ATF4 | FOXH1 | KIAA1190 | NRF | TAL2 |
| ATF7 | FOXJ1 | KIAA1321 | NRIP1 | TBP |
| ATRX | FOXL2 | KIAA1431 | OAZ | TBPL1 |
| BACH2 | FOXO3A | KIAA1528 | OCT11 | TBR1 |
| BAPX1 | FOXP1 | KIAA1542 | OG2x | TBX15 |
| BAZ1B | GATA3 | KLF5 | OVOL1 | TBX2 |
| BAZ2A | GBX1 | KLHL4 | P1P373C6 | TBX21 |
| BAZ2B | GBX2 | LAF4 | P38IP | TBX5 |
| BC002881 | GCMA | LBP-9 | PAF65A | TBX6 |
| BCL11A | GCMB | LDB1 | PAF65B | TCEAL1 |
| BCL11B | GFI1B | LDB2 | PAX5 | TCF-3 |
| BHLHB3 | GIOT-2 | LDOC1 | PAX7 | TCF21 |
| BLZF1 | GLI3 | LIM | PBX4 | TCF3 |
| BRD1 | GLIS2 | LMO1 | PC4 | TCFL1 |
| BRD4 | GLP | LMX1B | PCAR | TCFL5 |
| BTF3L1 | GTF2A1 | LOC51036 | PDEF | TEAD3 |
| C21orf18 | GTF2A2 | LOC51043 | PER2 | TFAP2A |
| C5orf7 | GTF2E2 | LOC51058 | PHAP1 | TFDP1 |
| CBX3 | GTF2F1 | LOC51088 | PIG7 | TFDP2 |
| CBX4 | GTF3A | LOC51131 | PILB | TFE3 |
| CCT4 | GTF3C3 | LOC51193 | PITX2 | TFEB |
| CDK7 | H-L(3)MBT | LOC51270 | PKNOX2 | TFEC |
| CDK8 | HAND2 | LOC51652 | PLAG1 | TGIF |
| CEBPB | HCNGP | LOC55885 | PLAGL2 | THRB |
| CEBPG | HDAC1 | LOC55893 | PMX2B | TIEG2 |
| CERD4 | HDAC2 | LOC57167 | POU2AF1 | TITF1 |
| CHD3 | HDAC4 | LOC57209 | POU4F1 | TNRC12 |
| CIAO1 | HES2 | LOC58500 | POU5F1 | TNRC18 |
| CITED1 | HES7 | LOC65243 | PP3501 | TNRC3 |
| CITED2 | HEY2 | LOC91120 | PPARD | TNRC4 |
| CL469780 | HEYL | LOC92283 | PRDM10 | TNRC5 |
| CNOT3 | HIF1A | LW-1 | PRDM11 | TNRC6 |
| CNOT4 | HIS1 | LZLP | PRDM13 | TNRC9 |
| CORO1A | HIVEP2 | LZTR1 | PRDM16 | TONDU |
| CREB1 | HKR1 | LZTS1 | PRDM2 | TP53 |
| CREBL2 | HKR3 | M96 | PRDM5 | TRIM15 |
| CRIP2 | HLXB9 | MAD4 | PRDM6 | TRIM22 |
| CROC4 | HMG2 | MADH1 | PREB | TRIP15 |
| CRSP7 | HMGIC | MADH2 | PSMC5 | TRIP6 |
| CRSP9 | HMGIY | MADH4 | PTTG1IP | TSC22 |
| CSRP1 | HMX2 | MADH5 | PURA | TZFP |
| CSRP2 | HNF3A | MADH6 | RAI15 | UBTF |
| CTNNB1 | HOX11L | MADH7 | RARA | UTF1 |
| DEAF1 | HOXA13 | MADH9 | RBBP5 | VENTX2 |
| DKFZP434B0335 | HOXA3 | MAFF | RBL1 | WHSC1 |
| DKFZP434N043 | HOXA4 | MAFG | RELB | XBP1 |
| DKFZP434P1750 | HOXA5 | MAPK8IP1 | RERE | ZFP36 |
| DKFZp547H236 | HOXA6 | MAX | RFP | ZFP91 |
| DKFZp762K2015 | HOXB13 | MBLL | RFX3 | ZFP92 |
| DLX3 | HOXB3 | MDS032 | RGC32 | ZFP93 |
| DRAP1 | HOXB5 | MECP2 | RING1 | ZFPL1 |
| DRPLA | HOXB6 | MEF2B | RNF13 | ZFY |
| DUX4 | HOXC4 | MEF2C | RNF14 | ZIC2 |
| DXYS155E | HOXC6 | MEF2D | RNF3 | ZIC4 |
| E2F2 | HOXD1 | MEIS2 | RNF4 | ZID |
| E4F1 | HOXD11 | MGC15716 | RNF8 | ZIM2 |
| EED | HOXD13 | MGC16733 | RORB | ZNF10 |
| EGR4 | HOXD8 | MGC2508 | RORC | ZNF131 |
| EHF | HOXD9 | MHC2TA | RREB1 | ZNF134 |
| ELF3 | HR | MITF | SAFB | ZNF142 |
| ELK1 | HRIHFB2122 | MLL2 | SALL1 | ZNF146 |
| EMX1 | HS747E2A | MLLT10 | SALL2 | ZNF155 |
| EMX2 | HSA275986 | MLLT2 | SALL3 | ZNF15L1 |
| EN2 | HSF1 | MLLT4 | SAP30 | ZNF161 |
| EP300 | HSF2BP | MLLT6 | SATB1 | ZNF162 |
| ERCC3 | HSHPX5 | MNAT1 | SBB103 | ZNF174 |
| ERCC6 | HSPC018 | MNT | SCML2 | ZNF179 |
| ESR1 | HSPX153 | MORF | SDCCAG33 | ZNF183 |
| ETV1 | HSU79252 | MSC | SETBP1 | ZNF185 |
| ETV4 | HTLF | MTA1L1 | SHARP | ZNF187 |
| ETV5 | ICBP90 | MTF1 | SHOX2 | ZNF205 |
| ETV6 | ID1 | MYBL2 | SIAH2 | ZNF21 |
| EVX1 | ID3 | MYC | SIX4 | ZNF211 |
| EYA4 | ID4 | MYCBP | SIX6 | ZNF212 |
| EZH1 | ILF1 | MYCL1 | SMARCA2 | ZNF220 |
| FHL1 | ILF2 | MYCL2 | SMARCA3 | ZNF230 |
| FHL2 | DNAJ | MYCN | SMARCA4 | ZNF236 |
| FLJ10142 | IRF2 | MYF6 | SMARCC1 | ZNF239 |
| FLJ10251 | IRF7 | MYOD1 | SMARCE1 | ZNF271 |
| FLJ10298 | IRLB | MYT2 | SMCX | ZNF273 |
| FLJ10469 | IRX4 | NCOA1 | SNAI1 | ZNF274 |
| FLJ10697 | IRX5 | NCOR2 | SNAPC4 | ZNF277 |
| FLJ10759 | JMJ | NEUD4 | SOX13 | ZNF281 |
| FLJ10891 | JUN | NEUROD4 | SOX2 | ZNF294 |
| FLJ11186 | JUND | NEUROG1 | SOX30 | ZNF31 |
| FLJ11191 | KIAA0026 | NEUROG2 | SOX4 | ZNF32 |
| FLJ12606 | KIAA0040 | NFIB | SP2 | ZNF361 |
| FLJ12644 | KIAA0071 | NFIC | SP3 | ZNF38 |
| FLJ12827 | KIAA0130 | NFKB2 | SPI1 | ZNF41 |
| FLJ13590 | KIAA0156 | NFKBIL1 | SRY | ZNF43 |
| FLJ13659 | KIAA0161 | NFKBIL2 | SSX2 | ZNF44 |
| FLJ14549 | KIAA0164 | NFRKB | SSX3 | ZNF46 |
| FLJ14967 | KIAA0173 | NFX1 | SSX4 | ZNF6 |
| FLJ20244 | KIAA0237 | NFYC | SSX5 | ZNF75A |
| FLJ20321 | KIAA0244 | NHLH2 | STAT1 | ZNF79 |
| FLJ20595 | KIAA0293 | NKX3A | STAT2 | ZNF80 |
| FLJ21603 | KIAA0306 | NKX6A | SUPT4H1 | ZNF90 |
| FLJ22301 | KIAA0326 | NMI | SURB7 | ZNF93 |
| FMR2 | KIAA0352 | NR1D2 | TADA3L | ZXDA |
| FOG2 | KIAA0395 | NR1H3 | TAF-172 | ZXDA/B |
|  |  |  |  |  |
