# Supplementary material for: Large Scale Gene Expression Profiles of Regenerating Inner Ear Sensory Epithelia
Source: PLoS One. 2007 Jun 13;2(6):e525. doi: 10.1371/journal.pone.0000525 (PMC1888727; doi:10.1371/journal.pone.0000525)
Supplement: Table S21 — Cochlea Detectably Expressed Genes (0.23 MB DOC) [file pone.0000525.s022.doc]

Supplementary Table S21.

1. Cochlea Neomycin Venn Diagram Gene List

| O HR EXPRESSION - 79 GENES | | | | | |
| --- | --- | --- | --- | --- | --- |
| AHR | FLJ10734 | KLF8 | NFE2L2 | TBX4 | |
| ALF | FOSL2 | KRML | ONECUT1 | TCF12 | |
| ATF3 | FOXI1 | LHX1 | PGR | TCF2 | |
| ATF4 | FOXO1A | LOC51173 | POU3F4 | TCF21 | |
| ATF6 | GTF2H1 | MADH9 | POU4F3 | TCF3 | |
| BARX2 | GTF3C2 | MEF2A | POU6F1 | TGIF | |
| BCL6 | GTF3C4 | MEOX1 | SCAND2 | TP73 | |
| BRD7 | HNF4G | MEOX2 | SOX14 | TSC22 | |
| CE1mm3 | HOXB9 | MID1 | SOX9 | UBP1 | |
| CLOCK | HOXC12 | MLL | STAT4 | USF1 | |
| DBP | ISGF3G | MT520 | STAT5B | USF2 | |
| DED | KIAA0211 | MYT1L | SUPT5H | ZNF131 | |
| DFKZP434E026 | KIAA1041 | NCOA4 | T | ZNF229 | |
| E2F1 | KIAA1542 | NFAT5 | TADA2L | ZNF275 | |
| EOMES | KLF13 | NFATC3 | TAF2G | ZNF44 | |
| ESRRB | KLF5 | NFE2 | TBX20 |  | |
|  |  |  |  |  | |
| 24 HR EXPRESSION – 121 GENES | | | | | |
| AF020591 | E2F4 | HOXD3 | PAX8 | ZFX | |
| AF093680 | ELF5 | HOXD4 | PER3 | ZIC5 | |
| AIB3 | EPAS1 | IRF4 | PHTF1 | ZNF123 | |
| ALY | ERCC3 | ISL1 | POU2F2 | ZNF141 | |
| ARNT | ERG | KIAA0478 | POU3F2 | ZNF142 | |
| ATF1 | ESRRG | KIAA0952 | PPARA | ZNF173 | |
| BRDT | ETV4 | LDB2 | PRDM5 | ZNF180 | |
| BTF3L1 | FALZ | LEF1 | R28830_2 | ZNF20 | |
| CBFA2T1 | FLJ10469 | LOC51087 | RB1 | ZNF220 | |
| CBFA2T3 | FLJ12525 | LOC51132 | RBL2 | ZNF230 | |
| CDK7 | FLJ22252 | MAZ | REST | ZNF262 | |
| CDX1 | FLJ22332 | MGC16733 | RNF3 | ZNF265 | |
| CDX4 | FOS | MORF | SHARP | ZNF274 | |
| CE1 | GABPB1 | MSX2 | SHOX | ZNF294 | |
| CEZANNE | GASC1 | MXI1 | SHOX2 | ZNF361 | |
| CHD4 | GCMB | MYBBP1A | SMARCA1 | ZNF74 | |
| CIR | GTF2F2 | MYCL1 | SOX10 | ZNF8 | |
| CRX | HIVEP2 | NEUROG2 | SRF | ZNF81 | |
| DACH | HKR2 | NFATC4 | TAF2C2 | ZNF83 | |
| DDIT3 | HMG20B | NFIA | TAF2S | ZNF91 | |
| DLX2 | HOXA10 | NFIX | TBX19 | ZNFN1A3 | |
| DLX5 | HOXC13 | NFYB | TEAD1 |  | |
| DMTF1 | HOXC5 | NPAS2 | TFAP2A |  | |
| DSIPI | HOXD10 | NR1I2 | TIMELESS |  | |
| E2F3 | HOXD11 | OTX2 | ZFHX1B |  | |
|  |  |  |  |  | |
| 48 HR EXPRESSION – 87 GENES | | | | | |
| BRD3 | FLJ20392 | MYOD1 | RBPSUHL | TRAP150 | |
| CBX5 | FUBP1 | MYOG | RNF15 | YY1 | |
| CDR2 | GATA4 | NAB1 | RORA | ZFP289 | |
| CHES1 | GCMA | NCOR2 | RPF-1 | ZFP37 | |
| CREBL2 | GTF2I | NEUROD6 | RUNX1 | ZFPL1 | |
| CREG | HHEX | NFATC1 | RUNX2 | ZIC1 | |
| CSRP3 | HOXA1 | NFE2L3 | SBB103 | ZNF11B | |
| EGR2 | HOXD9 | NFKB1 | SIAH1 | ZNF134 | |
| ELK1 | KIAA0194 | NFKBIL2 | SNAPC4 | ZNF266 | |
| ELK4 | KIAA0441 | NR2C1 | SNW1 | ZNF272 | |
| EN1 | KIAA1442 | NR2F1 | SRCAP | ZNF282 | |
| ERCC2 | KIAA1668 | NR2F2 | TAF1C | ZNF297 | |
| ESRRA | LMX1B | PAX3 | TAF2E | ZNF3 | |
| ETS1 | LOC51045 | PFDN5 | TAF2J | ZNF38 | |
| FACTP140 | MEF2C | PMF1 | TBX5 | ZNF46 | |
| FIP2 | MEF2D | PPARGC1 | TCF8 |  | |
| FLJ10211 | MITF | PRDM9 | TEAD4 |  | |
| FLJ10688 | MLLT7 | RBBP9 | TGFB1I1 |  | |
|  |  |  |  |  | |
| O HR - 24 HR CO-EXPRESSION – 85 GENES | | | | | |
| AR | DRPLA | HNF3A | NFYA | SUPT6H | |
| ATF7 | EDR1 | HOXA13 | NKX3A | TAF2I | |
| BACH2 | EGR3 | HOXB1 | NKX6A | TEAD2 | |
| BARHL1 | EMX2 | HOXB13 | NR2E1 | TFCP2 | |
| BATF | EN2 | IRX4 | NR4A2 | TFDP2 | |
| BS69 | ETV3 | KIAA0071 | PAX1 | TFEB | |
| C1orf2 | FLI1 | KIAA0304 | PAX9 | TIF1 | |
| CBF2 | FLJ14549 | KIAA0352 | PIAS3 | TNRC5 | |
| CE4 | FLJ20039 | KIAA0669 | PML | TNRC6 | |
| CEBPE | FLJ23309 | KIAA1528 | PMX2B | TRIM15 | |
| CREBL1 | FOXM1 | LOC51186 | PPARG | TRIM28 | |
| CREM | GATA1 | LOC51193 | RFX5 | ZFP92 | |
| CSEN | GFI1 | LYL1 | RNF22 | ZFR | |
| DKFZP434B0335 | GFI1B | MADH6 | SAP30 | ZNF174 | |
| DKFZP434N043 | GTF2B | MECP2 | SPI1 | ZNF211 | |
| DMRT2 | HIRA | MILD1 | STAT2 | ZNF76 | |
| DRIL1 | HIVEP1 | NFIL3 | STAT6 | ZNF92 | |
|  |  |  |  |  | |
| O HR - 48 HR CO-EXPRESSION – 120 GENES | | | | | |
| BC002881 | GLI | LOC51043 | RARB | SSRP1 | |
| BTF3 | GRLF1 | LOC51637 | RARG | TAF1A | |
| CBX1 | GSH2 | MAFK | RBL1 | TAF2K | |
| CE3 | GTF3A | MEFV | REL | TAF3B2 | |
| CITED1 | HEYL | MLLT3 | RELA | TAL2 | |
| CNOT8 | HKR3 | MYB | RFP2 | TBX15 | |
| COPEB | HMG2 | MYCN | RNF24 | TBX3 | |
| CROC4 | HOXA2 | NAB2 | RNF4 | TFAP2B | |
| CRSP7 | HOXA4 | NFKBIE | RNF8 | THRB | |
| CSDA | HOXA6 | NFX1 | RORC | TRIP15 | |
| DKFZp547H236 | HOXA9 | NKX2B | RXRB | TZFP | |
| DLX3 | HOXB7 | NR1H3 | SALL1 | ZFP93 | |
| DR1 | HOXC9 | NR2F6 | SATB1 | ZNF124 | |
| EED | HSF1 | NRIP1 | SIX3 | ZNF15L1 | |
| ELF2 | ILF1 | OAZ | SIX6 | ZNF185 | |
| ETV5 | IRF1 | P38IP | SLUG | ZNF189 | |
| FLJ125 | IRF7 | PAX4 | SMARCC2 | ZNF200 | |
| FLJ14967 | IRX5 | PCAR | SNAPC2 | ZNF212 | |
| FOSB | KIAA0293 | PILB | SOX13 | ZNF236 | |
| FOXE2 | KIAA1321 | POU2AF1 | SOX30 | ZNF281 | |
| FOXF1 | LDB1 | PRDM15 | SP3 | ZNF288 | |
| GABPA | LDOC1 | PRDM16 | SP4 | ZNF306 | |
| GATA2 | LHX4 | PRDM6 | SPIB | ZNF93 | |
| GBX2 | LMO1 | PROX1 | SREBF2 | ZXDA/B | |
|  |  |  |  |  | |
| 24 HR - 48 HR CO-EXPRESSION - 138 GENES | | | | |  |
| ATF5 | FMR2 | HSSOX6 | NFYC | TBPL1 | |
| BAZ1A | FOXB1 | ICSBP1 | NHLH2 | TBR1 | |
| BAZ2A | FOXF2 | IRF6 | NPAS1 | TBX10 | |
| BC011982 | FOXH1 | KIAA0395 | NR1D1 | TCF7 | |
| BHLHB2 | FOXL2 | KIAA0414 | NR1H2 | TCFL1 | |
| BLZF1 | FOXO3A | KIAA0602 | NR6A1 | TCFL4 | |
| BRD1 | GAS41 | KIAA0943 | PER2 | THRA | |
| BRPF1 | GATA6 | KIAA0972 | PHAP1 | TIEG | |
| CBX3 | GCN5L2 | KIAA0998 | PKNOX2 | TNRC3 | |
| COPS5 | GIOT-3 | KIAA1388 | PLAGL1 | YAF2 | |
| CREBBP | GLI2 | KLF3 | PLAGL2 | ZFP26 | |
| CRIP1 | GLIS2 | KLF4 | PRDM12 | ZFY | |
| CRSP3 | GTF2E2 | LBX1 | R32184_3 | ZID | |
| DKFZp762M136 | GTF2H4 | LHX2 | RAI15 | ZIM2 | |
| DRAP1 | HAND2 | LMO2 | RBBP5 | ZNF133 | |
| DUX2 | HDAC2 | LOC51270 | REQ | ZNF146 | |
| E4F1 | HESX1 | LOC51290 | RERE | ZNF160 | |
| ELF1 | HIS1 | LOC55885 | RFP | ZNF169 | |
| EPLIN | HKR1 | LOC57209 | RFXANK | ZNF221 | |
| ERF | HLF | LOC91614 | RNF13 | ZNF254 | |
| EYA2 | HMX1 | M96 | RNF2 | ZNF273 | |
| EYA3 | HOXA11 | MNAT1 | RORB | ZNF278 | |
| EZH1 | HOXA7 | MYF5 | SAFB | ZNF31 | |
| FHL2 | HOXC8 | NCOA3 | SAP18 | ZNF32 | |
| FKHL18 | HSAJ2425 | NEUD4 | SETDB1 | ZNF6 | |
| FLJ12827 | HSHPX5 | NEUROD1 | SMARCA3 | ZNF75A | |
| FLJ20531 | HSPC018 | NFIC | SMCX |  | |
| FLJ20557 | HSPX153 | NFKBIB | TAL1 |  | |
|  |  |  |  |  | |
| 0 HR - 24 HR - 48 HR CO-EXPRESSION - 414 GENES | | | | |  |
| ABT1 | FLJ13590 | KIAA0040 | NCOA1 | SREBF1 | |
| ADNP | FLJ13659 | KIAA0130 | NEUROG1 | SSX1 | |
| AF5Q31 | FLJ20244 | KIAA0156 | NFATC2 | SSX2 | |
| ARC | FLJ20321 | KIAA0161 | NFE2L1 | SSX3 | |
| ARIX | FLJ20595 | KIAA0173 | NFIB | SSX4 | |
| ARNT2 | FLJ22301 | KIAA0237 | NFKB2 | SSX5 | |
| ASH2L | FOG2 | KIAA0244 | NFKBIA | STAT1 | |
| ATBF1 | FOSL1 | KIAA0306 | NFKBIL1 | STAT3 | |
| ATOH1 | FOXC1 | KIAA0326 | NFRKB | SUPT4H1 | |
| ATRX | FOXC2 | KIAA0462 | NR0B1 | SURB7 | |
| BAPX1 | FOXE1 | KIAA0700 | NR1D2 | TADA3L | |
| BAZ2B | FOXJ1 | KIAA0961 | NR1I3 | TAF-172 | |
| BCL11A | FOXP1 | KIAA1190 | NR2E3 | TAF2B | |
| BCL11B | GATA3 | KIAA1431 | NR4A1 | TAF2C1 | |
| BHLHB3 | GBX1 | KLF15 | NR5A1 | TAF2D | |
| BRD2 | GCN5L1 | KLHL4 | NR5A2 | TAF2H | |
| BRD4 | GIOT-2 | LAF4 | NRF | TAF2N | |
| BRF2 | GLI3 | LBP-9 | NRF1 | TBP | |
| BRPF3 | GLP | LHX5 | OCT11 | TBX18 | |
| BTF3L2 | GTF2A1 | LHX6 | OG2x | TBX2 | |
| C11orf9 | GTF2A2 | LIM | ONECUT2 | TBX21 | |
| C21orf18 | GTF2E1 | LMO4 | OVOL1 | TBX6 | |
| C5orf7 | GTF2F1 | LMO6 | p100 | TCEAL1 | |
| CART1 | GTF2H2 | LMO7 | P1P373C6 | TCF-3 | |
| CBX4 | GTF3C1 | LOC51036 | PAF65A | TCF4 | |
| CBX8 | GTF3C3 | LOC51042 | PAF65B | TCF7L2 | |
| CCT4 | H_GS165L15 | LOC51058 | PAX6 | TCFL5 | |
| CDK8 | HBOA | LOC51088 | PAX7 | TEAD3 | |
| CDX2 | HCNGP | LOC51131 | PBX4 | TEL2 | |
| CEBPB | HDAC1 | LOC51652 | PC4 | TFAP4 | |
| CERD4 | HDAC4 | LOC55893 | PDEF | TFDP1 | |
| CHD3 | HES2 | LOC56270 | PIASX-BETA | TFEC | |
| CIAO1 | HES7 | LOC57167 | PIG7 | TIEG2 | |
| CITED2 | HEY1 | LOC57862 | PITX2 | TITF1 | |
| CL469780 | HEY2 | LOC58500 | PLAG1 | TNRC12 | |
| CNOT3 | HIF1A | LOC91120 | PLRG1 | TNRC18 | |
| CNOT4 | H-L(3)MBT | LOC92283 | PMX1 | TNRC4 | |
| CORO1A | HLX1 | LW-1 | POU4F1 | TNRC9 | |
| CREB1 | HLXB9 | LZLP | POU5F1 | TONDU | |
| CRIP2 | HMGIC | LZTR1 | PP3501 | TP53 | |
| CRSP9 | HMGIY | LZTS1 | PPARBP | TRIM22 | |
| CSRP1 | HNF3B | M6A | PPARD | TRIP6 | |
| CSRP2 | HNF3G | MAD | PRDM1 | UBTF | |
| CTNNB1 | HOX11 | MAD4 | PRDM10 | UTF1 | |
| CUTL1 | HOX11L | MADH1 | PRDM11 | VENTX2 | |
| CXorf6 | HOXA3 | MADH2 | PRDM13 | WHSC1 | |
| DATF1 | HOXA5 | MADH3 | PRDM2 | XBP1 | |
| DEAF1 | HOXB2 | MADH4 | PRDM7 | ZFP36 | |
| DKFZP434B195 | HOXB3 | MADH5 | PREB | ZFP91 | |
| DKFZP434P1750 | HOXB5 | MADH7 | PROP1 | ZIC2 | |
| DKFZp762K2015 | HOXB6 | MAF | PSMC5 | ZIC3 | |
| DLX1 | HOXB8 | MAFF | PTTG1IP | ZIC4 | |
| DLX4 | HOXC11 | MAFG | PURA | ZNF10 | |
| DUX4 | HOXC4 | MAPK8IP1 | RARA | ZNF138 | |
| DXYS155E | HOXC6 | MAX | RELB | ZNF147 | |
| E2F2 | HOXD1 | MBLL | RFX2 | ZNF155 | |
| EBF | HOXD12 | MDS032 | RFX3 | ZNF157 | |
| EGR1 | HOXD13 | MDS1 | RGC32 | ZNF161 | |
| EGR4 | HOXD8 | MED6 | RING1 | ZNF162 | |
| ELF3 | HRIHFB2122 | MEF2B | RREB1 | ZNF179 | |
| ELK3 | HRIHFB2436 | MEIS2 | RXRA | ZNF187 | |
| EMX1 | HS747E2A | MEIS3 | SALL2 | ZNF205 | |
| EP300 | HSA275986 | MGC10772 | SALL3 | ZNF207 | |
| ERCC6 | HSF4 | MGC11349 | SCML2 | ZNF21 | |
| ESR1 | HSU90653 | MGC12942 | SDCCAG33 | ZNF213 | |
| ESR2 | HTLF | MGC15716 | SETBP1 | ZNF23 | |
| ETV1 | ICBP90 | MGC2508 | SIAH2 | ZNF234 | |
| ETV6 | ID1 | MHC2TA | SIM2 | ZNF239 | |
| EZH2 | ID3 | MLL2 | SIX1 | ZNF25 | |
| FHL1 | ID4 | MLLT10 | SIX2 | ZNF261 | |
| FHX | IGHMBP2 | MLLT2 | SIX4 | ZNF268 | |
| FLJ10142 | ILF2 | MLLT4 | SLB | ZNF271 | |
| FLJ10251 | DNAJ | MLLT6 | SMARCA2 | ZNF304 | |
| FLJ10298 | IPF1 | MNT | SMARCA4 | ZNF41 | |
| FLJ10697 | IRF2 | MSC | SMARCB1 | ZNF43 | |
| FLJ10759 | IRF5 | MTA1 | SMARCC1 | ZNF45 | |
| FLJ10891 | IRLB | MTA1L1 | SMARCE1 | ZNF79 | |
| FLJ11186 | IRX7 | MTF1 | SNAI1 | ZNF80 | |
| FLJ12457 | JMJ | MYBL2 | SNAPC5 | ZNF9 | |
| FLJ12517 | JUN | MYC | SOX11 | ZNF90 | |
| FLJ12606 | JUND | MYCL2 | SOX2 | ZNF-kaiso | |
| FLJ12644 | KIAA0014 | MYF6 | SOX4 | ZXDA | |
| FLJ13222 | KIAA0026 | MYT2 | SP2 |  | |
|  |  |  |  |  | |

Supplementary Table S21.

Cochlea Laser Venn Diagram Gene List

| 30 MIN EXPRESSION - 19 GENES | | | | |
| --- | --- | --- | --- | --- |
| BCL6 | GTF2H1 | TBX20 | EZH2 | ISGF3G |
| ELF2 | HNF4G | MADH9 | HIRA | LHX1 |
| FLJ125 | KIAA0441 | CDX4 | HOXD4 | NR2C2 |
| GTF2F2 | MEOX2 | ERCC3 | HOXC4 |  |
|  |  |  |  |  |
| 1 HR EXPRESSION - 40 GENES | | | | |
| BACH2 | ESRRG | NCOA4 | SOX4 | ZNF226 |
| BARX2 | FLJ20392 | NFAT5 | SOX5 | ZNF26 |
| BRD1 | GATA1 | NFIX | SOX9 | ZNF262 |
| CBFA2T1 | HESX1 | NKX3A | SRA1 | ZNF295 |
| CDX1 | KLF5 | RB1 | TCF21 | ZNF31 |
| DDIT3 | LOC51045 | RBL2 | TRIM28 | ZNF81 |
| DLX2 | MXI1 | RORA | ZNF132 | ZNF91 |
| DLX5 | MYBBP1A | SMARCA4 | ZNF151 | ZNFN1A3 |
|  |  |  |  |  |
| 2 HR EXPRESSION - 30 GENES | | | | |
| SUPT3H | HOXD10 | POU2F2 | ZNF145 | ZNF228 |
| CXorf6 | HSF2BP | RFX5 | ZNF146 | ZNF24 |
| EYA1 | ID3 | RPF-1 | ZNF154 | ZNF265 |
| FOXH1 | LHX2 | RRN3 | ZNF180 | ZNF83 |
| GTF3C5 | PGR | TADA3L | ZNF215 | ZNFN1A1 |
| HCF2 | PKNOX1 | TEAD1 | ZNF225 | ZNF-U69274 |
|  |  |  |  |  |
| 3 HR EXPRESSION - 12 GENES | | | | |
| ERG | KIAA0164 | NYCM | VSX1 |  |
| EYA4 | KIAA0296 | RUNX1 | ZNF123 |  |
| HOXB1 | KIAA0535 | VAX2 | ZNF19 |  |
|  |  |  |  |  |
| 30 MIN - 1 HR CO-EXPRESSION - 45 GENES | | | | |
| ALX3 | CRX | GTF3C1 | KLF3 | NR2C1 |
| ALY | E2F1 | HNF3G | KRML | PAF65B |
| AR | E2F3 | HOXA7 | LOC51173 | REQ |
| ATF2 | EGR1 | IRF6 | MAD | RNF10 |
| ATF6 | ELK3 | ISL1 | MECP2 | SOX10 |
| BACH1 | FLJ10734 | KIAA0132 | MGC15716 | SPI1 |
| BS69 | FOXF2 | KIAA0478 | MLL | TBX21 |
| CDK7 | GATA3 | KIAA1431 | MYBL2 | XBP1 |
| CEBPE | GTF3A | KLF12 | NEUROD1 | ZNF23 |
|  |  |  |  |  |
| 1 HR - 2 HR CO-EXPRESSION - 53 GENES | | | | |
| ADNP | KIAA0669 | PBX2 | SUPT6H | TRPS1 |
| BRD7 | KIAA0798 | PBX3 | TADA2L | UBP1 |
| BRDT | KIAA0961 | PCAR | TAF1C | ZNF125 |
| CBX6 | KIAA1321 | PIAS3 | TBP | ZNF133 |
| CRSP8 | LAF4 | PMF1 | TCF12 | ZNF16 |
| E2F5 | MLLT2 | RNF15 | TIF1 | ZNF173 |
| ETS1 | MYOD1 | SCML2 | TIF1GAMMA | ZNF193 |
| EVX1 | NFE2L3 | SHARP | TP73 | ZNF278 |
| FLJ23309 | NMI | SHOX | TRAP150 | ZNF33A |
| GCMB | NR1D1 | SSRP1 | TRIP13 |  |
|  |  |  |  |  |
| 2 HR - 3 HR CO-EXPRESSION - 16 GENES | | | | |
| C1orf2 | RELB | ZF5128 | ZNF237 |  |
| HOXA13 | SOX3 | ZFP103 | ZNF259 |  |
| HOXB2 | TEF | ZFP161 | ZNF35 |  |
| KIAA1442 | TFAP2C | ZNF141 | ZNF9 |  |
|  |  |  |  |  |
| 30 MIN - 3 HR CO-EXPRESSION - 8 GENES | | | | |
| ELF1 | GTF2B | HOXA11 | KLF13 |  |
| GLI | GTF2H3 | HOXA2 | NEUROD6 |  |
|  |  |  |  |  |
| 30 MIN - 2 HR CO-EXPRESSION - 14 GENES | | | | |
| ARNTL | EPLIN | OTX2 | SOX13 | TAF2J |
| CEBPG | ETS2 | SNAPC1 | STAT4 | TCF17 |
| DED | FLJ20729 | SNAPC2 | TAF2I |  |
|  |  |  |  |  |
| 1 HR - 3 HR CO-EXPRESSION - 10 GENES | | | | |
| CRSP3 | EYA3 | GTF3C3 | PBX1 | ZNF137 |
| DATF1 | FLJ12457 | NR1H2 | SMARCA2 | ZNF74 |
|  |  |  |  |  |
| 30 MIN - 1 HR - 2 HR CO-EXPRESSION - 62 GENES | | | | |
| BNC | FOXF1 | NR2E1 | PRDM14 | ZFP37 |
| BTF3L1 | FUBP1 | NR2F1 | PRDM8 | ZNF124 |
| CLOCK | GABPB1 | NR4A3 | RXRB | ZNF136 |
| CREBBP | GTF2I | NRF | SAP30 | ZNF189 |
| CREG | HIVEP1 | NRF1 | SHOX2 | ZNF236 |
| CROC4 | HIVEP2 | PAX1 | SIX3 | ZNF286 |
| CSEN | KIAA0211 | PAX4 | SLUG | ZNF294 |
| CSRP3 | LOC51043 | PKNOX2 | TAF-172 | ZNF46 |
| DKFZP434N043 | LOC51290 | PML | TAF2D | ZNF7 |
| DKFZP564F1422 | LOC58500 | PMX2B | TAF2K | ZNF92 |
| DLX6 | MYCBP | POU1F1 | TRIP11 |  |
| E2F2 | NFKB1 | POU3F4 | USF1 |  |
| FHL2 | NR0B2 | PPARD | VDR |  |
|  |  |  |  |  |
| 1 HR - 2 HR - 3 HR CO-EXPRESSION – 54 GENES | | | | |
| ATRX | HIS1 | KLF4 | PIASX-BETA | TCF2 |
| BANP | HKR2 | LOC92283 | POU3F2 | TCF3 |
| BLZF1 | HMG2 | MAZ | PRDM9 | TFCP2 |
| CBX3 | HOXB3 | MEF2D | RBL1 | USF2 |
| CNOT8 | HSA275986 | MID1 | RFP2 | ZFP26 |
| CREB3 | ILF3 | MITF | RXRA | ZFP289 |
| CREBL2 | JMJ | MYOG | SCAND2 | ZNF22 |
| DKFZp762K2015 | KIAA0071 | NFATC4 | SMCX | ZNF232 |
| ERCC2 | KIAA0244 | NFIA | TAF1A | ZNF287 |
| ESRRB | KIAA0306 | NR5A2 | TAF2A | ZNF313 |
| GBX2 | KIAA1041 | PAX3 | TAL1 |  |
|  |  |  |  |  |
| 30 MIN - 2 HR – 3 HR CO-EXPRESSION 15 GENES | | | | |
| ETV4 | HOXD11 | NEUROD4 | TFE3 | ZFP92 |
| FLJ10891 | LMO2 | PEGASUS | TRIM22 | ZNF220 |
| FLJ20039 | MDS032 | PILB | ZFP | ZNF221 |
|  |  |  |  |  |
| 30 MIN - 1 HR - 3 HR CO-EXPRESSION – 33 GENES | | | | |
| AIB3 | E2F6 | GTF2A1 | ICSBP1 | PRDM1 |
| BAZ1A | EED | GTF2E2 | KIAA0304 | SIM2 |
| BRD2 | ETV3 | HNF3A | LMO7 | STAT5A |
| CBFA2T3 | EYA2 | HNF4A | MEF2A | ZFR |
| CTCF | FOXI1 | HOXB8 | NR4A2 | ZNF273 |
| DKFZp762M136 | FOXM1 | HOXC5 | PAX2 |  |
| E2F4 | GATA6 | HRY | POU6F1 |  |
|  |  |  |  |  |
| 30 MIN - 1 HR - 2 HR - 3 HR CO-EXPRESSION - 717 GENES | | | | |
| ABT1 | FOG2 | KLF8 | NRL | TAF2G |
| AF020591 | FOS | KLHL4 | OAZ | TAF2H |
| AF093680 | FOSB | LBP-9 | OCT11 | TAF2N |
| AF5Q31 | FOSL1 | LBX1 | OG2x | TAF2S |
| ARC | FOSL2 | LDB1 | ONECUT2 | TAL2 |
| ARIX | FOXB1 | LDB2 | OVOL1 | TBR1 |
| ARNT | FOXC1 | LDOC1 | p100 | TBX10 |
| ARNT2 | FOXC2 | LEF1 | P1P373C6 | TBX15 |
| ASH2L | FOXD1 | LHX4 | P38IP | TBX18 |
| ATBF1 | FOXE1 | LHX5 | PAF65A | TBX2 |
| ATF1 | FOXE2 | LHX6 | PAX5 | TBX3 |
| ATF3 | FOXJ1 | LHX9 | PAX6 | TBX4 |
| ATF4 | FOXL2 | LIM | PAX7 | TBX5 |
| ATF5 | FOXO3A | LMO1 | PAX8 | TBX6 |
| ATF7 | FOXP1 | LMO4 | PAX9 | TCEAL1 |
| ATOH1 | GABPA | LMO6 | PBX4 | TCF19 |
| BAPX1 | GATA2 | LMX1B | PC4 | TCF-3 |
| BARHL1 | GATA4 | LOC51036 | PDEF | TCF4 |
| BAZ1B | GBX1 | LOC51042 | PER1 | TCF7 |
| BAZ2A | GCMA | LOC51058 | PER2 | TCF7L2 |
| BAZ2B | GCN5L1 | LOC51087 | PER3 | TCF8 |
| BC002881 | GCN5L2 | LOC51088 | PFDN5 | TCFL1 |
| BC011982 | GFI1 | LOC51131 | PHAP1 | TCFL4 |
| BCL11A | GFI1B | LOC51132 | PIG7 | TCFL5 |
| BCL11B | GIOT-2 | LOC51186 | PITX2 | TEAD2 |
| BRD4 | GIOT-3 | LOC51193 | PLAG1 | TEAD3 |
| BRF2 | GLI2 | LOC51270 | PLAGL1 | TEL2 |
| BRPF3 | GLI3 | LOC51637 | PLAGL2 | TFAP2A |
| BTEB1 | GLIS2 | LOC51652 | PLRG1 | TFAP2B |
| BTF3 | GLP | LOC55885 | PMX1 | TFAP4 |
| BTF3L2 | GRLF1 | LOC55893 | POU2AF1 | TFDP1 |
| C11orf9 | GSH2 | LOC56270 | POU2F1 | TFDP2 |
| C21orf18 | GTF2A2 | LOC57167 | POU4F1 | TFEB |
| C5orf7 | GTF2E1 | LOC57209 | POU4F3 | TFEC |
| CBX1 | GTF2F1 | LOC57862 | POU5F1 | TGFB1I1 |
| CBX4 | GTF2H2 | LOC65243 | PP3501 | TGIF |
| CBX5 | GTF2H4 | LOC91120 | PPARA | THG-1 |
| CCT4 | GTF3C2 | LOC91614 | PPARBP | THRA |
| CDK8 | GTF3C4 | LW-1 | PPARG | THRB |
| CDR2 | H_GS165L15 | LYL1 | PPARGC1 | TIEG |
| CDX2 | HAND2 | LZLP | PRDM10 | TIEG2 |
| CE3 | HBOA | LZTR1 | PRDM11 | TIMELESS |
| CE4 | HCNGP | LZTS1 | PRDM12 | TNRC12 |
| CEBPB | HDAC1 | M6A | PRDM13 | TNRC18 |
| CERD4 | HDAC2 | M96 | PRDM15 | TNRC3 |
| CHD2 | HDAC4 | MAD4 | PRDM16 | TNRC4 |
| CHD3 | HES2 | MADH1 | PRDM2 | TNRC5 |
| CHES1 | HES7 | MADH2 | PRDM4 | TNRC6 |
| CIAO1 | HEY1 | MADH3 | PRDM5 | TNRC9 |
| CITED1 | HEY2 | MADH4 | PRDM6 | TONDU |
| CITED2 | HEYL | MADH5 | PRDM7 | TP53 |
| CL469780 | HHEX | MADH6 | PREB | TRIM15 |
| CNOT3 | HIF1A | MADH7 | PROP1 | TRIP15 |
| CNOT4 | HKR1 | MAF | PROX1 | TRIP6 |
| COPEB | HKR3 | MAFF | PSMC5 | TSC22 |
| COPS5 | H-L(3)MBT | MAFK | PTTG1IP | TZFP |
| CORO1A | HLF | MAPK8IP1 | PURA | UBTF |
| CREB1 | HLX1 | MAX | R28830_2 | UTF1 |
| CREBL1 | HLXB9 | MBLL | R32184_3 | VENTX2 |
| CRIP1 | HMG20B | MDS1 | RAI15 | WHN |
| CRIP2 | HMGIC | MED6 | RARA | WHSC1 |
| CRSP6 | HMGIY | MEF2B | RARB | YAF2 |
| CRSP7 | HMX1 | MEF2C | RARG | ZFP36 |
| CRSP9 | HNF3B | MEFV | RBBP5 | ZFP91 |
| CSDA | HOX11 | MEIS1 | RBBP9 | ZFP93 |
| CSRP1 | HOX11L | MEIS2 | RBPSUHL | ZFP95 |
| CSRP2 | HOXA1 | MEIS3 | REL | ZFPL1 |
| CTNNB1 | HOXA10 | MEOX1 | RELA | ZFY |
| CUTL1 | HOXA3 | MGC10772 | RERE | ZHX1 |
| DBP | HOXA4 | MGC11349 | REST | ZIC1 |
| DEAF1 | HOXA5 | MGC12942 | RFP | ZIC2 |
| DFKZP434E026 | HOXA6 | MGC16733 | RFX2 | ZIC3 |
| DKFZP434B0335 | HOXA9 | MGC2508 | RFX3 | ZIC4 |
| DKFZP434B195 | HOXB13 | MHC2TA | RFX4 | ZIC5 |
| DKFZP434P1750 | HOXB6 | MILD1 | RFXANK | ZID |
| DKFZp547H236 | HOXB7 | MLL2 | RGC32 | ZIM2 |
| DLX1 | HOXB9 | MLLT1 | RING1 | ZNF10 |
| DLX3 | HOXC11 | MLLT10 | RNF13 | ZNF11B |
| DLX4 | HOXC12 | MLLT3 | RNF14 | ZNF131 |
| DR1 | HOXC13 | MLLT4 | RNF2 | ZNF134 |
| DRAP1 | HOXC6 | MLLT6 | RNF24 | ZNF138 |
| DRIL1 | HOXC9 | MLLT7 | RNF3 | ZNF142 |
| DRPLA | HOXD1 | MNAT1 | RNF4 | ZNF147 |
| DSIPI | HOXD12 | MNDA | RNF8 | ZNF155 |
| DUX2 | HOXD13 | MNT | RORB | ZNF157 |
| DUX4 | HOXD8 | MORF | RORC | ZNF15L1 |
| DXYS155E | HOXD9 | MSC | RREB1 | ZNF160 |
| E4F1 | HRIHFB2122 | MSX2 | RUNX2 | ZNF161 |
| EBF | HRIHFB2436 | MTA1 | SAFB | ZNF162 |
| EDR1 | HS747E2A | MTA1L1 | SALL1 | ZNF169 |
| EGR3 | HSAJ2425 | MTF1 | SALL2 | ZNF174 |
| EGR4 | HSF1 | MYB | SALL3 | ZNF179 |
| EHF | HSF4 | MYC | SAP18 | ZNF183 |
| ELF3 | HSGT1 | MYCL1 | SATB1 | ZNF185 |
| ELF4 | HSHPX5 | MYCL2 | SBB103 | ZNF187 |
| ELF5 | HSPC018 | MYCN | SDCCAG33 | ZNF20 |
| ELK1 | HSPC189 | MYF5 | SETBP1 | ZNF200 |
| ELK4 | HSPX153 | MYF6 | SETDB1 | ZNF205 |
| EMX1 | HSSOX6 | MYT1L | SIAH2 | ZNF207 |
| EMX2 | HTLF | MYT2 | SIM1 | ZNF208 |
| EN2 | ICBP90 | NAB1 | SIX1 | ZNF21 |
| EOMES | ID1 | NAB2 | SIX2 | ZNF211 |
| EP300 | ID2 | NCOA1 | SIX4 | ZNF212 |
| ERCC6 | ID4 | NCOA3 | SIX6 | ZNF213 |
| ERF | IGHMBP2 | NCOR2 | SLB | ZNF216 |
| ESR1 | ILF1 | NEUD4 | SMARCA3 | ZNF229 |
| ESR2 | ILF2 | NEUROG1 | SMARCB1 | ZNF230 |
| ESRRA | DNAJ | NEUROG2 | SMARCC1 | ZNF234 |
| ETV1 | IPF1 | NFATC2 | SMARCC2 | ZNF239 |
| ETV2 | IRF1 | NFATC3 | SMARCE1 | ZNF25 |
| ETV5 | IRF2 | NFE2L1 | SNAI1 | ZNF254 |
| ETV6 | IRF3 | NFE2L2 | SNAPC3 | ZNF261 |
| EZH1 | IRF5 | NFIB | SNAPC4 | ZNF268 |
| FACTP140 | IRF7 | NFIC | SNAPC5 | ZNF271 |
| FHL1 | IRLB | NFIL3 | SNW1 | ZNF274 |
| FHX | IRX4 | NFKB2 | SOX11 | ZNF275 |
| FIP2 | IRX5 | NFKBIA | SOX14 | ZNF277 |
| FKHL18 | IRX7 | NFKBIB | SOX2 | ZNF281 |
| FLJ10142 | JUN | NFKBIE | SOX30 | ZNF288 |
| FLJ10211 | JUND | NFKBIL1 | SP2 | ZNF297 |
| FLJ10251 | KIAA0014 | NFKBIL2 | SP3 | ZNF304 |
| FLJ10298 | KIAA0026 | NFRKB | SP4 | ZNF306 |
| FLJ10688 | KIAA0040 | NFX1 | SPIB | ZNF32 |
| FLJ10697 | KIAA0130 | NFYA | SRCAP | ZNF361 |
| FLJ10759 | KIAA0156 | NFYB | SREBF1 | ZNF37A |
| FLJ11186 | KIAA0161 | NFYC | SREBF2 | ZNF38 |
| FLJ12517 | KIAA0173 | NHLH2 | SRF | ZNF41 |
| FLJ12525 | KIAA0194 | NKX2B | SSX1 | ZNF43 |
| FLJ12606 | KIAA0222 | NKX6A | SSX2 | ZNF44 |
| FLJ12644 | KIAA0237 | NPAS1 | SSX3 | ZNF45 |
| FLJ12827 | KIAA0293 | NPAS2 | SSX4 | ZNF6 |
| FLJ13222 | KIAA0326 | NR0B1 | SSX5 | ZNF75A |
| FLJ13590 | KIAA0414 | NR1D2 | STAT1 | ZNF76 |
| FLJ13659 | KIAA0462 | NR1H3 | STAT2 | ZNF79 |
| FLJ14549 | KIAA0469 | NR1I2 | STAT3 | ZNF8 |
| FLJ14967 | KIAA0602 | NR1I3 | STAT6 | ZNF80 |
| FLJ20244 | KIAA0700 | NR2E3 | SUPT4H1 | ZNF90 |
| FLJ20321 | KIAA0943 | NR2F2 | SUPT5H | ZNF93 |
| FLJ20531 | KIAA1388 | NR2F6 | SURB7 | ZNF-kaiso |
| FLJ20557 | KIAA1528 | NR3C2 | T | ZXDA |
| FLJ20595 | KIAA1542 | NR4A1 | TAF2B | ZXDA/B |
| FLJ22252 | KIAA1668 | NR5A1 | TAF2C1 |  |
| FLJ22301 | KLF15 | NR6A1 | TAF2E |  |
| FMR2 | KLF7 | NRIP1 | TAF2F |  |
|  |  |  |  |  |
